# Catalytic radical difluoromethoxylation of arenes and heteroarenes†
†Electronic supplementary information (ESI) available. See DOI: 10.1039/c8sc05390a


**DOI:** 10.1039/c8sc05390a

**Published:** 2019-02-11

**Authors:** Johnny W. Lee, Weijia Zheng, Cristian A. Morales-Rivera, Peng Liu, Ming-Yu Ngai

**Affiliations:** a Department of Chemistry , Institute of Chemical Biology and Drug Discovery , Stony Brook University , Stony Brook , NY 11794 , USA . Email: ming-yu.ngai@stonybrook.edu; b Department of Chemistry , University of Pittsburgh , Pittsburgh , PA 15260 , USA . Email: pengliu@pitt.edu

## Abstract

Intermolecular C–H difluoromethoxylation of (hetero)arenes remains a long-standing and unsolved problem in organic synthesis. Herein, we report the first catalytic protocol employing a redox-active difluoromethoxylating reagent **1a** and photoredox catalysts for the direct C–H difluoromethoxylation of (hetero)arenes. Our approach is operationally simple, proceeds at room temperature, and uses bench-stable reagents. Its synthetic utility is highlighted by mild reaction conditions that tolerate a wide variety of functional groups and biorelevant molecules. Experimental and computational studies suggest single electron transfer (SET) from excited photoredox catalysts to **1a** forming a neutral radical intermediate that liberates the OCF_2_H radical exclusively. Addition of this radical to (hetero)arenes gives difluoromethoxylated cyclohexadienyl radicals that are oxidized and deprotonated to afford the products of difluoromethoxylation.

## Abstract 2

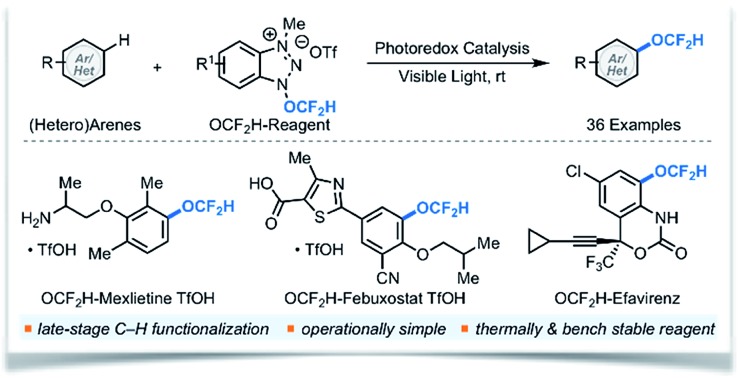
The first visible light photocatalytic generation and utilization of the OCF_2_H radical for direct (hetero)aryl C–H difluoromethoxylation at room temperature.

## Introduction

**Scheme 1:**
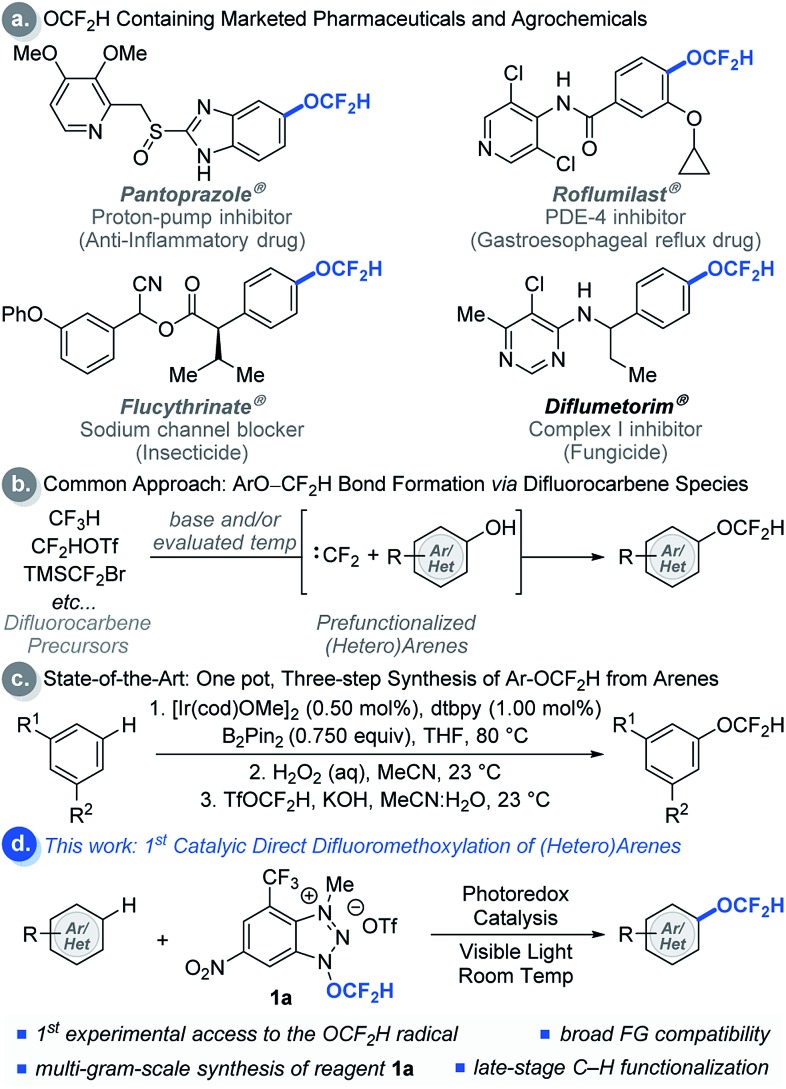
Applications and strategies for the synthesis of difluoromethoxylated (hetero)arenes.

Modern drug discovery and development involves extensive fine-tuning of physicochemical properties of drug candidates. A common approach to control these properties involves incorporation of fluorine-containing functional groups such as the difluoromethoxy (OCF_2_H) group into drug candidates.^1^ The OCF_2_H moiety is a privileged functional group in medicinal chemistry because molecules bearing the OCF_2_H group have dynamic lipophilicity, where they can adjust their lipophilicity to adapt to the chemical environment *via* simple bond rotations.^2^ In addition, OCF_2_H-containing aromatic compounds can have an orthogonal structural geometry that enriches molecular spatial complexity and provides additional binding affinity to active sites in a target.^3^ Thus, incorporation of the OCF_2_H group into organic molecules often enhances their therapeutic efficacy by increasing metabolic stability, improving cellular membrane permeability, and altering pharmacokinetic properties.^3^ As a result, the OCF_2_H group is prevalent among pharmaceuticals and agrochemicals such as Pantoprazole® (a proton-pump inhibitor that is one of the top 100 selling drugs),^4^ Roflumilast®, Flucythrinate®, and Diflumetorim® (Scheme 1a).

Even though numerous biologically active molecules have the OCF_2_H motif in an aromatic system, access to such analogues often requires the installation of the OCF_2_H group at an early stage of a multi-step synthetic sequence. The most common approach relies on *O*-difluoromethylation of phenols using different difluorocarbene precursors under basic and/or evaluated temperature conditions (Scheme 1b).^5^ This strategy has facilitated the site-selective synthesis of aryl difluoromethyl ethers, but identification of the ideal position of the OCF_2_H substitution in a drug candidate still requires parallel and laborious multi-step syntheses from aryl precursors bearing activating or directing groups at various positions in an aromatic ring. Hartwig *et al.* recently developed an elegant one-pot, three-step aryl C–H difluoromethoxylation protocol involving (i) catalytic C–H borylation of arenes, (ii) oxidation of the boronate esters, and (iii) difluoromethylation of phenols (Scheme 1c).5*h* Although this method has advanced the state-of-the-art, a catalytic, direct intermolecular C–H difluoromethoxylation of (hetero)arenes remains elusive.

As a part of our ongoing program to access and harness the reactivity of heteroatom radicals,^6^ we questioned whether a radical-mediated aromatic substitution using the OCF_2_H radical would allow direct introduction of the OCF_2_H group to a drug candidate generating multiple regioisomers in a single chemical operation. Such an approach is appealing because it obviates the need for laborious synthetic effort and the pre-functionalization of aromatic compounds. Moreover, the preparation and isolation of regioisomers would allow rapid assays of the biological activity of OCF_2_H analogues, a feature which would be particularly beneficial to modern drug discovery programs. Herein, we report the development of redox-active difluoromethoxylating reagents for late-stage, direct difluoromethoxylation of unactivated arenes and heteroarenes through a radical-mediated mechanism under visible light photocatalytic conditions at room temperature (Scheme 1d).^7^–^9^

## Results and discussion

A key to the success of the proposed transformation is the ability to generate and trap the OCF_2_H radical under mild reaction conditions. Although computational studies of the OCF_2_H radical have been reported, experimental access to such a radical intermediate remains rare.^10^ We envision that the ability to generate the OCF_2_H radical in a controllable, catalytic, and selective manner under mild conditions will open a new reaction platform for the preparation of an important class of difluoromethoxylated molecules. Our recent success in the development of trifluoromethoxylating reagents by taking advantage of the weak N–O bond (Δ*G*_N–O_ ≈ 57 kcal mol^–1^)^6^,^11^ prompted us to question whether we could develop difluoromethoxylating reagents for the first photocatalytic formation and utilization of the OCF_2_H radical in organic synthesis. Thus, we synthesized and examined a series of benzotriazole-based OCF_2_H reagents (**1a**, **DR1–5**, Table 1) for direct aryl C–H difluoromethoxylation of benzene. We found that cationic nature of the reagent is critical as it enhances the oxidizing power of the reagent and undergoes a photocatalytic single electron reduction to produce a neutral radical **1a′** that liberates the OCF_2_H radical selectively. Incorporation of electron deficient substituents on the benzotriazole ring prevents the addition of the OCF_2_H radical to the reagent byproducts and improves the reaction yields (entries 1, 3–6). Further reaction optimizations revealed that the reaction works with 1 equivalent of benzene albeit with diminished yield (40%) accompanied with additional 24% of bis(difluoromethoxylated) side products (entry 7). Control experiments showed that photoredox catalysts and light are essential, but the oxygen free environment is not required (entries 8–10). It is noteworthy that reagent **1a** (mp = 153–154 °C) be prepared in a multi-gram scale and is thermally stable beyond 200 °C. Also, it can be manipulated and stored under ambient conditions without noticeable decomposition (see ESI†).

**Table 1:** Difluoromethoxylating reagent and reaction optimization^*a*^

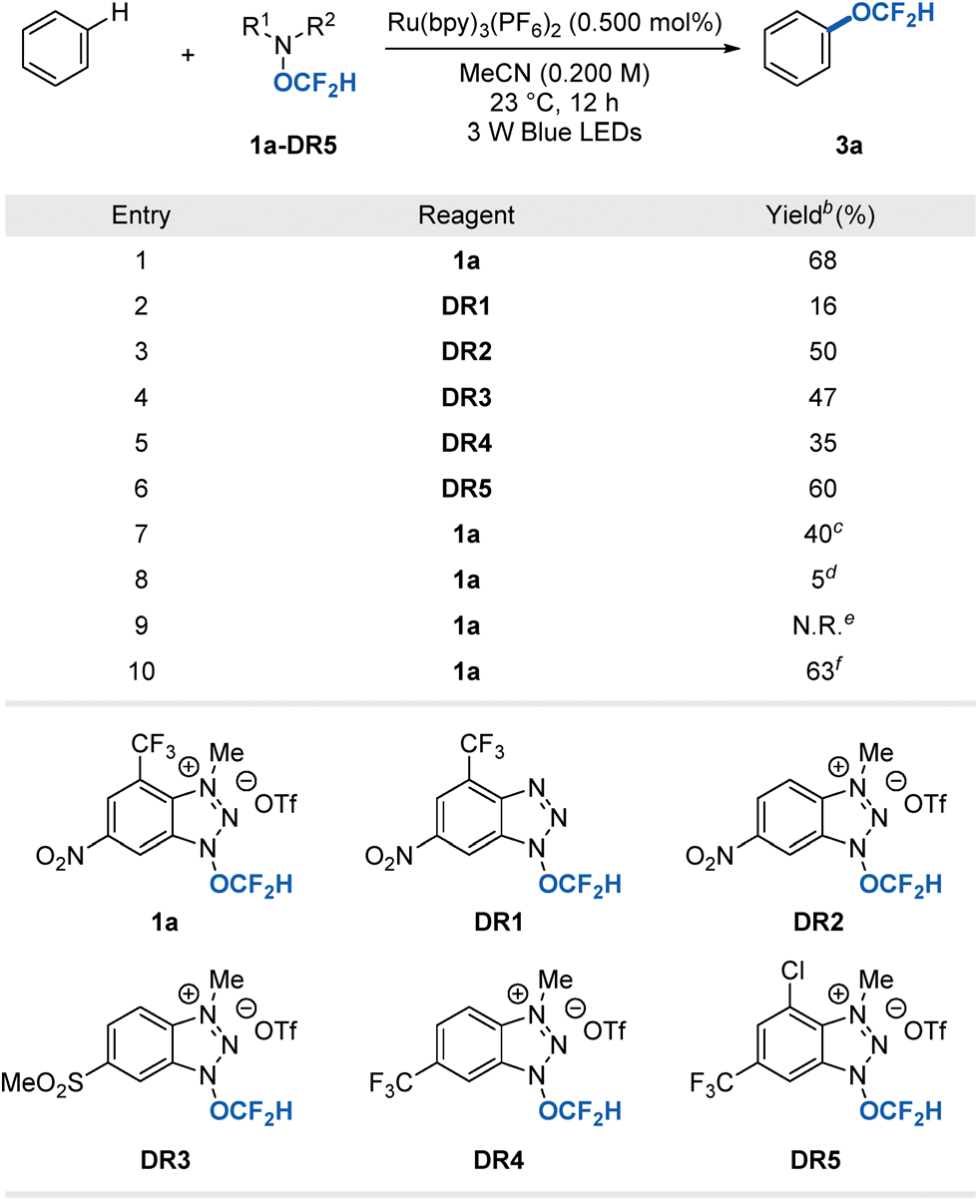

^*a*^Reactions were performed using 1 equivalent of reagent and 10 equivalents of benzene.

^*b*^Yields were determined by ^19^F NMR spectroscopy using trifluorotoluene as an internal standard.

^*c*^1 equivalent of benzene.

^*d*^Without Ru(bpy)_3_(PF_6_)_2_.

^*e*^Without light.

^*f*^The reaction was set-up under air atomsphere.

With the redox-active cationic difluoromethoxylating reagent **1a**^12^ and optimized photoredox-catalysed aryl C–H difluoromethoxylation reaction conditions in hand, we then test the generality of the reaction against a wide array of arenes and heteroarenes. As shown in Table 2, a broad array of arenes and heteroarenes with diverse electronic properties and substitution patterns underwent photocatalytic (hetero)aryl C–H difluoromethoxylation under optimized reaction conditions using reagent **1a** at room temperature. The reaction tolerated halide substituents such as fluoride (**3r**), chloride (**3b–3d**), and bromide (**3e**, **3f**, **3ab–3ad**), which is important from a synthetic perspective since these substituents provide useful handles for further structural elaboration through metal-catalysed coupling reactions. The weak benzylic C–H bond (BDE ≈ 88 kcal mol^–1^, **3f–3i**),^13^ which is often a site for undesired reactivity in radical processes, proved compatible. More remarkably, unprotected alcohols (**3i**) and phenols (**3k–3n**) remained intact during the reaction. Carbonyl derivatives such as aldehydes (**3n**), ketones with or without enolizable protons (**3o**, **3p**), carboxylic acids (**3r**, **3s**, **3ad**), esters (**3q**), amides (**3x**), and carbonates (**3z**) reacted smoothly to afford the desired products in good yields. Other functional groups such as trifluoromethyl (**3d**), methoxy (**3q**), trifluoromethoxy (**3x**), cyano (**3j**, **3k**, **3ac**), nitro (**3l**, **3m**), sulfonyl (**3y**), and pyridinium (**3v**) were all well tolerated under the reaction conditions. Moreover, no competing radical addition to electron deficient olefin (**3m**) or alkyne (**3t**) was observed during the aryl difluoromethoxylation reaction. Heteroarenes such as pyridine (**3aa**) and thiophene (**3ab–3ad**) derivatives were also viable substrates. The reaction proceeded with one equivalent of arenes, but higher yields were obtained using ten equivalents of arenes.^12^ In such cases, we could recover 8.3–9.1 equivalents (see ESI†) of the aromatic substrates at the end of the reaction, which is critical for valuable aromatic compounds.

**Table 2:** Selected examples of difluoromethoxylation of (hetero)arenes^*a*^

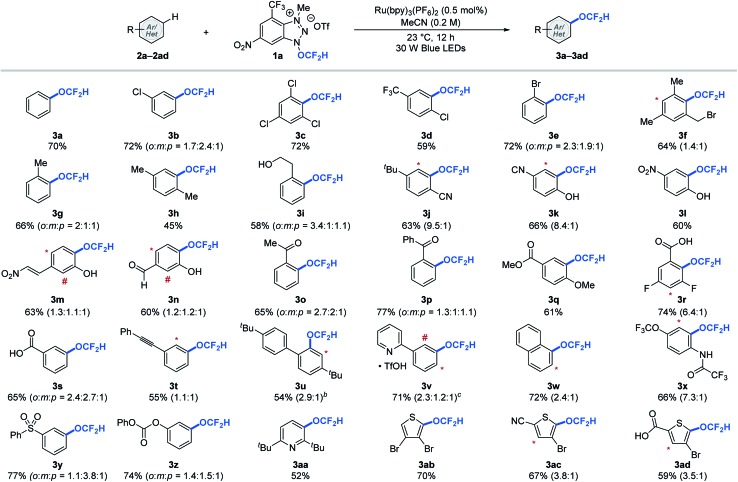

^*a*^Reactions were performed using 1.0 equivalent of reagent **1a** and 10.0 equivalents of (hetero)arene. The asterisk (*) and number sign (#) denote functionalization of minor regioisomeric products. Overall yields and the ratio of the constitutional isomers were determined by ^19^F NMR spectroscopy using trifluorotoluene as an internal standard.

^*b*^Reaction performed with MeCN and CH_2_Cl_2_ (1 : 1, 0.2 M).

^*c*^Reaction performed with 10.0 equiv. of TfOH. See ESI for experimental details.

Late-stage modifications of biologically active molecules are often a key to identification of medicinal agents.^14^ To demonstrate the amenability of the photocatalytic difluoromethoxylation processes to late-stage synthetic applications, bio-relevant molecules were subjected to our standard reaction conditions using arenes as limiting reactants (Table 3). Approved drug molecules such as Baclofen® (muscle relaxant), Febuxostat® (anti-hyperuricemic), Mexiletine® (anti-arrhythmic), Efavirenz® (antiretroviral drug for treating HIV), as well as Metronidazole® (antiparasitic) and l-menthol (decongestants and analgesics) analogues were successfully difluoromethoxylated using reagent **1a** to afford the desired products (**5a–5f**) in synthetically useful 42–76% yields, based on the recovery of the starting materials (BRSM). Our difluoromethoxylation strategy is applicable to a range of drug molecules and tolerates a number of sensitive functionalities, and this shows its potential utility in modern drug discovery programs.

**Table 3:** Selected examples of difluoromethoxylation of biorelevant molecules^*a*^

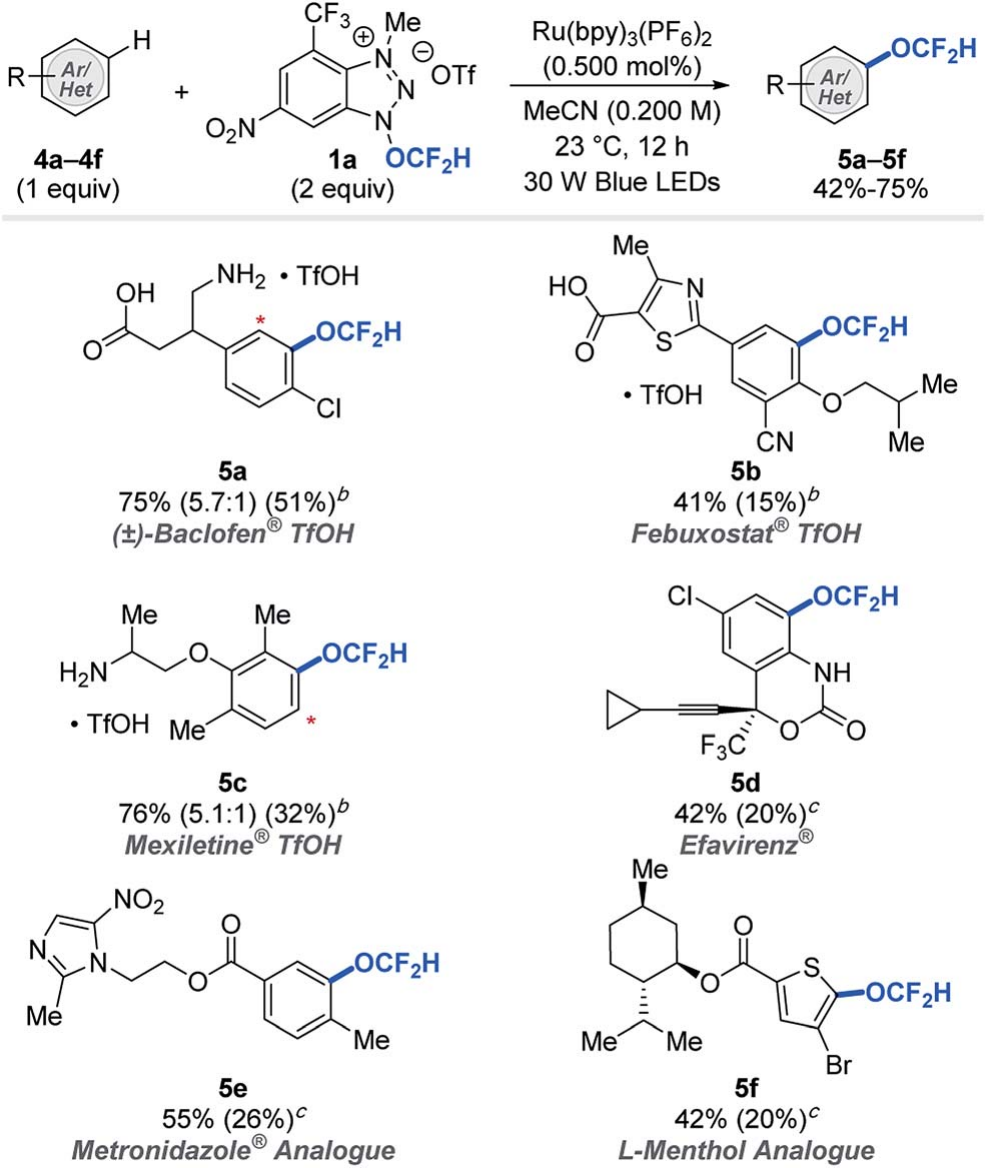

^*a*^Yields were determined based on the recovered starting material. The yield in parentheses is the isolated yield. The asterisk (*) denotes functionalization of a minor regioisomeric product.

^*b*^Reaction performed with 1.00 equivalent of TfOH.

^*c*^1.00 equivalent of K_2_CO_3_. See ESI for experimental details.

Our approach capable of forming multiple regioisomers in a single synthetic operation is complementary to the conventional site-selective protocols using phenols as substrates and could be useful in discovery chemistry. The regioselectivity of the reaction resembles that of radical-mediated aromatic substitution processes and is guided by the electronics of the substituent except in the case of a bulky substituent such as **3j**, in which case the OCF_2_H radical adds preferably to the position distal from the *tert*-butyl group. If an aromatic substrate has multiple reaction sites, the OCF_2_H radical will add to these sites to form regioisomeric products, which could be separated to provide pure isomers (see ESI†). Such reactivity is particularly attractive from a drug discovery point of view because it allows rapid access to various OCF_2_H derivatives without labour-intensive, parallel multi-step analogue synthesis.^14^,^15^ More importantly, it will increase the efficiency of structure–activity relationship (SAR) studies of OCF_2_H analogues and can conveniently produce promising new candidates that might have never been evaluated otherwise.

**Scheme 2:**
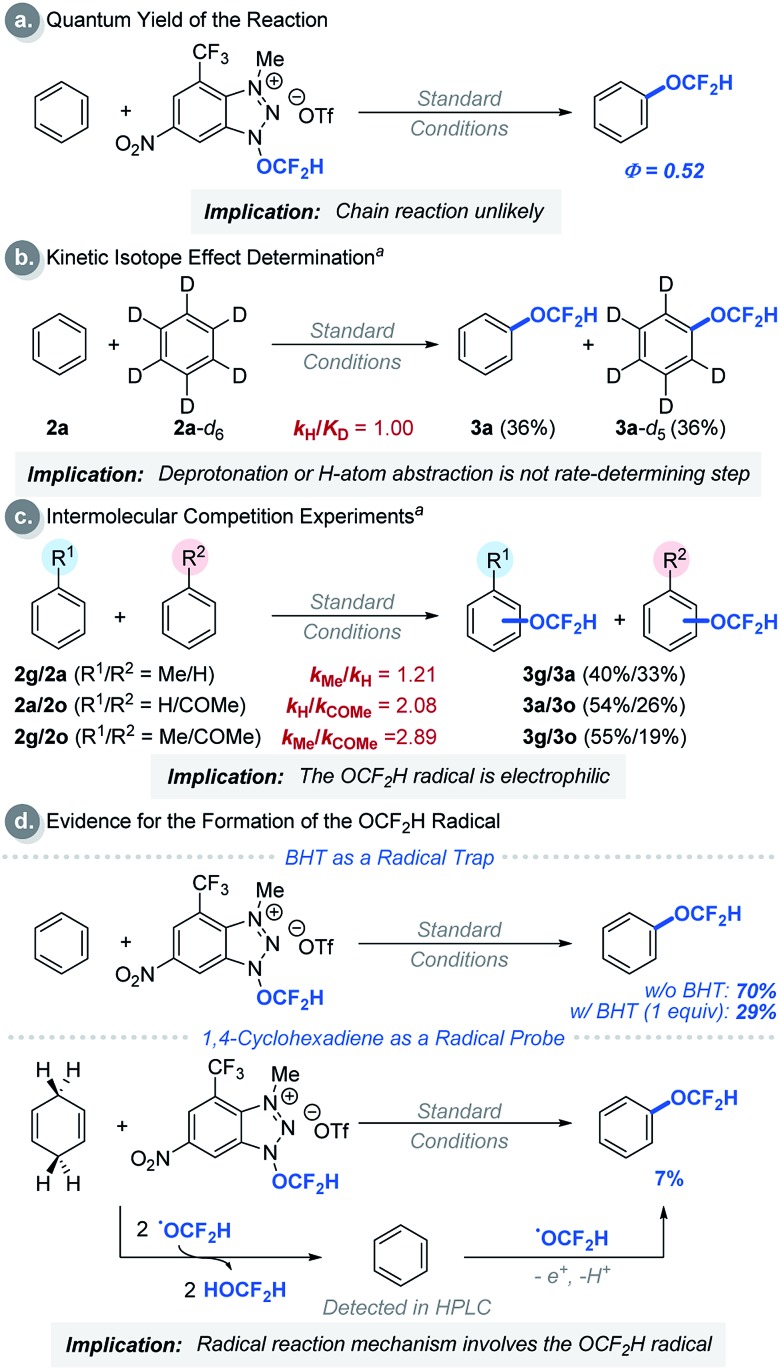
Experimental mechanism studies: ^a^reactions were performed using 5.00 equivalents of arenes each. See ESI† for experimental details.

**Scheme 3:**
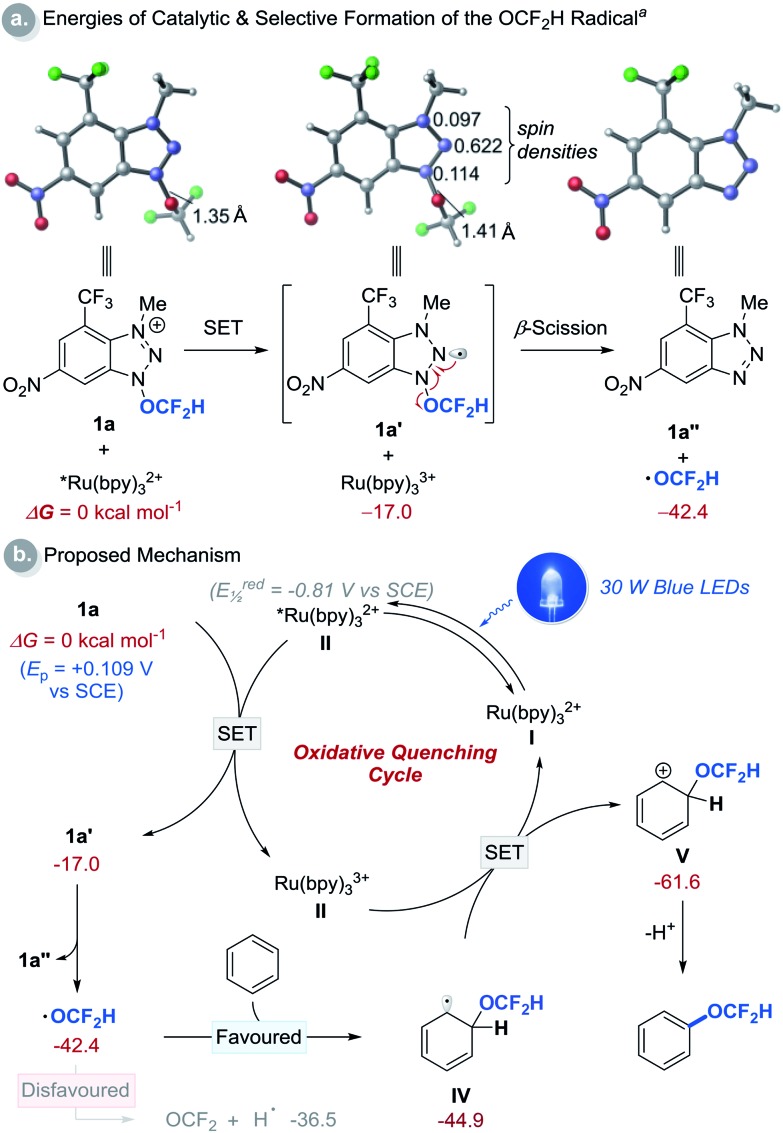
Computational studies and proposed reaction mechanism. ^a^DFT calculations were performed at the M06-2X/6-311++G(d,p)/SMD(MeCN)//M06-2X/6-31+G(d) level of theory using reagent **1a** and benzene as a substrate. All energies are in kcal mol^–1^ and are with respect to **II** and **1a**. See (ESI†) for details.

We then performed a series of experiments and DFT calculations to better understand the reactivity of the OCF_2_H radical and the reaction mechanism (Scheme 2). The quantum yield of the reaction is 0.52, which supports that an extended radical chain mechanism is unlikely. This observation corroborates DFT calculations (see Fig. S24†). A series of Stern–Volmer quenching studies showed that only **1a** quenched the excited *Ru(bpy)_3_^2+^ efficiently (*k*_q_ = 2.08 × 10^9^ M^–1^ s^–1^) (Fig. S8†). To further probe the reaction mechanism, kinetic isotope effect (KIE) experiments were conducted using a 1 : 1 mixture of benzene and *d*_6_-benzene in the presence of reagent **1a**, affording the desired products Ph-OCF_2_H and *d*_5_-Ph-OCF_2_H in a 1 : 1 ratio (Scheme 2b). This result excludes the possibility of H-atom abstraction/deprotonation as the rate-determining step. Moreover, intermolecular competition experiments using two electronically diverse arenes revealed that the OCF_2_H radical reacts more favourably with electron-rich arenes, and this confirms its electrophilic character (Scheme 2c). The formation of the OCF_2_H radical is the key for the success of the (hetero)aryl C–H difluoromethoxylation and is supported by (i) the regioselectivity of the reaction, and (ii) radical trap experiments using butylated hydroxytoluene (BHT) and 1,4-cyclohexadiene (Scheme 2d). Addition of 1 equivalent of BHT to the reaction mixture lowered the product yield from 70% to 29%. When 1,4-cyclohexadiene was used as a substrate, we observed the formation of the desired product **3a** in 7% yield. Presumably, once the OCF_2_H radical is formed, it undergoes two consecutive H-atom abstraction from 1,4-cyclohexadiene, generating benzene as the product. Subsequently, this benzene can react with the OCF_2_H radical under photocatalytic conditions, furnishing the difluoromethoxylated product. A key feature of our cationic redox-active reagent **1a** is its susceptibility to single electron reduction to form a neutral radical (**1a′**) that undergoes β-scission liberating the OCF_2_H radical exclusively (Scheme 3a). DFT calculations showed that both steps are energetically favourable in the presence of an excited photoredox catalyst, *Ru(bpy)_3_^2+^. Once the OCF_2_H radical is formed, the subsequent steps (*i.e.*, the addition of the OCF_2_H radical to an arene, oxidation of the resulting cyclohexadienyl radical by Ru(bpy)_3_^3+^, and deprotonation) are all exergonic (Fig. S24†). We have determined the peak potential of reagent **1a** [*E*_p_(**1a^+^**/**1a**) = +0.109 V *versus* saturated calomel electrode (SCE) in MeCN, Fig. S6†], and so it can be reduced by the excited *Ru(bpy)_3_^2+^ (*E*red1/2 = –0.81 V *versus* SCE in MeCN).^16^

Based on these preliminary results, a catalytic cycle of this transformation was hypothesized and depicted in Scheme 3b. Initial excitation of the Ru(bpy)_3_^2+^ photocatalyst (**I**, bpy = 2,2′-bipyridine) produces the long-lived triplet-excited state of *Ru(bpy)_3_^2+^ (**II**, *t*_1/2_ = 1.1 μs).^17^ This catalyst (**II**) (*E*red1/2 = –0.81 V *versus* SCE in MeCN)^16^ undergoes SET with the redox-active cationic reagent **1a** (*E*_p_ of **1a** = +0.109 V *versus* SCE in MeCN) generating Ru(bpy)_3_^3+^ and neutral radical **1a′** that undergoes β-scission to liberate benzotriazole (**1a′′**) and the OCF_2_H radical. The addition of this radical to an arene to form cyclohexadienyl radical **IV** is thermodynamically more favourable than the decomposition of the OCF_2_H radical to fluorophosgene and hydrogen atom.10*b* Oxidation of **IV** by Ru(bpy)_3_^3+^ (*E*red1/2 = +1.28 V, *versus* SCE in MeCN) affords cyclohexadienyl cation **V**, which is deprotonated to give the desired C–H difluoromethoxylated arenes.

## Conclusions

In summary, we have developed a redox-active cationic reagent **1a** and identified photocatalytic conditions that allow facile difluoromethoxylation of arenes and heteroarenes without the need for aryl ring pre-functionalization or pre-activation. This radical-based aromatic substitution process provides rapid access to multiple regioisomers in a single synthetic operation, which will facilitate molecular screening and SAR studies of OCF_2_H analogues. The synthetic utility of our strategy has been highlighted by the late-stage difluoromethoxylation of bio-relevant molecules at ambient temperature and pressure. Notably, this report not only provides the first experimental access to and utilization of the OCF_2_H radical but also establishes the first photocatalytic and selective formation of the OCF_2_H radical. We expect that this reagent and protocol will create a new avenue for the design and development of difluoromethoxylation reactions of hydrocarbons to aid the discovery and synthesis of new pharmaceuticals.

## Conflicts of interest

The authors declare no conflict of interest.

## Supplementary Material

Supplementary information
