# Supplementary material for: Catalytic radical difluoromethoxylation of arenes and heteroarenes
Source: Chem Sci. 2019 Feb 11;10(11):3217–22. doi: 10.1039/c8sc05390a (PMC6429774; doi:10.1039/c8sc05390a)
Supplement: Supplementary file 1 [file SC-010-C8SC05390A-s001.pdf]

Electronic Supplementary Material (ESI) for Chemical Science.
This journal is © The Royal Society of Chemistry 2019

Supporting Information

Catalytic Radical Difluoromethoxylation of Arenes and Heteroarenes

Johnny W. Lee,[†] Weijia Zheng,[†] Cristian A. Morales-Rivera,[‡] Peng Liu,*[‡] and Ming-Yu Ngai*[†]
ming-yu.ngai@stonybrook.edu
pengliu@pitt.edu

[†]Department of Chemistry and Institute of Chemical Biology and Drug Discovery, State University of New York, Stony Brook, NY 11794, USA

[‡]Department of Chemistry, University of Pittsburgh, Pittsburgh, PA, 15260, USA

Table of Contents

General Information **4**

HPLC Purification Methods **5**

Reaction Set-Up **5**

Reaction Set-Up at 23 °C 5

Difluoromethoxylation of Arenes and Heteroarenes **6**

Reaction Optimization 6

Reagent Synthesis 9

6-Nitro-4-(trifluoromethyl)-1H-benzo[d][1,2,3]triazol-1-ol (S1a) 9

1-(Difluoromethoxy)-6-nitro-4-(trifluoromethyl)-1H-benzo[d][1,2,3]triazole (DR1) 9

1-(Difluoromethoxy)-3-methyl-6-nitro-4-(trifluoromethyl)-1H-benzo[d][1,2,3]triazol-3-ium trifluoromethanesulfonate (1a) 10

1-(Difluoromethoxy)-3-methyl-6-nitro-1H-benzo[*d*][1,2,3]triazol-3-ium trifluoromethanesulfonate (DR2) 11

1-(Difluoromethoxy)-3-methyl-6-(methylsulfonyl)-1*H*-benzo[*d*][1,2,3]triazol-3-ium trifluoromethanesulfonate (DR3) 11

1-(Difluoromethoxy)-3-methyl-6-(trifluoromethyl)-1*H*-benzo[*d*][1,2,3]triazol-3-ium trifluoromethanesulfonate (DR4) 12

4-Chloro-1-(difluoromethoxy)-3-methyl-6-(trifluoromethyl)-1*H*-benzo[*d*][1,2,3]triazol-3-ium trifluoromethanesulfonate (DR5) 13

General Procedure A: Drifluoromethoxylation of (Hetero)Arenes 14

General Procedure B: Difluoromethoxylation of Complex Substrates 14

(Difluoromethoxy)benzene (3a) 15

1-Chloro-3-(difluoromethoxy)benzene (3b) 15

1,3,5-Trichloro-2-(difluoromethoxy)benzene (3c) 16

1-Bromo-2-(difluoromethoxy)benzene (3e) 16

1-Chloro-2-(difluoromethoxy)-4-(trifluoromethyl)benzene (3d) 17

1-(Bromomethyl)-2-(difluoromethoxy)-3,5-dimethylbenzene (3f) 17

1-(Difluoromethoxy)-2-methylbenzene (3g) 18

2-(Difluoromethoxy)-1,4-dimethylbenzene (3h) 18

2-(2-(Difluoromethoxy)phenyl)ethan-1-ol (3i) 19

4-(*tert*-Butyl)-2-(difluoromethoxy)benzonitrile (3j) 19

3-(Difluoromethoxy)-4-hydroxybenzonitrile (3k) 20

2-(Difluoromethoxy)-4-nitrophenol (3l) 20

(*E*)-2-(Difluoromethoxy)-5-(2-nitrovinyl)phenol (3m) 21

4-(Difluoromethoxy)-3-hydroxybenzaldehyde (3n) 21

1-(2-(Difluoromethoxy)phenyl)ethan-1-one (3o) 22

(2-(Difluoromethoxy)phenyl)(phenyl)methanone (3p) 22

1-(Difluoromethoxy)-3-(phenylethynyl)benzene (3t) 23

Methyl 3-(difluoromethoxy)-4-methoxybenzoate (3q) 24

2-(Difluoromethoxy)-3,5-difluorobenzoic acid (3r) 24

3-(Difluoromethoxy)benzoic acid (3s) 25

4,4'-Di-*tert*-butyl-2-(difluoromethoxy)-1,1'-biphenyl (3u) 25

2-(3-(Difluoromethoxy)phenyl)pyridine (3v) 26

1-(Difluoromethoxy)naphthalene (3w) 27

N-(2-(Difluoromethoxy)-4-(trifluoromethoxy)phenyl)-2,2,2-trifluoroacetamide (3x) 27

1-(Difluoromethoxy)-3-(phenylsulfonyl)benzene (3y) 28

3-(Difluoromethoxy)phenyl phenyl carbonate (3z) 28

2,6-Di-*tert*-butyl-3-(difluoromethoxy)pyridine (3aa) 29

3,4-Dibromo-2-(difluoromethoxy)thiophene (3ab) 29

4-Bromo-5-(difluoromethoxy)thiophene-2-carbonitrile (3ac) 30

4-Bromo-5-(difluoromethoxy)thiophene-2-carboxylic acid (3ad) 30

4-Amino-3-(4-chloro-2-(difluoromethoxy)phenyl)butanoic acid di-trifluoroacetic acid (5a) 31

2-(3-cyano-5-(Difluoromethoxy)-4-isobutoxyphenyl)-4-methylthiazole-5-carboxylic acid trifluoroacetic acid (5b) 32

1-(3-(Difluoromethoxy)-2,6-dimethylphenoxy)propan-2-amine (5c) 32

(S)-6-Chloro-4-(cyclopropylethynyl)-8-(difluoromethoxy)-4-(trifluoromethyl)-1,4-dihydro-2*H*-benzo[*d*][1,3]oxazin-2-one (5d) 33

2-(2-Methyl-5-nitro-1*H*-imidazol-1-yl)ethyl 3-(difluoromethoxy)-4-methylbenzoate (5e) 33

(1*R*,2*S*,5*R*)-2-Isopropyl-5-methylcyclohexyl 4-bromo-5-(difluoromethoxy)thiophene-2-carboxylate (5f) 34

Physical Properties Studies 35

Differential Scanning Calorimetry (DSC) 35
Absorption and Emission Spectra 36
Electron donor-acceptor (EDA) Complexes Study 36
Cyclic Voltammetry 38
Ferrocene Standard: 0.336 V vs Ag/AgCl electrode: 38
Reagent 1a: 0.063 V vs Ag/AgCl, -0.273 V vs Fc/Fc+, (+0.109 V vs SCE): 39
Mechanistic Studies 40
Stern–Volmer Luminescence Quenching 40
Quantum Yield Experiment 41
Determination of Fraction of Light Absorbed at 450 nm: 41
Determination of the Light Intensity at 450 nm: 42
Determination of Quantum Yield: 43
Light On/Off Experiment 44
Reaction without $Ru(bpy)_3^{2+}$ 45
Bandpass Filter Experiment 45
Emission Spectrum of 30 W Blue LEDs with and without 488 nm Bandpass Filter 46
Standard Reaction using 30 W Blue LEDs with 488 nm Bandpass Filter 46
Intermolecular Kinetic Isotope Effect 47
Intermolecular Competition Experiment 48
Reaction of the OCF_2H Radical with Electron Rich and Electron Deficient Arenes: 48
Relative Reactivity of the OCF_2H Radical and the OCF_3 Radical 49
Radical Probe Experiments 49
Butylated Hydroxytoluene (BHT) as a Radical Trap 49
1,4-Cyclohexadiene as a Radical Probe 50
Density Functional Theory (DFT) Calculations 52
Computational Details 52
Energies of photocatalytic difluoromethoxylation of benzene 52
References 53
Spectroscopic Data of Difluoromethoxylation Reactions 54
Cartesian Coordinates 141

General Information

All air- and moisture-insensitive reactions were carried out under an ambient atmosphere, magnetically stirred, and monitored by thin layer chromatography (TLC) using Agela Technologies TLC plates pre-coated with 250 µm thickness silica gel 60 F254 plates and visualized by fluorescence quenching under UV light. Flash chromatography was performed on SiliaFlash® Silica Gel 40-63µm 60Å particle size using a forced flow of eluent at 0.3–0.5 bar pressure.[1] Preparative TLC was performed on Uniplate® UV254 (20 x 20 cm) with 1000 µm thickness and visualized fluorescence quenching under UV light.

All air and moisture-sensitive manipulations were performed using oven-dried glassware, including standard Schlenk and glovebox techniques under an atmosphere of nitrogen. All reaction vials were capped using green caps with F-217 PTFE liners. Diethyl ether and THF were distilled from deep purple sodium benzophenone ketyl. Acetonitrile were dried over CaH_2 and distilled. Acetonitrile was degassed *via* three freeze-pump-thaw cycles.

All deuterated solvents were purchased from Cambridge Isotope Laboratories. NMR spectra were recorded on (i) a Bruker Ascend 700 spectrometer operating at 700 MHz for ^{1}H acquisitions and 175 MHz for ^{13}C acquisitions, (ii) a Bruker 500 Advance spectrometer operating at 500 MHz, 125 MHz, and 470 MHz for ^{1}H, ^{13}C, and ^{19}F acquisitions, or (iii) a Bruker 400 Nanobay spectrometer operating at 400 MHz, 100 MHz, and 376 MHz for ^{1}H, ^{13}C, and ^{19}F acquisitions. Chemical shifts were referenced to the residual proton solvent peaks (^{1}H: $CDCl_3$, δ 7.26; $(CD_3)_2SO$, δ 2.50), solvent ^{13}C signals ($CDCl_3$, δ 77.16; $(CD_3)_2SO$, δ 39.52),[2] dissolved or external neat $PhCF_3$ (^{19}F, δ –63.3 relative to $CFCl_3$).[3] Signals are listed in ppm, and multiplicity identified as s = singlet, br = broad, d = doublet, t = triplet, q = quartet, m = multiplet; coupling constants in Hz; integration.

Absorptions were measured on a Cary 100 UV-Vis spectrophotometer from Agilent Technologies. Emission of LED was measured on a broad range spectrometer LR1-B from ASEQ instruments. Cyclic voltammetry was performed using BioLogic VSP-300 potentiostat. High-resolution mass spectra were performed at Mass Spectrometry Services at the Univ. of Illinois at Urbana-Champaign and were obtained using Waters Q-TOF Ultima ESI mass spectrometer. IR were measured on a Nicolet iS10 FT-IR spectrometer from Thermo Scientific. The appearance of each IR group is reported as: s = strong, m = medium, w = weak.

Reagents were purchased at highest quality. Liquid reagents were distilled and degassed before use. Solid reagents were used without further purification unless otherwise stated. Compounds **S2a** was prepared according to the literature procedure.[4a] Yields of trifluoromethoxylated products were calculated by ^{19}F NMR using $PhCF_3$ as an internal standard, other yields refer to purified and spectroscopically pure compounds unless otherwise noted. Concentration under reduced pressure was performed by rotary evaporation at 25 ºC at appropriate pressure.

The bluelight emitting diodes: *30 W Blue LEDs* (LEDs, 30 W Royal Blue 455nm, chip size = 45.0 x 45.0 mm) and the heat sink (diameter: 90.0 mm) were purchased from Babaoshop on eBay (https://www.ebay.com/usr/babaoshop). The DC-12V power plug adapter male female connector and *3*

Blue LEDs (5050 3528 SMD LED strip light) was purchased from GreatPrice2010 on eBay (http://stores.ebay.com/greatprice2010/).

HPLC Purification Methods

All HPLC chromatograms were generated on a Shimadzu LC-20AP system equipped with an auto injector, a fraction collector, and a UV detector (model: SPD-20A). Analytical injections were performed on a Luna® PFP(2) analytic column (size: 250 × 4.60 mm, AXIA™ Packs) with a flow rate of 0.500 mL/min. Preparative isolation were performed on a Luna® PFP(2) preparative column 100 Å (size: 250 x 21.2 mm, AXIA™ Packs) or Gemini® 5 µm NX-C18 110 Å (size: 250 x 10 mm) with a flow rate of 10.6 mL/min. The column was fitted with a column guard. Chromatograms were obtained with a solvent composition of acetonitrile in water. Chromatograms of compounds containing acid functional groups were obtained with a solvent composition of 0.100% trifluoroacetic acid in water and 0.100% trifluoroacetic acid acetonitrile.

Reaction Set-Up

Reaction Set-Up at 23 °C

A 20 mL capped vial was placed on a stir plate at ambient temperature (23 °C). Then a 30 W blue LED lamp was placed 20.0 mm from the vial.

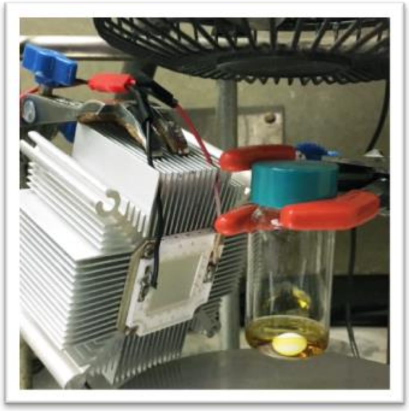

Side View

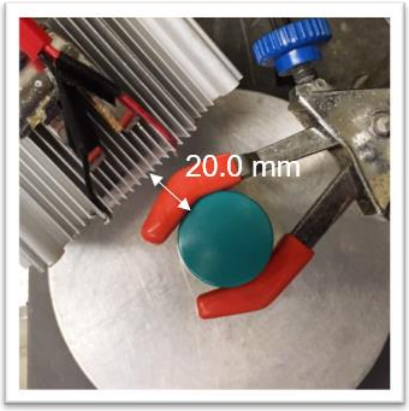

Top View

Difluoromethoxylation of Arenes and Heteroarenes

Reaction Optimization

Table S1. Photoredox Catalyst Screening

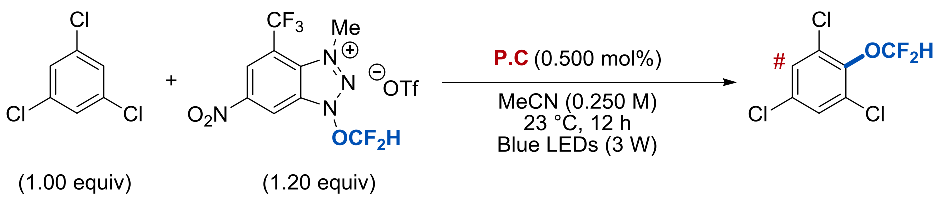

Entry	P.C	Yield (%)	Entry	P.C	Yield (%)
1	–	8	7	$Ir(ppy)_2(dtbbpy)PF_6$	40 (9:1)
2	$Ru(bpy)_3(PF_6)_2$	53 (5:1)	8	*fac*-$Ir(ppy)_3$	45 (8:1)
3	$Ru(dmb)_3(PF_6)_2$	53 (5:1)	9	*fac*-$Ir(Fppy)_3$	43 (7.6:1)
4	$Ru(dtbbpy)_3(PF_6)_2$	52 (5.5:1)	10	*fac*-$Ir(dmppy)_2(dtbbpy)$	45 (8:1)
5	$Ru(phen)_3(PF_6)_2$	49 (6:1)	11	$Ir(dtbppy)_2(dtbbpy)PF_6$	56 (4.6:1)
6	$Ru(bpz)_3(PF_6)_2$	7	12	$Ir[(dF(CF_3)ppy)_2(dtbbpy)](PF_6)$	50 (7.3:1)

[#] Position of the bis product.

Table S2. Photoredox Catalyst Loading Screening

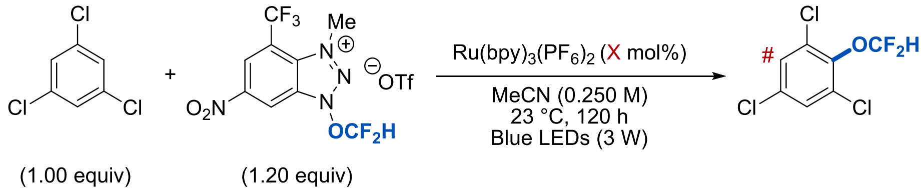

Entry	X	Yield (%)
1	1.00	52 (4.8:1)
2	0.50	54 (5.0:1)
3	0.25	52 (4.8:1)
4	0.10	56 (5.2:1)
5	0.05	59 (4.9:1)
6	0.01	47 (15:1)

Position of the bis product.

Table S3. Substrate Stoichiometry Screening

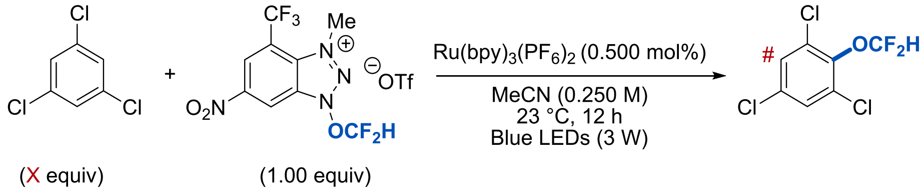

Entry	X	Yield (%)
1	1.00	48 (7:1)
2	1.20	50 (7.3:1)
3	1.50	50 (12:1)
4	2.00	53 (17:1)
5	3.00	59 (19:1)
6	5.00	61 (19:1)
7	10.0	70

Position of the bis product.

Table S4. Reagent Screening

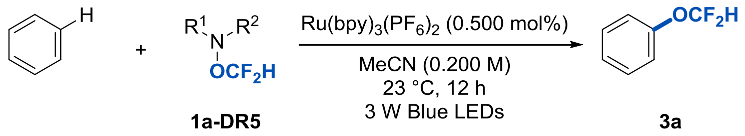

Entry	Reagent	Yield (%)[b]
1	**1a**	68
2	**DR1**	16
3	**DR2**	50
4	**DR3**	47
5	**DR4**	35
6	**DR5**	60
7	**1a**	40[c]
8	**1a**	5[d]
9	**1a**	N.R.[e]
10	**1a**	63[f]

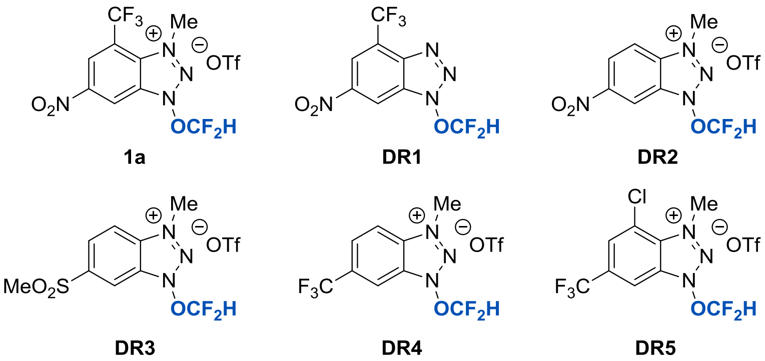

[a]Reactions were performed using 1 equivalent of reagent and 10 equivalents of benzene. [b]Yields were determined by ^{19}F NMR spectroscopy using trifluorotoluene as an internal standard. [c]1 equivalent of benzene. [d]Without $Ru(bpy)_3(PF_6)_2$. [e]Without light. [f]The reaction was set-up under air atomsphere.

Reagent Synthesis

6-Nitro-4-(trifluoromethyl)-1H-benzo[d][1,2,3]triazol-1-ol (S1a)

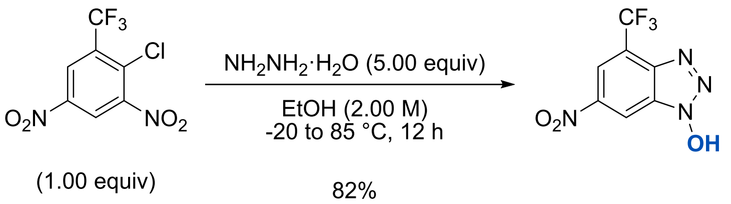

Under ambient atmosphere, to an 200 mL round bottom flask equipped a stir bar was added 2-chloro-1,5-dinitro-3-(trifluoromethyl)benzene (43.3 g, 160 mmol, 1.00 equiv and EtOH (80.0 mL, 2.00 M, with respect to the arene). The suspension was cooled to -20 °C in a cryogenic ethanol bath and hydrazine monohydrate (40.0 g, 38.8 mL, 800 mmol, 5.00 equiv) was added dropwise with an addition funnel with pressure-equalization arm. *Caution: this is an exothermic reaction, make sure the reaction flask is immersed into the cooling bath and the stirrer is stirring vigorously duing the addition.* After the addition, the reaction mixture was allowed to stir for additional 1 hour in the cooling bath before replacing the funnel with a reflux condenser. The reaction mixture was refluxed at 85 °C for 12 h, and then cooled to ambient temperature (23 °C) and concentrated in vacuo. To the reside was added 37% HCl (aq) (80 mL) and the reaction mixture was stirred for 10 min, at which point a pale brown suspension was observed. The solids were collected by filtration and washed with 1.00 M HCl (3 × 100 mL) and then DCM (3 × 100 mL). The combined solids were then dried under vacuum to afford the title compound as a pale brown solid (32.7 g, 132 mmol, 82% yield).

^{1}H NMR (700 MHz, $(CD_3)_2SO$, 25 ºC): δ 9.01 (d, J = 1.6 Hz, 1H), 8.47 (d, J = 1.6 Hz, 1H). ^{13}C NMR (175 MHz, $(CD_3)_2SO$, 25 ºC): δ 145.36, 140.16, 128.20, 122.26 (q, J = 269.7 Hz), 119.64 (q, J = 31.9 Hz), 117.58 (q, J = 5.4 Hz), 112.68. ^{19}F NMR (376 MHz, $(CD_3)_2SO$, 25 ºC): δ –60.3 (s, 3F). HRMS (ESI) *m/z* calcd for $C_7H_4N_4O_3F_3$ $[(M+H)^+]$, 249.0235, found, 249.0237. *m.p* (°C): 198–199. FT-IR (cm^{-1}): 1536 (s, N–O *asymmetric*), 1345 (s, N–O *symmetric*).

1-(Difluoromethoxy)-6-nitro-4-(trifluoromethyl)-1H-benzo[d][1,2,3]triazole (DR1)

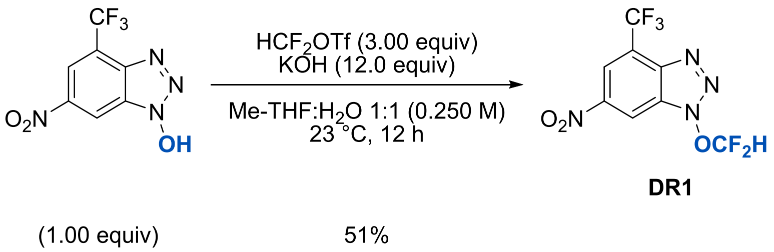

Under nitrogen atmosphere, to an oven-dried 100 mL round bottom flask equipped a stir bar was added 6-nitro-4-(trifluoromethyl)-1*H*-benzo[d][1,2,3]triazol-1-ol (**S1a**) (2.71 g, 10.0 mmol, 1.00 equiv), difluoromethyl triflate (6.00 g, 3.79 mL, 30.0 mmol, 3.00 equiv), Me-THF (24.0 mL, 0.500 M, with respect to **S1a**) and H_2O (24.0 mL, 0.500 M, with respect to **S1a**). To this solution was added 85% potassium

hydroxide (6.73 g, 120 mmol, 12.0 equiv) slowly and the reaction vial was stirred at 23 °C for 12 h. Afterwards, the reaction mixture was diluted with DCM (50 mL) and the organic layer was separated, and the aqueous layer was extracted twice with DCM (50 mL). The combined organics were dried over anhydrous Mg_2SO_4 and concentrated in vacuo. The residue was purified by column chromatography (eluting from 1 to 5% (v/v) EtOAc in Hexanes) to afford the title compound as a white solid (1.53 g, 5.14 mmol, 51% yield).

^{1}H NMR (700 MHz, $CDCl_3$, 25 ºC): δ 8.79 (s, 1H), 8.63 (s, 1H), 7.07 (t, $^2J_{HF}$ = 66.8 Hz, 1H). ^{13}C NMR (175 MHz, $CDCl_3$, 25 ºC): δ 147.47, 141.04, 129.91, 123.89 (q, J = 36.4 Hz), 121.69 (q, $^1J_{CF}$ = 273.7 Hz), 118.51 (q, J = 4.8 Hz), 117.15 (t, $^1J_{CF}$ = 279.8 Hz), 110.24. ^{19}F NMR (376 MHz, $CDCl_3$, 25 ºC): δ 61.05 (s, 3F), -87.32 (d, $^2J_{FH}$ = 67.0 Hz, 2F). HRMS (ESI) *m/z* calcd for $C_8H_4N_4O_3F_5$ [$(M+H)^+$], 249.0235, found, 249.0237. *m.p* (°C): 87–88. FT-IR (cm^{-1}): 1545 (s, N–O *asymmetric*), 1347 (s, N–O *symmetric*).

1-(Difluoromethoxy)-3-methyl-6-nitro-4-(trifluoromethyl)-1H-benzo[d][1,2,3]triazol-3-ium trifluoromethanesulfonate (1a)

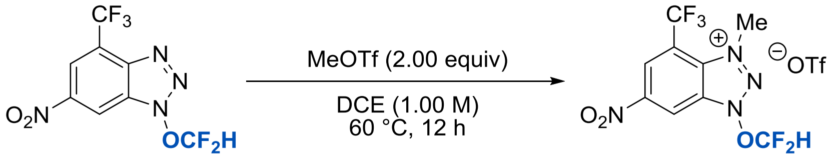

DR1 (1.00 equiv) 98% **1a**

Under nitrogen atmosphere, to an oven-dried 20 mL screw cap vial equipped a stir bar was added 1-(difluoromethoxy)-6-nitro-4-(trifluoromethyl)-1H-benzo[d][1,2,3]triazole (**DR1**) (2.15 g, 7.20 mmol, 1.00 equiv) and DCE (7.20 mL, 1.00 M, with respect to **DR1**). To this solution was added methyl trifluoromethanesulfonate (2.36 g, 1.63 mL, 0.200 mmol, 1.00 equiv) and the reaction vial was stirred at 60 °C for 12h. Afterwards the reaction vial was cooled to ambient temperature (23 °C) and a white suspension was observed. Hexanes (7.2 mL) was added the reaction vial and the solids were collected by filtration and washed with hexanes (3 × 10 mL). The combined solids were then dried in vacuo to afford the title compound as a white solid (3.27 g, 7.07 mmol, 98% yield).

^{1}H NMR (700 MHz, $(CD_3)_2SO$, 25 ºC): δ 9.92 (d, J = 1.6 Hz, 1H), 9.06 (d, J = 1.6 Hz, 1H), 8.05 (t, $^2J_{HF}$ = 64.5 Hz, 1H), 4.77 (s, 3H). ^{13}C NMR (175 MHz, $(CD_3)_2SO$, 25 ºC): δ 148.91, 133.48, 133.45, 126.33 (q, J = 6.1 Hz), 120.65 (q, $^1J_{CF}$ = 322.1 Hz), 120.64 (q, $^1J_{CF}$ = 272.8 Hz), 118.18 (t, $^1J_{CF}$ = 285.4 Hz), 116.04 (q, J = 37.2 Hz), 115.10, 42.84 (q, J = 4.6 Hz). ^{19}F NMR (376 MHz, $(CD_3)_2SO$, 25 ºC): δ -56.10 (s, 3F), -77.91 (s, 3F), -87.11 (d, $^2J_{FH}$ = 64.6 Hz, 2F). HRMS (ESI) *m/z* calcd for $C_9H_6N_4O_3F_5$ [M^+], 313.0360, found, 313.0359. *m.p* (°C): 153–154 (*melting point data is also supported DSC of reagent **1a***). FT-IR (cm^{-1}): 1564 (s, N–O *asymmetric*), 1350 (s, N–O *symmetric*).

1-(Difluoromethoxy)-3-methyl-6-nitro-1H-benzo[*d*][1,2,3]triazol-3-ium trifluoromethanesulfonate (DR2)

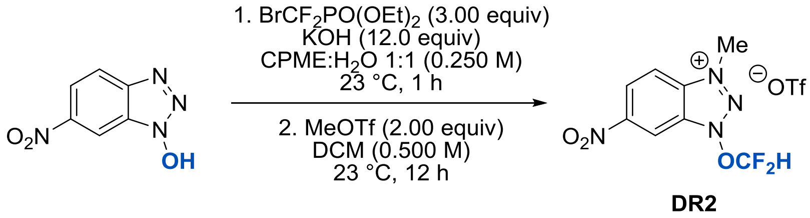

(1.00 equiv) 4%

Under nitrogen atmosphere, to an oven-dried 100 mL 100 mL round bottom flask equipped a stir bar was added 6-nitro-1H-benzo[d][1,2,3]triazol-1-ol (1.17 g, 6.50 mmol, 1.00 equiv), 85% potassium hydroxide (5.15 g, 78.0 mmol, 12.0 equiv), CPME (13.00 mL, 0.500 M, with respect to 1-hydroxy-benzotriazole) and H_2O (13.00 mL, 0.500 M, with respect to 1-hydroxy-benzotriazole). To this solution was added diethyl (bromodifluoromethyl)phosphonate (5.21 g, 3.46 mL, 19.5 mmol, 3.00 equiv) and the reaction mixture was stirred at 23 °C for 1 h. Afterwards, the reaction mixture was diluted with DCM (20 mL) and the organic layer was separated, and the aqueous layer was extracted twice with DCM (20 mL). The combined organics were dried over anhydrous Mg_2SO_4 and concentrated in vacuo. The residue was purified by column chromatography (R_f = 0.34 10% (v/v) EtOAc in Hexanes, eluting from 5 to 10% (v/v) EtOAc in Hexanes) to afford the an off-white solid. Next, the solid was dissolved in DCM (13.0 mL, 0.500 M, with respect to 1-hydroxy-benzotriazole) and to this solution was added methyl trifluoromethanesulfonate (1.28 g, 0.883 mL, 7.80 mmol, 1.20 equiv) and the reaction vial was stirred at 23 °C for 12h. Afterwards a white suspension was observed. Hexanes (5 mL) was added the reaction vial and the solids were collected by filtration and washed with Hexanes (3 × 5 mL). The combined solids were then dried in vacuo to afford the title compound as a white solid (112 mg, 0.284 mmol, 4% yield).

^{1}H NMR (700 MHz, $(CD_3)_2SO$, 25 ºC): δ 9.43 (d, *J* = 1.7 Hz, 1H), 8.83 (d, *J* = 9.5 Hz, 1H), 8.73 (dd, *J* = 1.7, 9.5 Hz, 1H), 7.99 (t, $^2J_{HF}$ = 65.0 Hz, 1H), 4.76 (s, 3H). ^{13}C NMR (175 MHz, $(CD_3)_2SO$, 25 ºC): δ 150.58, 138.04, 131.59, 127.13, 121.25 (q, $^1J_{CF}$ = 323.9 Hz), 118.73 (t, $^1J_{CF}$ = 285.2 Hz), 117.39, 110.44, 40.60. ^{19}F NMR (376 MHz, $(CD_3)_2SO$, 25 ºC): δ -77.86 (s, 3F), -87.39 (d, $^2J_{FH}$ = 65.8 Hz, 2F). HRMS (ESI) *m/z* calcd for $C_8H_7N_4O_3F_2$ [M$^+$], 245.0486, found, 245.0489. *m.p* (°C): 120–121. FT-IR (cm^{-1}): 1550 (s, N–O *asymmetric*), 1350 (s, N–O *symmetric*).

1-(Difluoromethoxy)-3-methyl-6-(methylsulfonyl)-1*H*-benzo[*d*][1,2,3]triazol-3-ium trifluoromethanesulfonate (DR3)

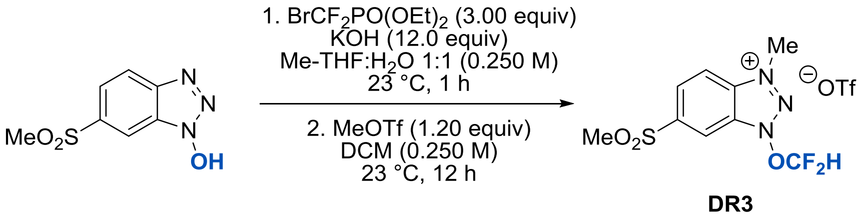

(1.00 equiv) 13%

Under nitrogen atmosphere, to an oven-dried 20 mL screw cap vial equipped a stir bar was added 6-nitro-1*H*-benzo[*d*][1,2,3]triazol-1-ol (0.640 g, 3.20 mmol, 1.00 equiv), 85% potassium hydroxide (2.38 g, 36.0 mmol, 12.0 equiv), Me-THF (6.00 mL, 0.500 M, with respect to 1-hydroxy-benzotriazole) and H_2O (6.00 mL, 0.500 M, with respect to 1-hydroxy-benzotriazole). To this suspension was added diethyl (bromodifluoromethyl)phosphonate (2.40 g, 1.60 mL, 9.00 mmol, 3.00 equiv) and the reaction vial was stirred at 23 °C for 1 h. Afterwards, the reaction mixture was diluted with DCM (10 mL) and the organic layer was separated, and the aqueous layer was extracted twice with DCM (10 mL). The combined organics were dried over anhydrous Mg_2SO_4 and concentrated in vacuo. The residue was purified by column chromatography (R_f = 0.11 60% (v/v) EtOAc in Hexanes, eluting from 30 to 80% (v/v) EtOAc in Hexanes) to afford the a white solid. Next, the solid was dissolved in DCM (12.0 mL, 0.250 M, with respect to 1-hydroxy-benzotriazole) and to this solution was added methyl trifluoromethanesulfonate (0.591 g, 0.407 mL, 3.60 mmol, 1.20 equiv) and the reaction vial was stirred at 23 °C for 12h. Afterwards a white suspension was observed. Hexanes (5 mL) was added the reaction vial and the solids were collected by filtration and washed with Hexanes (3 × 5 mL). The combined solids were then dried in vacuo to afford the title compound as a white solid (165 mg, 0.386 mmol, 13% yield).

^{1}H NMR (700 MHz, $(CD_3)_2SO$, 25 ºC): δ 9.03 (s, 1H), 8.79 (dd, *J* = 1.5, 9.0 Hz, 1H), 8.62 (dd, *J* = 1.5, 9.0 Hz, 1H), 8.01 (t, $^2J_{HF}$ = 65.3 Hz, 1H), 4.77 (s, 3H), 3.51 (s, 3H). ^{13}C NMR (175 MHz, $(CD_3)_2SO$, 25 ºC): δ 144.95, 137.23, 130.75, 129.31, 120.68 (q, $^1J_{CF}$ = 321.9 Hz), 118.21 (t, $^1J_{CF}$ = 283.4 Hz), 116.96, 113.09, 42.92. ^{19}F NMR (376 MHz, $(CD_3)_2SO$, 25 ºC): -77.86 (s, 3F), -87.31 (d, $^2J_{FH}$ = 65.3 Hz, 2F). HRMS (ESI) *m/z* calcd for $C_9H_{10}N_3O_3F_2S$ [M^+], 278.0411, found, 278.0409. *m.p* (°C): 142–143. FT-IR (cm^{-1}): 1320 (s, S–O *asymmetric*), 1135 (s, S–O *symmetric*).

1-(Difluoromethoxy)-3-methyl-6-(trifluoromethyl)-1*H*-benzo[*d*][1,2,3]triazol-3-ium trifluoromethanesulfonate (DR4)

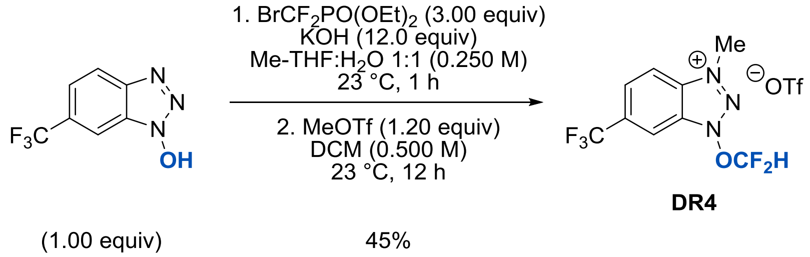

Under nitrogen atmosphere, to an oven-dried 100 mL 100 mL round bottom flask equipped a stir bar was added 6-(trifluoromethyl)-1*H*-benzo[*d*][1,2,3]triazol-1-ol (1.63 g, 8.00 mmol, 1.00 equiv), 85% potassium hydroxide (6.34 g, 96.0 mmol, 12.0 equiv), Me-THF (16.00 mL, 0.500 M, with respect to **S1e**) and H_2O (16.00 mL, 0.500 M, with respect to 1-hydroxy-benzotriazole). To this solution was added diethyl (bromodifluoromethyl)phosphonate (6.40 g, 4.26 mL, 24.0 mmol, 3.00 equiv) and the reaction mixture was stirred at 23 °C for 1 h. Afterwards, the reaction mixture was diluted with DCM (30 mL) and the organic layer was separated, and the aqueous layer was extracted twice with DCM (30 mL). The combined organics were dried over anhydrous Mg_2SO_4 and concentrated in vacuo. The residue was purified by column chromatography (R_f = 0.31 5% (v/v) EtOAc in Hexanes, eluting from 1 to 5% (v/v) EtOAc in Hexanes) to

afford the a white solid. Next, the solid was dissolved in DCM (16.0 mL, 0.500 M, with respect to 1-hydroxy-benzotriazole) and to this solution was added methyl trifluoromethanesulfonate (1.58 g, 1.09 mL, 9.60 mmol, 1.20 equiv) and the reaction vial was stirred at 23 °C for 12h. Afterwards a white suspension was observed. Hexanes (10 mL) was added the reaction vial and the solids were collected by filtration and washed with Hexanes (3 × 10 mL). The combined solids were then dried in vacuo to afford the title compound as a white solid (1.51 g, 3.61 mmol, 45% yield).

^{1}H NMR (700 MHz, $(CD_3)_2SO$, 25 ºC): δ 9.09 (s, 1H), 8.77 (dd, J = 1.3, 9.0 Hz, 1H), 8.51 (dd, J = 1.3, 9.0 Hz, 1H), 8.00 (t, $^2J_{HF}$ = 65.3 Hz, 1H), 4.77 (s, 3H). ^{13}C NMR (175 MHz, $(CD_3)_2SO$, 25 ºC): δ 137.01, 132.77 (q, $^1J_{CF}$ = 33.7 Hz), 130.84, 128.26, 122.09 (q, $^1J_{CF}$ = 273.2 Hz), 120.67 (q, $^1J_{CF}$ = 321.9 Hz), 118.18 (t, $^1J_{CF}$ = 283.1 Hz), 117.09, 111.77, 40.01. ^{19}F NMR (376 MHz, $(CD_3)_2SO$, 25 ºC): -60.98 (s, 3F), -77.88 (s, 3F), -87.42 (d, $^2J_{FH}$ = 65.3 Hz, 2F). HRMS (ESI) *m/z* calcd for $C_9H_7N_3OF_5$ [M^+], 268.0509, found, 268.0509. *m.p* (°C): 151–152.

4-Chloro-1-(difluoromethoxy)-3-methyl-6-(trifluoromethyl)-1*H*-benzo[*d*][1,2,3]triazol-3-ium trifluoromethanesulfonate (DR5)

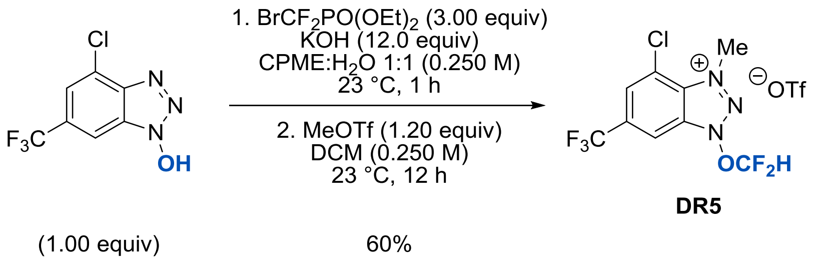

Under nitrogen atmosphere, to an oven-dried 100 mL 100 mL round bottom flask equipped a stir bar was added 4-chloro-6-(trifluoromethyl)-1*H*-benzo[*d*][1,2,3]triazol-1-ol (0.594 g, 2.50 mmol, 1.00 equiv), 85% potassium hydroxide (1.98 g, 30.0 mmol, 12.0 equiv), CPME (5.00 mL, 0.500 M, with respect to 1-hydroxy-benzotriazole) and H_2O (5.00 mL, 0.500 M, with respect to 1-hydroxy-benzotriazole). To this solution was added diethyl (bromodifluoromethyl)phosphonate (2.00 g, 1.33 mL, 7.50 mmol, 3.00 equiv) and the reaction mixture was stirred at 23 °C for 1 h. Afterwards, the reaction mixture was diluted with DCM (15 mL) and the organic layer was separated, and the aqueous layer was extracted twice with DCM (15 mL). The combined organics were dried over anhydrous Mg_2SO_4 and concentrated in vacuo. The residue was purified by column chromatography (R_f = 0.44 5% (v/v) EtOAc in Hexanes, eluting from 1 to 5% (v/v) EtOAc in Hexanes) to afford the a white solid. Next, the solid was dissolved in DCM (10.0 mL, 0.250 M, with respect to 1-hydroxy-benzotriazole) and to this solution was added methyl trifluoromethanesulfonate (0.492 g, 0.340 mL, 3.00 mmol, 1.20 equiv) and the reaction vial was stirred at 23 °C for 12h. Afterwards a white suspension was observed. Hexanes (5 mL) was added the reaction vial and the solids were collected by filtration and washed with Hexanes (3 × 5 mL). The combined solids were then dried in vacuo to afford the title compound as a white solid (0.630 g, 1.51 mmol, 60% yield).

^{1}H NMR (700 MHz, $(CD_3)_2SO$, 25 ºC): δ 9.13 (s, 1H), 8.76 (s, 1H), 8.04 (t, $^2J_{HF}$ = 64.7 Hz, 1H), 4.91 (s, 3H). ^{13}C NMR (175 MHz, $(CD_3)_2SO$, 25 ºC): δ 134.20, 133.43 (q, J = 34.5 Hz), 132.83, 128.92, 122.09

(q, $^1J_{CF}$ = 274.8 Hz), 121.97, 119.75 (q, $^1J_{CF}$ = 321.3 Hz), 118.10 (t, $^1J_{CF}$ = 284.6 Hz), 111.04 (q, J = 8.5 Hz), 42.31. ^{19}F NMR (376 MHz, $(CD_3)_2SO$, 25 ºC): -61.03 (s, 3F), -77.88 (s, 3F), -87.44 (d, $^2J_{FH}$ = 64.8 Hz, 2F). HRMS (ESI) *m/z* calcd for $C_9H_6N_3OF_5Cl$ [M^+], 302.0120, found, 302.0120. *m.p* (°C): 173–174.

General Procedure A: Drifluoromethoxylation of (Hetero)Arenes

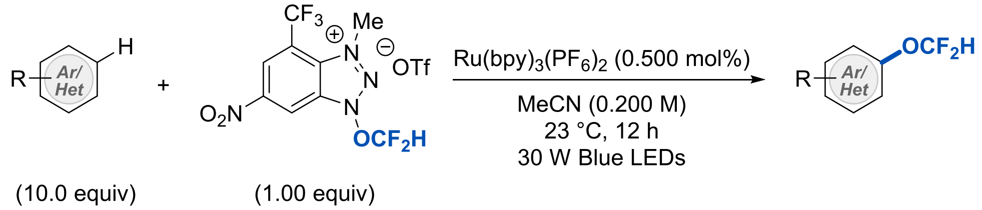

2a–2ad **1a** **3a–3ad**

In a glovebox, to an oven-dried 20 mL screw cap vial was added 1-(difluoromethoxy)-3-methyl-6-nitro-4-(trifluoromethyl)-1*H*-benzo[*d*][1,2,3]triazol-3-ium trifluoromethanesulfonate (**1a**) (92.4 mg, 0.200 mmol, 1.00 equiv), (hetero)arene (2.00 mmol, 10.0 equiv), $Ru(bpy)_3(PF_6)_2$, (0.860 mg, 1.00 µmol, 0.500 mol%), and MeCN (1.00 mL, 0.200 M, with respect to **1a**). To this suspension or solution was added a magnetic stir bar. Next, the reaction vial was capped and taken out of the glovebox. The reaction mixture was stirred at ambient temperature (23 °C) and irradiated with blue LEDs (30 W, λ_{max} = 450 nm) which was placed 20.0 mm from the vial for 12 h. To determine the yield of the products, an internal standard, trifluorotoluene ($PhCF_3$) (14.6 mg, 12.3 µL, 0.100 mmol, 0.500 equiv) was added to the vial. Then, a 100 µL of the reaction mixture was taken and then dilute with 500 µL CD_3CN followed by ^{19}F NMR (the NMR sample was recombined with the rest of the reaction mixture afterward). The combined reaction mixture was then purified by HPLC on the Luna® PFP(2) preparative column (250 × 21.2 mm) column eluting with MeCN:H_2O (v/v) with a flow rate of 10.6 mL/min to provide the purified products. In cases of closely-eluting peaks, products were isolated as a mixture of isomers. Afterwards, the products were extracted with $CDCl_3$ (3 × 1 mL), dried with magnesium sulfate, and filtered. The filtrate was concentrated in vacuo to afford the desired product(s). For very volatile compounds, the products were extracted immediately with $CDCl_3$ (1 × 1 mL) and then directly characterized. ^{1}H and ^{13}C NMR of these compound(s) contains MeCN residue signal (^{1}H NMR: δ 1.94, ^{13}C NMR: δ 118.26, 1.32 in $CDCl_3$).

General Procedure B: Difluoromethoxylation of Complex Substrates

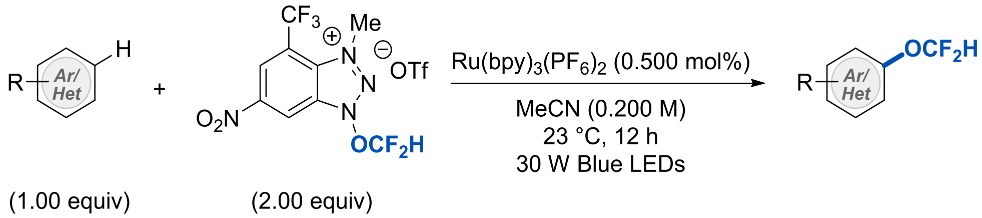

4a-4f **1a** **5a-5f**

In a glovebox, to an oven-dried 20 mL screw cap vial was added 1-(difluoromethoxy)-3-methyl-6-nitro-4-(trifluoromethyl)-1*H*-benzo[*d*][1,2,3]triazol-3-ium trifluoromethanesulfonate (**1a**) (185 mg, 0.400 mmol, 2.00 equiv), (hetero)arene (0.200 mmol, 1.00 equiv), $Ru(bpy)_3(PF_6)_2$, (0.860 mg, 1.00 μmol, 0.500 mol%), and MeCN (1.00 mL, 0.200 M, with respect to (hetero)arene). To this suspension or solution was added a magnetic stir bar. Next, the reaction vial was capped and taken out of the glovebox. The reaction mixture was stirred at ambient temperature (23 °C) and irradiated with blue LEDs (30 W, λ_{max} = 450 nm) which was placed 20.0 mm from the vial for 12 h. The combined reaction mixture was then purified by HPLC on the Luna® PFP(2) preparative column (250 × 21.2 mm) column eluting with MeCN:H_2O (v/v) with a flow rate of 10.6 mL/min to provide the purified products. In cases of closely-eluting peaks, products were isolated as a mixture of isomers. Afterwards, the product(s) was concentrated in vacuo to afford the desired product(s).

(Difluoromethoxy)benzene (3a)

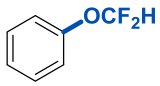

3a

Prepared according to the **General Procedure A** using benzene (156 mg, 178 μL, 2.00 mmol, 10.0 equiv) as the substrate. After 12 h, the yield was determined to be 70% by ^{19}F NMR and afterwards the samples was spiked with an authentic sample of (difluoromethoxy)benzene to confirm the product.

^{19}F NMR (376 MHz, $CDCl_3$, 25 °C): δ –82.7 (d, $^{2}J_{FH}$ = 74.5 Hz, 2F). Spectral data match those previously reported.[5]

1-Chloro-3-(difluoromethoxy)benzene (3b)

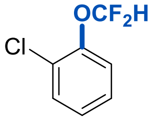

3b-*ortho*

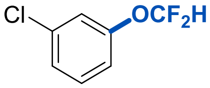

3b-*meta*

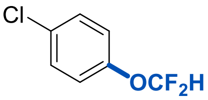

3b-*para*

Prepared according to the **General Procedure A** using chlorobenzene (225 mg, 204 μL, 2.00 mmol, 10.0 equiv) as the substrate. After 12 h, the reaction mixture (72% yield, **3b**-*ortho*:**3b**-*meta*:**3b**-*para* = 2.4:1.7:1 by ^{19}F NMR) was purified by HPLC to provide the title compound(s).

1-Chloro-2-(difluoromethoxy)benzene (3b-*ortho*) t_R = 119 min, 35% (v/v) acetonitrile in water. ^{1}H NMR (700 MHz, $CDCl_3$, 25 °C): δ 7.37 (dd, *J* = 1.5, 8.0 Hz, 1H), 7.21 (m, 1H), 7.14 (d, *J* = 8.0 Hz, 1H), 7.12 (dd, *J* = 1.5, 8.0 Hz, 1H), 6.49 (t, $^{2}J_{HF}$ = 73.6 Hz, 1H). ^{13}C NMR (175 MHz, $CDCl_3$, 25 °C): δ 146.65, 130.57, 127.88, 126.50, 125.79, 121.36, 115.72 (t, $^{1}J_{CF}$ = 261.3 Hz). ^{19}F NMR (376 MHz, $CDCl_3$, 25 °C): δ –83.1 (d, $^{2}J_{FH}$ = 73.6 Hz, 2F).

1-Chloro-3-(difluoromethoxy)benzene (3b-*meta*) and **1-chloro-4-(difluoromethoxy)benzene (3b-*para*)** t_R = 133 min, 35% (v/v) acetonitrile in water. ^{1}H NMR (700 MHz, $CDCl_3$, 25 °C): δ 7.23 (m, 2.56H), 7.11 (d, *J* = 8.1 Hz, 0.56H), 7.04 (m, 0.52H), 6.97 (d, *J* = 9.0 Hz, 2H), 6.93 (m, 0.55H), 6.48 (t, $^2J_{HF}$ = 73.4 Hz, 0.57H), 6.45 (t, $^2J_{HF}$ = 73.6 Hz, 1H). ^{13}C NMR (175 MHz, $CDCl_3$, 25 °C): δ 151.32, 149.30, 134.64, 130.51, 130.37, 129.54, 125.33, 120.75, 119.62, 117.40, 115.51 (t, $^1J_{CF}$ = 260.1 Hz), 115.42 (t, $^1J_{CF}$ = 260.3 Hz). ^{19}F NMR (376 MHz, $CDCl_3$, 25 °C): δ –83.3 (d, $^2J_{FH}$ = 73.6 Hz, 2F), –83.5 (d, $^2J_{FH}$ = 73.4 Hz, 1.12F). HRMS (EI) *m/z* calcd for $C_7H_5OF_2Cl$ [M^+], 177.9997, found, 177.9994.

1,3,5-Trichloro-2-(difluoromethoxy)benzene (3c)

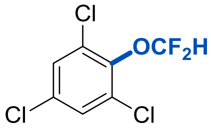

3c

Prepared according to the **General Procedure A** using 1,3,5-trichlorobenzene (363 mg, 2.00 mmol, 10.0 equiv) as the substrate. After 12 h, the reaction mixture (72% yield by ^{19}F NMR) was purified by HPLC to provide the title compound(s).

t_R = 108 min, 50% (v/v) acetonitrile in water. ^{1}H NMR (700 MHz, $CDCl_3$, 25 °C): δ 7.40 (s, 2H), 6.56 (t, $^2J_{HF}$ = 73.7 Hz, 1H). ^{13}C NMR (175 MHz, $CDCl_3$, 25 °C): δ 142.87, 132.65, 130.71, 129.32, 116.24 (t, $^1J_{CF}$ = 265.4 Hz). ^{19}F NMR (376 MHz, $CDCl_3$, 25 °C): δ –81.3 (d, $^2J_{FH}$ = 73.7 Hz, 2F). HRMS (EI) *m/z* calcd for $C_7H_3OF_2Cl_3$ [M^+], 245.9218, found, 245.9214.

1-Bromo-2-(difluoromethoxy)benzene (3e)

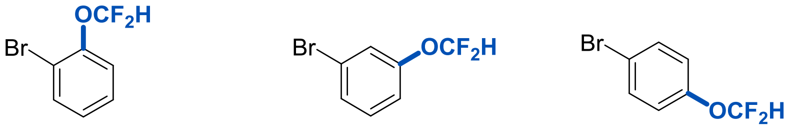

3e-*ortho* **3e**-*meta* **3e**-*para*

Prepared according to the **General Procedure A** using bromobenzene (314 mg, 211 μL, 2.00 mmol, 10.0 equiv) as the substrate. After 12 h, the reaction mixture (72% yield, **3e**-*ortho*:**3e**-*meta*:**3e**-*para* = 2.3:1.9:1 by ^{19}F NMR) was purified by HPLC to provide the title compound(s).

1-Bromo-2-(difluoromethoxy)benzene (3e-*ortho*) t_R = 83.9 min, 40% (v/v) acetonitrile in water. ^{1}H NMR (700 MHz, $CDCl_3$, 25 °C): δ 7.62 (dd, *J* = 1.4, 8.0 Hz, 1H), 7.31 (m, 1H), 7.22 (d, *J* = 8.0 Hz, 1H), 7.11 (dt, *J* = 1.4, 7.7 Hz, 1H), 6.53 (t, $^2J_{HF}$ = 73.6 Hz, 1H). ^{13}C NMR (175 MHz, $CDCl_3$, 25 °C): δ 148.09, 133.93, 128.69, 126.99, 121.67, 115.86 (t, $^1J_{CF}$ = 262.3 Hz), 115.38. ^{19}F NMR (376 MHz, $CDCl_3$, 25 °C): δ –83.0 (d, $^2J_{FH}$ = 73.6 Hz, 2F).

1-Bromo-3-(difluoromethoxy)benzene (3e-*meta*) and **1-bromo-4-(difluoromethoxy)benzene (3e-*para*)** t_R = 99.5 min, 40% (v/v) acetonitrile in water. ^{1}H NMR (700 MHz, $CDCl_3$, 25 °C): δ 7.36 (d, *J* = 8.8 Hz, 2H), 7.22 (m, 0.53H), 7.15 (m, 1.27H), 6.95 (m, 0.53H), 6.89 (d, *J* = 8.8 Hz, 1H), 6.50 (t, $^2J_{HF}$ = 73.8 Hz,

0.46H), 6.42 (t, $^2J_{HF}$ = 73.9 Hz, 1H). ^{13}C NMR (175 MHz, $CDCl_3$, 25 ºC): δ 151.25, 149.78, 132.42, 130.76, 129.79, 128.13, 122.32, 122.19, 120.96, 117.77, 115.39 (t, $^1J_{CF}$ = 259.9 Hz), 115.36 (t, $^1J_{CF}$ = 260.1 Hz). ^{19}F NMR (376 MHz, $CDCl_3$, 25 ºC): δ –83.3 (d, $^2J_{FH}$ = 73.9 Hz, 2F), –83.5 (d, $^2J_{FH}$ = 73.8 Hz, 2F). HRMS (EI) *m/z* calcd for $C_7H_5OF_2Br$ [M^+], 221.9492, found, 221.9493.

1-Chloro-2-(difluoromethoxy)-4-(trifluoromethyl)benzene (3d)

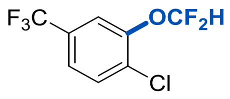

3d

Prepared according to the **General Procedure A** using 1-chloro-4-(trifluoromethyl)benzene (361 mg, 2.00 mmol, 10.0 equiv) as the substrate. After 12 h, the reaction mixture (59% yield by ^{19}F NMR) was purified by HPLC to provide the title compound(s).

t_R = 66.7 min, 50% (v/v) acetonitrile in water. ^{1}H NMR (700 MHz, $CDCl_3$, 25 ºC): δ 7.50 (d, *J* = 8.3 Hz, 1H), 7.36 (s, 1H), 7.35 (d, *J* = 8.3 Hz, 1H), 6.57 (t, $^2J_{HF}$ = 72.9 Hz, 1H). ^{13}C NMR (175 MHz, $CDCl_3$, 25 ºC): δ 146.93, 131.54, 130.19, 130.00, 123.34 (q, *J* = 6.2 Hz), 122.24 (q, $^1J_{CF}$ = 273.4 Hz), 118.31 (q, *J* = 3.5 Hz), 115.59 (t, $^1J_{CF}$ = 263.3 Hz). ^{19}F NMR (376 MHz, $CDCl_3$, 25 ºC): δ –64.7 (s, 3F), –83.8 (d, $^2J_{FH}$ = 72.9 Hz, 2F). HRMS (EI) *m/z* calcd for $C_8H_4OF_5Cl$ [M^+], 245.9871, found, 245.9871.

1-(Bromomethyl)-2-(difluoromethoxy)-3,5-dimethylbenzene (3f)

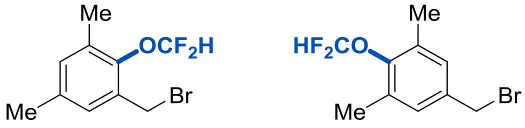

3f **3f'**

Prepared according to the **General Procedure A** using 1-(bromomethyl)-3,5-dimethylbenzene (398 mg, 2.00 mmol, 10.0 equiv) as the substrate. After 12 h, the reaction mixture (64% **3f**:**3f'** = 1.4:1 yield by ^{19}F NMR) was purified by HPLC to provide the title compound(s).

1-(Bromomethyl)-2-(difluoromethoxy)-3,5-dimethylbenzene (3f) and 5-(bromomethyl)-2-(difluoromethoxy)-1,3-dimethylbenzene (3f'): t_R = 62.8 min, 50% (v/v) acetonitrile in water. ^{1}H NMR (700 MHz, $CDCl_3$, 25 ºC): δ 2.5 (s, 2.5H), 7.00 (s, 1H), 6.47 (t, $^2J_{HF}$ = 74.6 Hz, 1H), 6.32 (t, $^2J_{HF}$ = 74.5 Hz, 0.75H), 4.53 (s, 2H), 4.41 (s, 1.5H), 2.29 (s, 3H), 2.29 (s, 2.25H), 2.28 (s, 3H). ^{13}C NMR (175 MHz, $CDCl_3$, 25 ºC): δ 148.67, 145.83, 136.77, 135.79, 133.09, 132.40, 132.23, 131.53, 130.00, 129.95, 117.49 (t, $^1J_{CF}$ = 259.5 Hz), 117.30 (t, $^1J_{CF}$ = 259.3 Hz), 32.88, 27.94, 20.84, 16.79, 16.77. ^{19}F NMR (376 MHz, $CDCl_3$, 25 ºC): δ –79.9 (d, $^2J_{FH}$ = 74.5 Hz, 1.5F), –80.1 (d, $^2J_{FH}$ = 74.6 Hz, 2F). HRMS (ESI) *m/z* calcd for $C_{10}H_{11}OF_2Br$ [M^+], 263.9961, found, 263.9960.

1-(Difluoromethoxy)-2-methylbenzene (3g)

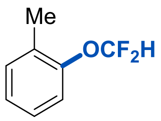

3g-*ortho*

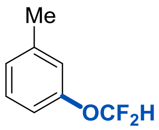

3g-*meta*

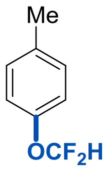

3g-*para*

Prepared according to the **General Procedure A** using toluene (184 mg, 212 μL 2.00 mmol, 10.0 equiv) as the substrate. After 12 h, the reaction mixture (66% yield, **3g**-*ortho*:**3g**-*meta*:**3g**-*para* = 2:1:1 by ^{19}F NMR) was purified by HPLC to provide the title compound(s).

1-(Difluoromethoxy)-2-methylbenzene (3g-*ortho*), 1-(difluoromethoxy)-3-methylbenzene (3g-*meta*), and **1-(difluoromethoxy)-4-methylbenzene (3g-*para*):** t_R = 85.4 min, (v/v) acetonitrile in water. ^{1}H NMR (700 MHz, $CDCl_3$, 25 °C): δ 7.26 (m, 1.10H), 7.21 (m, 1H), 7.18 (m, 1H), 7.14 (m, 1H), 7.09 (m, 1H), 7.04 (m, 1.48H), 6.95 (m, 0.82H), 6.52 (t, $^2J_{HF}$ = 74.8 Hz, 1H), 6.52 (t, $^2J_{HF}$ = 74.6 Hz, 1H), 6.49 (t, $^2J_{HF}$ = 74.8 Hz, 1H), 2.39 (s, 1.28H), 2.36 (s, 1.64H), 2.32 (s, 3H). ^{13}C NMR (175 MHz, $CDCl_3$, 25 °C): δ 151.30, 149.79, 149.02, 140.15, 135.16, 131.51, 130.27, 129.98, 129.52, 127.05, 126.14, 125.40, 120.11, 119.54, 119.03, 116.38 (t, $^1J_{CF}$ = 258.8 Hz), 116.34, 116.13 (t, $^1J_{CF}$ = 259.2 Hz), 116.06 (t, $^1J_{CF}$ = 258.8 Hz), 21.35, 20.74, 16.17. ^{19}F NMR (376 MHz, $CDCl_3$, 25 °C): δ –81.8.6 (d, $^2J_{FH}$ = 74.8 Hz, 2F), –82.4 (d, $^2J_{FH}$ = 74.6 Hz, 2F), –82.5 (d, $^2J_{FH}$ = 74.8 Hz, 2F). HRMS (EI) *m/z* calcd for $C_8H_8OF_2$ [M^+], 158.0543, found, 158.0544.

2-(Difluoromethoxy)-1,4-dimethylbenzene (3h)

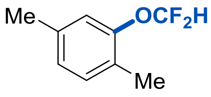

3h

Prepared according to the **General Procedure A** using *p*-xylene (212 mg, 246 μL, 2.00 mmol, 10.0 equiv) as the substrate. After 12 h, the reaction mixture (45% yield by ^{19}F NMR) was purified by HPLC to provide the title compound(s).

t_R = 49.2 min, 50% (v/v) acetonitrile in water. ^{1}H NMR (700 MHz, $CDCl_3$, 25 °C): δ 7.10 (d, *J* = 7.6 Hz, 1H), 6.92 (d, *J* = 7.6 Hz, 1H), 6.88 (s, 1H), 6.47 (t, $^2J_{HF}$ = 74.5 Hz, 1H), 2.32 (s, 3H), 2.24 (s, 3H). ^{13}C NMR (175 MHz, $CDCl_3$, 25 °C): δ 149.74, 137.21, 131.28, 126.74, 126.19, 119.82, 116.56 (t, $^1J_{CF}$ = 258.5 Hz), 21.14, 15.89. ^{19}F NMR (376 MHz, $CDCl_3$, 25 °C): δ –81.6 (d, $^2J_{FH}$ = 74.5 Hz, 2F). HRMS (EI) *m/z* calcd for $C_9H_{10}OF_2$ [M^+], 172.0700, found, 172.0701.

2-(2-(Difluoromethoxy)phenyl)ethan-1-ol (3i)

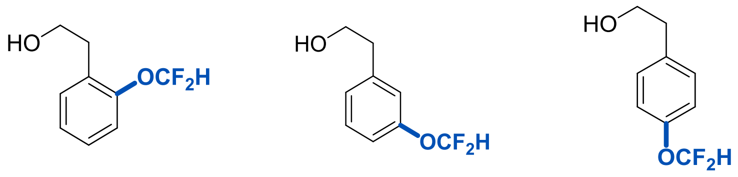

3i-*ortho* **3i**-*meta* **3g**-*para*

Prepared according to the **General Procedure A** using 2-phenylethan-1-ol (244 mg, 240 µL, 2.00 mmol, 10.0 equiv) as the substrate. After 12 h, the reaction mixture (58% yield, **3i**-*ortho*:**3i**-*meta*:**3i**-*para* = 3.4:1:1.1 by ^{19}F NMR) was purified by HPLC to provide the title compound(s).

2-(2-(Difluoromethoxy)phenyl)ethan-1-ol (3i-*ortho*), 2-(3-(difluoromethoxy)phenyl)ethan-1-ol (3i-*meta*), and 2-(4-(difluoromethoxy)phenyl)ethan-1-ol (3i-*para*): t_R = 66.4 min, 50% (v/v) acetonitrile in water. ^{1}H NMR (700 MHz, $CDCl_3$, 25 °C): δ 7.30 (m, 1.50H), 7.23 (m, 1.88H), 7.17 (m, 1H), 7.09 (m, 2.52H), 7.00 (m, 1H), 6.53 (t, $^2J_{HF}$ = 74.1 Hz, 1H), 6.51 (t, $^2J_{HF}$ = 74.1 Hz, 1H), 6.48 (t, $^2J_{HF}$ = 74.1 Hz, 1H), 3.86 (s, 1H), 2.94 (s, 1H), 2.87 (s, 1H). ^{13}C NMR (175 MHz, $CDCl_3$, 25 °C): δ 151.57, 150.10, 149.93, 141.06, 135.98, 131.65, 130.47, 130.26, 130.02, 128.13, 126.19, 125.65, 120.25, 119.95, 118.91, 117.53, 116.54 (t, $^1J_{CF}$ = 258.8 Hz), 116.12 (t, $^1J_{CF}$ = 259.5 Hz), 115.34 (d, $^1J_{CF}$ = 259.3 Hz), 63.69, 63.49, 62.67, 39.05, 38.52, 33.60. ^{19}F NMR (376 MHz, $CDCl_3$, 25 °C): δ –79.9 (d, $^2J_{FH}$ = 74.1 Hz, 2F), –80.6 (d, $^2J_{FH}$ = 74.1 Hz, 1.14F), –80.6 (d, $^2J_{FH}$ = 74.1 Hz, 1.30F). HRMS (EI) *m/z* calcd for $C_9H_{10}O_2F_2$ [M^+], 188.0649, found, 188.0649.

4-(*tert*-Butyl)-2-(difluoromethoxy)benzonitrile (3j)

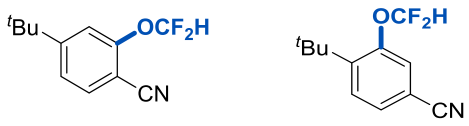

3j **3j'**

Prepared according to the **General Procedure A** using 4-(tert-butyl)benzonitrile (319 mg, 2.00 mmol, 10.0 equiv) as the substrate. After 12 h, the reaction mixture (63% yield, **3j**:**3j'** = 9.5:1 by ^{19}F NMR) was purified by HPLC to provide the title compound(s).

4-(*tert*-Butyl)-2-(difluoromethoxy)benzonitrile (3j): t_R = 103 min, 45% (v/v) acetonitrile in water. ^{1}H NMR (700 MHz, $CDCl_3$, 25 °C): δ 7.60 (d, J = 8.2 Hz, 1H), 7.34 (s, 1H), 7.33 (d, J = 8.2 Hz, 1H), 7.30 (s, 1H), 6.64 (t, $^2J_{HF}$ = 72.1 Hz, 1H), 1.33 (s, 9H). ^{13}C NMR (175 MHz, $CDCl_3$, 25 °C): δ 159.37, 151.85, 133.56, 123.30, 117.92, 115.43, 115.31 (t, $^1J_{CF}$ = 265.5 Hz), 103.45, 35.71, 30.99. ^{19}F NMR (376 MHz, $CDCl_3$, 25 °C): δ –81.8 (d, $^2J_{FH}$ = 72.1 Hz, 2F). FT-IR (cm^{-1}): 2358 (w, C≡N).

4-(*tert*-Butyl)-3-(difluoromethoxy)benzonitrile (3j'): t_R = 116 min, 45% (v/v) acetonitrile in water. ^{1}H NMR (700 MHz, $CDCl_3$, 25 °C): δ 7.49 (d, J = 8.2 Hz, 1H), 7.44 (d, J = 8.2 Hz, 1H), 7.33 (s, 1H), 6.54 (t, $^2J_{HF}$ = 72.9 Hz, 1H), 1.40 (s, 9H). ^{13}C NMR (175 MHz, $CDCl_3$, 25 °C): δ 151.00, 146.43, 128.90, 128.88, 120.98, 118.01, 116.07 (t, $^1J_{CF}$ = 259.8 Hz), 111.28, 35.67, 29.76. ^{19}F NMR (376 MHz, $CDCl_3$, 25 °C): δ –83.8 (d, $^2J_{FH}$ = 72.9 Hz, 2F). HRMS (EI) *m/z* calcd for $C_{12}H_{13}NOF_2$ [M^+], 225.0965, found, 225.0965.

3-(Difluoromethoxy)-4-hydroxybenzonitrile (3k)

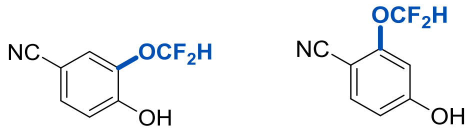

3k **3k'**

Prepared according to the **General Procedure A** using 4-hydroxybenzonitrile (238 mg, 2.00 mmol, 10.0 equiv) as the substrate. After 12 h, the reaction mixture (66% yield, **3k**:**3k'** = 8.4:1 by ^{19}F NMR) was purified by HPLC to provide the title compound(s). Afterwards, the product(s) was concentrated in vacuo to afford the desired product(s).

3-(Difluoromethoxy)-4-hydroxybenzonitrile (3k): t_R = 30.8 min, 40% (v/v) acetonitrile in water. ^{1}H NMR (700 MHz, $CDCl_3$, 25 °C): δ 7.47 (s, 1H), 7.46 (d, *J* = 8.1 Hz, 1H), 7.10 (d, *J* = 8.1 Hz, 1H), 6.60 (t, $^2J_{HF}$ = 73.8 Hz, 1H), 6.27 (br. s, 1H). ^{13}C NMR (175 MHz, $CDCl_3$, 25 °C): δ 153.72, 137.83, 131.67, 124.33, 118.14, 118.02, 115.57 (t, $^1J_{CF}$ = 265.7 Hz), 104.07. ^{19}F NMR (376 MHz, $CDCl_3$, 25 °C): δ –83.6 (d, $^2J_{FH}$ = 73.8 Hz, 2F). HRMS (ESI-TOF) *m/z* calcd for $C_8H_4F_2NO_2$ [(M – H)$^-$], 184.0216, found, 184.0215.

2-(Difluoromethoxy)-4-hydroxybenzonitrile (3k'): t_R = 76.9 min, 40% (v/v) acetonitrile in water. ^{1}H NMR (700 MHz, $CDCl_3$, 25 °C): δ 7.53 (d, *J* = 8.4 Hz, 1H), 6.81 (s, 1H), 6.77 (d, *J* = 8.4 Hz) 6.61 (t, $^2J_{HF}$ = 74.7 Hz, 1H), 6.27 (br. s, 1H). ^{13}C NMR (175 MHz, $CDCl_3$, 25 °C): δ 161.36, 153.18, 135.24, 115.57, 115.17 (t, $^1J_{CF}$ = 266.2 Hz), 113.30, 107.59, 97.13. ^{19}F NMR (376 MHz, $CDCl_3$, 25 °C): δ –84.1 (d, $^2J_{FH}$ = 74.7 Hz, 2F).

2-(Difluoromethoxy)-4-nitrophenol (3l)

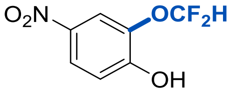

3l

Prepared according to the **General Procedure A** using 4-nitrophenol (278 mg, 2.00 mmol, 10.0 equiv) as the substrate. After 12 h, the reaction mixture (60% yield by ^{19}F NMR) was concentrated and added 1.00 M NaOH (aq) (5 mL), dichloromethane (10 mL), and extracted with dichloromethane (3 × 10 mL). Then to the aqueous layer was added 1.00 M HCl (aq) (20 mL) and extracted with ethyl acetate (5 × 20 mL), dried with $MgSO_4$ and concentrated. Then the mixture was purified by HPLC to provide the title compound(s). Afterwards, the product(s) was concentrated in vacuo to afford 21.0 mg (51% yield) of the desired product(s). And 248 mg, 89% of 4-nitrophenol was recovered. (**t_R =** 16.6 min, 40% (v/v) acetonitrile in water).

t_R = 31.3 min, 40% (v/v) acetonitrile in water. ^{1}H NMR (700 MHz, $CDCl_3$, 25 °C): δ 8.11 (d, *J* = 2.5 Hz, 1H), 8.10 (d, *J* = 8.9 Hz, 1H), 8.10 (d, *J* = 2.5 Hz, 1H), 7.14 (d, *J* = 8.9 Hz, 1H), 6.65 (t, $^2J_{HF}$ = 73.5 Hz, 1H), 6.17 (s, 1H). ^{13}C NMR (175 MHz, $CDCl_3$, 25 °C): δ 153.33, 141.09, 137.19, 123.22, 116.80, 116.17, 115.71 (t, $^1J_{CF}$ = 265.8 Hz). ^{19}F NMR (376 MHz, $CDCl_3$, 25 °C): δ –83.9 (d, $^2J_{FH}$ = 73.5 Hz, 2F). HRMS (ESI-TOF) *m/z* calcd for $C_7H_4F_2NO_4$ [(M – H)$^-$], 204.0114, found, 204.0109. FT-IR (cm^{-1}): 3420 (s, O–H).

(*E*)-2-(Difluoromethoxy)-5-(2-nitrovinyl)phenol (3m)

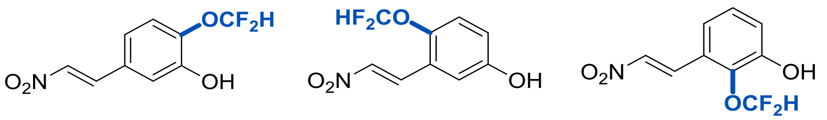

3m **3m'** **3m''**

Prepared according to the **General Procedure A** using (*E*)-3-(2-nitrovinyl)phenol (330 mg, 2.00 mmol, 10.0 equiv) as the substrate. After 12 h, the reaction mixture (63% yield, **3m**:**3m**':**3m**'' = 1.3:1.1:1 by ^{19}F NMR) was purified by HPLC to provide the title compound(s). Afterwards, the product(s) was concentrated in vacuo to afford the desired product(s).

(*E*)-2-(Difluoromethoxy)-5-(2-nitrovinyl)phenol (3m) and **(*E*)-4-(difluoromethoxy)-3-(2-nitrovinyl)phenol (3m')** and **(*E*)-2-(difluoromethoxy)-3-(2-nitrovinyl)phenol (3m''):** t_R = 70.6 min, 35% (v/v) acetonitrile in water. ^{1}H NMR (700 MHz, $CDCl_3$, 25 ºC): δ 8.16 (d, *J* = 13.7 Hz, 0.42H), 8.13 (d, *J* = 13.8 Hz, 0.42H), 7.92 (d, *J* = 13.6 Hz, 1H), 7.63 (d, *J* = 13.7 Hz, 0.42H), 7.62 (d, *J* = 13.8 Hz, 0.42H), 7.51 (d, *J* = 13.6 Hz, 1H), 7.22 (m, 1.21H), 7.19 (m, 0.89H), 7.16 (m, 1.24H), 7.10 (dd, *J* = 1.8, 8.4 Hz, 1H), 7.03 (d, *J* = 2.9 Hz, 0.42H), 6.97 (dd, *J* = 2.9, 8.9 Hz, 0.42H), 6.62 (t, $^2J_{HF}$ = 73.6 Hz, 1H), 6.61 (t, $^2J_{HF}$ = 73.4 Hz, 0.42H), 6.53 (t, $^2J_{HF}$ = 74.9 Hz, 0.42H), 5.52 (br. s, 1.78H). ^{13}C NMR (175 MHz, $CDCl_3$, 25 ºC): δ 153.41, 149.58, 147.92, 143.99, 140.91, 139.35, 139.25, 138.03, 137.58, 137.09, 133.15, 133.10, 128.64, 128.16, 125.81, 124.02, 122.52, 122.23, 120.95, 120.94, 120.24, 120.00, 116.69, 116.66 (t, $^1J_{CF}$ = 265.3 Hz), 115.87 (t, $^1J_{CF}$ = 263.3 Hz), 115.80 (t, $^1J_{CF}$ = 264.3 Hz), 115.68. ^{19}F NMR (376 MHz, $CDCl_3$, 25 ºC): δ –83.6.5 (d, $^2J_{FH}$ = 73.4 Hz, 1F), –83.0 (d, $^2J_{FH}$ = 74.9 Hz, 2F), –83.3 (d, $^2J_{FH}$ = 73.6 Hz, 2F). HRMS (ESI-TOF) *m/z* calcd for $C_9H_6F_2NO_4$ [$(M – H)^–$], 230.0270, found, 230.0274.

4-(Difluoromethoxy)-3-hydroxybenzaldehyde (3n)

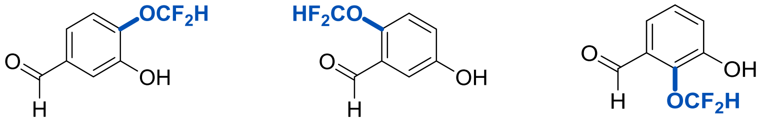

3n **3n'** **3n''**

Prepared according to the **General Procedure A** using 3-hydroxybenzaldehyde (244 mg, 2.00 mmol, 10.0 equiv) as the substrate. After 12 h, the reaction mixture (60% yield, **3n**:**3n**':**3n**'' = 1.2:1.2:1 by ^{19}F NMR) was purified by HPLC to provide the title compound(s).

4-(Difluoromethoxy)-3-hydroxybenzaldehyde (3n) and **2-(difluoromethoxy)-5-hydroxybenzaldehyde (3n'):** t_R = 57.8 min, 25% (v/v) acetonitrile in water. ^{1}H NMR (700 MHz, $CDCl_3$, 25 ºC): δ 10.30 (s, 1H), 9.92 (s, 0.50H), 7.56 (d, *J* = 1.8 Hz, 0.50H), 7.46 (m, 1H), 7.40 (m, 0.50H), 7.27 (m, 1H), 7.17 (m, 1H), 7.14 (m, 1H), 6.66 (t, $^2J_{HF}$ = 73.8 Hz, 0.50H), 6.58 (t, $^2J_{HF}$ = 74.9 Hz, 1H), 5.95 (br. s, 1.50H). ^{13}C NMR (175 MHz, $CDCl_3$, 25 ºC): δ 191.25, 189.08, 153.96, 147.86, 146.44, 143.11, 134.65, 129.09, 123.40, 123.17, 122.88, 119.31, 117.19, 115.86 (t, $^1J_{CF}$ = 263.3 Hz), 115.73 (t, $^1J_{CF}$ = 263.8 Hz), 114.10. ^{19}F NMR

(376 MHz, $CDCl_3$, 25 ºC): δ –83.5 (d, $^2J_{FH}$ = 73.8 Hz, 1F), –84.1 (d, $^2J_{FH}$ = 74.9 Hz, 2F). HRMS (ESI-TOF) *m/z* calcd for $C_8H_5F_2O_3$ [(M – H)$^-$], 187.0212, found, 187.0213.

2-(Difluoromethoxy)-3-hydroxybenzaldehyde (3n''): t_R = 55.4 min, 25% (v/v) acetonitrile in water. ^{1}H NMR (700 MHz, $CDCl_3$, 25 ºC): δ 10.17 (s, 1H), 7.45 (dd, *J* = 1.3, 7.5 Hz, 1H), 7.34 (t, *J* = 7.5 Hz, 1H), 7.31 (d, *J* = 1.3 Hz, 1H), 7.30 (d, *J* = 1.3 Hz, 1H), 6.70 (t, $^2J_{HF}$ = 73.9 Hz, 1H), 5.73 (s, 1H). ^{13}C NMR (175 MHz, $CDCl_3$, 25 ºC): δ 189.24, 149.71, 137.86, 130.24, 127.96, 123.27, 123.13, 116.89 (t, ^1J_C = 264.6 Hz). ^{19}F NMR (376 MHz, $CDCl_3$, 25 ºC): δ –83.1 (d, $^2J_{FH}$ = 73.9 Hz, 2F).

1-(2-(Difluoromethoxy)phenyl)ethan-1-one (3o)

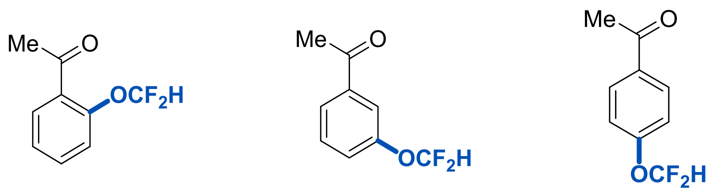

3o-*ortho* **3o**-*meta* **3o**-*para*

Prepared according to the **General Procedure A** using acetophenone (240 mg, 233 μL, 2.00 mmol, 10.0 equiv) as the substrate. After 12 h, the reaction mixture (65% yield, **3o**-*ortho*:**3o**-*meta*:**3o**-*para* = 2.7:2:1 by ^{19}F NMR) was purified by HPLC to provide the title compound(s).

1-(2-(Difluoromethoxy)phenyl)ethan-1-one (3o-*ortho*) and **1-(3-(difluoromethoxy)phenyl)ethan-1-one (3o-*para*):** t_R = 101 min, 25% (v/v) acetonitrile in water. ^{1}H NMR (700 MHz, $CDCl_3$, 25 ºC): δ 7.99 (m, 0.60H), 7.76 (m, 1H), 7.53 (m, 1H), 7.52 (m, 1H), 7.30 (m, 1H), 7.18 (m, 1.60H), 6.60 (t, $^2J_{HF}$ = 73.5 Hz, 1H), 6.60 (t, $^2J_{HF}$ = 73.0 Hz, 0.30H), 2.63 (s, 3H), 2.60 (s, 0.90H). ^{13}C NMR (175 MHz, $CDCl_3$, 25 ºC): δ 198.56, 196.61, 154.73, 149.54, 134.11, 133.42, 131.58, 130.51, 130.48, 125.68, 119.75, 118.76, 116.11 (t, $^1J_{CF}$ = 260.6 Hz), 115.33 (t, $^1J_{CF}$ = 261.5 Hz), 31.25, 26.58. ^{19}F NMR (376 MHz, $CDCl_3$, 25 ºC): δ –82.8 (d, $^2J_{FH}$ = 73.5 Hz, 2F), –84.0 (d, $^2J_{FH}$ = 73.0 Hz, 2F). Spectral data match those previously reported.[5]

1-(4-(Difluoromethoxy)phenyl)ethan-1-one (3o-*meta*) t_R = 111 min, 25% (v/v) acetonitrile in water. ^{1}H NMR (700 MHz, $CDCl_3$, 25 ºC): δ 7.80 (m, 1H), 7.70 (t, *J* = 1.7 Hz, 1H), 7.48 (t, *J* = 7.9 Hz, 1H), 7.34 (dd, *J* = 7.9, 1.7 Hz, 1H), 6.57 (t, $^2J_{HF}$ = 73.3 Hz, 1H), 2.61 (s, 3H) ^{13}C NMR (175 MHz, $CDCl_3$, 25 ºC): δ 197.01, 151.43, 138.97, 130.26, 125.52, 124.52, 119.17, 115.75 (t, $^1J_{CF}$ = 261.2 Hz), 26.85. ^{19}F NMR (376 MHz, $CDCl_3$, 25 ºC): δ –83.3 (d, $^2J_{FH}$ = 73.3 Hz, 2F). FT-IR (cm^{-1}): 1696 (s, C=O).

(2-(Difluoromethoxy)phenyl)(phenyl)methanone (3p)

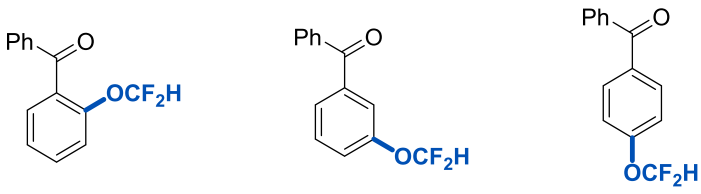

3p-*ortho* **3p**-*meta* **3p**-*para*

Prepared according to the **General Procedure A** using benzophenone (364 mg, 2.00 mmol, 10.0 equiv) as the substrate. After 12 h, the reaction mixture (77% yield, **3p**-*ortho*:**3p**-*meta*:**3p**-*para* = 1.3:1:1.4 by ^{19}F NMR) was purified by HPLC to provide the title compound(s).

(2-(Difluoromethoxy)phenyl)(phenyl)methanone (3p-*ortho*) t_R = 57.6 min, 45% (v/v) acetonitrile in water. ^{1}H NMR (700 MHz, $CDCl_3$, 25 ºC): δ 7.81 (m, 2H), 7.60 (m, 1H), 7.54 (m, 1H), 7.46 (m, 3H), 7.34 (m, 1H), 7.30 (m, 1H), 6.45 (t, $^{2}J_{HF}$ = 73.8 Hz, 1H). ^{13}C NMR (175 MHz, $CDCl_3$, 25 ºC): δ 194.72, 148.27, 137.04, 133.53, 132.46, 131.90, 130.01, 129.94, 128.48, 125.58, 121.18, 115.98 (t, $^{1}J_{CF}$ = 262.4 Hz). ^{19}F NMR (376 MHz, $CDCl_3$, 25 ºC): δ –82.8 (d, $^{2}J_{FH}$ = 73.8 Hz, 2F). Spectral data match those previously reported.[5]

(3-(Difluoromethoxy)phenyl)(phenyl)methanone (3p-*meta*) and **(4-(difluoromethoxy)phenyl)(phenyl)methanone (3p-*para*)** t_R = 73.2 min, 50% (v/v) acetonitrile in water. ^{1}H NMR (700 MHz, $CDCl_3$, 25 ºC): δ 7.86 (d, *J* = 8.7 Hz, 2H), 7.79 (m, 3.71H), 7.62 (m, 2.56H), 7.57 (s, 0.82H), 7.51 (m, 4.37H), 7.36 (m, 0.84H), 7.21 (d, *J* = 8.7 Hz, 2H), 6.62 (t, $^{2}J_{HF}$ = 73.1 Hz, 1H), 6.57 (t, $^{2}J_{HF}$ = 73.3 Hz, 0.87H). ^{13}C NMR (175 MHz, $CDCl_3$, 25 ºC): δ 153.72, 137.83, 131.67, 124.33, 118.14, 118.02, 115.57 (t, $^{1}J_{CF}$ = 265.7 Hz), 104.07. ^{19}F NMR (376 MHz, $CDCl_3$, 25 ºC): δ –83.3 (d, $^{2}J_{FH}$ = 73.7 Hz, 2F), –83.9 (d, $^{2}J_{FH}$ = 73.6 Hz, 2F). Spectral data match those previously reported.[5]

1-(Difluoromethoxy)-3-(phenylethynyl)benzene (3t)

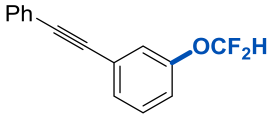

3t

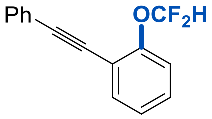

3t'

Prepared according to the **General Procedure A** using 1,2-diphenylethyne (357 mg, 2.00 mmol, 10.0 equiv) as the substrate. After 12 h, the reaction mixture (55% yield, **3t**:**3t'** = 1.1:1 by ^{19}F NMR) was purified by HPLC to provide the title compound(s).

1-(Difluoromethoxy)-3-(phenylethynyl)benzene (3t): t_R = 220 min, 45% (v/v) acetonitrile in water. ^{1}H NMR (700 MHz, $CDCl_3$, 25 ºC): δ 7.53 (m, 4H), 7.35 (m, 3H), 7.10 (m, 2H), 6.53 (t, $^{2}J_{HF}$ = 73.9 Hz, 1H). ^{13}C NMR (175 MHz, $CDCl_3$, 25 ºC): δ 151.03, 133.31, 131.73, 128.56, 128.53, 128.49, 128.40, 123.13, 120.67, 119.54, 115.82 (t, $^{1}J_{CF}$ = 260.6 Hz), 89.78, 88.34. ^{19}F NMR (376 MHz, $CDCl_3$, 25 ºC): δ –83.3 (d, $^{2}J_{FH}$ = 73.9 Hz, 2F). FT-IR (cm^{-1}): 2362 (w, C≡C).

1-(Difluoromethoxy)-2-(phenylethynyl)benzene (3t'): t_R = 244 min, 50% (v/v) acetonitrile in water. ^{1}H NMR (700 MHz, $CDCl_3$, 25 ºC): δ 7.56 (m, 3H), 7.36 (m, 4H), 7.22 (m, 2H), 6.65 (t, $^{2}J_{HF}$ = 74.1 Hz, 1H). ^{13}C NMR (175 MHz, $CDCl_3$, 25 ºC): δ 151.58, 133.65, 131.83, 129.81, 128.84, 128.55, 125.86, 122.93, 120.98, 116.91, 116.47 (t, $^{1}J_{CF}$ = 260.2 Hz), 94.95, 83.98. ^{19}F NMR (376 MHz, $CDCl_3$, 25 ºC): δ –82.7 (d, $^{2}J_{FH}$ = 74.1 Hz, 2F). HRMS (EI) *m/z* calcd for $C_{15}H_{10}OF_2$ [M^+], 244.0700, found, 244.0700.

Methyl 3-(difluoromethoxy)-4-methoxybenzoate (3q)

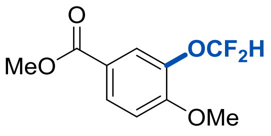

3q

Prepared according to the **General Procedure A** using methyl 4-methoxybenzoate (332 mg, 2.00 mmol, 10.0 equiv) as the substrate. After 12 h, the reaction mixture (61% yield by ^{19}F NMR) was purified by HPLC to provide the title compound(s).

t_R = 157 min, 30% (v/v) acetonitrile in water. ^{1}H NMR (700 MHz, $CDCl_3$, 25 ºC): δ 7.93 (dd, J = 8.6, 1.8 Hz, 1H), 7.83 (d, J = 1.8 Hz, 1H), 7.00 (d, J = 8.6 Hz, 1H), 6.56 (t, $^2J_{FH}$ = 74.6 Hz, 1H), 3.94 (s, 3H), 3.90 (s, 3H). ^{13}C NMR (175 MHz, $CDCl_3$, 25 ºC): δ 165.98, 154.98, 139.43, 128.81, 123.39, 123.02, 115.93 (t, $^1J_{CF}$ = 260.7 Hz), 111.80, 56.20, 52.21. ^{19}F NMR (376 MHz, $CDCl_3$, 25 ºC): δ –83.2 (d, $^2J_{FH}$ = 74.6 Hz, 2F). HRMS (ESI-TOF) m/z calcd for $C_{10}H_{11}F_2O_4$ [(M + H)$^+$], 233.0620, found, 233.0621. FT-IR (cm^{-1}): 1715 (s, C=O).

2-(Difluoromethoxy)-3,5-difluorobenzoic acid (3r)

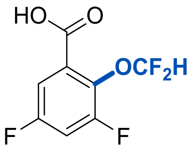

3r

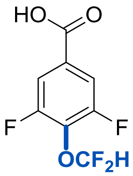

3r'

Prepared according to the **General Procedure A** using 3,5-difluorobenzoic acid (316 mg, 2.00 mmol, 10.0 equiv) as the substrate. After 12 h, the reaction mixture (74% yield, **3r**:**3r'** = 6.4:1 by ^{19}F NMR) was purified by HPLC to provide the title compound(s).

2-(Difluoromethoxy)-3,5-difluorobenzoic acid (3r): t_R = 59.2 min, 30% (v/v) acetonitrile in water. ^{1}H NMR (700 MHz, $CDCl_3$, 25 ºC): δ 7.53 (m, 1H), 7.52 (m, 1H), 7.18 (s, 1H), 6.63 (t, $^2J_{HF}$ = 74.2 Hz, 1H). ^{19}F NMR (376 MHz, $CDCl_3$, 25 ºC): δ -83.00 (dd, J = 74.2, 9.0 Hz, 2F), -109.62 (m, 1F), -120.51 (m, 1F). HRMS (ESI-TOF) m/z calcd for $C_8H_3F_4O_3$ [(M – H)$^-$], 233.0024, found, 223.0024.

4-(Difluoromethoxy)-3,5-difluorobenzoic acid (3r'): t_R = 82.9 min, 30% (v/v) acetonitrile in water. ^{1}H NMR (700 MHz, $CDCl_3$, 25 ºC): δ 7.73 (m, 1H), 6.68 (t, $^2J_{HF}$ = 72.6 Hz, 1H). ^{19}F NMR (376 MHz, $CDCl_3$, 25 ºC): δ -82.35 (dt, J = 72.6, 7.4 Hz, 2F), -123.17 (m, 2F).

3-(Difluoromethoxy)benzoic acid (3s)

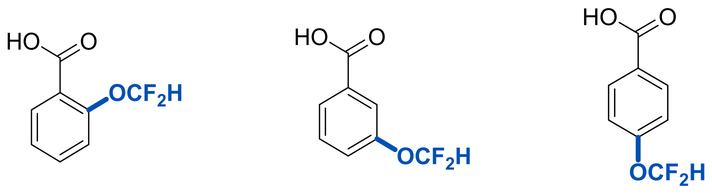

3s-*ortho* **3s**-meta **3s**-para

Prepared according to the **General Procedure A** using benzoic acid (244 mg, 2.00 mmol, 10.0 equiv) as the substrate. After 12 h, the reaction mixture (65% yield, **3s**-*ortho*:**3s**-*meta*:**3s**-*para* = 2.4:2.7:1 by ^{19}F NMR) was purified by HPLC to provide the title compound(s).

2-(Difluoromethoxy)benzoic acid (3s-*ortho*) t_R = 56.7 min, 20% (v/v) acetonitrile in water. ^{1}H NMR (700 MHz, $CDCl_3$, 25 ºC): δ 8.07 (d, *J* = 7.9 Hz, 1H), 7.61 (t, *J* = 7.9 Hz, 1H), 7.35 (t, *J* = 8.5 Hz, 1H), 7.29 (d, *J* = 8.5 Hz, 1H), 6.62 (t, $^2J_{HF}$ = 73.8 Hz, 1H). ^{13}C NMR (175 MHz, $CDCl_3$, 25 ºC): δ 166.35, 150.44, 134.77, 133.02, 126.25, 122.46, 122.25, 116.23 (t, $^1J_{CF}$ = 262.1 Hz). ^{19}F NMR (376 MHz, $CDCl_3$, 25 ºC): δ –83.3 (d, $^2J_{FH}$ = 73.8 Hz, 2F). HRMS (ESI-TOF) *m/z* calcd for $C_8H_5F_2O_3$ [(M – H)$^-$], 187.0212, found, 187.0214.

3-(Difluoromethoxy)benzoic acid (3s-*meta*) and **4-(difluoromethoxy)benzoic acid (3s-*para*)** t_R = 88.7 min, 20% (v/v) acetonitrile in water. ^{1}H NMR (700 MHz, $CDCl_3$, 25 ºC): δ 8.13 (d, *J* = 8.6 Hz, 0.75H), 7.97 (d, *J* = 7.8 Hz, 1H), 7.85 (s, 1H), 7.50 (t, *J* = 7.9 Hz, 1H), 7.39 (d, *J* = 6.5 Hz, 1H), 7.20 (d, *J* = 8.5 Hz, 0.75H), 6.63 (t, *J* = 73.9 Hz, 0.38H), 6.56 (t, *J* = 73.6 Hz, 1H). ^{13}C NMR (175 MHz, $CDCl_3$, 25 ºC): δ 168.81, 168.77, 155.46, 151.19, 132.54, 131.02, 130.22, 127.32, 125.98, 125.29, 121.21, 118.85, 115.44 (t, $^1J_{CF}$ = 261.6 Hz), 114.98 (t, $^1J_{CF}$ = 392.2 Hz). ^{19}F NMR (376 MHz, $CDCl_3$, 25 ºC): δ –83.4 (d, $^2J_{FH}$ = 73.9 Hz, 2F), –84.0 (d, $^2J_{FH}$ = 73.6 Hz, 0.75F).

4,4'-Di-*tert*-butyl-2-(difluoromethoxy)-1,1'-biphenyl (3u)

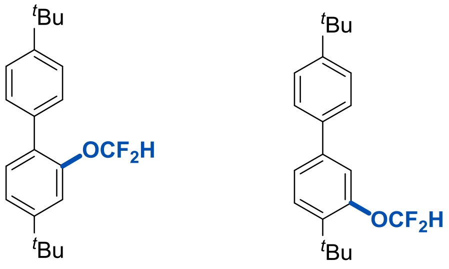

3u **3u'**

Prepared according to the **General Procedure A** using 4,4'-di-*tert*-butyl-1,1'-biphenyl (533 mg, 2.00 mmol, 10.0 equiv) as the substrate with MeCN:DCM 1:1 (1.00 mL, 0.200 M, with respect to **1a**). After 12 h, the reaction mixture (54% yield, **3u**:**3u'** = 2.9:1 by ^{19}F NMR) was purified by HPLC to provide the title compound(s).

4,4'-Di-*tert*-butyl-2-(difluoromethoxy)-1,1'-biphenyl (3u): t_R = 149 min, 60% (v/v) acetonitrile in water. ^{1}H NMR (700 MHz, $CDCl_3$, 25 °C): δ 7.44 (m, 4H), 7.35 (d, *J* = 8.1 Hz, 1H), 7.30 (dd, *J* = 1.8, 8.1 Hz, 1H), 7.22 (d, *J* = 1.8, 1H), 6.30 (t, $^2J_{HF}$ = 74.3 Hz, 1H), 1.36 (s, 9H), 1.36 (s, 9H).^{13}C NMR (175 MHz, $CDCl_3$, 25 °C): δ 152.44, 150.42, 148.20, 134.02, 131.02, 130.98, 129.08, 125.37, 123.10, 117.92, 116.56 (t, $^1J_{CF}$ = 258.8 Hz), 34.85, 34.71, 31.50, 31.38. ^{19}F NMR (376 MHz, $CDCl_3$, 25 °C): δ –82.8 (d, $^2J_{FH}$ = 74.3 Hz, 2F). HRMS (EI) *m/z* calcd for $C_{21}H_{26}OF_2$ [M^+], 332.1952, found, 332.1953.

4,4'-Di-*tert*-butyl-3-(difluoromethoxy)-1,1'-biphenyl (3u'): t_R = 163 min, 60% (v/v) acetonitrile in water. ^{1}H NMR (700 MHz, $CDCl_3$, 25 °C): 7.48 (m, 4H), 7.43 (d, *J* = 8.2 Hz, 1H), 7.34 (dd, *J* = 1.8, 8.2 Hz, 1H), 7.23 (d, *J* = 1.8, 1H), 6.57 (t, $^2J_{HF}$ = 74.8 Hz, 1H), 1.43 (s, 9H), 1.36 (s, 9H). ^{13}C NMR (175 MHz, $CDCl_3$, 25 °C): δ 151.64, 150.88, 140.68, 139.03, 137.05, 128.08, 126.76, 125.97, 123.34, 116.95 (t, $^1J_{CF}$ = 255.6 Hz), 116.62, 34.79, 34.72, 31.48, 30.23. ^{19}F NMR (376 MHz, $CDCl_3$, 25 °C): δ –81.8 (d, $^2J_{FH}$ = 74.8 Hz, 2F).

2-(3-(Difluoromethoxy)phenyl)pyridine (3v)

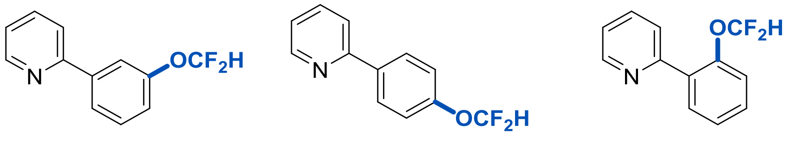

3v **3v'** **3v''**

Prepared according to the **General Procedure A** using 2-phenylpyridine (244 mg, 2.00 mmol, 10.0 equiv) as the substrate with trifluoromethanesulfonic acid (300 mg, 177 μL, 2.00 mmol, 10.0 equiv). After 12 h, the reaction mixture (71% yield, **3v:3v':3v''** = 2.3:1.2:1 by ^{19}F NMR) was quenched with 10% $NaHCO_3$ in water (2 mL), extracted with DCM (3 × 2 mL), and concentrated in vacuo. The residue was purified by HPLC to provide the title compound(s).

2-(3-(Difluoromethoxy)phenyl)pyridine (3v) t_R = 132 min, 50% (v/v) acetonitrile in water. ^{1}H NMR (700 MHz, $CDCl_3$, 25 °C): 8.72 (d, *J* = 4.7 Hz, 1H), 7.79 (dd, *J* = 1.4, 7.7 Hz, 1H), 7.76 (td, *J* = 7.7, 1.4 Hz, 1H), 7.71 (d, *J* = 7.8 Hz, 1H), 7.42 (m, 1H), 7.35 (t, *J* = 7.5 Hz, 1H), 7.28 (d, *J* = 7.3 Hz, 1H), 7.24 (d, *J* = 8.1 Hz, 1H), 6.49 (t, $^2J_{HF}$ = 74.5 Hz, 1H). ^{13}C NMR (175 MHz, $CDCl_3$, 25 °C): δ 155.00, 149.78, 148.78, 136.26, 133.00, 131.77, 130.14, 126.23, 125.02, 122.50, 120.53, 116.76 (t, $^1J_{CF}$ = 259.2 Hz). ^{19}F NMR (376 MHz, $CDCl_3$, 25 °C): δ –83.5 (d, $^2J_{FH}$ = 73.5 Hz, 2F). HRMS (ESI-TOF) *m/z* calcd for $C_{12}H_{10}F_2NO$ [$(M + H)^+$], 222.0725, found, 222.0724.

2-(4-(Difluoromethoxy)phenyl)pyridine (3v') and **2-(2-(difluoromethoxy)phenyl)pyridine (3v'')** t_R = 118 min, 50% (v/v) acetonitrile in water. ^{1}H NMR (700 MHz, $CDCl_3$, 25 °C): δ 8.70 (d, *J* = 4.7 Hz, 0.74H), 8.69 (d, *J* = 4.8 Hz, 1H), 8.01 (d, *J* = 8.7 Hz, 2H), 7.76 (m, 5.13H), 7.47 (t, *J* = 7.8 Hz, 0.77H), 7.24 (m, 2H), 6.60 (t, $^2J_{HF}$ = 73.3 Hz, 0.80H), 6.57 (t, $^2J_{HF}$ = 73.5 Hz, 1H). ^{13}C NMR (175 MHz, $CDCl_3$, 25 °C): δ 156.43, 156.22, 152.05, 151.96, 149.94, 149.89, 141.59, 137.06, 137.01, 136.78, 130.27, 128.58, 123.92, 122.83, 122.37, 120.78, 120.45, 120.11, 119.68, 118.10, 116.18 (t, $^1J_{CF}$ = 259.5 Hz), 115.99 (t, $^1J_{CF}$ = 259.5 Hz). ^{19}F NMR (376 MHz, $CDCl_3$, 25 °C): δ –83.5 (d, $^2J_{FH}$ = 73.3 Hz, 1.56F), –84.3 (d, $^2J_{FH}$ = 73.5 Hz, 2F).

1-(Difluoromethoxy)naphthalene (3w)

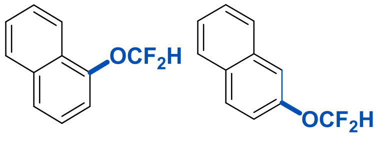

3w **3w'**

Prepared according to the **General Procedure A** using naphthalene (256 mg, 2.00 mmol, 10.0 equiv) as the substrate. After 12 h, the reaction mixture (72% yield, **3w**:**3w'** = 2.4:1 by ^{19}F NMR) was purified by HPLC to provide the title compound(s).

1-(Difluoromethoxy)naphthalene (3w) and **2-(difluoromethoxy)naphthalene (3w')** t_R = 23.8 min, 50% (v/v) acetonitrile in water. ^{1}H NMR (700 MHz, $CDCl_3$, 25 °C): δ 8.19 (m, 1H), 7.86 (m, 1.75H), 7.80 (m, 0.37H), 7.71 (m, 1.92H), 7.56 (m, 0.70H), 7.52 (m, 0.36H), 7.42 (t, J = 7.9 Hz, 1H), 7.29 (m, 0.34H), 7.20 (m, 1H), 6.67 (t, $^2J_{HF}$ = 74.1 Hz, 1H), 6.63 (t, $^2J_{HF}$ = 73.9 Hz, 0.40H). ^{13}C NMR (175 MHz, $CDCl_3$, 25 °C): δ 149.09, 147.57, 134.83, 133.91, 131.17, 130.22, 127.91, 127.89, 127.63, 127.10, 127.09, 127.09, 126.75, 126.58, 125.83, 125.52, 125.47, 121.75, 119.83, 116.70 (t, $^1J_{CF}$ = 259.1 Hz), 116.20 (t, J_{CF} = 259.1 Hz), 115.48, 113.83. ^{19}F NMR (376 MHz, $CDCl_3$, 25 °C): δ –80.0 (d, $^2J_{FH}$ = 74.1 Hz, 2F), –80.7 (d, $^2J_{FH}$ = 74.0 Hz, 0.80F). HRMS (EI) *m/z* calcd for $C_{11}H_8OF_2$ [M^+], 194.0543, found, 194.0542. Spectral data match those previously reported.[6]

***N*-(2-(Difluoromethoxy)-4-(trifluoromethoxy)phenyl)-2,2,2-trifluoroacetamide (3x)**

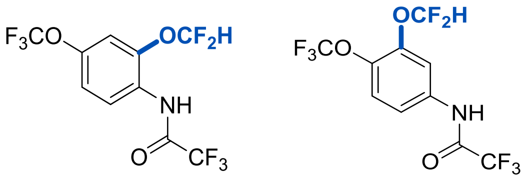

3x **3x'**

Prepared according to the **General Procedure A** using 2,2,2-trifluoro-*N*-(4-(trifluoromethoxy)phenyl)acetamide (546 mg, 2.00 mmol, 10.0 equiv) as the substrate. After 12 h, the reaction mixture (66% yield, **3x**:**3x'** = 7.3:1 by ^{19}F NMR) was purified by HPLC to provide the title compound(s).

***N*-(2-(Difluoromethoxy)-4-(trifluoromethoxy)phenyl)-2,2,2-trifluoroacetamide (3x):** t_R = 151 min, 40% (v/v) acetonitrile in water. ^{1}H NMR (700 MHz, $CDCl_3$, 25 °C): 8.41 (d, J = 9.0 Hz, 1H), 8.26 (br. s, 1H), 7.19 (d, J = 9.0 Hz, 1H), 7.13 (s, 1H), 6.63 (t, $^2J_{HF}$ = 72.7 Hz, 1H). ^{13}C NMR (175 MHz, $CDCl_3$, 25 °C): δ 154.74 (q, $^2J_{CF}$ = 38.1 Hz), 146.15, 140.55, 126.12, 122.49, 120.28 (q, $^1J_{CF}$ = 258.6 Hz), 118.76, 115.52 (t, $^1J_{CF}$ = 265.6 Hz), 115.41 (q, $^1J_{CF}$ = 288.4 Hz), 112.74. ^{19}F NMR (376 MHz, $CDCl_3$, 25 °C): δ –63.7 (s, 3F), –81.3 (s, 3F), –86.9. (d, $^2J_{FH}$ = 72.7 Hz, 2F). HRMS (ESI-TOF) *m/z* calcd for $C_{10}H_6F_8NO_3$ [$(M + H)^+$], 340.0214, found, 340.0217. FT-IR (cm^{-1}): 1725 (s, C=O).

***N*-(3-(Difluoromethoxy)-4-(trifluoromethoxy)phenyl)-2,2,2-trifluoroacetamide (3x'):** t_R = 168 min, 40% (v/v) acetonitrile in water. ^{1}H NMR (700 MHz, $CDCl_3$, 25 °C): 7.89 (br. s, 1H), 7.67 (d, J = 2.4 Hz,

1H), 7.46 (dd, J = 2.4, 8.9 Hz, 1H), 7.38 (d, J = 8.9 Hz, 1H), 6.56 (t, $^2J_{HF}$ = 72.9 Hz, 1H). ^{13}C NMR (175 MHz, $CDCl_3$, 25 ºC): δ 154.88 (q, $^2J_{CF}$ = 38.2 Hz), 143.33, 138.04, 134.43, 123.91, 120.35 (q, $^1J_{CF}$ = 259.1 Hz), 117.92, 115.43 (q, $^1J_{CF}$ = 288.1 Hz), 115.37 (t, $^1J_{CF}$ = 265.0 Hz), 114.40. ^{19}F NMR (376 MHz, $CDCl_3$, 25 ºC): δ –63.8 (s, 3F), –81.1 (s, 3F), –87.2. (d, $^2J_{FH}$ = 72.9 Hz, 2F).

1-(Difluoromethoxy)-3-(phenylsulfonyl)benzene (3y)

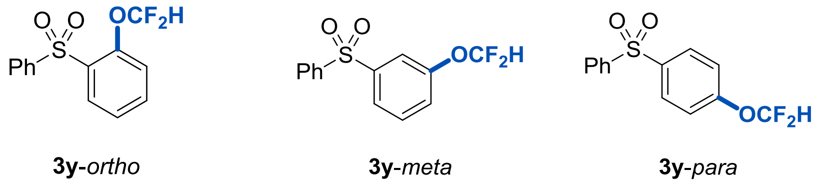

Prepared according to the **General Procedure A** using sulfonyldibenzene (437 mg, 2.00 mmol, 10.0 equiv) as the substrate. After 12 h, the reaction mixture (77% yield, **3y**-*ortho*:**3y**-*meta*:**3y**-*para* = 1.1:3.8:1 by ^{19}F NMR) was purified by HPLC to provide the title compound(s).

1-(Difluoromethoxy)-2-(phenylsulfonyl)benzene (3y-*ortho*) t_R = 70.8 min, 40% (v/v) acetonitrile in water. ^{1}H NMR (700 MHz, $CDCl_3$, 25 ºC): δ 8.25 (m, 1H), 7.98 (d, J = 7.4 Hz, 2H), 7.61 (t, J= 7.4 Hz, 2H), 7.52 (t, J = 7.8 Hz, 2H), 7.43 (t, J = 7.8 Hz, 1H), 7.21 (d, J = 7.5 Hz, 1H), 6.50 (t, $^2J_{HF}$ = 74.2 Hz, 1H). ^{13}C NMR (175 MHz, $CDCl_3$, 25 ºC): δ 148.58, 140.73, 135.56, 133.73, 133.59, 130.31, 129.07, 128.61, 126.22, 121.98, 116.18 (t, $^1J_{CF}$ = 262.3 Hz). ^{19}F NMR (376 MHz, $CDCl_3$, 25 ºC): δ –83.6 (d, $^2J_{FH}$ = 74.2 Hz, 2F). FT-IR (cm^{-1}): 1388 (s, S–O *asymmetric*), 1160 (s, S–O *symmetric*).

1-(Difluoromethoxy)-3-(phenylsulfonyl)benzene (3y-*meta*) and **1-(difluoromethoxy)-4-(phenylsulfonyl)benzene (3y-*para*))** t_R = 90.6 min, 40% (v/v) acetonitrile in water. ^{1}H NMR (700 MHz, $CDCl_3$, 25 ºC): δ 7.96 (m, 3.44H), 7.79 (m, 1H), 7.70 (m, 1H), 7.59 (m, 1.31H), 7.52 (m, 3.79H), 7.32 (m, 1H), 7.22 (m, 0.67H), 6.57 (t, $^2J_{HF}$ = 73.2 Hz, 0.38H), 6.55 (t, $^2J_{HF}$ = 73.4 Hz, 1H). ^{13}C NMR (175 MHz, $CDCl_3$, 25 ºC): δ 154.66, 151.34, 143.67, 141.53, 141.06, 138.42, 133.71, 133.50, 131.04, 130.07, 129.60, 129.54, 127.94, 127.76, 124.66, 124.53, 119.74, 118.93, 116.20 (t, $^1J_{CF}$ = 394.8 Hz), 115.98 (t, $^1J_{CF}$ = 394.2 Hz). ^{19}F NMR (376 MHz, $CDCl_3$, 25 ºC): δ –83.9 (d, $^2J_{FH}$ = 73.4 Hz, 2F), –84.5 (d, $^2J_{FH}$ = 73.2 Hz, 2F). HRMS (EI) m/z calcd for $C_{13}H_{10}O_3F_2S$ [M^+], 284.0319, found, 284.0318.

3-(Difluoromethoxy)phenyl phenyl carbonate (3z)

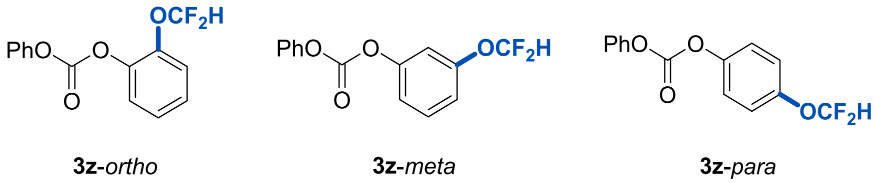

Prepared according to the **General Procedure A** using diphenyl carbonate (428 mg, 2.00 mmol, 10.0 equiv) as the substrate. After 12 h, the reaction mixture (74% yield, **3z**-*ortho*:**3z**-*meta*:**3z**-*para* = 1.4:1.5:1 by ^{19}F NMR) was purified by HPLC to provide the title compound(s).

2-(Difluoromethoxy)phenyl phenyl carbonate (3z-*ortho*)) t_R = 88.9 min, 45% (v/v) acetonitrile in water. ^{1}H NMR (700 MHz, $CDCl_3$, 25 ºC): δ 7.42 (m, 2H), 7.34 (m, 1H), 7.28 (m, 6H), 6.49 (t, $^2J_{HF}$ = 73.4 Hz, 1H). ^{13}C NMR (175 MHz, $CDCl_3$, 25 ºC): δ 151.74, 151.13, 143.15, 142.44, 129.79, 127.74, 126.70, 126.64, 123.32, 121.63, 120.99, 116.41 (t, $^1J_{CF}$ = 261.3 Hz). ^{19}F NMR (376 MHz, $CDCl_3$, 25 ºC): δ –82.3 (d, $^2J_{FH}$ = 73.4 Hz, 2F). FT-IR (cm^{-1}): 1774 (s, C=O).

3-(Difluoromethoxy)phenyl phenyl carbonate (3z-*meta*) and **4-(difluoromethoxy)phenyl phenyl carbonate (3z-*para*))** t_R = 100 min, 4% (v/v) acetonitrile in water. ^{1}H NMR (700 MHz, $CDCl_3$, 25 ºC): δ 7.43 (m, 4.54H), 7.28 (m, 7.38H), 7.18 (m, 2.76H), 7.11 (m, 0.60H), 7.06 (m, 0.62H), 6.53 (t, $^2J_{HF}$ = 73.8 Hz, 0.55H), 6.50 (t, $^2J_{HF}$ = 74.0 Hz, 1H). ^{13}C NMR (175 MHz, $CDCl_3$, 25 ºC): δ 152.13, 151.83, 151.78, 151.72, 151.02, 150.98, 148.90, 148.25, 130.54, 129.77, 129.71, 126.62, 126.58, 126.45, 122.42, 121.03, 120.98, 118.10, 117.41, 115.90 (t, $^1J_{CF}$ = 260.9 Hz), 115.80 (t, $^1J_{CF}$ = 260.8 Hz), 113.04. ^{19}F NMR (376 MHz, $CDCl_3$, 25 ºC): δ –83.1 (d, $^2J_{FH}$ = 74.0 Hz, 2F), –83.5 (d, $^2J_{FH}$ = 73.8 Hz, 1.55F). HRMS (EI) *m/z* calcd for $C_{14}H_{10}O_4F_2$ [M$^+$], 280.0547, found, 280.0549.

2,6-Di-*tert*-butyl-3-(difluoromethoxy)pyridine (3aa)

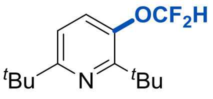

3aa

Prepared according to the **General Procedure A** using 2,6-di-*tert*-butylpyridine (383 mg, 2.00 mmol, 10.0 equiv) as the substrate. After 12 h, the reaction mixture (52% yield by ^{19}F NMR) was purified by HPLC to provide the title compound(s).

t_R = 154 min, 55% (v/v) acetonitrile in water. ^{1}H NMR (700 MHz, $CDCl_3$, 25 ºC): 7.26 (d, J = 8.4 Hz, 1H), 7.14 (d, J = 8.4 Hz, 1H), 6.48 (t, $^2J_{HF}$ = 73.9 Hz, 1H), 1.41 (s, 9H), 1.33 (s, 9H). ^{13}C NMR (175 MHz, $CDCl_3$, 25 ºC): δ 163.69, 156.98, 144.57, 125.74, 116.87, 116.61 (t, $^1J_{CF}$ = 258.3 Hz), 38.39, 37.55, 30.25, 29.17. ^{19}F NMR (376 MHz, $CDCl_3$, 25 ºC): δ –81.3 (d, $^2J_{FH}$ = 73.9 Hz, 2F). HRMS (ESI-TOF) *m/z* calcd for $C_{14}H_{22}F_2NO$ [(M + H)$^+$], 258.1664, found, 258.1661.

3,4-Dibromo-2-(difluoromethoxy)thiophene (3ab)

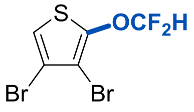

3ab

Prepared according to the **General Procedure A** using 3,4-dibromothiophene (484 mg, 2.00 mmol, 10.0 equiv) as the substrate. After 12 h, the reaction mixture (70% yield by ^{19}F NMR) was purified by HPLC to provide the title compound(s).

t_R = 111 min, 50% (v/v) acetonitrile in water. ^{1}H NMR (700 MHz, $CDCl_3$, 25 ºC): 7.08 (s, 1H), 6.48 (t, $^2J_{HF}$ = 72.1 Hz, 1H). ^{13}C NMR (175 MHz, $CDCl_3$, 25 ºC): δ 146.51, 116.31, 115.55 (t, $^1J_{CF}$ = 269.2 Hz), 111.43,

105.59. ^{19}F NMR (376 MHz, CDCl$_3$, 25 °C): δ –84.7 (d, $^2J_{FH}$ = 72.1 Hz, 2F). HRMS (EI) *m/z* calcd for $C_5H_2OSBr_2F_2$ [M$^+$], 305.8161, found, 305.8161.

4-Bromo-5-(difluoromethoxy)thiophene-2-carbonitrile (3ac)

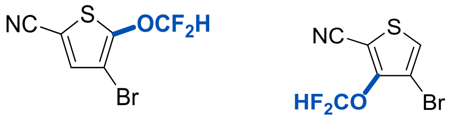

3ac **3ac'**

Prepared according to the **General Procedure A** using 4-bromothiophene-2-carbonitrile (376 mg, 2.00 mmol, 10.0 equiv) as the substrate. After 12 h, the reaction mixture (67% yield, **3ac**:**3ac'** = 3.8:1 by ^{19}F NMR) was purified by HPLC to provide the title compound(s). 342 mg, 91% 4-bromothiophene-2-carbonitrile was recovered (**t*R*** = 33 min, 35% (v/v) acetonitrile in water).

4-Bromo-5-(difluoromethoxy)thiophene-2-carbonitrile (3ac): 24.5 mg, 48% yield, t_R = 118 min, 35% (v/v) acetonitrile in water. ^{1}H NMR (700 MHz, CDCl$_3$, 25 °C): 7.41 (s, 1H), 6.58 (t, $^2J_{HF}$ = 71.4 Hz, 1H). ^{13}C NMR (175 MHz, CDCl$_3$, 25 °C): δ 150.25, 137.61, 114.79 (t, $^1J_{CF}$ = 271.8 Hz), 112.53, 104.12, 102.20. ^{19}F NMR (376 MHz, CDCl$_3$, 25 °C): δ –85.4 (d, $^2J_{FH}$ = 71.4 Hz, 2F).

4-Bromo-3-(difluoromethoxy)thiophene-2-carbonitrile (3ac'): 6.8 mg, 13% yield. t_R = 139 min, 35% (v/v) acetonitrile in water. ^{1}H NMR (700 MHz, CDCl$_3$, 25 °C): 7.54 (s, 1H), 6.71 (t, $^2J_{HF}$ = 71.8 Hz, 1H). ^{13}C NMR (175 MHz, CDCl$_3$, 25 °C): δ 150.76, 128.82, 115.15 (t, $^1J_{CF}$ = 269.7 Hz), 110.67, 107.60, 100.70. ^{19}F NMR (376 MHz, CDCl$_3$, 25 °C): δ –82.2 (d, $^2J_{FH}$ = 71.8 Hz, 2F). HRMS (EI) *m/z* calcd for $C_6H_2NOSF_2Br$ [M$^+$], 252.9009, found, 252.9008.

4-Bromo-5-(difluoromethoxy)thiophene-2-carboxylic acid (3ad)

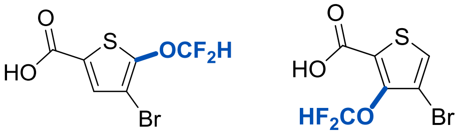

3ad **3ad'**

Prepared according to the **General Procedure A** using 4-bromothiophene-2-carboxylic acid (414 mg, 2.00 mmol, 10.0 equiv) as the substrate. After 12 h, the reaction mixture (59% yield, **3ad**:**3ad'** = 3.5:1 by ^{19}F NMR) was concentrated and added 1.00 M NaOH (aq) (5 mL), dichloromethane (10.0 mL), and extracted with dichloromethane (3 × 10 mL). Then to the aqueous layer was added 1.00 M HCl (aq) (20 mL), extracted with diethyl ether (5 × 20 mL), dried with $MgSO_4$, and concentrated. Then the mixture was purified by HPLC to provide the title compound(s). 342 mg, 83% of 4-bromothiophene-2-carboxylic acid was recovered. (**t*R* =** 33.2 min, 30% (v/v) acetonitrile in water with 0.100% TFA).

4-Bromo-5-(difluoromethoxy)thiophene-2-carboxylic acid (3ad): 23.1 mg, 42% yield, t_R = 85.8 min, 30% (v/v) acetonitrile in water. ^{1}H NMR (700 MHz, CDCl$_3$, 25 °C): 7.68 (s, 1H), 6.58 (t, $^2J_{HF}$ = 71.5 Hz, 1H).^{13}C NMR (175 MHz, CDCl$_3$, 25 °C): δ 166.06, 152.43, 135.43, 125.14, 115.17 (t, $^1J_{CF}$ = 270.1 Hz),

102.35. ^{19}F NMR (376 MHz, $CDCl_3$, 25 ºC): δ –85.2 (d, $^{2}J_{FH}$ = 71.5 Hz, 2F). HRMS (ESI-TOF) *m/z* calcd for $C_6H_2BrF_2O_3S$ [(M – H)$^-$], 270.8882, found, 270.8893.

4-Bromo-3-(difluoromethoxy)thiophene-2-carboxylic acid (3ad'): 6.2 mg, 11% yield, t_R = 73.9 min, 30% (v/v) acetonitrile in water. ^{1}H NMR (700 MHz, $CDCl_3$, 25 ºC): 7.58 (s, 1H), 6.80 (t, $^{2}J_{HF}$ = 74.6 Hz, 1H). ^{13}C NMR (175 MHz, $CDCl_3$, 25 ºC): δ 163.23, 149.07, 129.39, 119.77, 116.46 (t, $^{1}J_{CF}$ = 265.3 Hz), 109.33. ^{19}F NMR (376 MHz, $CDCl_3$, 25 ºC): δ –83.2 (d, $^{2}J_{FH}$ = 74.6 Hz, 2F).

4-Amino-3-(4-chloro-2-(difluoromethoxy)phenyl)butanoic acid di-trifluoroacetic acid (5a)

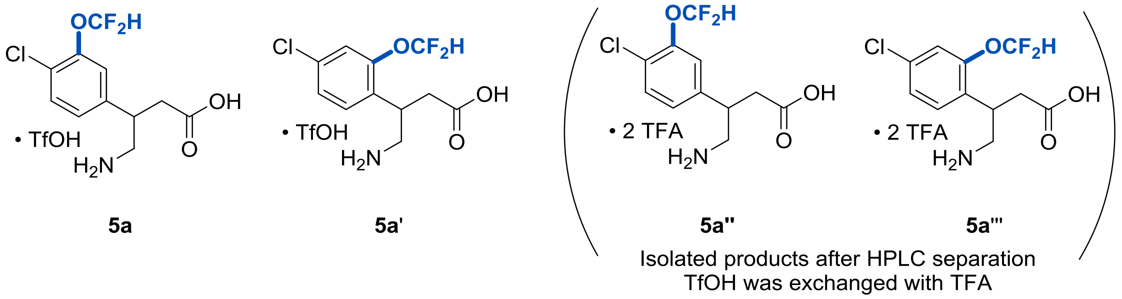

5a **5a'** **5a''** **5a'''**

Isolated products after HPLC separation
TfOH was exchanged with TFA

Prepared according to the **General Procedure B** using (±)-Baclofen® (42.7 mg, 0.200 mmol, 1.00 equiv) as the substrate with trifluoromethanesulfonic acid (30.0 mg, 17.8 μL, 0.200 mmol, 1.0 equiv). After 12 h, the reaction mixture (51.7 mg, 51% yield, **5a**:**5a'** = 5.7:1) was purified by HPLC to provide the title compound(s). Substrate (±)-Baclofen® was also recovered (**t_R** = 22.2 min, 28.2 mg, 32% yield) as the di-trifluoroacetic acid salt.

4-Amino-3-(4-chloro-3-(difluoromethoxy)phenyl)butanoic acid di-trifluoroacetic acid (5a'') and **4-amino-3-(4-chloro-2-(difluoromethoxy)phenyl)butanoic acid di-trifluoroacetic acid (5a'''):** t_R = 62.0 min, 15% (v/v) acetonitrile in water with 0.100% TFA. ^{1}H NMR (700 MHz, CD_3CN, 25 ºC): δ 10.82 (br. s, 2.1H), 7.52 (d, *J* = 8.3 Hz, 1H), 7.37 (d, *J* = 8.4 Hz, 0.39H), 7.29 (dd, *J* = 1.9, 8.4 Hz, 0.32H), 7.26 (m, 0.85H), 7.25 (m, 0.31H), 7.21 (m, 1H), 7.21 (br. s, 2.74), 6.86 (t, $^{2}J_{HF}$ = 73.2 Hz, 0.45H), 6.86 (t, $^{2}J_{HF}$ = 73.4 Hz, 1H), 3.79 (m, 0.43H), 3.49 (m, 1H), 3.37 (m, 1.43H), 3.25 (m, 1.44H), 2.84 (m, 3H). ^{13}C NMR (175 MHz, CD_3CN, 25 ºC): δ 174.16, 173.82, 151.27, 148.41, 141.57, 134.71, 132.15, 131.80, 130.15, 127.42, 126.72, 125.52, 121.62, 119.55, 117.55 (t, $^{1}J_{CF}$ = 259.0 Hz), 117.43 (t, $^{1}J_{CF}$ = 258.6 Hz), 45.08, 44.28, 40.09, 38.67, 37.74, 34.63. ^{19}F NMR (376 MHz, $CDCl_3$, 25 ºC): δ –82.6 (d, $^{2}J_{FH}$ = 73.4 Hz, 0.35F), –82.8 (d, $^{2}J_{FH}$ = 73.4 Hz, 2F). HRMS (ESI) *m/z* calcd for $C_{11}H_{13}NO_3F_2Cl$ [(M+H)$^+$], 280.0552, found, 280.0554.

2-(3-cyano-5-(Difluoromethoxy)-4-isobutoxyphenyl)-4-methylthiazole-5-carboxylic acid trifluoroacetic acid (5b)

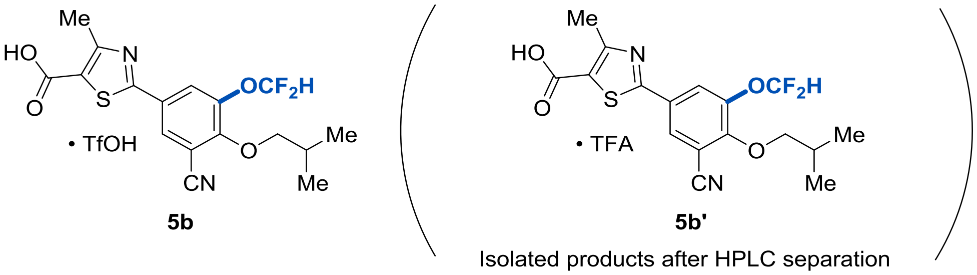

Isolated products after HPLC separation
TfOH was exchanged with TFA

Prepared according to the **General Procedure B** using Febuxostat® (63.3 mg, 0.200 mmol, 1.00 equiv) as the substrate with trifluoromethanesulfonic acid (30.0 mg, 17.8 μL, 0.200 mmol, 1.0 equiv). After 12 h, the reaction mixture (14.6 mg, 15% yield) was purified by HPLC to provide the title compound(s). Substrate Febuxostat® was also recovered (**t*R*** = 52.6 min, 54.4 mg, 63% yield) as the trifluoroacetic acid salt.

2-(3-cyano-5-(difluoromethoxy)-4-isobutoxyphenyl)-4-methylthiazole-5-carboxylic acid trifluoroacetic acid (5b') t_R = 99.6 min, 45% (v/v) acetonitrile in water with 0.100% TFA. ^{1}H NMR (700 MHz, $CDCl_3$, 25 ºC): δ 8.03 (d, *J* = 2.2 Hz, 1H), 8.00 (d, *J* = 2.2 Hz, 1H), 6.62 (t, $^2J_{HF}$ = 73.0 Hz, 1H), 5.62 (br. s, 2H), 4.12 (d, *J* = 6.3 Hz, 2H), 2.80 (s, 3H), 2.16 (m, 1H), 1.09 (s, 3H), 1.08 (s, 3H). ^{13}C NMR (175 MHz, $CDCl_3$, 25 ºC): δ 167.34, 166.60, 163.28, 155.69, 143.96, 129.12, 128.75, 124.91, 122.28, 115.83 (t, $^1J_{CF}$ = 263.6 Hz), 115.00, 108.52, 82.04, 29.33, 19.00, 17.71. ^{19}F NMR (376 MHz, $CDCl_3$, 25 ºC): δ –81.3 (s, 3F). –86.8 (d, $^2J_{FH}$ = 73.0 Hz, 2F). HRMS (ESI) *m/z* calcd for $C_{17}H_{17}N_2O_4F_2S$ [(M + H)$^+$], 383.0877, found, 383.0876.

1-(3-(Difluoromethoxy)-2,6-dimethylphenoxy)propan-2-amine (5c)

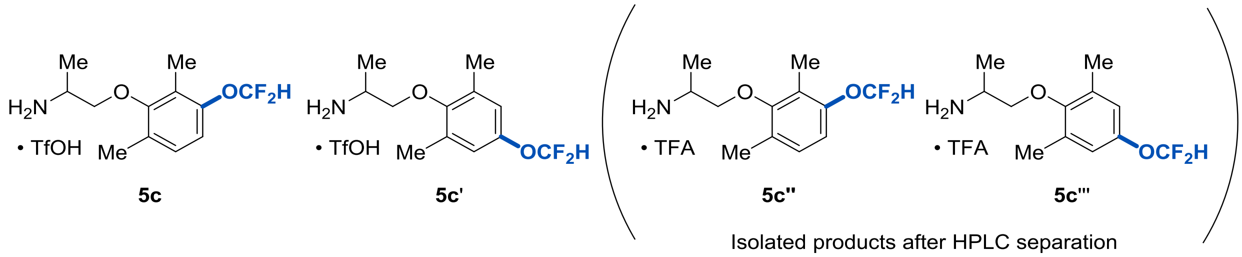

Isolated products after HPLC separation
TfOH was exchanged with TFA

Prepared according to the **General Procedure B** using Mexlietine® HCl (43.1 mg, 0.200 mmol, 1.00 equiv) as the substrate with trifluoromethanesulfonic acid (30.0 mg, 17.8 μL, 0.200 mmol, 1.0 equiv). After 12 h, the reaction mixture (22.3 mg, 33% yield) was purified by HPLC to provide the title compound(s). Substrate Mexlietine® HCl was also recovered (**t*R*** = 37.6 min, 33.9 mg, 58% yield) as the trifluoroacetic acid salt.

1-(3-(Difluoromethoxy)-2,6-dimethylphenoxy)propan-2-amine (5c) and **1-(4-(difluoromethoxy)-2,6-dimethylphenoxy)propan-2-amine (5c')** t_R = 116 min, 20% (v/v) acetonitrile in water with 0.100% TFA. ^{1}H NMR (700 MHz, CD_3CN, 25 ºC): δ 7.63 (br. s, 3.37H), 7.09 (d, *J* = 8.2 Hz, 1H), 6.90 (d, *J* = 8.2 Hz, 1H), 6.86 (s, 0.33H), 6.71 (t, $^2J_{HF}$ = 74.6 Hz, 1.28H), 3.90 (m, 2H), 3.81 (m, 1.13H), 2.28 (s, 4.34H), 2.21

(s, 2.86H), 1.97 (m, 1.21H), 1.43 (m, 4.05H). ^{13}C NMR (175 MHz, $CDCl_3$, 25 °C): δ 156.09, 152.67, 149.59, 148.29, 133.74, 130.24, 129.62, 129.33, 124.38, 120.37, 117.91 (t, $^{1}J_{CF}$ = 256.9 Hz), 117.55 (t, $^{1}J_{CF}$ = 256.7 Hz), 116.38, 72.71, 72.54, 49.40, 16.47, 16.24, 16.10, 15.12, 13.67, 9.86. ^{19}F NMR (376 MHz, $CDCl_3$, 25 °C): δ –76.4 (s, 3F). –82.0 (d, $^{2}J_{FH}$ = 74.6 Hz, 2F), –82.7 (d, $^{2}J_{FH}$ = 74.6 Hz, 2F). HRMS (ESI) *m/z* calcd for $C_{12}H_{18}NO_2F_2$ [(M + H)$^+$], 246.1306, found, 246.1311.

(S)-6-Chloro-4-(cyclopropylethynyl)-8-(difluoromethoxy)-4-(trifluoromethyl)-1,4-dihydro-2*H*-benzo[*d*][1,3]oxazin-2-one (5d)

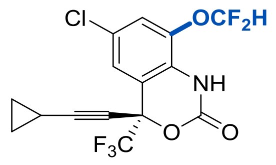

5d

Prepared according to the **General Procedure B** using Efavirenz® (63.3 mg, 0.200 mmol, 1.00 equiv) as the substrate with potassium carbonate (27.6 mg, 0.200 mmol, 1.0 equiv). After 12 h, the reaction mixture (15.9 mg, 21% yield) was purified by HPLC to provide the title compound(s). Substrate Efavirenz® was also recovered (**t_R** = 96.4 min, 31.7 mg, 50% yield).

(S)-6-Chloro-4-(cyclopropylethynyl)-8-(difluoromethoxy)-4-(trifluoromethyl)-1,4-dihydro-2*H*-benzo[*d*][1,3]oxazin-2-one (5d) t_R = 146 min, 40% (v/v) acetonitrile in water. ^{1}H NMR (700 MHz, $CDCl_3$, 25 °C): δ 8.17 (s, 1H), 7.39 (d, *J* = 1.5 Hz, 1H), 7.30 (d, *J* = 1.5 Hz, 1H), 6.62 (t, $^{2}J_{HF}$ = 71.4 Hz, 1H), 1.40 (m, 1H), 0.93 (m, 2H), 0.86 (m, 2H). ^{13}C NMR (175 MHz, $CDCl_3$, 25 °C): δ 147.03, 136.32, 128.56, 126.69, 124.87, 122.64, 122.13 (q, $^{1}J_{CF}$ = 287.5 Hz), 116.57, 116.00 (t, $^{1}J_{CF}$ = 269.57 Hz), 96.40, 79.02 (q, $^{2}J_{CF}$ = 35.1 Hz), 65.92, 9.00, 8.98, -0.47. ^{19}F NMR (376 MHz, $CDCl_3$, 25 °C): δ –80.8 (s, 3F). –82.2 (d, $^{2}J_{FH}$ = 71.4 Hz, 1F), –82.4 (d, $^{2}J_{FH}$ = 71.4 Hz, 1F). HRMS (ESI) *m/z* calcd for $C_{15}H_{10}NO_3F_5Cl$ [(M + H)$^+$], 382.0269, found, 382.0265. FT-IR (cm^{-1}): 1738 (s, C=O).

2-(2-Methyl-5-nitro-1*H*-imidazol-1-yl)ethyl 3-(difluoromethoxy)-4-methylbenzoate (5e)

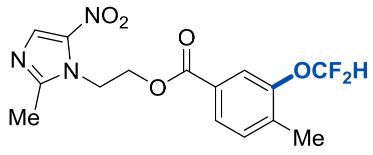

5e

Prepared according to the **General Procedure B** using 2-(2-methyl-5-nitro-1*H*-imidazol-1-yl)ethyl 4-methylbenzoate[7] (57.9 mg, 0.200 mmol, 1.00 equiv) as the substrate with potassium carbonate (27.6 mg, 0.200 mmol, 1.0 equiv). After 12 h, the reaction mixture (18.2 mg, 26% yield) was purified by HPLC to provide the title compound(s). Substrate 2-(2-methyl-5-nitro-1*H*-imidazol-1-yl)ethyl 4-methylbenzoate was also recovered (**t_R** = 61.9 min, 30.5 mg, 53% yield).

2-(2-Methyl-5-nitro-1*H*-imidazol-1-yl)ethyl 3-(difluoromethoxy)-4-methylbenzoate (5e) t_R = 117 min, 35% (v/v) acetonitrile in water. ^{1}H NMR (700 MHz, $CDCl_3$, 25 ºC): δ 7.97 (s, 1H), 7.67 (m, 1H), 7.61 (s, 1H), 7.30 (d, *J* = 7.9 Hz, 1H), 6.57 (t, $^2J_{HF}$ = 73.4 Hz, 1H), 4.72 (t, *J* = 5.1 Hz, 2H), 4.66 (t, J = 5.1 Hz, 2H), 2.49 (s, 3H), 2.34 (s, 3H). ^{13}C NMR (175 MHz, $CDCl_3$, 25 ºC): δ 165.23, 150.92, 149.60, 138.72, 136.66, 133.43, 131.88, 128.23, 126.59, 119.71, 115.95 (t, $^1J_{CF}$ = 260.9 Hz), 63.15, 45.27, 16.71, 14.43. ^{19}F NMR (376 MHz, $CDCl_3$, 25 ºC): δ –82.6 (d, $^2J_{FH}$ = 73.4 Hz, 2F). HRMS (ESI) *m/z* calcd for $C_{15}H_{16}N_3O_5F_2$ [(M + H)$^+$], 356.1058, found, 356.1049. FT-IR (cm^{-1}): 1723 (s, C=O).

(1*R*,2*S*,5*R*)-2-Isopropyl-5-methylcyclohexyl 4-bromo-5-(difluoromethoxy)thiophene-2-carboxylate (5f)

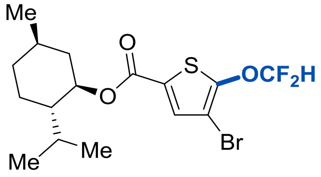

5f

Prepared according to the **General Procedure B** using (1*R*,2*S*,5*R*)-2-isopropyl-5-methylcyclohexyl 4-bromothiophene-2-carboxylate[8] (69.1 mg, 0.200 mmol, 1.00 equiv) as the substrate with potassium carbonate (27.6 mg, 0.200 mmol, 1.0 equiv). After 12 h, the reaction mixture (16.4 mg, 20% yield) was purified by HPLC to provide the title compound(s). Substrate (1*R*,2*S*,5*R*)-2-isopropyl-5-methylcyclohexyl 4-bromothiophene-2-carboxylate was also recovered (**t_R** = 63.7 min, 36.9 mg, 53% yield).

(1*R*,2*S*,5*R*)-2-Isopropyl-5-methylcyclohexyl 4-bromo-5-(difluoromethoxy)thiophene-2-carboxylate (5f) t_R = 80.0 min, 65% (v/v) acetonitrile in water. ^{1}H NMR (700 MHz, $CDCl_3$, 25 ºC): δ 7.55 (s, 1H), 6.54 (t, $^2J_{HF}$ = 71.9 Hz, 1H), 4.86 (m, 1H), 2.07 (m, 1H), 1.89 (m, 1H), 1.71 (m, 2H), 1.51 (m, 1H), 1.09 (m, 2H), 0.92 (d, *J* = 7.4 Hz, 3H), 0.91 (, *J* = 7.4 Hz, 3H), 0.78 (d, *J* = 7.0 Hz, 3H). ^{13}C NMR (175 MHz, $CDCl_3$, 25 ºC): δ 160.56, 150.87, 133.23, 127.07, 115.32 (t, $^1J_{CF}$ = 269.6 Hz), 101.91, 76.25, 47.23, 40.95, 34.28, 31.56, 26.65, 23.73, 22.12, 20.83, 16.64. ^{19}F NMR (376 MHz, $CDCl_3$, 25 ºC): δ –85.2 (d, $^2J_{FH}$ = 72.1 Hz, 2F). HRMS (ESI) *m/z* calcd for $C_{10}H_{11}OF_2Br$ [(M + H)$^+$], 280.0552, found, 280.0554. FT-IR (cm^{-1}): 1719 (s, C=O).

Physical Properties Studies

Differential Scanning Calorimetry (DSC)

Differential Scanning Calorimeter (DSC) was performed to determine the temperature and heat flow associated with our material as a function of time and temperature (reagent **1a** is non-explosive).

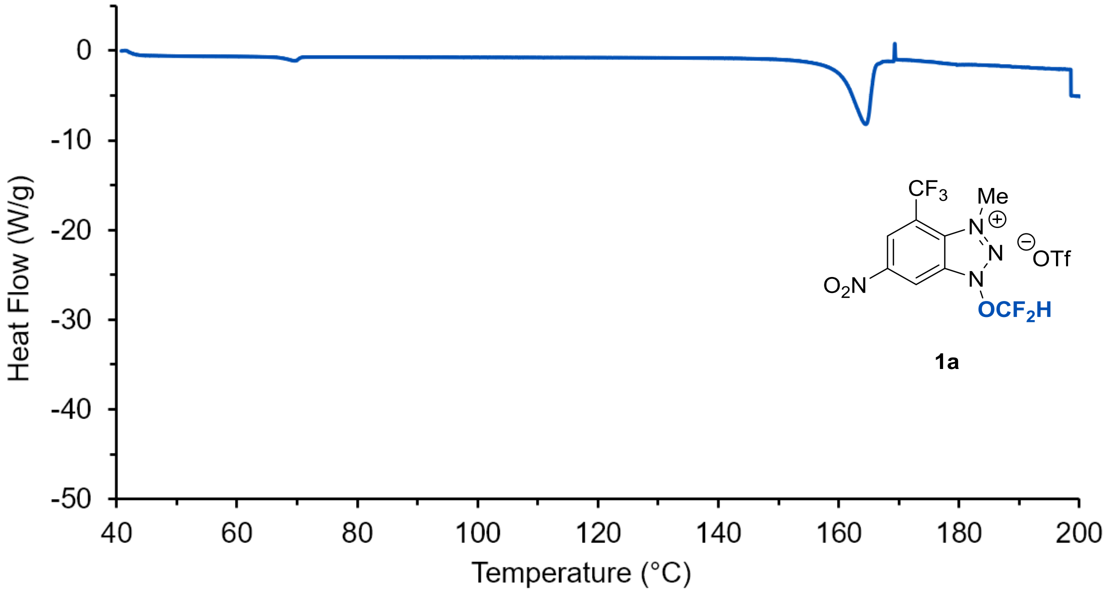

Figure S1. DSC of reagent **1a**.

Absorption and Emission Spectra

Absorption of reagent **1a** and emission spectrum of the 30 W blue LEDs.

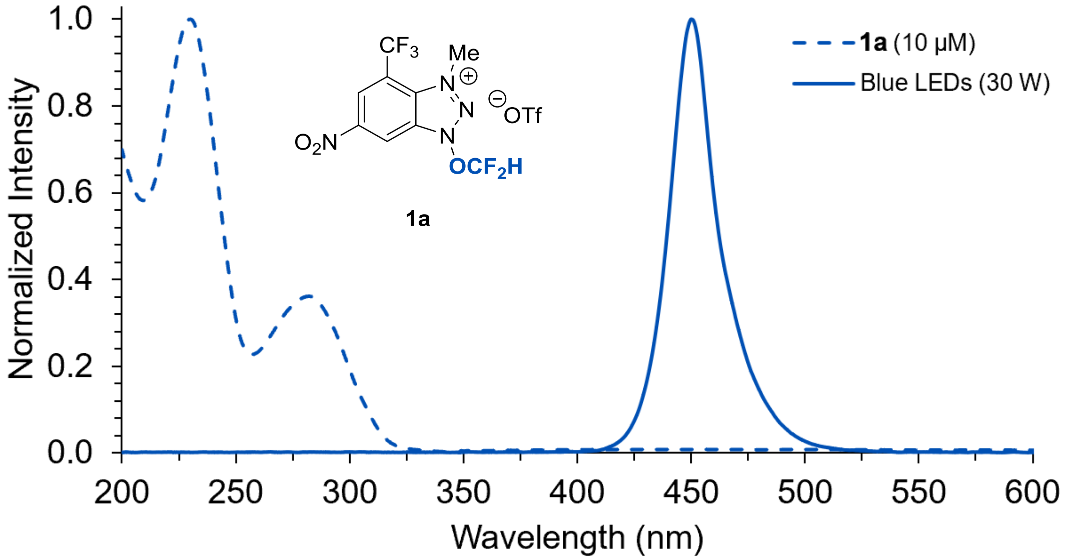

Figure S2. Absorption spectrum of **1a** (λ_{max} = 230 nm) and emission spectrum of the 30 W blue LEDs (λ_{max} = 450 nm).

Electron donor-acceptor (EDA) Complexes Study

Absorption of reagent **1a** with toluene and 3,4-dibromothiophene. Based on these absorption spectra no electron donor-acceptor was obvserved with **1a**.

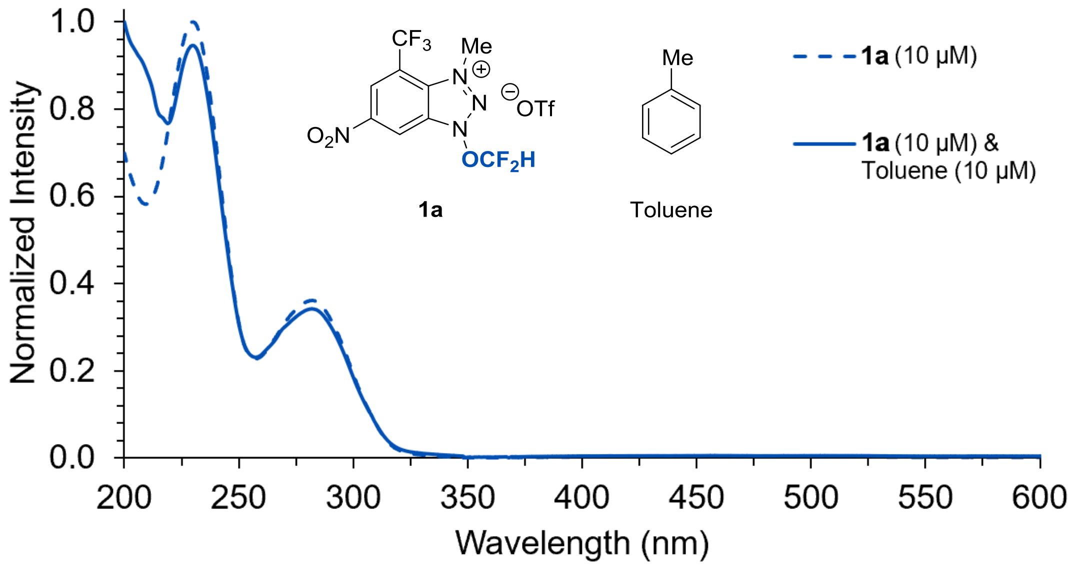

Figure S3. Absorption spectrum of **1a** (λ_{max} = 230 nm) and absorption spectrum **1a** with toluene.

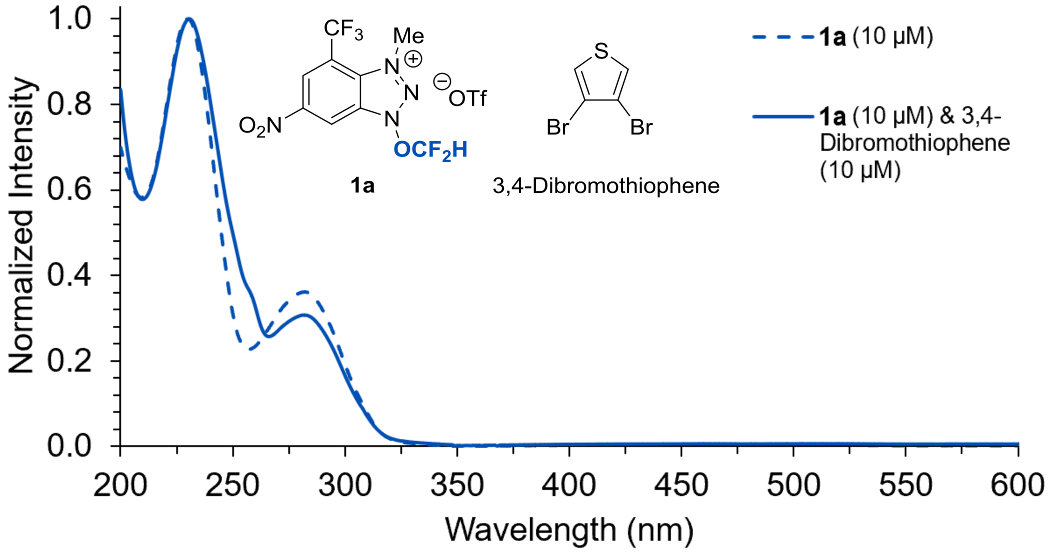

Figure S4. Absorption spectrum of **1a** (λ_{max} = 230 nm) and absorption spectrum **1a** with 3,4-Dibromothiophene.

Cyclic Voltammetry

Working Electrode: Glassy Carbon (3 mm diameter)

Counter Electrode: Platinum Wire.

Reference Electrode: Ag/AgCl (0.1 M). 5 mM compound, 0.1 M TBAPF$_6$ in CH_3CN, purged argon for 10 minutes before data collection. Scan rate: 100 mV s^{-1}.

Ferrocene Standard: 0.336 V vs Ag/AgCl electrode:

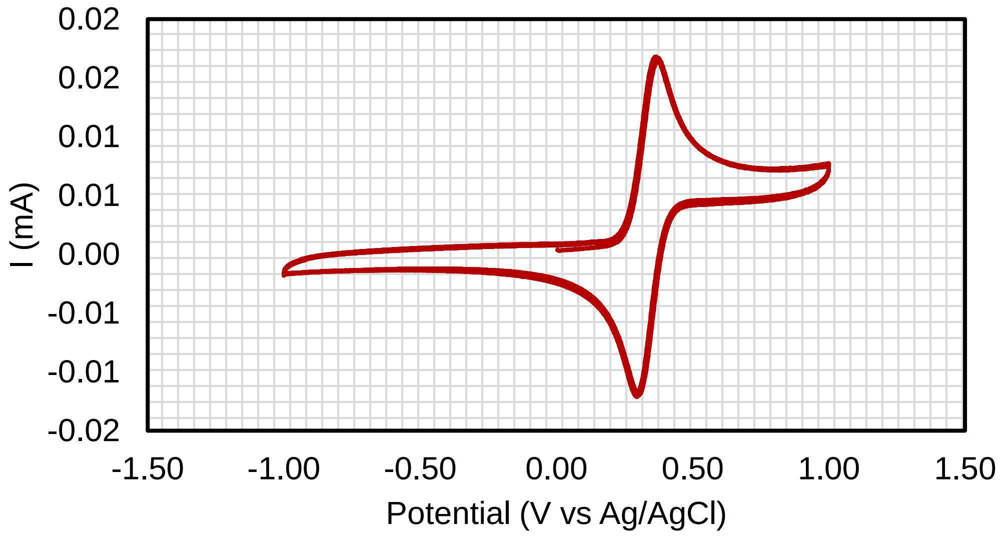

Figure S5. Cyclic Voltammertry of Fc/Fc$^+$ vs Ag/AgCl

Reagent 1a: 0.063 V vs Ag/AgCl, -0.273 V vs Fc/Fc+, (+0.109 V vs SCE):

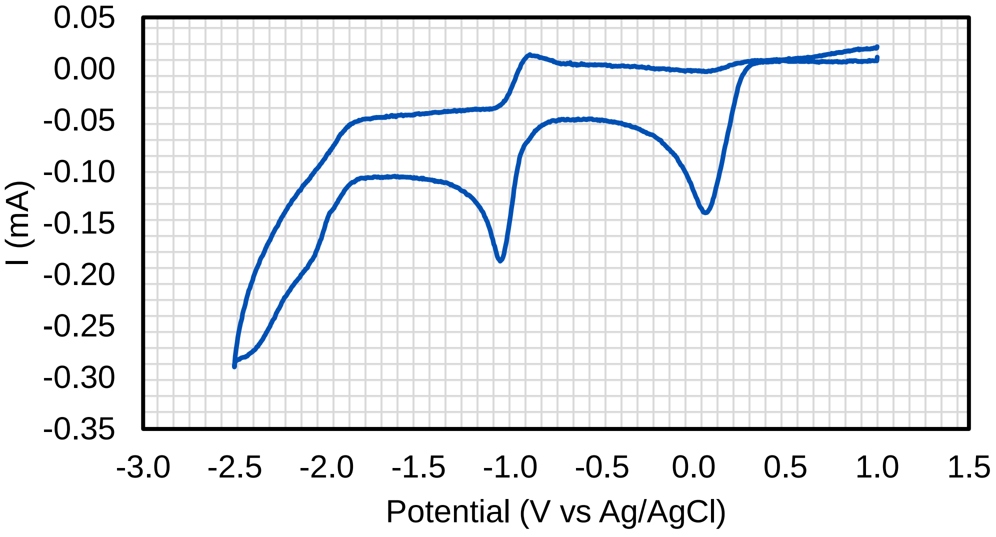

Figure S6. Cyclic Voltammertry of **1a** vs Ag/AgCl.

Mechanistic Studies

Stern–Volmer Luminescence Quenching

Emission intensities were recorded using a Perkin Elmer LS50B Luminescence spectrometer. All quenching data was recorded in the dark using a 1.00 cm screw-top quartz cuvette at 23 °C in the presence of $Ru(bpy)_3(PF_6)_2$ (3.00 μM) and varying concentration of quencher in degassed MeCN. Excitation of the sample was performed at 450 nm with a slit width of 10.0 nm and emission was detected at 615 nm. After acquisition, the data was plotted according to the Stern-Volmer equation shown below.

$$\frac{\mathbf{I_o}}{\mathbf{I}} = \mathbf{1} + \mathbf{K_{SV}}[\mathbf{Q}]$$

$$K_{SV} = k_q \tau_o$$

Where I_o is the luminescence intensity in the absence of the quencher, I is the intensity in the presence of the quencher, K_{SV} is the Stern−Volmer constant, k_q is the quenching rate, τ_o is the life-time of the photoredox catalyst ($\tau_o = 1.10 \times 10^{-6}$ s for $Ru(bpy)_3^{2+}$),[9] and [Q] is the concentration of the quencher.

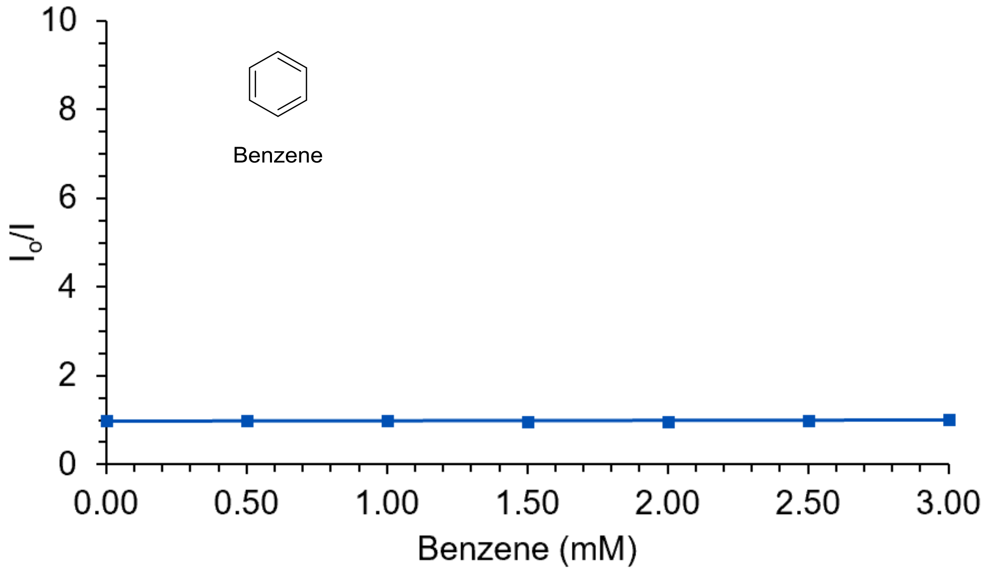

Figure S7. $Ru(bpy)_3^{2+}$ emission quenching of benzene. No observable quenching was detected.

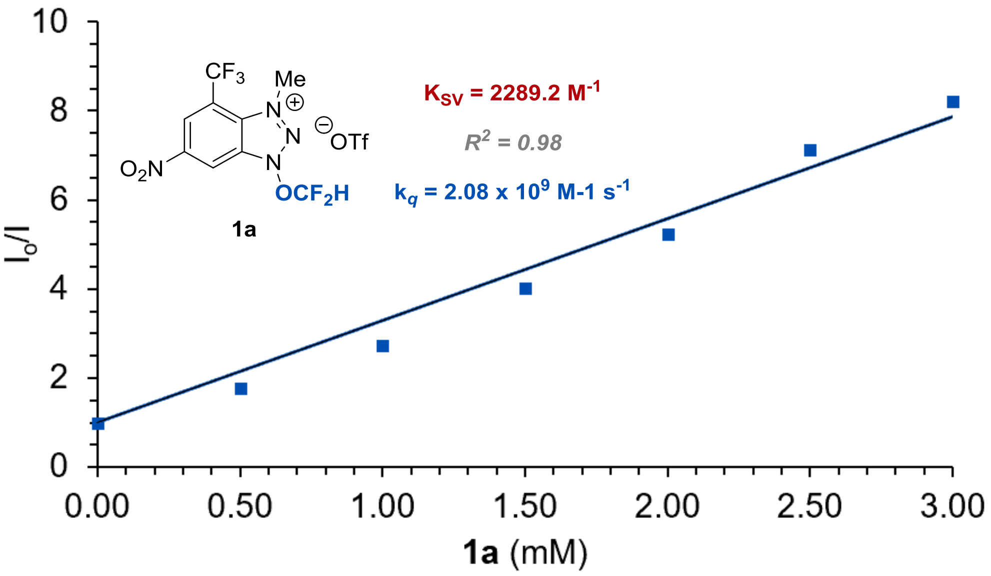

Figure S8. Ru(bpy)$_3^{2+}$ emission quenching of **1a**. $k_q = 2.08 \times 10^9$ M^{-1} s^{-1} was observed.

Quantum Yield Experiment

Quantum yield experiments suggest that an extended radical chain propagation is unlikely under our reaction conditions which is also support by (i) light on-and-off experiments (Figure S14) and (ii) DFT calculations (*DFT calculations suggest that SET between the Ru^{3+} and* ***IV*** *is thermodynamically more favourable than the chain reaction mechanism (see Figure S24 for more details).* The following quantum yield measurements are adapted from the procedure developed by Yoon *et al.*[10]

Determination of Fraction of Light Absorbed at 450 nm:

The fraction of light absorbed (*f*) by this solution was calculated as shown in Figure S10. Where the absorbance of the ferrioxalate solution at 450 nm was measured to be 1.84454, indicating f = 0.98567).

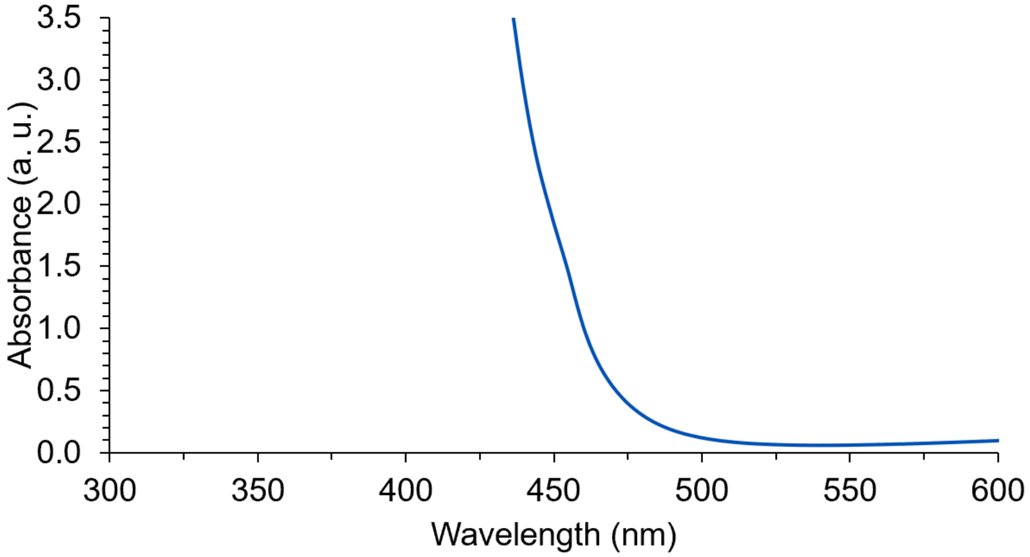

Figure S9. Absorbance of the ferrioxalate solution at 450 nm (A = 1.84454).

1. Fraction of light absorbed at 450 nm

$f = 1 - 10^{-A}$ A = 1.84454 *(Measured absorbance of ferrioxalate solution at 450 nm)*

$f = 1 - 10^{-1.84454} = 0.98567$

Figure S10. Determination of the fraction of light absorbed (*f*) by ferrioxalate solution at 450 nm.

Determination of the Light Intensity at 450 nm:

The photon flux of the 30 W Blue LEDs (λ_{max} = 450 nm) was determined by standard ferrioxalate actinometry.[11] A 0.150 M solution of ferrioxalate was prepared by dissolving 2.21 g of potassium ferrioxalate hydrate ($K_3[Fe(C_2O_4)_3] \cdot 3\ H_2O$) in 30.0 mL of 0.05 M H_2SO_4 (aq). Next, a buffered solution of phenanthroline was prepared by dissolving 50.0 mg of phenanthroline and 11.25 g of sodium acetate in 50.0 mL of 0.500 M H_2SO_4. Both solutions were stored in an amber vial in the dark. To determine the photon flux of the 30 W Blue LEDs, 2.00 mL of the ferrioxalate solution was placed in a cuvette and irradiated for 5.00 seconds at λ = 450 nm. After irradiation, 0.500 mL of the phenanthroline solution was added to the cuvette. The solution was then rested for 1 h in the dark to allow the ferrous ions to completely coordinate to the phenanthroline A non-irradiated sample was also prepared and developed in the dark as well (*note: after developing the non/irradiated samples they were diluted with a dilution factor of 4 to prevent deviation fro the Beer-Lambert law at high concentrations A = >2. Thus, to obtain the actual mol of Fe^{2+} they were multiplied by four. The values of the optical difference is the average of three trials*).

Afterwards, the absorbance of the both solutions were measured at 510 nm and with mol of Fe^{2+} known, next, the photon flux determined to be 1.80×10^{-7} einstein s^{-1}. We can obtain the quantum yield of our

reaction provided if it is irradiated using the same geometry (*note: although Φ = 1.01 at 436 nm was used for the calculation of the photon flux it known that the ferrioxalate system varied little with the wavelength as the Φ remained between 0.9 and 1.1 at wavelength between 400–480 nm*).[11a]

1. Ferrioxalate Actinometry

$$\text{mol } Fe^{2+} = 4 \cdot \left[\frac{V \cdot \Delta A_{510\text{ nm}}}{l \cdot \varepsilon_{510\text{ nm}}} \right]$$

V = 0.00250 L *(Total Volume)*

$\Delta A_{510\text{ nm}}$ = 0.91737 *(Optical Difference in Absorbance at 510 nm)*

l = 1.00 cm *(Path Length)*

$\varepsilon_{510\text{ nm}}$ = 11,100 L mol^{-1} cm^{-1} (*Molar Absorptivity of Fe at 510 nm*)

$$\text{mol } Fe^{2+} = 4 \cdot \left[\frac{0.000250\text{ L} \cdot 0.91737}{1.00\text{ cm} \cdot 11{,}100\text{ L mol}^{-1}\text{ cm}^{-1}} \right] = 8.27 \times 10^{-7}\text{ mol}$$

2. Determination of Photon Flux of 30 W Blue LEDs

$$\text{Photon Flux} = \frac{\text{mol } Fe^{2+}}{\Phi \cdot t \cdot f}$$

Φ = 1.01 (Quantum Yield of the Ferrioxalate Actinometer at 436 nm)

t = 5.00 s *(Time)*

f = 0.98568 *(fraction of light absorbed at 450 nm)*

$$\text{Photon Flux} = \frac{8.27 \times 10^{-7}\text{ mol}}{1.01 \cdot 5.00\text{ s} \cdot 0.98568} = 1.66 \times 10^{-7}\text{ einstein s}^{-1}$$

Figure S11. Determination of the light intensity (photon flux) at 450 nm via ferrioxalate actinometry (ε = 11,100 L mol^{-1} cm^{-1}).[11a]

Determination of Quantum Yield:

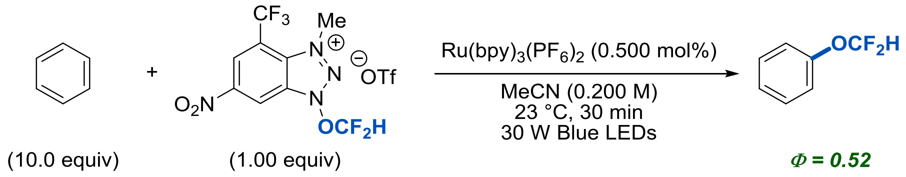

Figure S12. Qunatum yield of 0.52 was observed for the reaction.

In a glovebox, the same cuvette used to determine the photon flux was charged with 1-(difluoromethoxy)-3-methyl-6-nitro-4-(trifluoromethyl)-1*H*-benzo[*d*][1,2,3]triazol-3-ium trifluoromethanesulfonate (**1a**) (185 mg, 0.400 mmol, 1.00 equiv), benzene (212.1 mg, 358 μL, 4.00 mmol, 10.0 equiv), $Ru(bpy)_3(PF_6)_2$, (1.72 mg, 2.00 μmol, 0.500 mol%) in MeCN (2.00 mL, 0.200 M, with respect to **1a**). Afterwards the cuvette was capped with a PTFE stopper and taken out of the glovebox. The reaction mixture was irradiated (λ_{max} = 450 nm) for 1800 s (30 min) with the same 30 W Blue LEDs. To determine the yield of the product, an internal standard, trifluorotoluene ($PhCF_3$) (29.2 mg, 24.6 μL, 0.200 mmol, 0.500 equiv) was added to the cuvette. Then, a 200 μL of the reaction mixture was taken and then dilute with 500 μL CD_3CN followed by ^{19}F NMR. The quantum yield was determined using the equation shown below.

1. Quantum Yield:

$$\Phi = \frac{mol_{1a} \cdot Yield}{photon\ flux \cdot t \cdot f}$$

mol_{1a} = 0.00004 mol *(mol of 1a)*
Yield = 39% *(Yield of desired product)*
t = 1800 s *(time)*
f = 1 *(fraction of light absorbed by* $Ru(bpy)_3^{2+}$ *at 450 nm)*

$$\Phi = \frac{0.0004\ mol \cdot 39\%}{1.66 \times 10^{-7}\ einstein\ s^{-1} \cdot 1800\ s \cdot 1.00} = \mathbf{0.52}$$

Figure S13. Determination of quantum yield of the reaction.

Light On/Off Experiment

Based on the result of light on and off experiment, it was observed that the transformation proceeded smoothly under light, but no further conversion was observed when the light is turned off. This result suggests that a long-lived radical chain propagation is unlikely (*note: quantum yield measurements are the best way to determine a radical chain mechanism).*

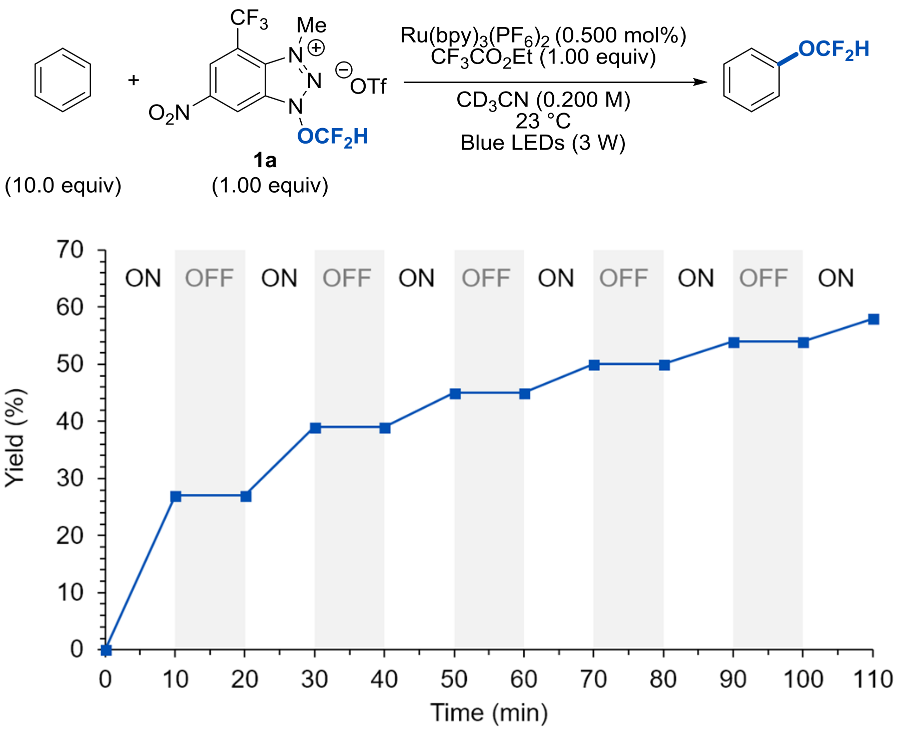

Figure S14. Light on-and-off experiments of difluoromethoxylation reaction of benzene

In a glovebox, to an vacuum-dried screw cap NMR tube was added a solution of 1-(difluoromethoxy)-3-methyl-6-nitro-4-(trifluoromethyl)-1*H*-benzo[*d*][1,2,3]triazol-3-ium trifluoromethanesulfonate (**1a**) (46.2 mg, 0.100 mmol, 1.00 equiv), benzene (78.1 mg, 89.4 µL, 1.00 mmol, 10.0 equiv), ethyl trifluoroacetate (14.2 mg, 11.9 µL, 0.100 mmol, 10.0 equiv) (an internal standard, to determine the yield of the product),

$Ru(bpy)_3(PF_6)_2$, (0.430 mg, 0.500 μmol, 0.500 mol%) in CD_3CN (0.500 mL, 0.200 M, with respect to **1a**). Afterwards the NMR tube was capped and taken out of the glovebox. The reaction mixture was irradiated alternatively at ambient temperature (23 °C) with blue LEDs (3 W, λ_{max} = 450 nm) which was placed 30.0 mm from the NMR tube and kept in the dark in 10 minutes intervals.

Reaction without $Ru(bpy)_3^{2+}$

We observed that in the absence of $Ru(bpy)_3^{2+}$ we could detect a trace amount of the desired product (<5% yield). When we proformed the same reaction with longer reaction time, we could observe the slugished formation of the desired product in <9% yield over a peroids of 3 days (72 hours).

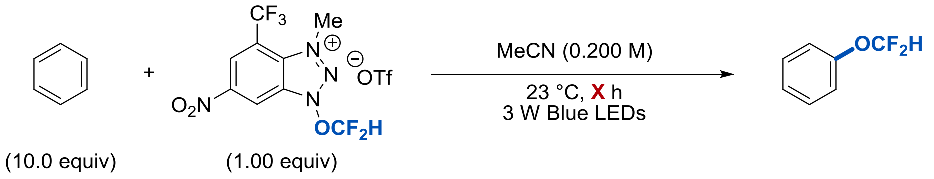

Entry	X h	Yield (%)
1	12	<5%
2	24	<5%
3	48	<6%
4	72	<9%

Figure S15. Reaction without $Ru(bpy)_3^{2+}$.

In a glovebox, to an oven-dried 4 mL screw cap vial was added 1-(difluoromethoxy)-3-methyl-6-nitro-4-(trifluoromethyl)-1*H*-benzo[*d*][1,2,3]triazol-3-ium trifluoromethanesulfonate (**1a**) (9.24 mg, 0.0200 mmol, 1.00 equiv), benzene (15.6 mg, 17.9 μL, 200 mmol, 10.0 equiv), and MeCN (0.100 mL, 0.200 M, with respect to **1a**). To this solution was added a magnetic stir bar. Next, the reaction vial was capped and taken out of the glovebox. The reaction mixture was stirred at ambient temperature (23 °C) and irradiated with blue LEDs (3 W, λ_{max} = 450 nm) which was placed 20.0 mm from the vial for 12 h. To determine the yield of the products, an internal standard, trifluorotoluene ($PhCF_3$) (1.46 mg, 1.23 μL, 0.0100 mmol, 0.500 equiv) was added to the vial. Then, the reaction mixture was diluted with 500 μL of $CDCl_3$ followed by ^{19}F NMR.

Bandpass Filter Experiment

Note: We observed reagent ***1a*** *can absorb small amounts of visible light (λ = <400 nm at >0.01 mM).*

To exclude the possibility of the catalytic reaction occuring via light promoted homolysis of the N–O bond, we performed a reaction using a λ_{max} = 488 bandpass filter where the reagent does not absorb light but

$Ru(bpy)_3^{2+}$ does. As shown in the figures below, even with the 488 nm bandpass filter the reaction still proceeded without any diminished yields. These results suggests that photoexcitation of $Ru(bpy)_3^{2+}$ is necessary and the OCF_2H radical is likely generated through single electron transfer between the excited $*Ru(bpy)_3^{2+}$ complex and reagent **1a**.

Emission Spectrum of 30 W Blue LEDs with and without 488 nm Bandpass Filter

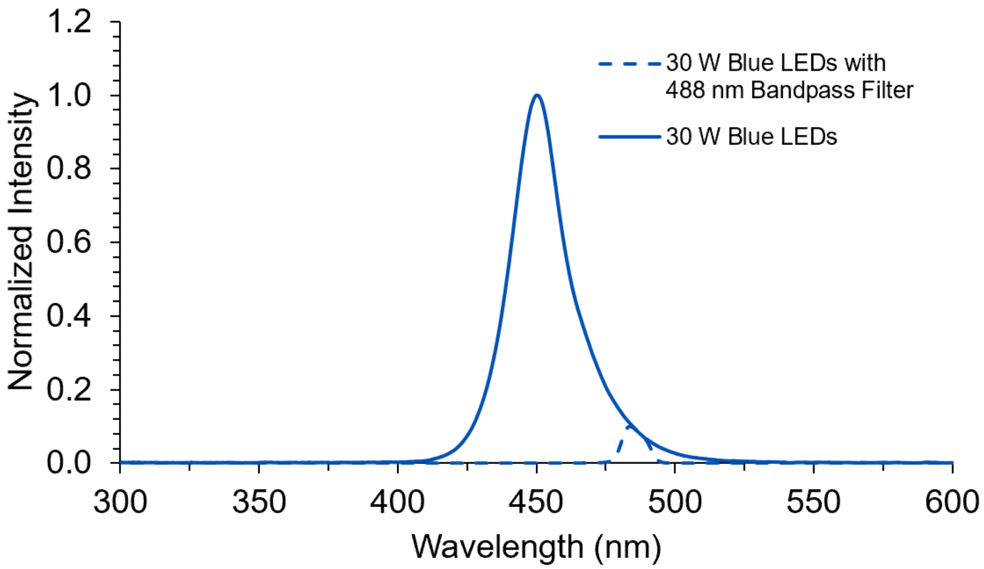

Figure S16. Emission spectrum of the 30 W blue LEDs (λ_{max} = 450 nm) and emission spectrum of the 30 W blue LEDs with 488 nm bandpass filter (λ_{max} = 488 nm).

Standard Reaction using 30 W Blue LEDs with 488 nm Bandpass Filter

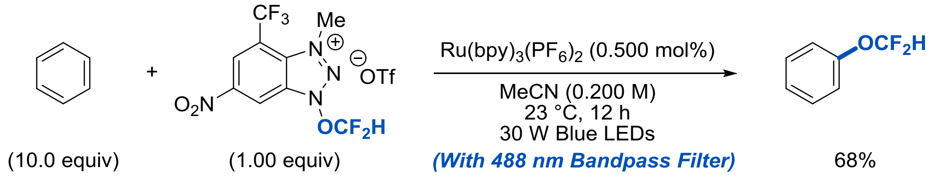

Figure S17. Standard reaction with 488 nm bandpass filter

In a glovebox, to an oven-dried 4 mL screw cap vial was added 1-(difluoromethoxy)-3-methyl-6-nitro-4-(trifluoromethyl)-1*H*-benzo[*d*][1,2,3]triazol-3-ium trifluoromethanesulfonate (**1a**) (9.24 mg, 0.0200 mmol, 1.00 equiv), benzene (15.6 mg, 17.9 μL, 200 mmol, 10.0 equiv), $Ru(bpy)_3(PF_6)_2$, (0.0860 mg, 0.100 μmol, 0.500 mol%), and MeCN (0.100 mL, 0.200 M, with respect to **1a**). To this solution was added a magnetic stir bar. Next, the reaction vial was capped and taken out of the glovebox. The reaction mixture was stirred at ambient temperature (23 °C) and irradiated with blue LEDs (30 W with 488 nm bandpass filter, λ_{max} = 488 nm) which was placed 20.0 mm from the vial for 12 h. To determine the yield of the products, an

internal standard, trifluorotoluene ($PhCF_3$) (1.46 mg, 1.23 μL, 0.0100 mmol, 0.500 equiv) was added to the vial. Then, the reaction mixture was diluted with 500 μL of $CDCl_3$ followed by ^{19}F NMR.

Intermolecular Kinetic Isotope Effect

We have performed deuterium kinetic isotope effect study using 5 equiv of benzene and 5 equiv of d_6-benzene in the presence of 1 equiv of reagent **1a**. The desired products Ph-OCF_2H and d_5-Ph-OCF_2H were obtained in 36% and 36% yields, respectively. This result rules out the possibility of H-atom abstraction/deprotonation as the rate-determining step

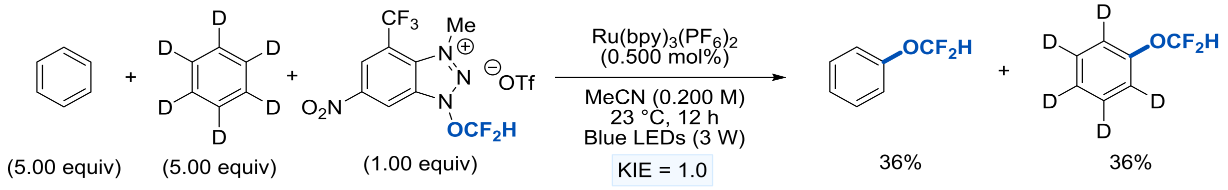

Figure S18. Deuterium kinetic isotope effect study using 5 equiv of benzene and 5 equiv of d_6-benzene.

In a glovebox, to an oven-dried 4 mL screw cap vial was added 1-(difluoromethoxy)-3-methyl-6-nitro-4-(trifluoromethyl)-1*H*-benzo[*d*][1,2,3]triazol-3-ium trifluoromethanesulfonate (**1a**) (9.24 mg, 0.0200 mmol, 1.00 equiv), benzene (7.81 mg, 8.94 μL, 100 mmol, 5.00 equiv), hexadeuterobenzene (8.42 mg, 8.86 μL, 0.100 mmol, 5.00 equiv), $Ru(bpy)_3(PF_6)_2$, (0.0860 mg, 0.100 μmol, 0.500 mol%), and MeCN (0.100 mL, 0.200 M, with respect to **1a**). To this solution was added a magnetic stir bar. Next, the reaction vial was capped and taken out of the glovebox. The reaction mixture was stirred at ambient temperature (23 °C) and irradiated with blue LEDs (3 W, λ_{max} = 450 nm) which was placed 20.0 mm from the vial for 12 h. To determine the yield of the products, an internal standard, trifluorotoluene ($PhCF_3$) (1.46 mg, 1.23 μL, 0.0100 mmol, 0.500 equiv) was added to the vial. Then, the reaction mixture was diluted with 500 μL of $CDCl_3$ followed by ^{19}F NMR.

Intermolecular Competition Experiment

Reaction of the OCF2H Radical with Electron Rich and Electron Deficient Arenes:

We have performed competition reactions, and as anticipated, the electrophilic OCF_2H radical react faster with electron-rich arenes.

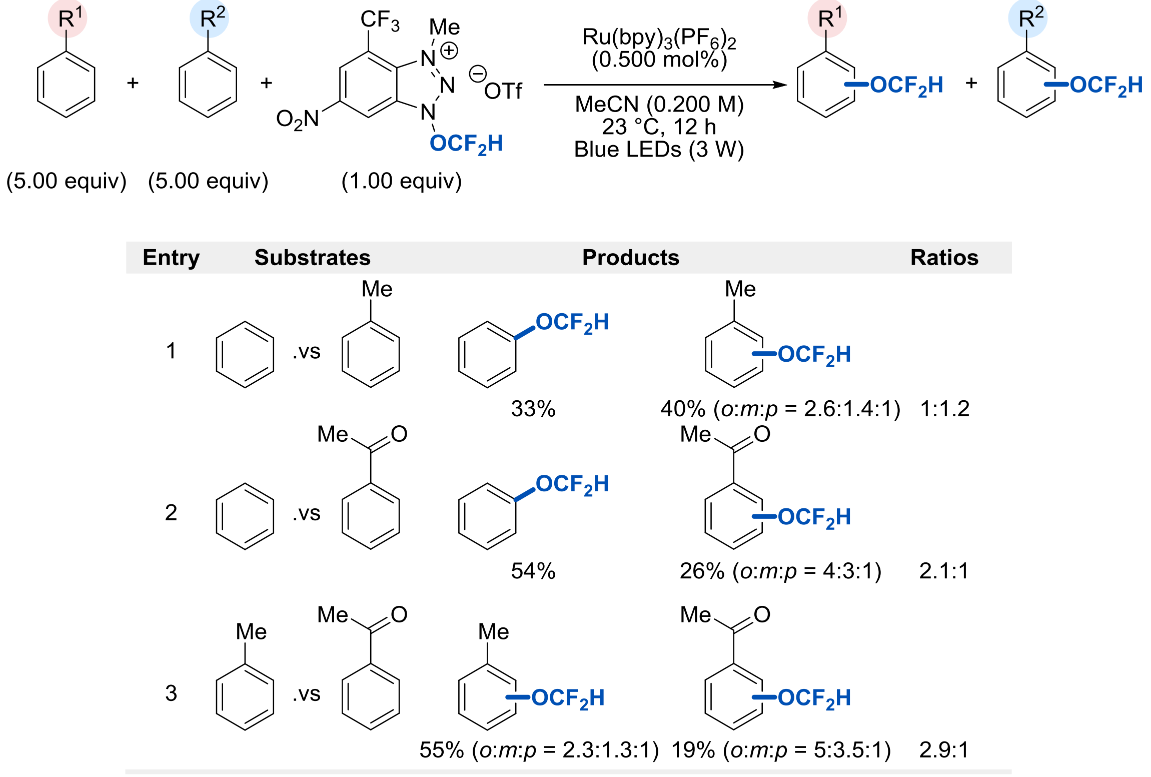

Figure S19. Intermolecular competition experiment of difluoromethoxylation reactions

In a glovebox, to an oven-dried 4 mL screw cap vial was added 1-(difluoromethoxy)-3-methyl-6-nitro-4-(trifluoromethyl)-1*H*-benzo[*d*][1,2,3]triazol-3-ium trifluoromethanesulfonate (**1a**) (9.24 mg, 0.0200 mmol, 1.00 equiv), arene (0.100 mmol, 5.00 equiv), arene (0.100 mmol, 5.00 equiv), $Ru(bpy)_3(PF_6)_2$, (0.0860 mg, 0.100 μmol, 0.500 mol%), and MeCN (0.100 mL, 0.200 M, with respect to **1a**). To this solution was added a magnetic stir bar. Next, the reaction vial was capped and taken out of the glovebox. The reaction mixture was stirred at ambient temperature (23 °C) and irradiated with blue LEDs (3 W, λ_{max} = 450 nm) which was placed 20.0 mm from the vial for 12 h. To determine the yield of the products, an internal standard, trifluorotoluene ($PhCF_3$) (1.46 mg, 1.23 μL, 0.0100 mmol, 0.500 equiv) was added to the vial. Then, the reaction mixture was diluted with 500 μL of $CDCl_3$ followed by ^{19}F NMR.

Relative Reactivity of the OCF_2H Radical and the OCF_3 Radical

Since we have cationic OCF_2H reagent (**1a**) and cationic OCF_3 reagent, their relative reactivity was explored. These two reagents have similar reduction potentials: the OCF_2H reagent (**1a**, +0.109 V vs SCE in MeCN) and the OCF_3 reagent (+0.140 V vs SCE in MeCN).[12] When we subjected these two reagents to the standard reaction conditions, we observed a product distribution of 1:2.7 (OCF_2H product:OCF_3 product). These results implicate that the OCF_3 radical likely reacts with an arene faster than the OCF_2H radical.

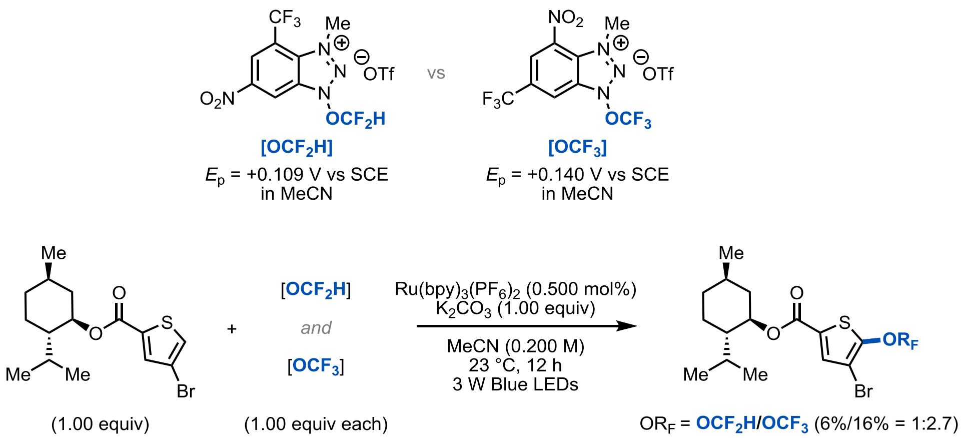

Figure S20. Relative reactivity of the OCF_2H radical and the OCF_3 radical.

In a glovebox, to an oven-dried 4 mL screw cap vial was added 1-(difluoromethoxy)-3-methyl-6-nitro-4-(trifluoromethyl)-1*H*-benzo[*d*][1,2,3]triazol-3-ium trifluoromethanesulfonate (**1a**) (9.24 mg, 0.0200 mmol, 1.00 equiv), 1-(trifluoromethoxy)-3-methyl-4-nitro-6-(trifluoromethyl)-1*H*-benzo[*d*][1,2,3]triazol-3-ium trifluoromethanesulfonate (9.60 mg, 0.0200 mmol, 1.00 equiv), arene (0.0200 mmol, 1.00 equiv), $Ru(bpy)_3(PF_6)_2$, (0.0860 mg, 0.100 μmol, 0.500 mol%), and MeCN (0.100 mL, 0.200 M, with respect to arene). To this solution was added a magnetic stir bar. Next, the reaction vial was capped and taken out of the glovebox. The reaction mixture was stirred at ambient temperature (23 °C) and irradiated with blue LEDs (3 W, λ_{max} = 450 nm) which was placed 20.0 mm from the vial for 12 h. To determine the yield of the products, an internal standard, trifluorotoluene ($PhCF_3$) (1.46 mg, 1.23 μL, 0.0100 mmol, 0.500 equiv) was added to the vial. Then, the reaction mixture was diluted with 500 μL of $CDCl_3$ followed by ^{19}F NMR.

Radical Probe Experiments

Butylated Hydroxytoluene (BHT) as a Radical Trap

Under our standard conditions we added 1 equivalent of butylated hydroxytoluene (BHT) and observed a dramatic decrease in the yield of the desired product indicating the likelihood of a radical mechanism (Figure S21).

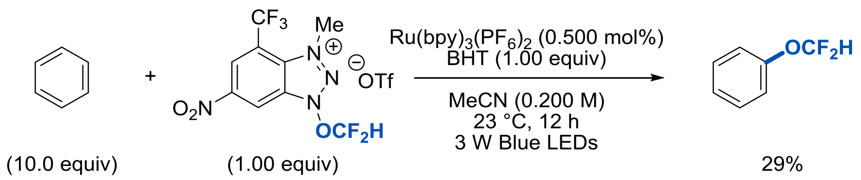

Figure S21. Addition of butylated hydroxytoluene (BHT) leads to diminished yield indicating the possibily of a radical mechanism.

In a glovebox, to an oven-dried 4 mL screw cap vial was added 1-(difluoromethoxy)-3-methyl-6-nitro-4-(trifluoromethyl)-1*H*-benzo[*d*][1,2,3]triazol-3-ium trifluoromethanesulfonate (**1a**) (9.24 mg, 0.0200 mmol, 1.00 equiv), benzene (17.8 μL, 15.6 mg, 0.200 mmol, 10.0 equiv), Ru(bpy)$_3$(PF$_6$)$_2$, (0.0860 mg, 0.100 μmol, 0.500 mol%), butylated hydroxytoluene (4.40 mg, 20.0 μmol, 1 equiv), and MeCN (0.100 mL, 0.200 M, with respect to **1a**). To this solution was added a magnetic stir bar. Next, the reaction vial was capped and taken out of the glovebox. The reaction mixture was stirred at ambient temperature (23 °C) and irradiated with blue LEDs (3 W, λ_{max} = 450 nm) which was placed 20.0 mm from the vial for 12 h. To determine the yield of the products, an internal standard, trifluorotoluene (PhCF$_3$) (1.46 mg, 1.23 μL, 0.0100 mmol, 0.500 equiv) was added to the vial. Then, the reaction mixture was diluted with 500 μL of CDCl$_3$ followed by ^{19}F NMR.

1,4-Cyclohexadiene as a Radical Probe

We hypothesized that if the OCF$_2$H radical is formed, it undergoes two consecutive H-atom abstraction from 1,4-cyclohexadiene, generating benzene as the product. Subsequently, this benzene can react with the OCF$_2$H radical under photocatalytic conditions, furnishing the difluoromethoxylated product. Indeed under standard reaction conditions using 1,4-dicyclohexadiene as a substrate, we observed 7% of the difluoromethoxylated benzene (Figure S22). Additionally, HPLC analysis of the reaction mixture indicated the formation of benzene as well (Figure S23).

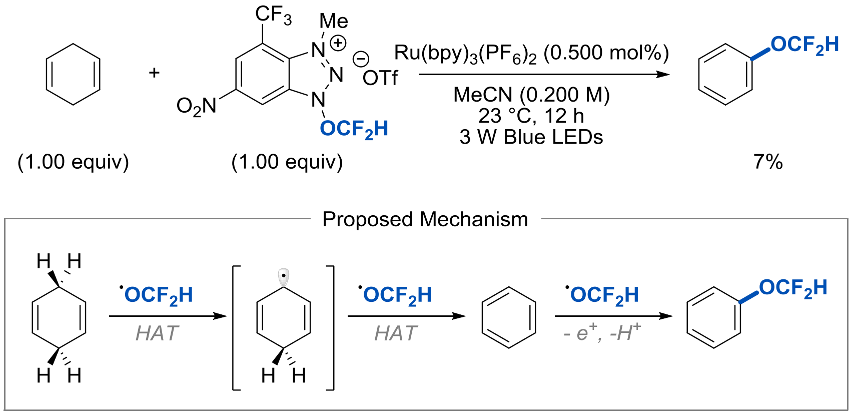

Figure S22. 1,4-Cyclohexadiene as a radical probe for the difluoromethoxyl radical.

In a glovebox, to an oven-dried 4 mL screw cap vial was added 1-(difluoromethoxy)-3-methyl-6-nitro-4-(trifluoromethyl)-1*H*-benzo[*d*][1,2,3]triazol-3-ium trifluoromethanesulfonate (**1a**) (9.24 mg, 0.0200 mmol, 1.00 equiv), freshly fractionally distilled 1,4-cyclohexadiene (1.89 μL, 1.60 mg, 0.0200 mmol, 1.00 equiv), $Ru(bpy)_3(PF_6)_2$, (0.0860 mg, 0.100 μmol, 0.500 mol%), and MeCN (0.100 mL, 0.200 M, with respect to **1a**). To this solution was added a magnetic stir bar. Next, the reaction vial was capped and taken out of the glovebox. The reaction mixture was stirred at ambient temperature (23 °C) and irradiated with blue LEDs (3 W, λ_{max} = 450 nm) which was placed 20.0 mm from the vial for 12 h. To determine the yield of the products, an internal standard, trifluorotoluene ($PhCF_3$) (1.46 mg, 1.23 μL, 0.0100 mmol, 0.500 equiv) was added to the vial. Then, the reaction mixture was diluted with 500 μL of $CDCl_3$ followed by ^{19}F NMR.

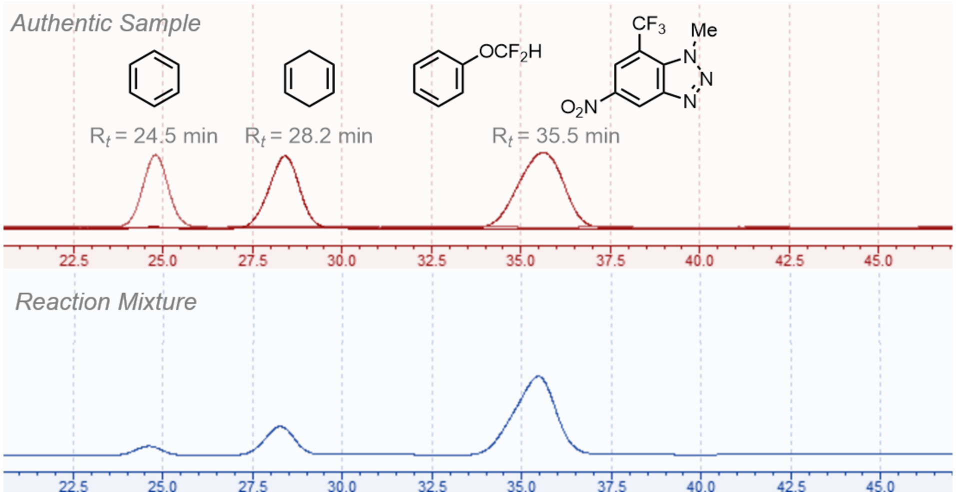

Figure S23. HPLC of authentic samples and anyalsis of the crude reaction mixture using 40% (v/v) acetonitrile in water.

Density Functional Theory (DFT) Calculations

Computational Details

All DFT calculations were performed with the Gaussian 09[13] software package. Geometries were optimized using the M06-2X[14] functional and the 6-31+G(d) basis set in gas phase. Single point energies were calculated using M06-2X and 6-311++G(d,p) and the SMD[15] solvation model in MeCN. Reported Gibbs free energies and enthalpies in solution include thermal corrections computed at 298 K. The experimental standard reduction potential (SRP) of Ru*$(bpy)_3^{2+}$ (−0.81 V vs. SCE in MeCN)[16] was used in the computations of the reaction Gibbs free energies of the single electron transfer (SET) processes with the photoredox catalyst. The detailed computational procedure was described in a recent computational study from Liu group[8].

Energies of photocatalytic difluoromethoxylation of benzene

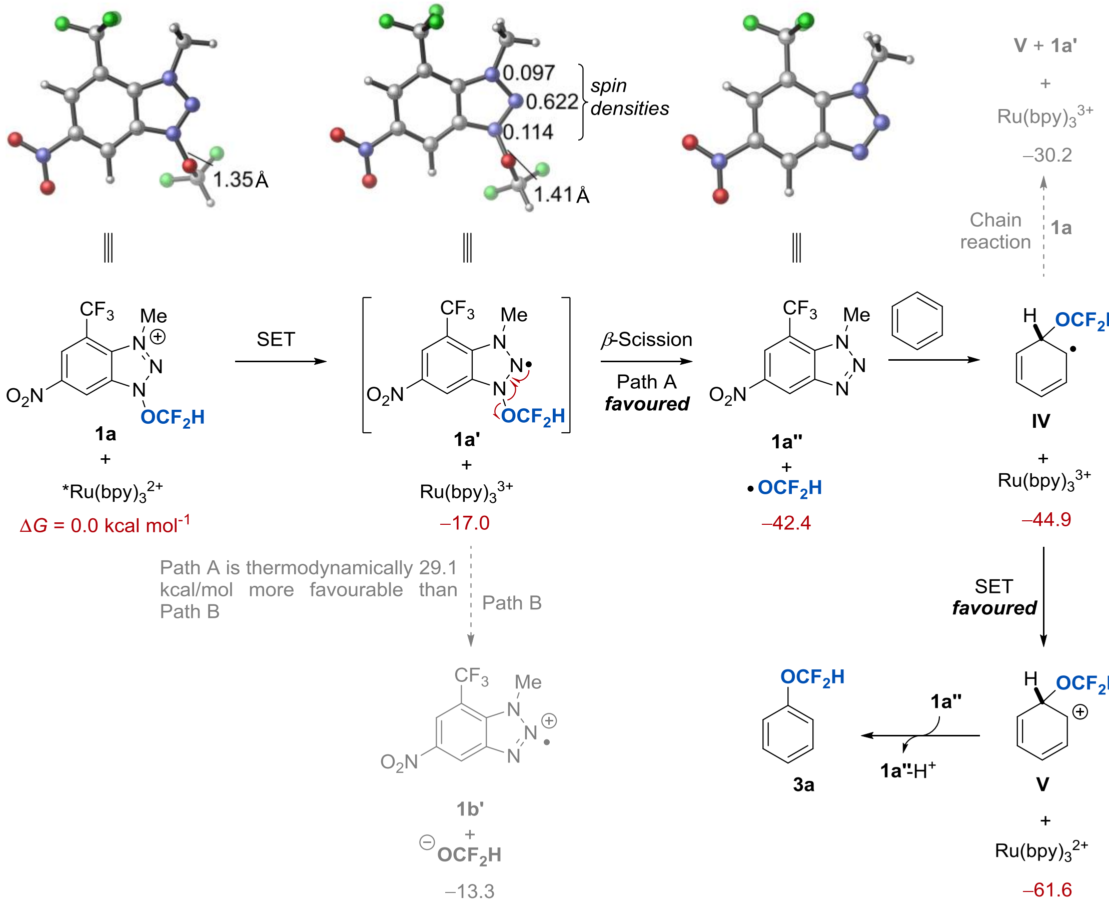

Figure S24. Energies of photocatalytic difluoromethoxylation of benzene. All energies are in kcal/mol and are with respect to **1a** and *$Ru(bpy)_3^{2+}$. The N-O bond distances in **1a** and **1a'** are in Å. The Mulliken spin densities in **1a'** are provided.

References

1. W. C. Still, M. Kahn and A. Mitra, *J. Org. Chem.*, 1978, **43**, 2923-2925.
2. G. R. Fulmer, A. J. M. Miller, N. H. Sherden, H. E. Gottlieb, A. Nudelman, B. M. Stoltz, J. E. Bercaw and K. I. Goldberg, *Organometallics*, 2010, **29**, 2176-2179.
3. C. Huang, T. Liang, S. Harada, E. Lee and T. Ritter, *J. Am. Chem. Soc.*, 2011, **133**, 13308-13310.
4. a) K. N. Hojczyk, P. J. Feng, C. B. Zhan and M. Y. Ngai, *Angew. Chem. Int. Ed.*, 2014, **53**, 14559-14563; b) P. Feng, K. N. Lee, J. W. Lee, C. Zhan and M.-Y. Ngai, *Chem. Sci.*, 2016, **7**, 424-429.
5. Y. Zafrani, G. Sod-Moriah and Y. Segall, *Tetrahedron*, 2009, **65**, 5278-5283.
6. J. Yang, M. Jiang, Y. Jin, H. Yang and H. Fu, *Org. Lett.*, 2017, **19**, 2758-2761.
7. U. Salar, K. M. Khan, M. Taha, N. H. Ismail, B. Ali, S. Perveen, M. Ghufran and A. Wadood, *Eur. J. Med. Chem.*, 2017, **125**, 1289-1299.
8. W. Zheng, C. A. Morales-Rivera, J. W. Lee, P. Liu and M. Y. Ngai, *Angew. Chem. Int. Ed.*, 2018, **57**, 9645-9649.
9. A. Juris, V. Balzani, P. Belser and A. von Zelewsky, *Hel. Chim. Acta*, 1981, **64**, 2175-2182.
10. M. A. Cismesia and T. P. Yoon, *Chem. Sci.*, 2015, **6**, 5426-5434.
11. a) C. G. Hatchard, C. A. Parker and J. Bowen Edmund, *Proc. R. Soc. Lond. A Math. Phy. Sci.*, 1956, **235**, 518-536; b) H. J. Kuhn, S. E. Braslavsky and R. Schmidt, *Journal*, 2004, **76**, 2105-2146; c) M. Montalti and S. L. Murov, *Handbook of Photochemistry*, CRC/Taylor & Francis, Boca Raton, 3rd Ed., 2006.
12. W. Zheng, J. W. Lee, C. A. Morales-Rivera, P. Liu and M. Y. Ngai, *Angew. Chem. Int. Ed.*, 2018, **57**, 13795-13799.
13. M. Frisch, G. Trucks, H. Schlegel, G. Scuseria, M. Robb, J. Cheeseman, G. Scalmani, V. Barone, B. Mennucci and G. Petersson, *There is no corresponding record for this reference*.
14. Y. Zhao and D. G. Truhlar, *Theor. Chem. Acc.*, 2008, **120**, 215-241.
15. A. V. Marenich, C. J. Cramer and D. G. Truhlar, *J. Phys. Chem. B*, 2009, **113**, 6378-6396.
16. C. R. Bock, T. J. Meyer and D. G. Whitten, *J. Am. Chem. Soc.*, 1975, **97**, 2909-2911.

Spectroscopic Data of Difluoromethoxylation Reactions

^{1}H NMR (700 MHz, $(CD_3)_2SO$, 25 ºC) of S1a

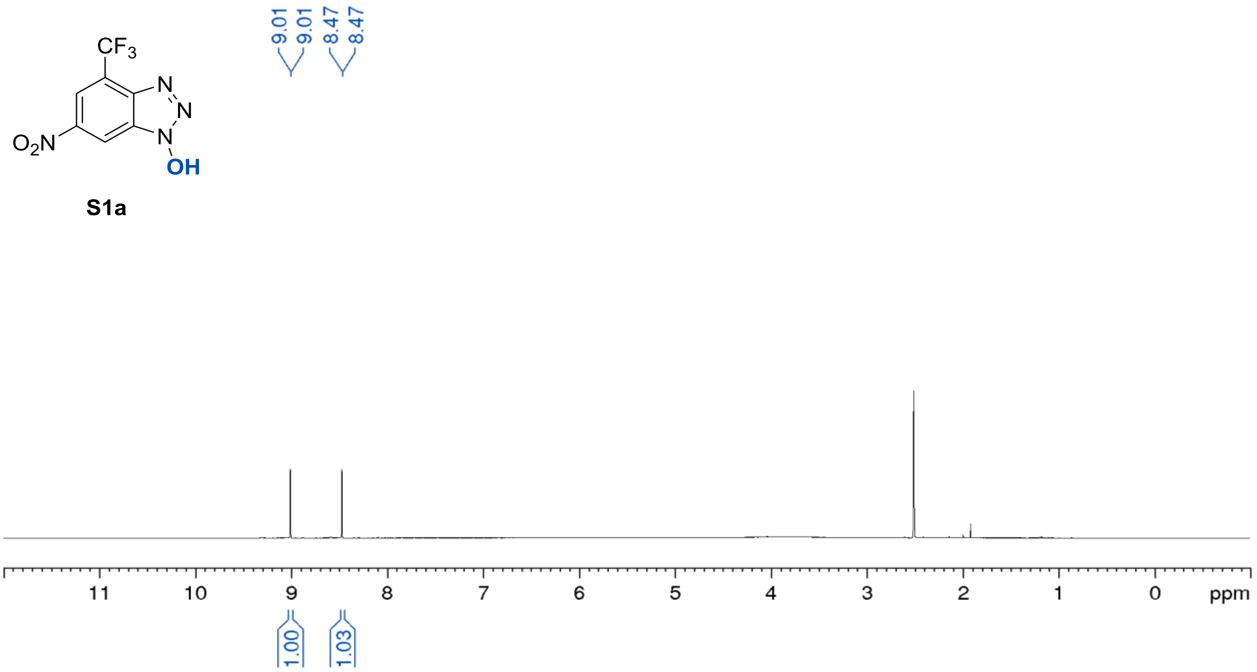

S1a

^{13}C NMR (175 MHz, $(CD_3)_2SO$, 25 ºC) of S1a

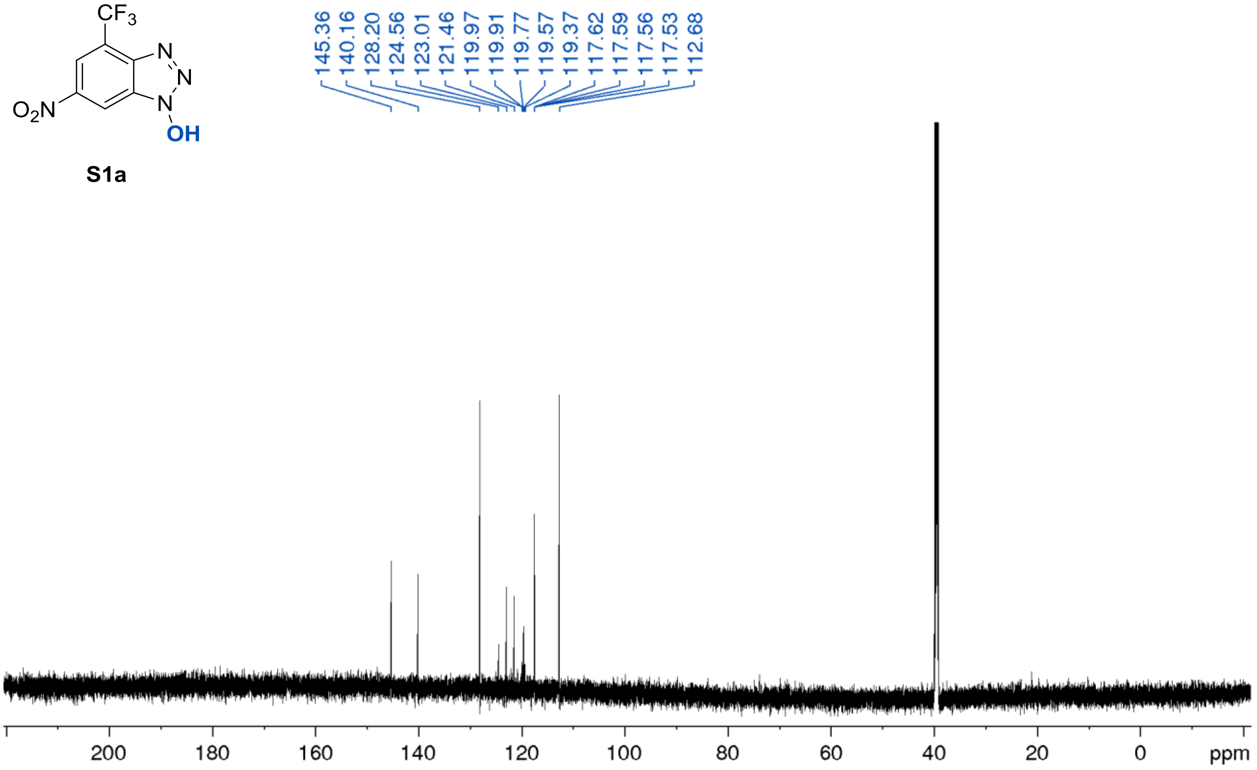

S1a

^{19}F NMR (376 MHz, $(CD_3)_2SO$, 25 °C) of S1a

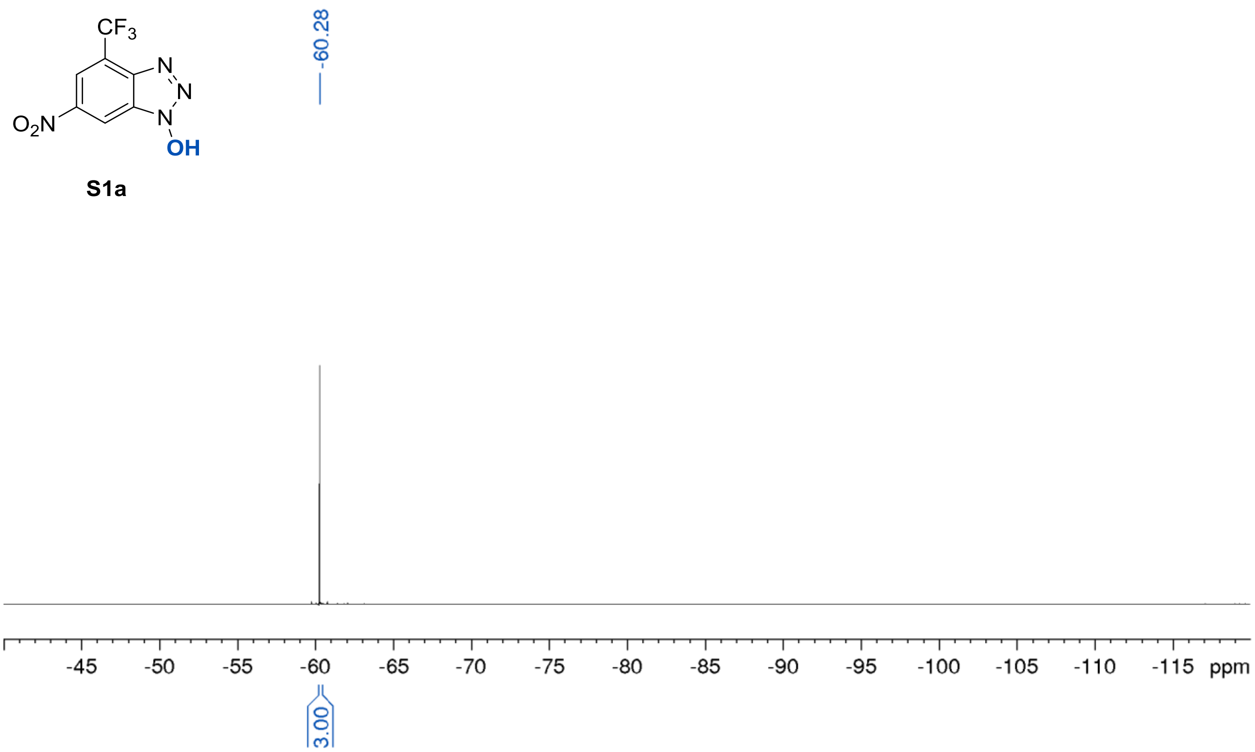

^{1}H NMR (700 MHz, $CDCl_3$, 25 °C) of DR1

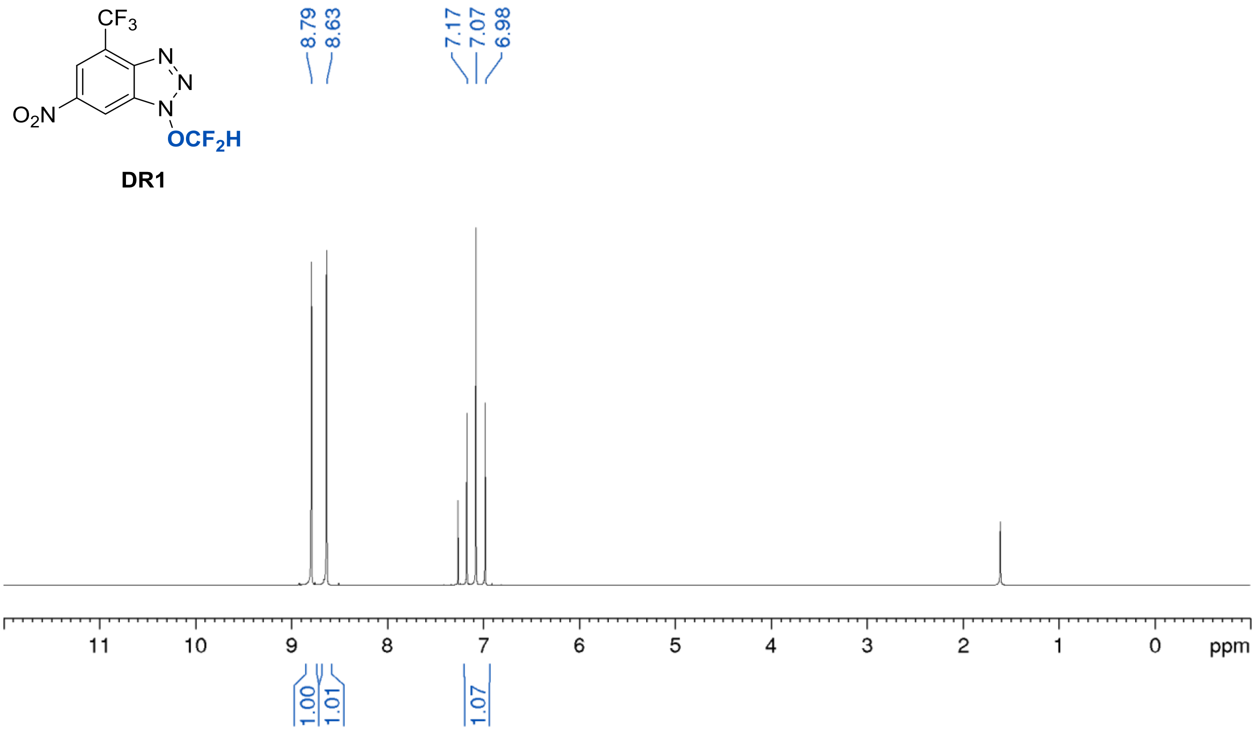

^{13}C NMR (175 MHz, $CDCl_3$, 25 ºC) of DR1

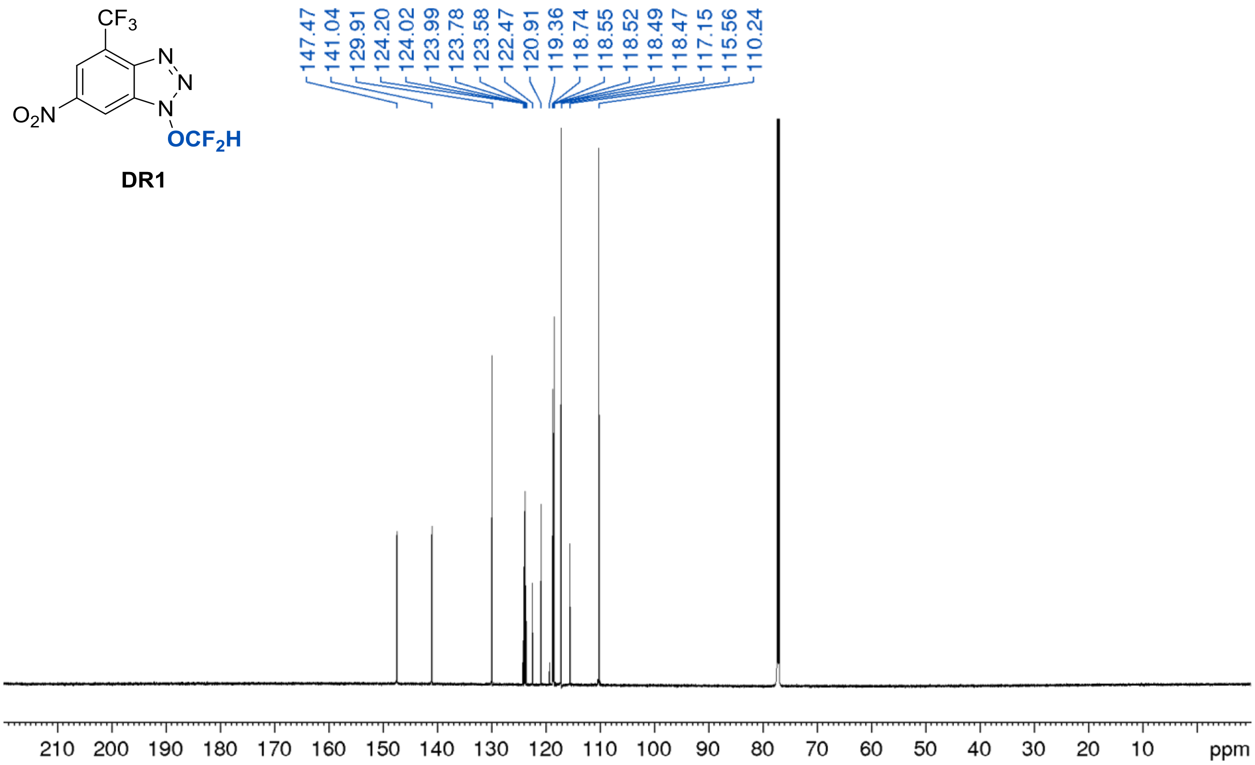

^{19}F NMR (376 MHz, $CDCl_3$, 25 ºC) of DR1

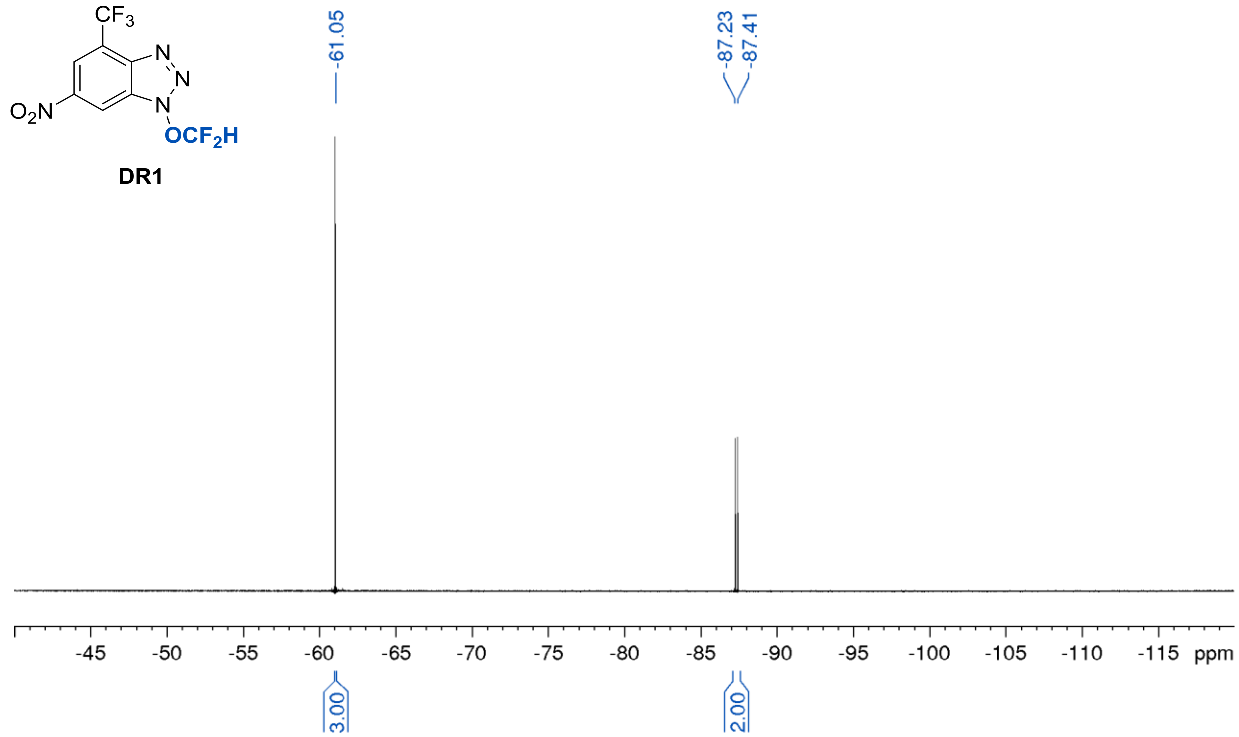

^{1}H NMR (700 MHz, $(CD_3)_2SO$, 25 °C) of 1a

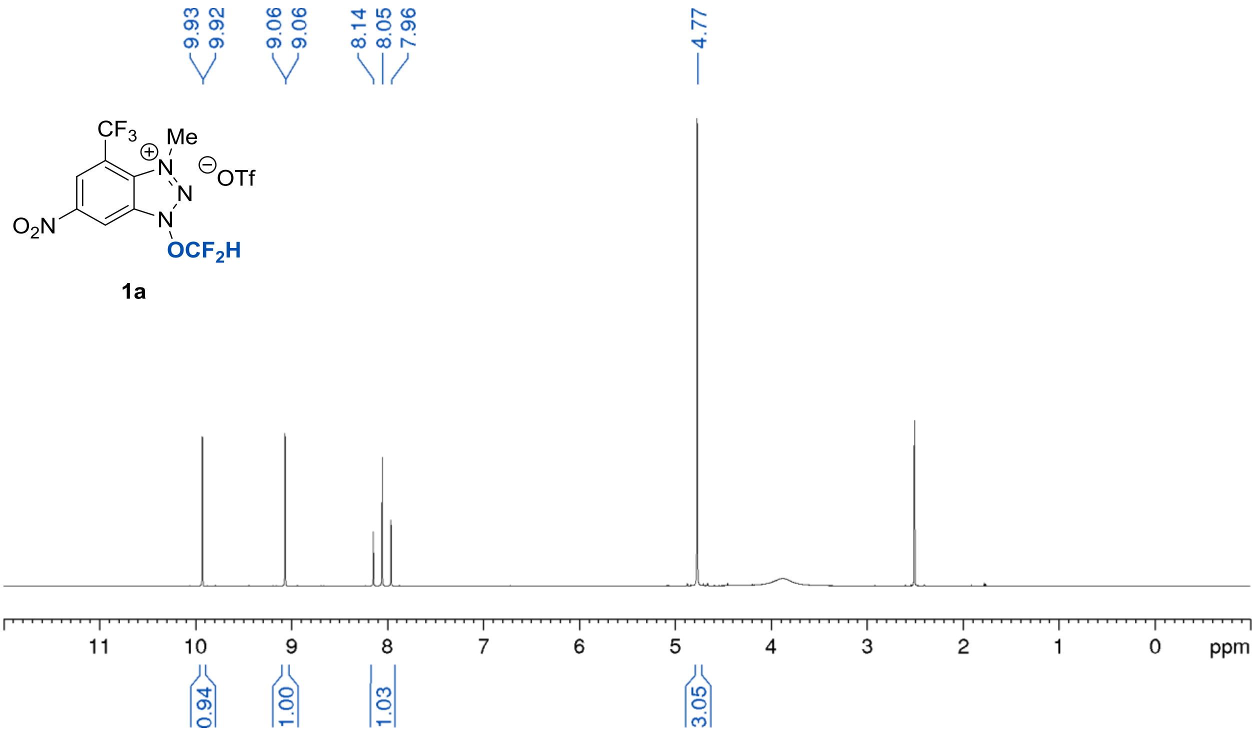

^{13}C NMR (175 MHz, $(CD_3)_2SO$, 25 °C) of 1a

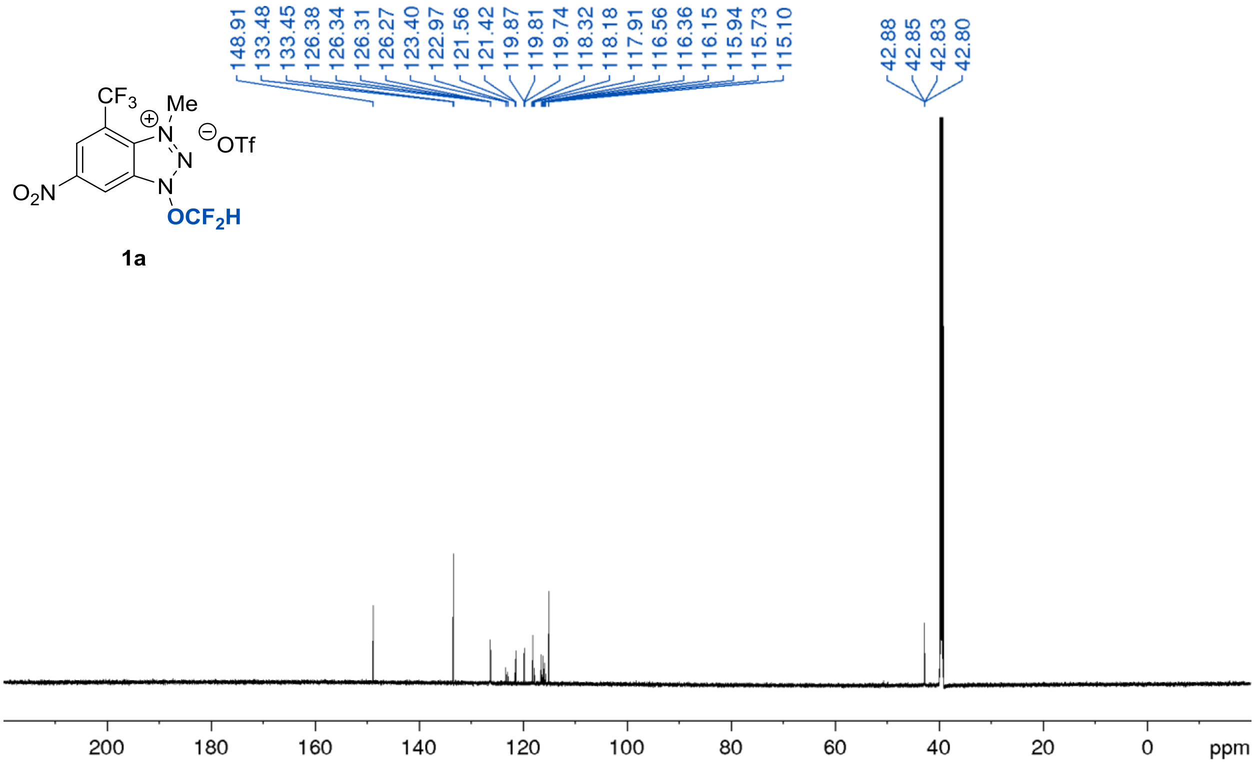

^{19}F NMR (376 MHz, $(CD_3)_2SO$, 25 ºC) of 1a

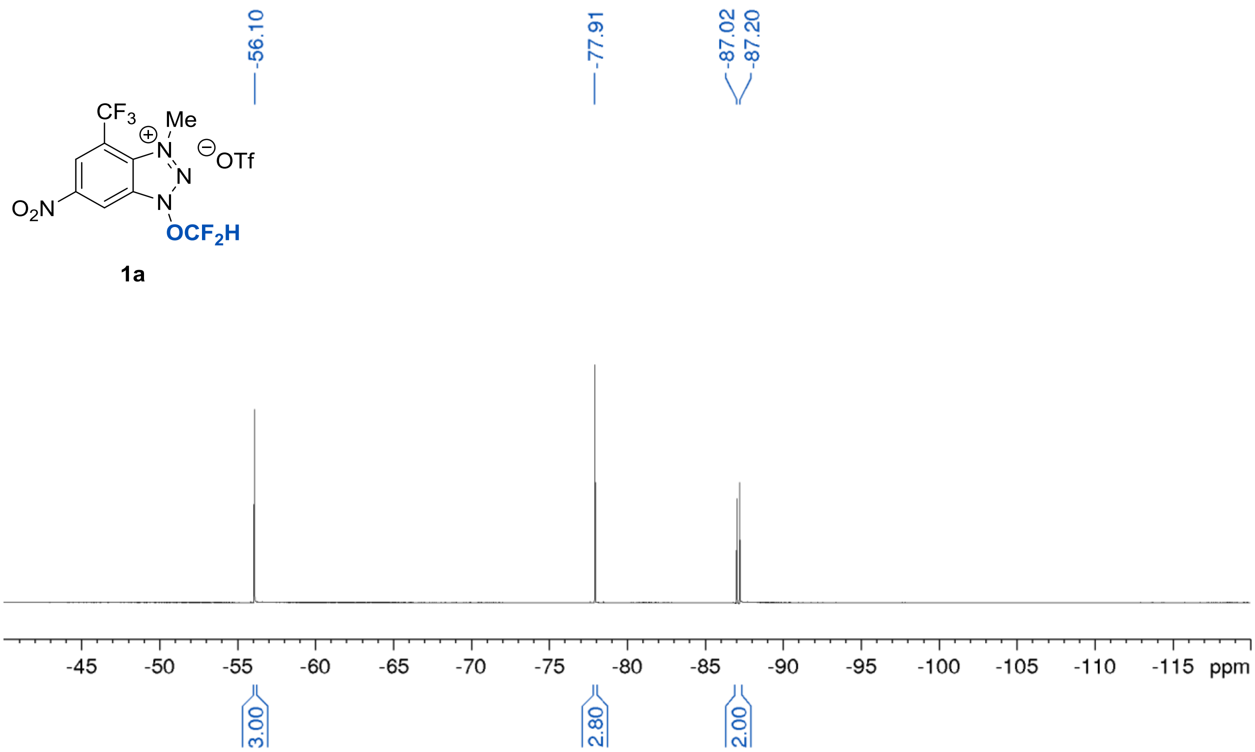

^{1}H NMR (700 MHz, $(CD_3)_2SO$, 25 ºC) of DR2

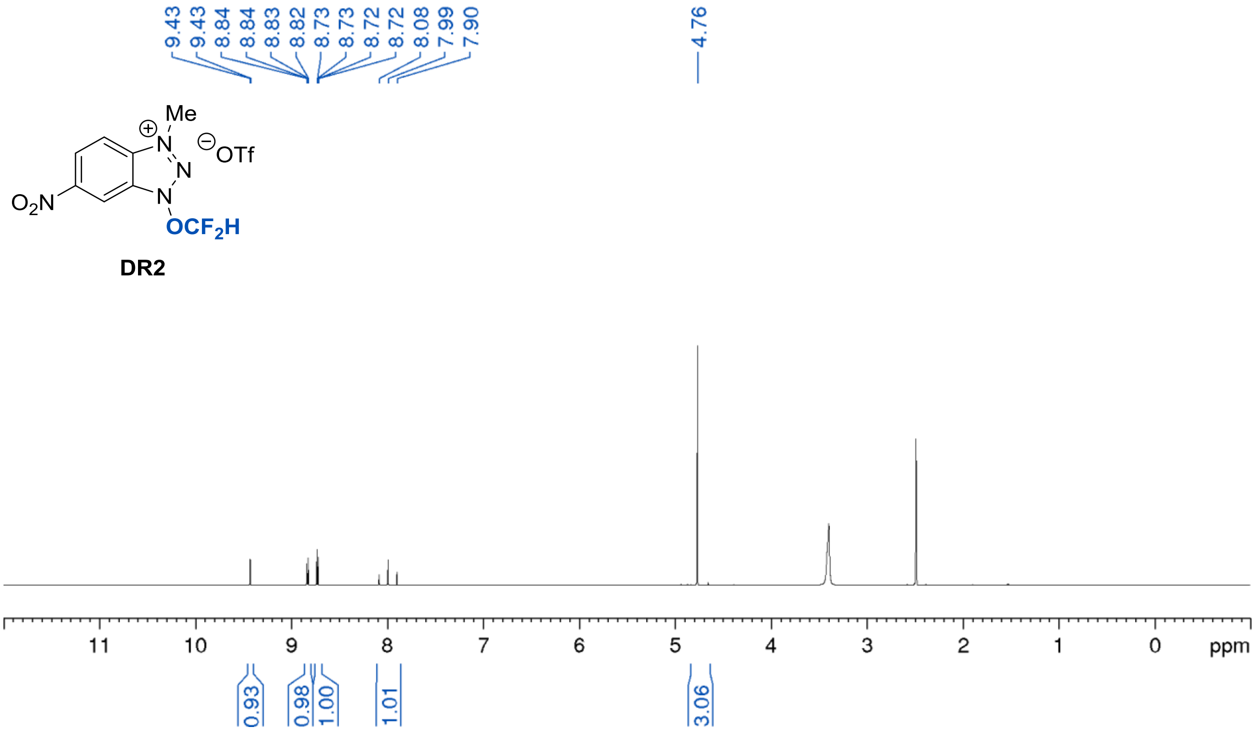

^{13}C NMR (175 MHz, $(CD_3)_2SO$, 25 ºC) of DR2

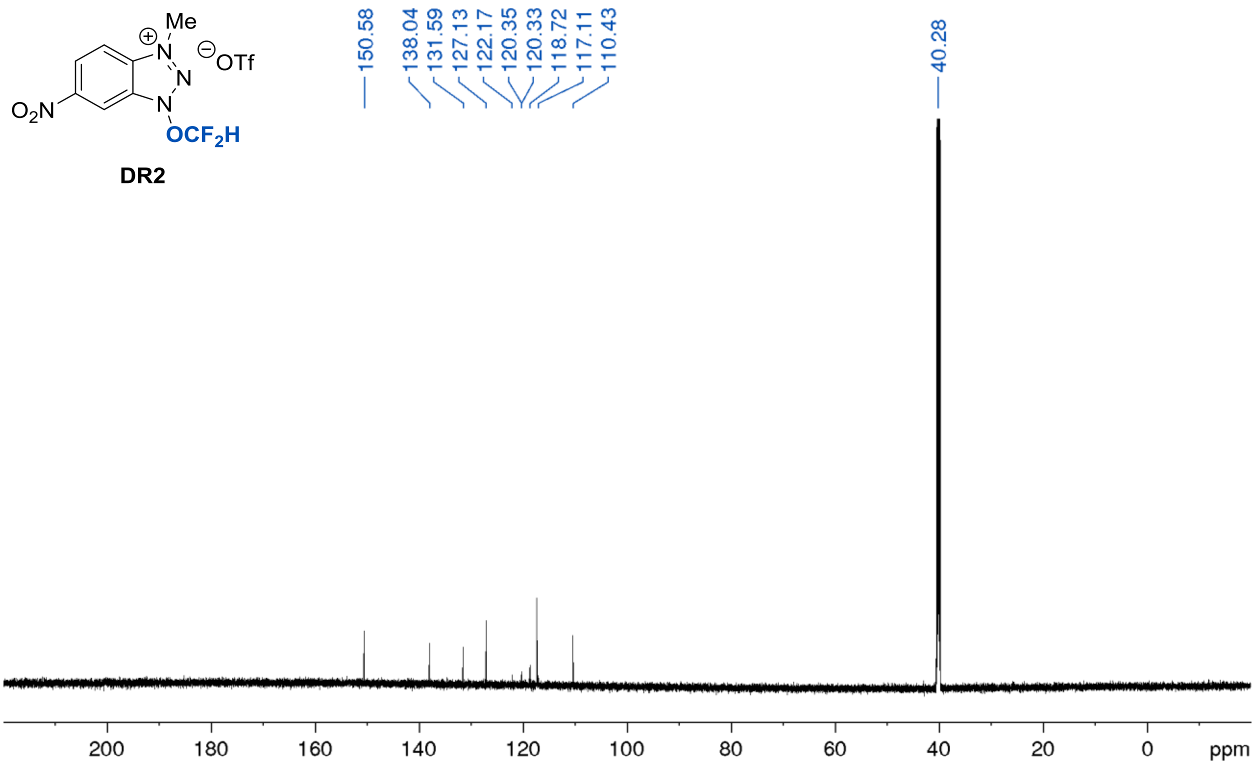

^{19}F NMR (376 MHz, $(CD_3)_2SO$, 25 ºC) of DR2

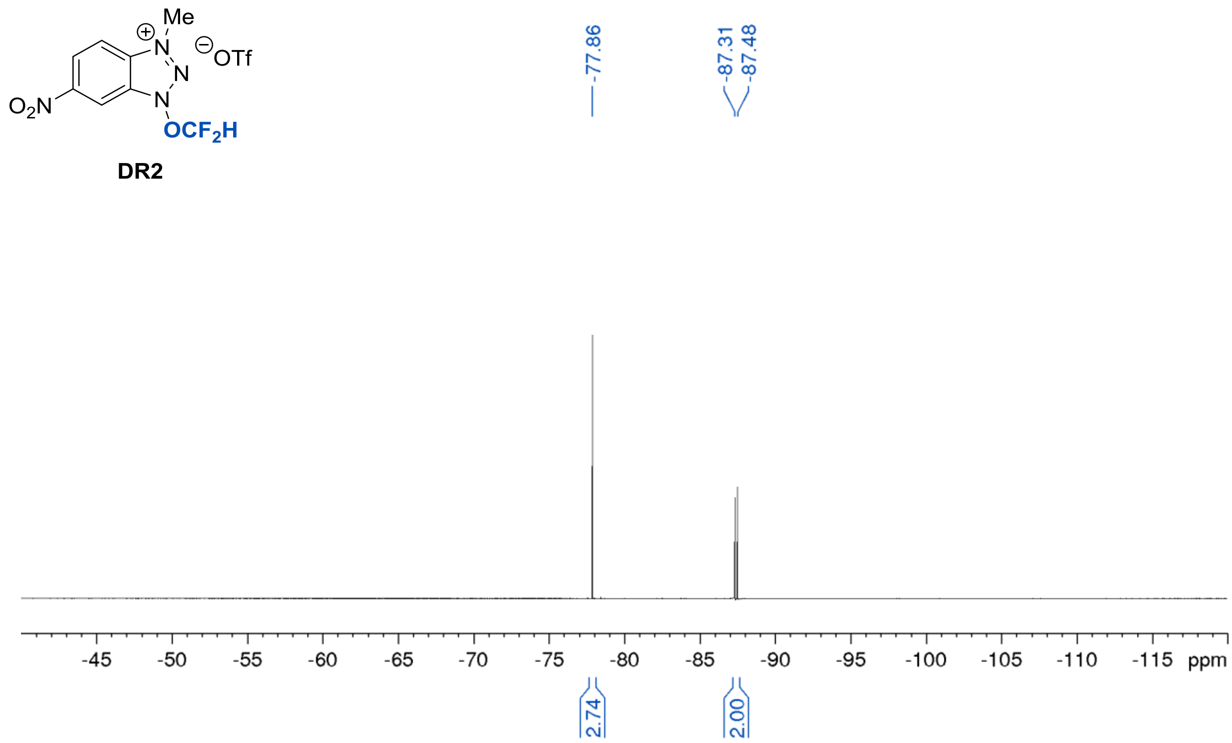

^{1}H NMR (700 MHz, $(CD_3)_2SO$, 25 °C) of DR3

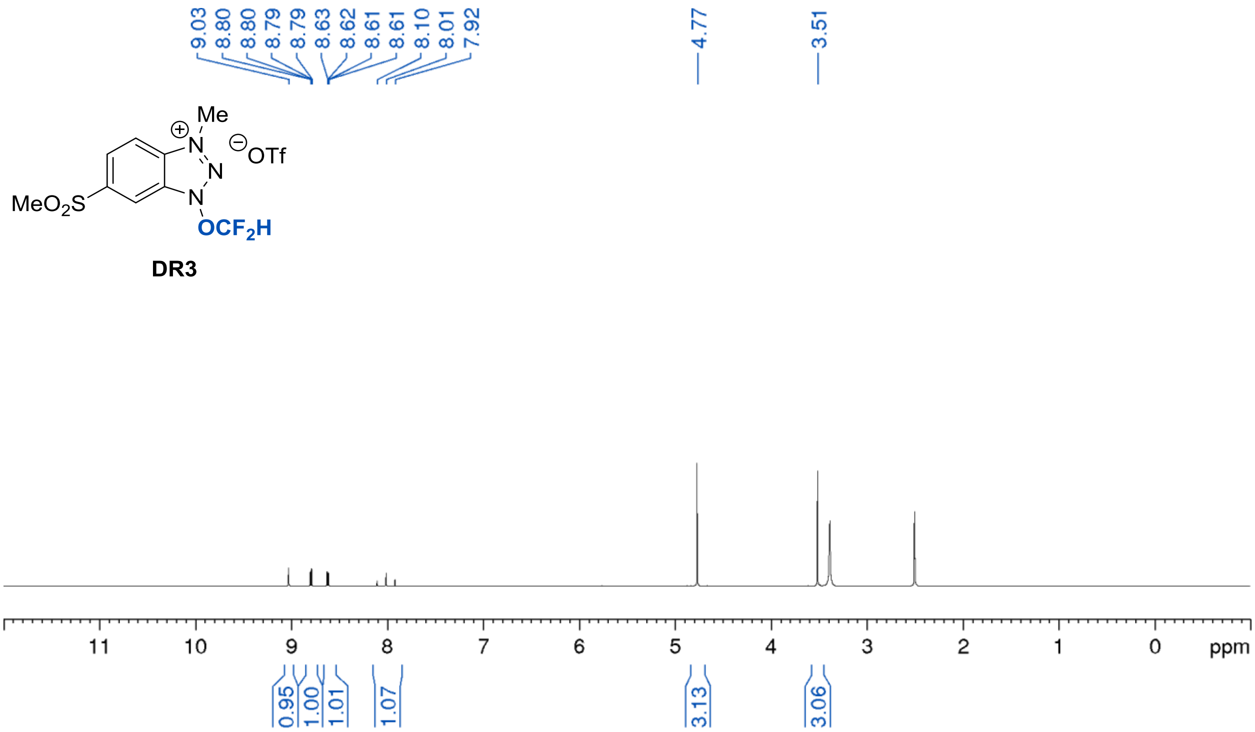

^{13}C NMR (175 MHz, $(CD_3)_2SO$, 25 °C) of DR3

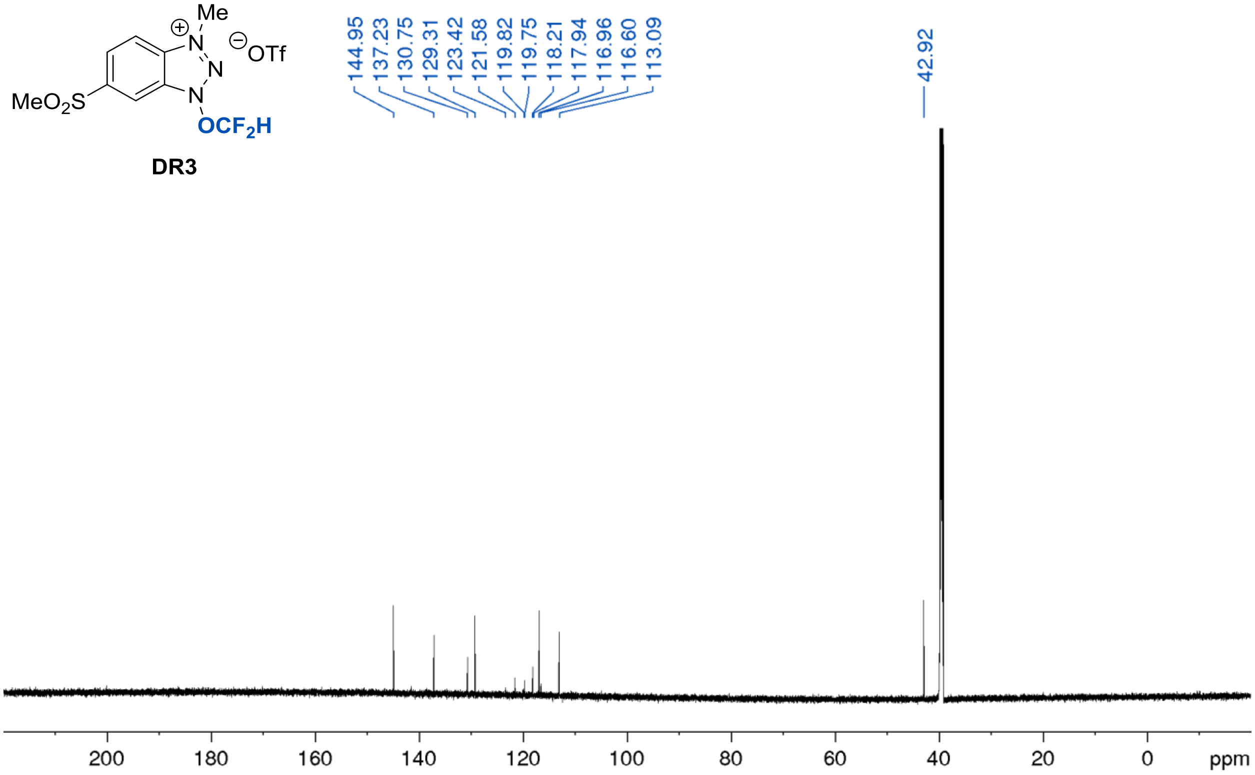

^{19}F NMR (376 MHz, $(CD_3)_2SO$, 25 °C) of DR3

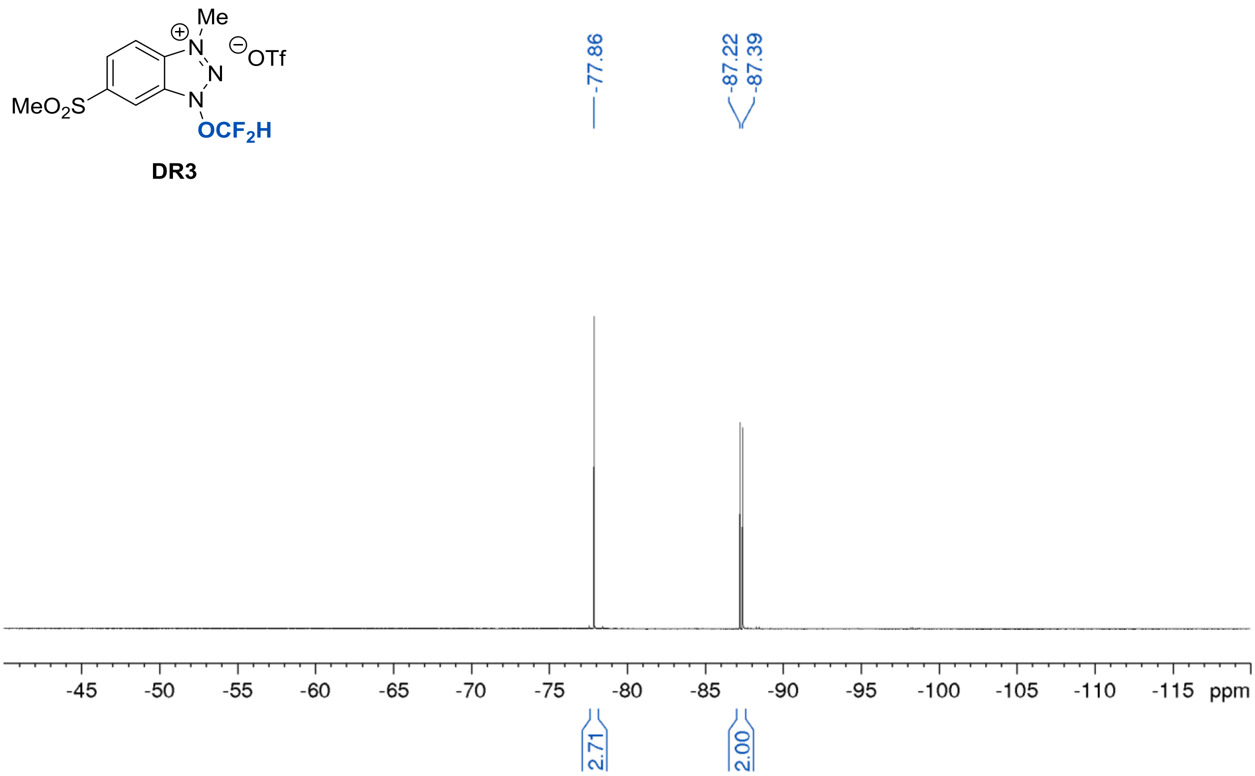

^{1}H NMR (700 MHz, $(CD_3)_2SO$, 25 °C) of DR4

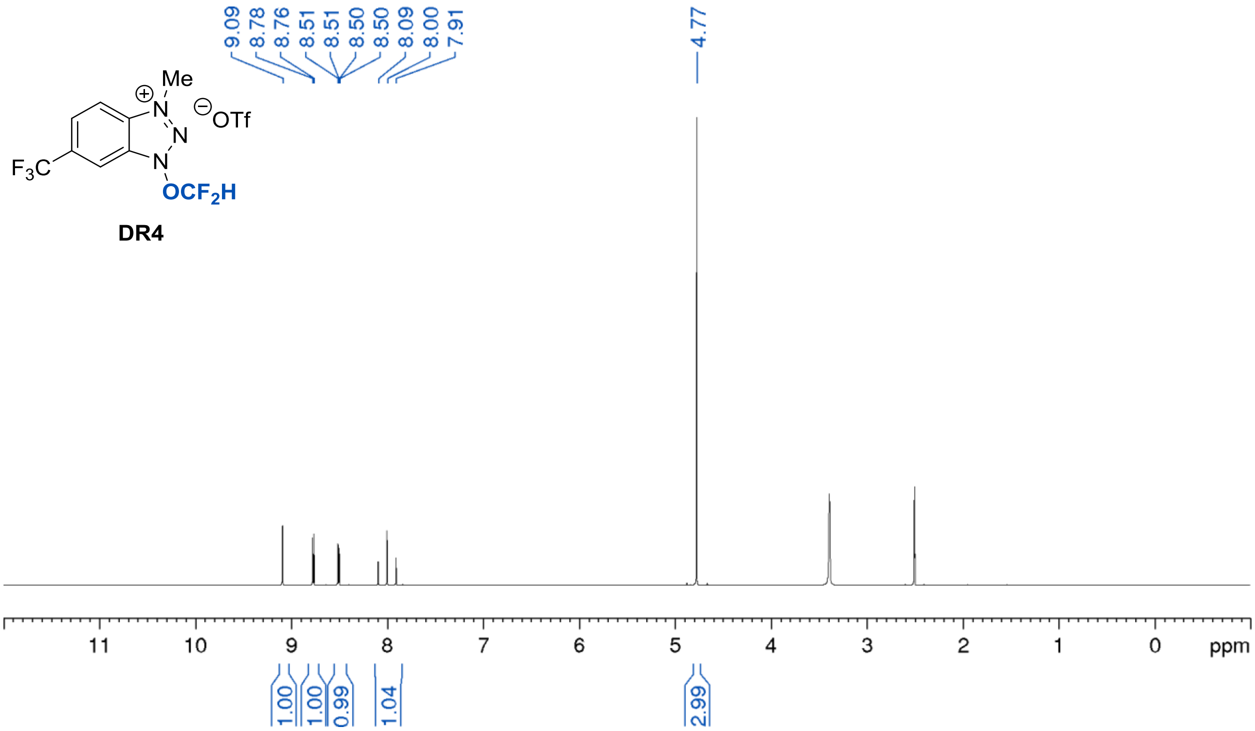

^{13}C NMR (175 MHz, $(CD_3)_2SO$, 25 ºC) of DR4

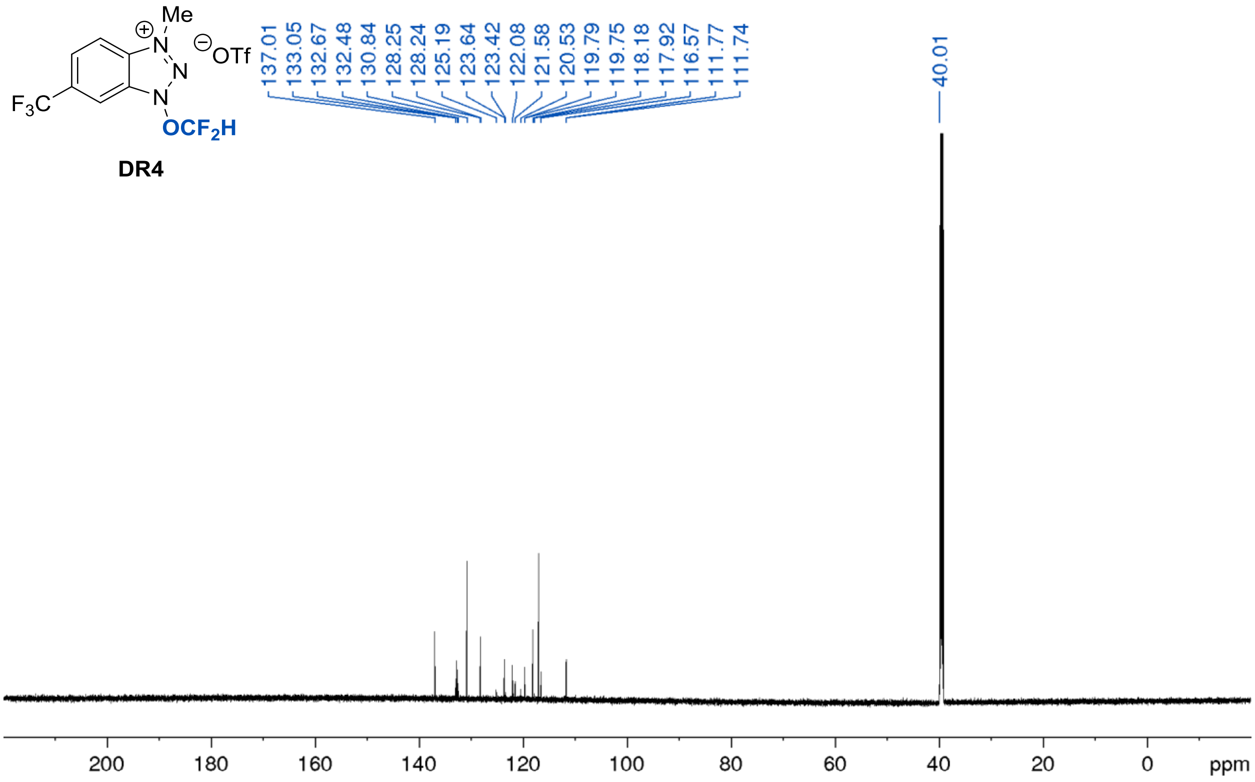

^{19}F NMR (376 MHz, $(CD_3)_2SO$, 25 ºC) of DR4

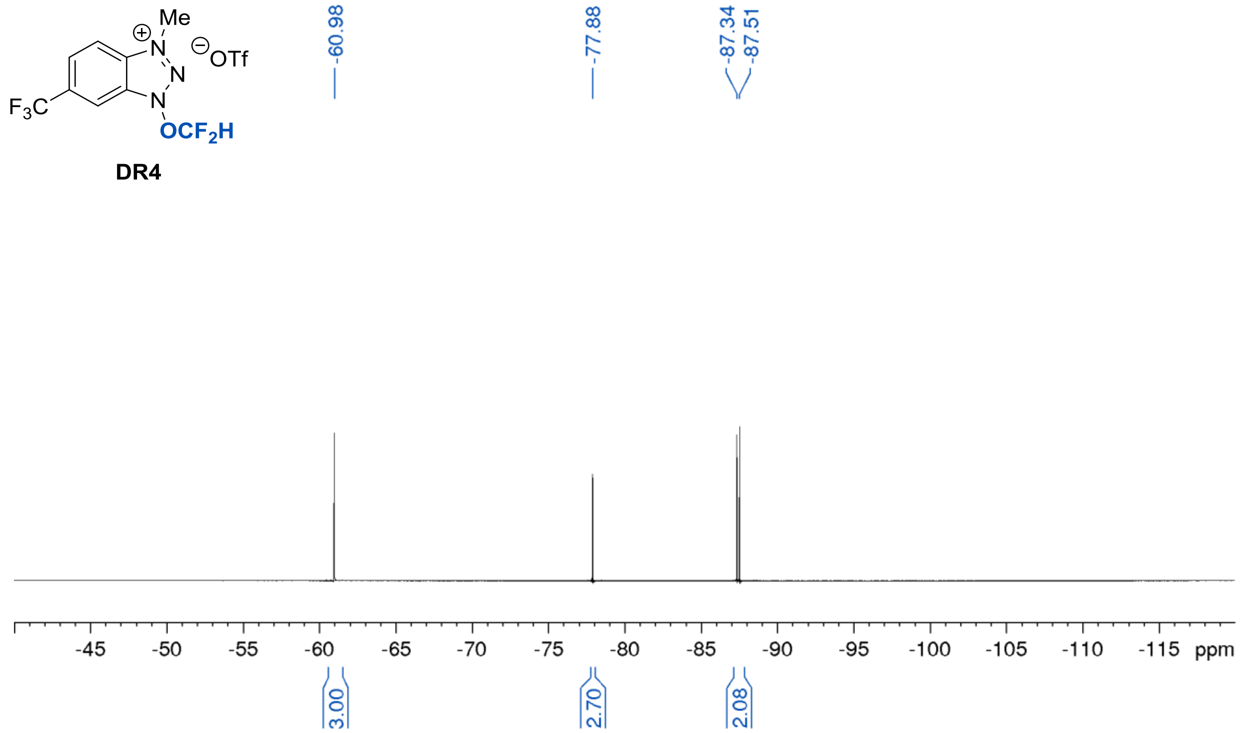

^{1}H NMR (700 MHz, $(CD_3)_2SO$, 25 °C) of DR4

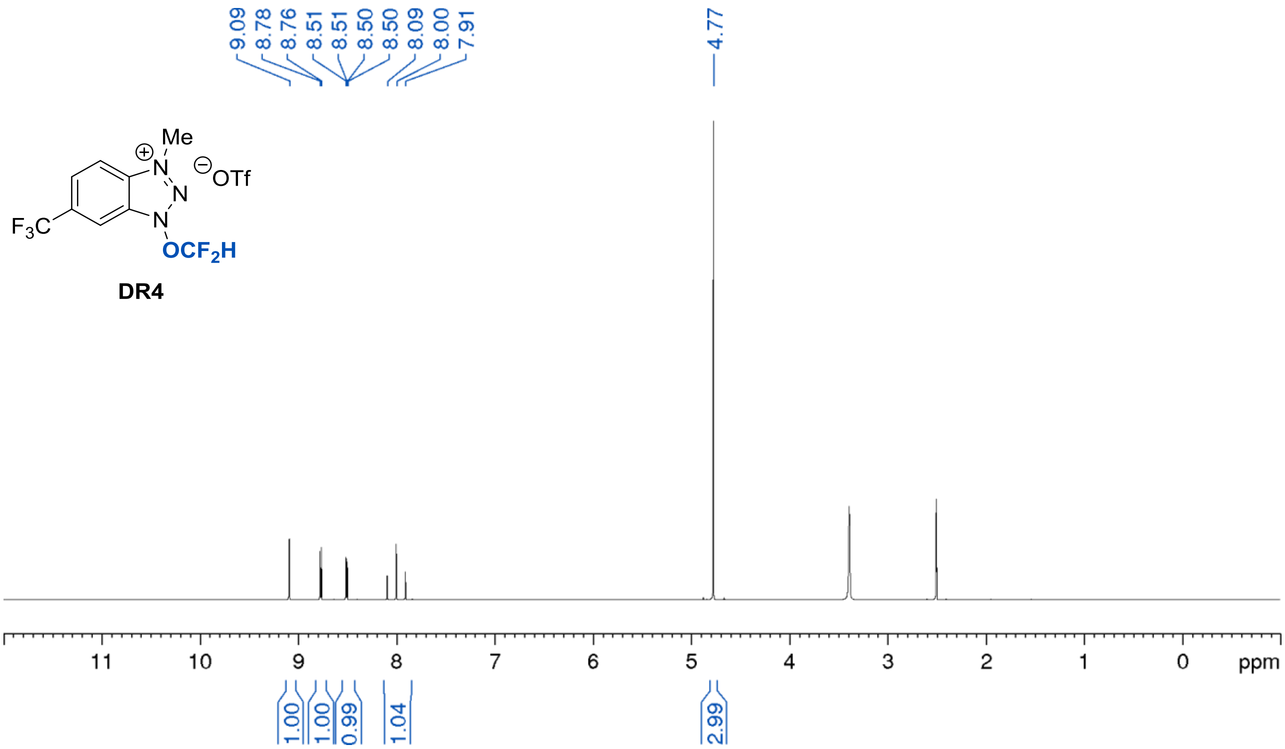

DR4

^{1}H NMR (700 MHz, $(CD_3)_2SO$, 25 °C) of DR5

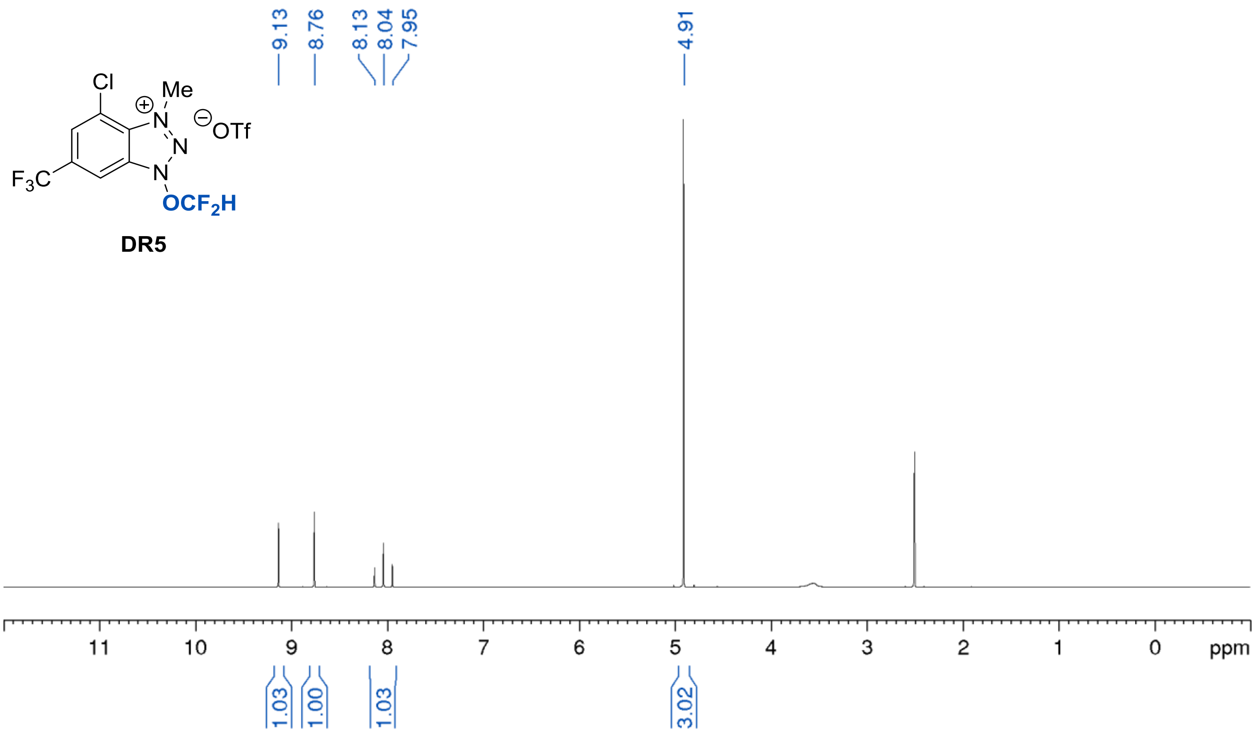

DR5

^{13}C NMR (175 MHz, $(CD_3)_2SO$, 25 ºC) of DR5

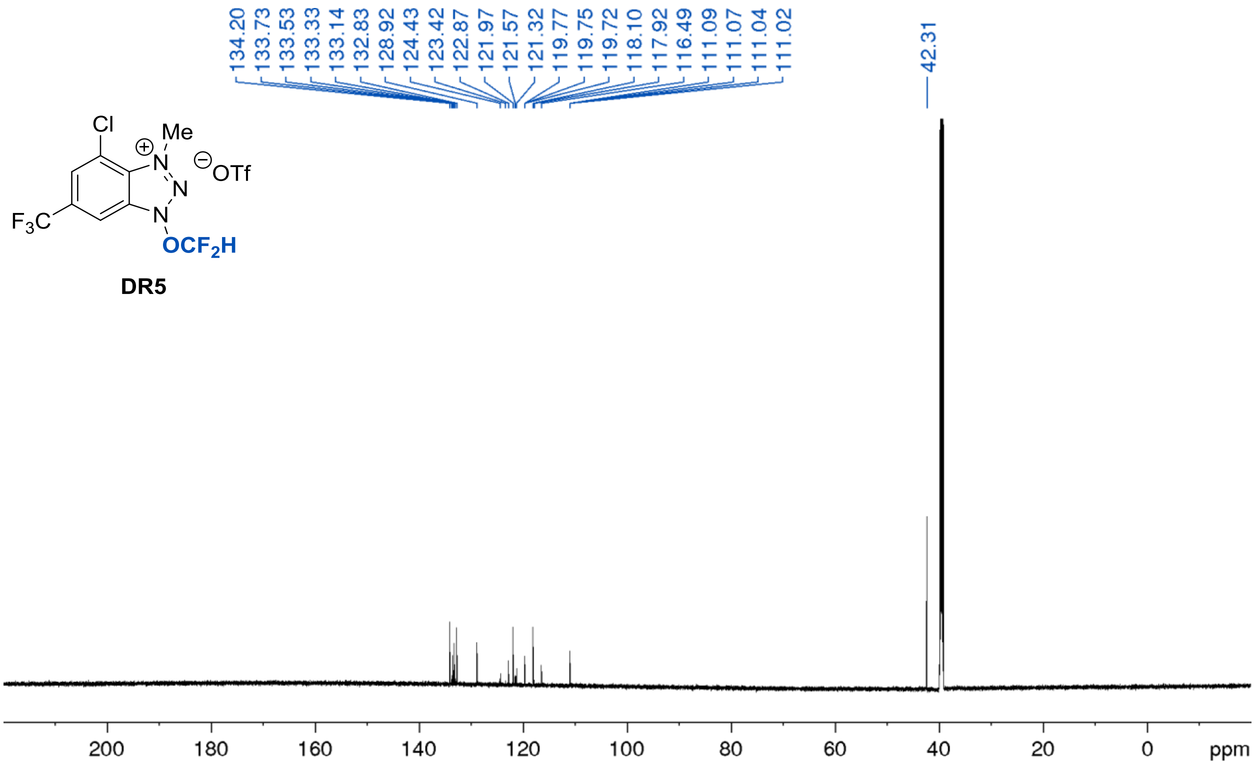

^{19}F NMR (376 MHz, $(CD_3)_2SO$, 25 ºC) of DR5

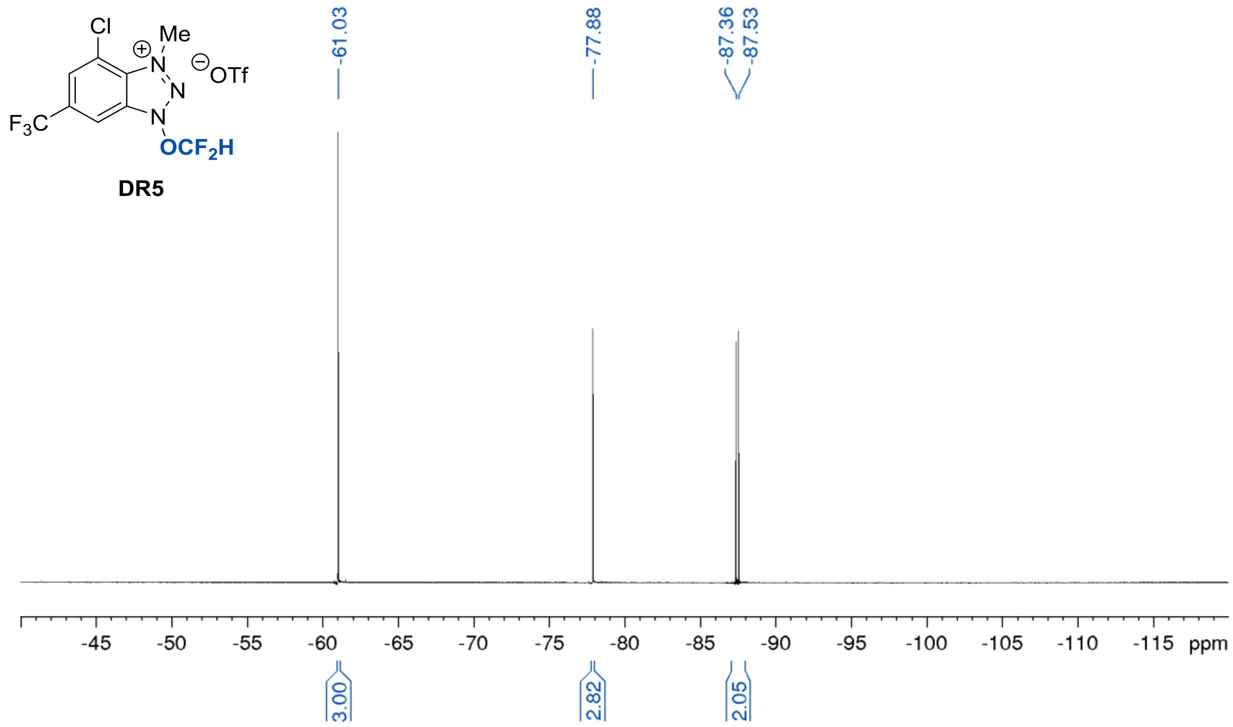

^{1}H NMR (700 MHz, CDCl$_3$, 25 °C) of 3b-*ortho*

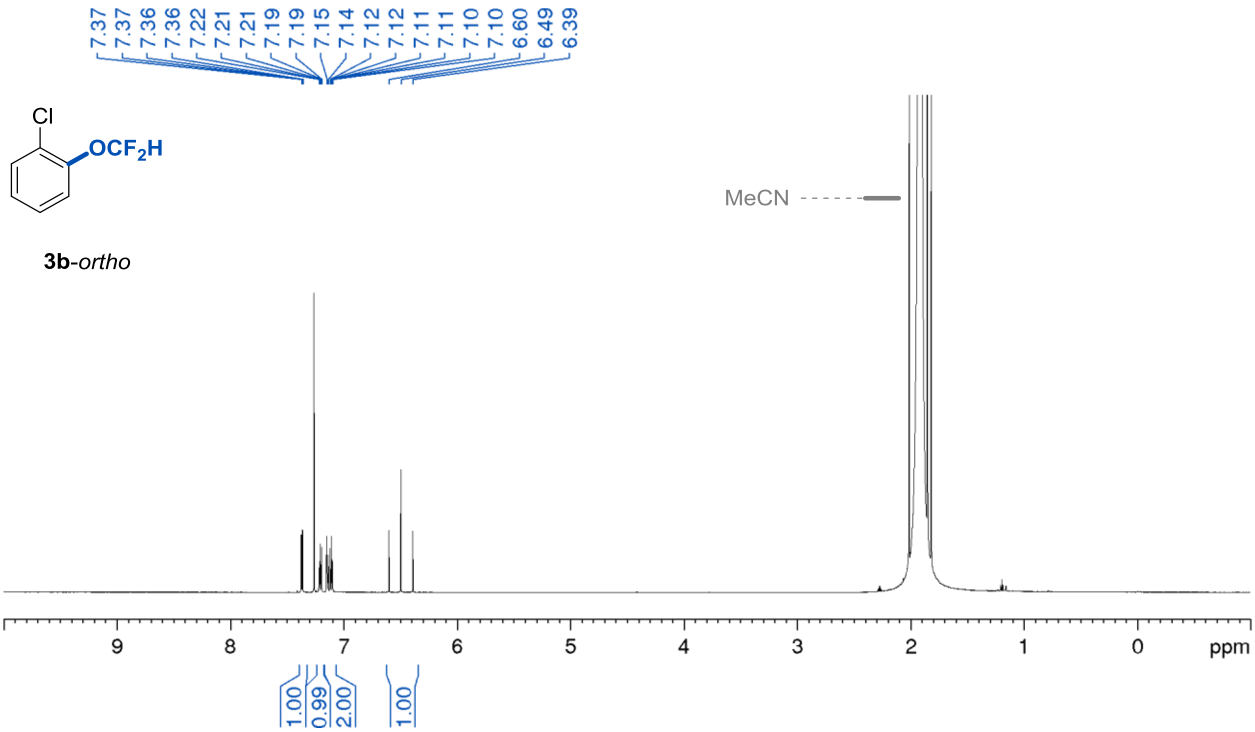

^{13}C NMR (175 MHz, CDCl$_3$, 25 °C) of 3b-*ortho*

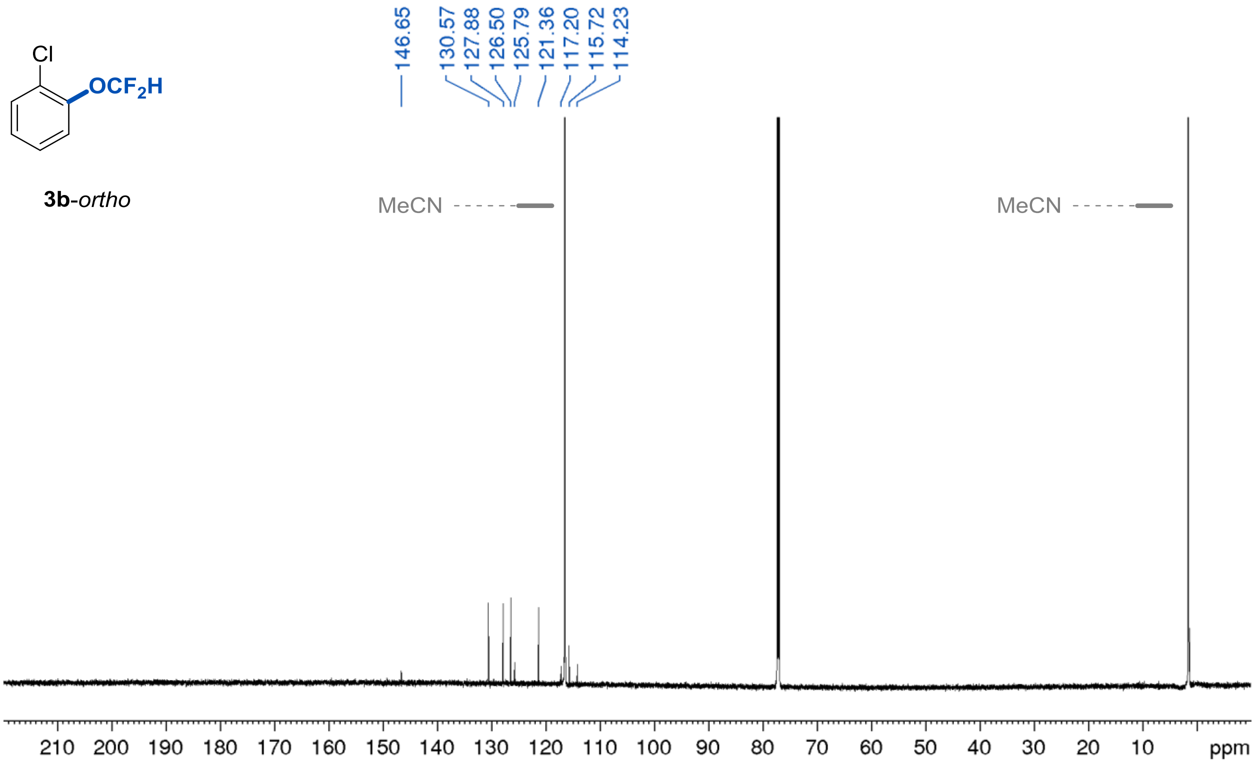

^{19}F NMR (376 MHz, $CDCl_3$, 25 °C) of 3b-*ortho*

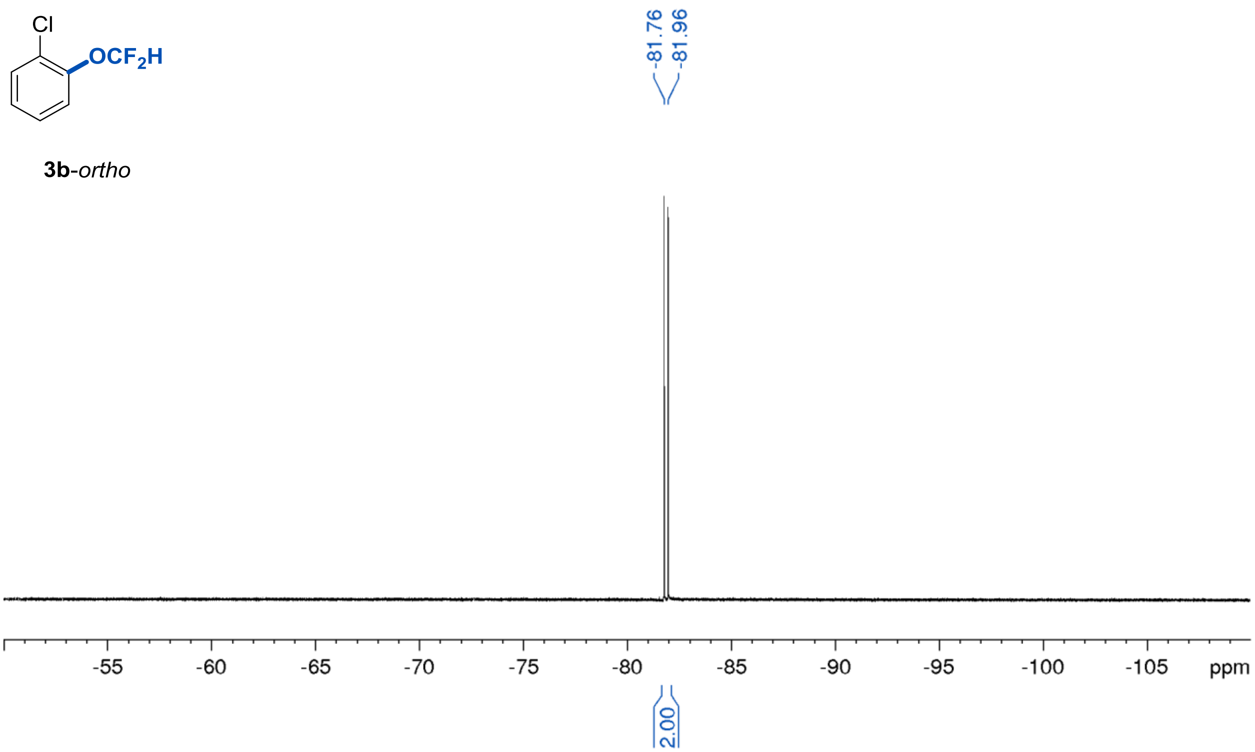

^{1}H NMR (700 MHz, $CDCl_3$, 25 °C) of 3b-*meta* and -*para*

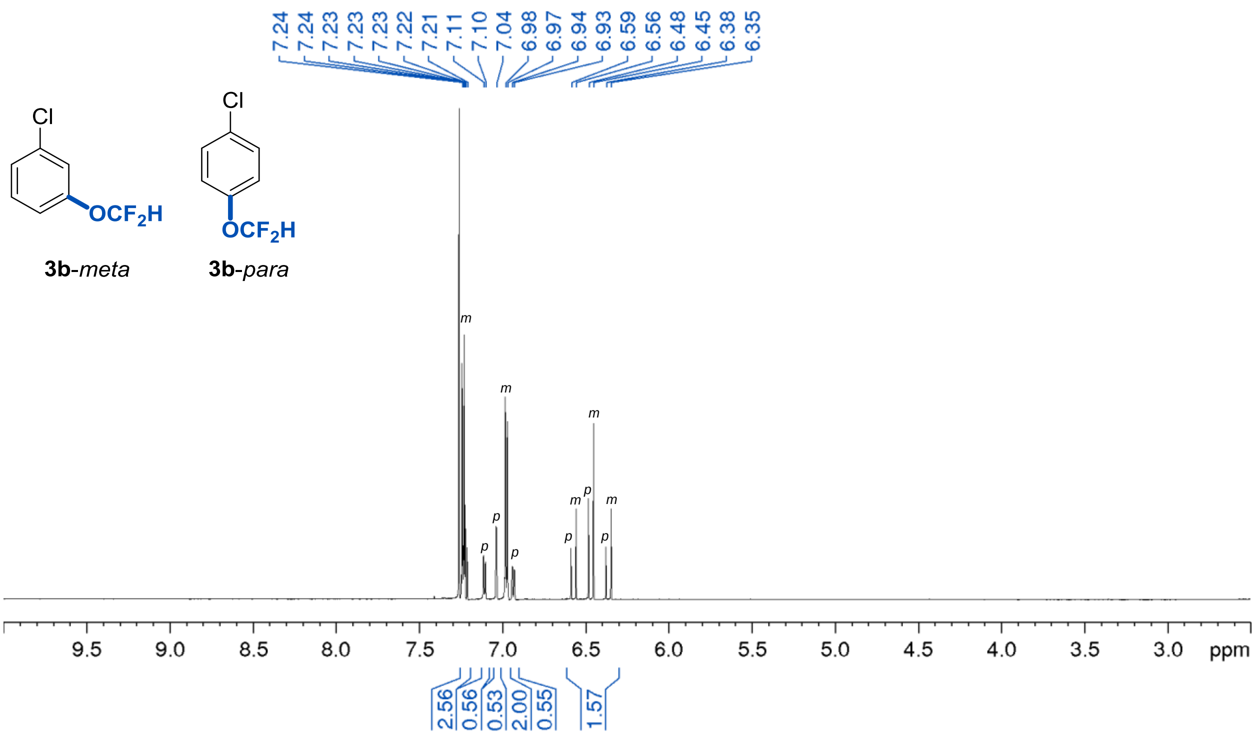

^{13}C NMR (175 MHz, $CDCl_3$, 25 ºC) of 3b-*meta* and -*para*

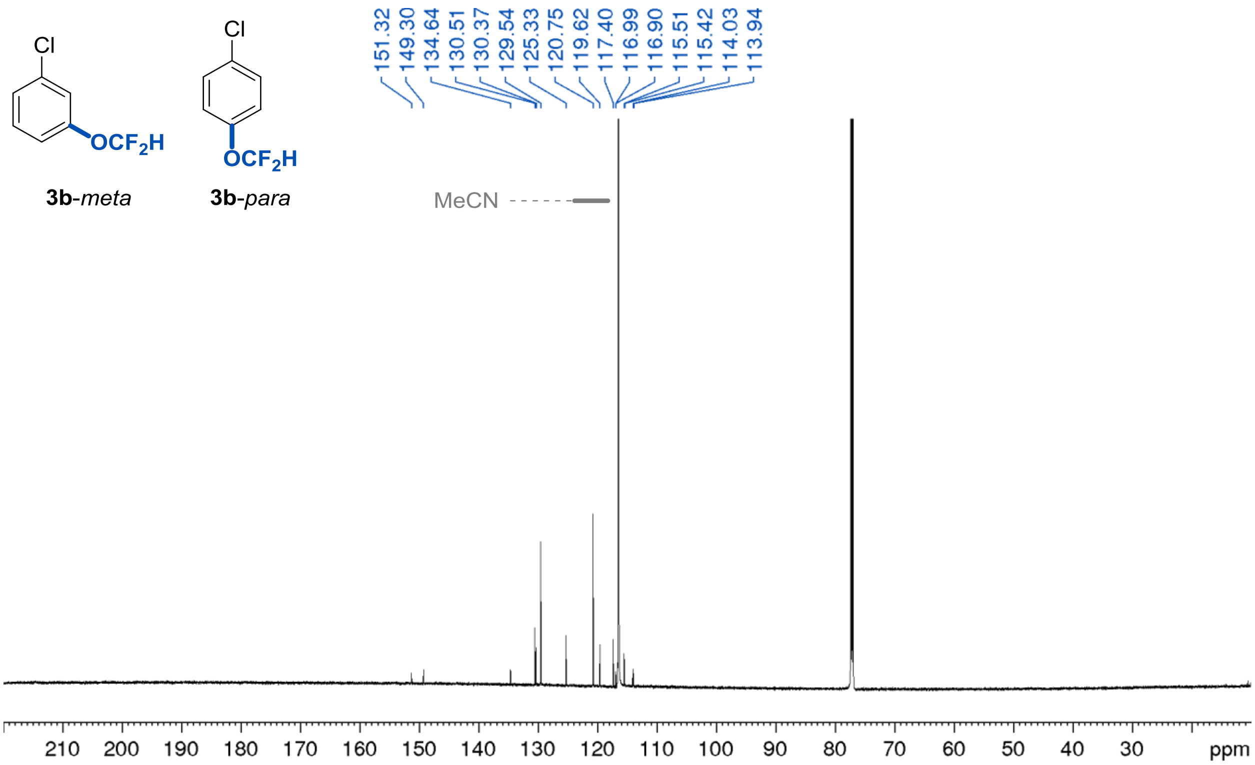

^{19}F NMR (376 MHz, $CDCl_3$, 25 ºC) of 3b-*meta* and -*para*

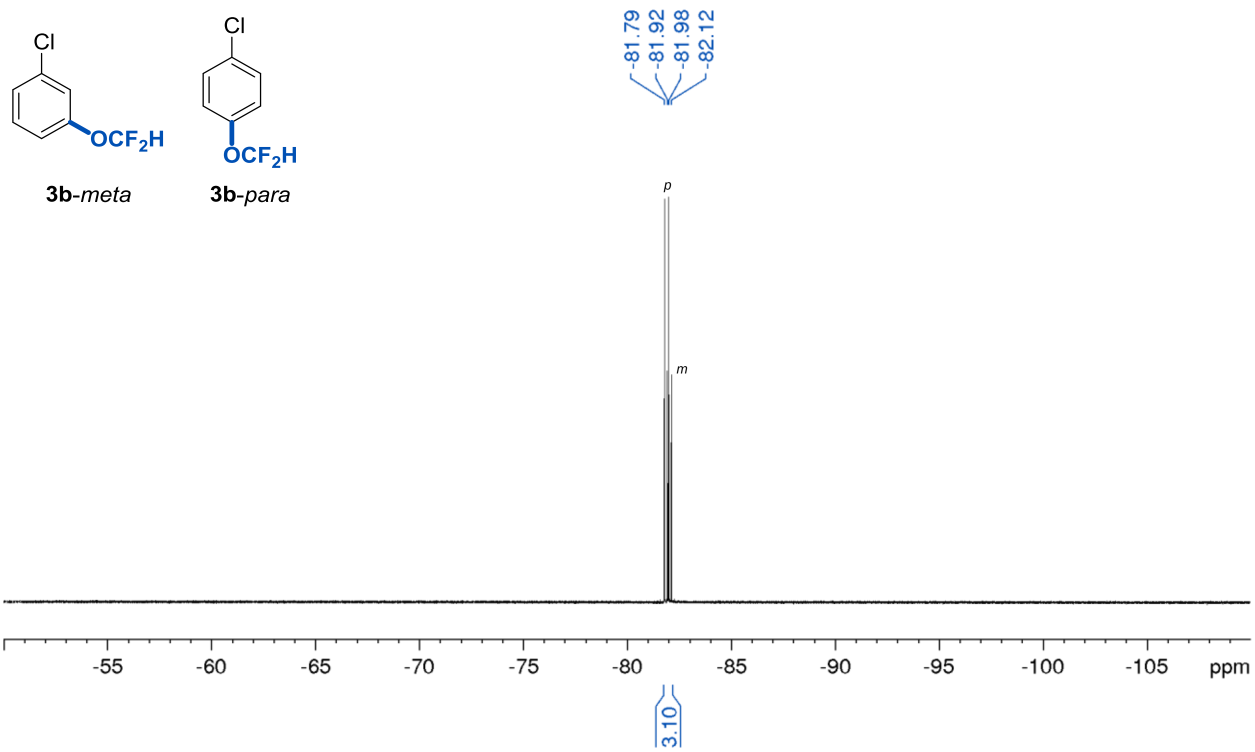

^{1}H NMR (700 MHz, CDCl$_3$, 25 °C) of 3c

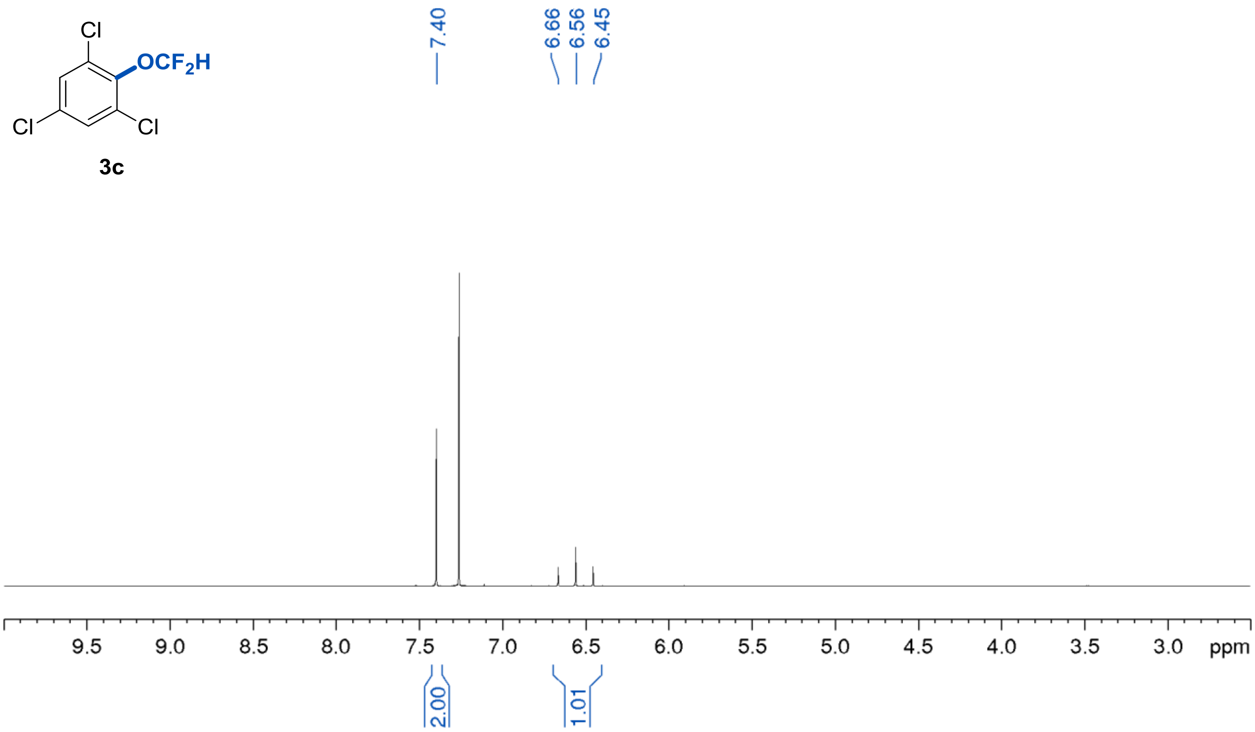

^{13}C NMR (175 MHz, CDCl$_3$, 25 °C) of 3c

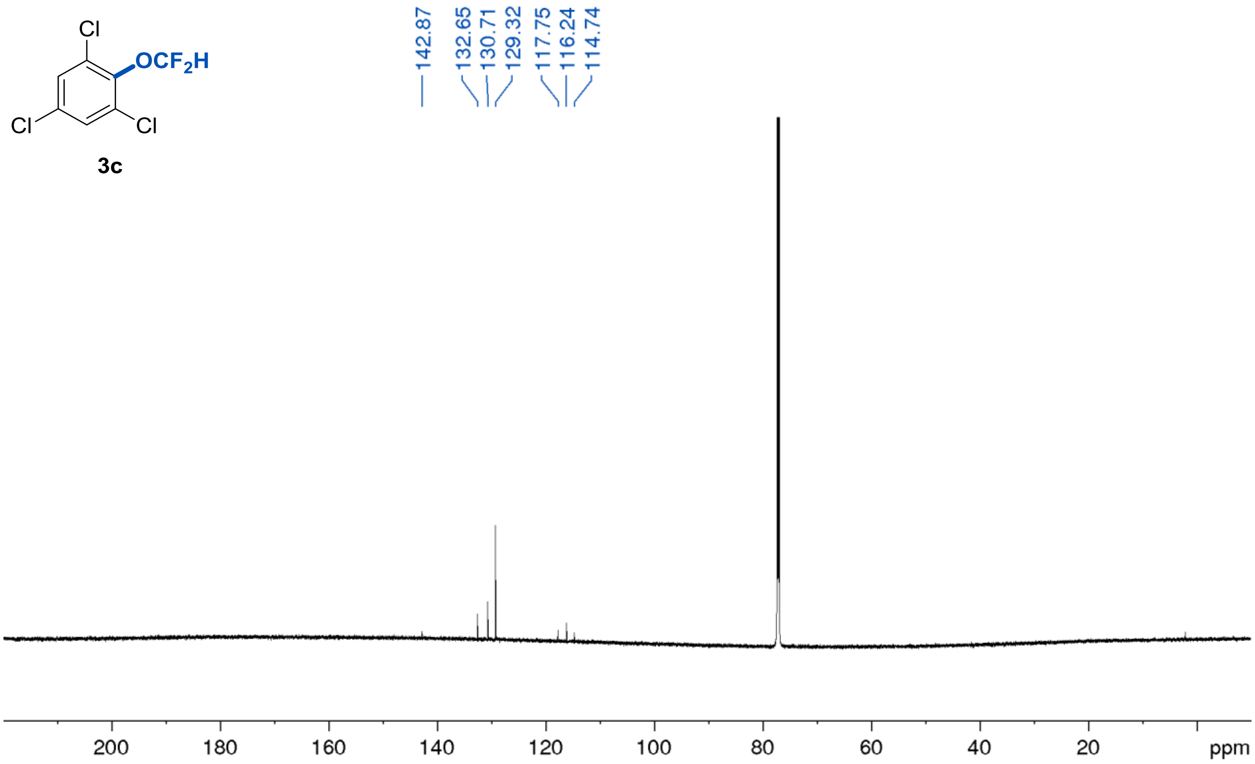

^{19}F NMR (376 MHz, $CDCl_3$, 25 °C) of 3c

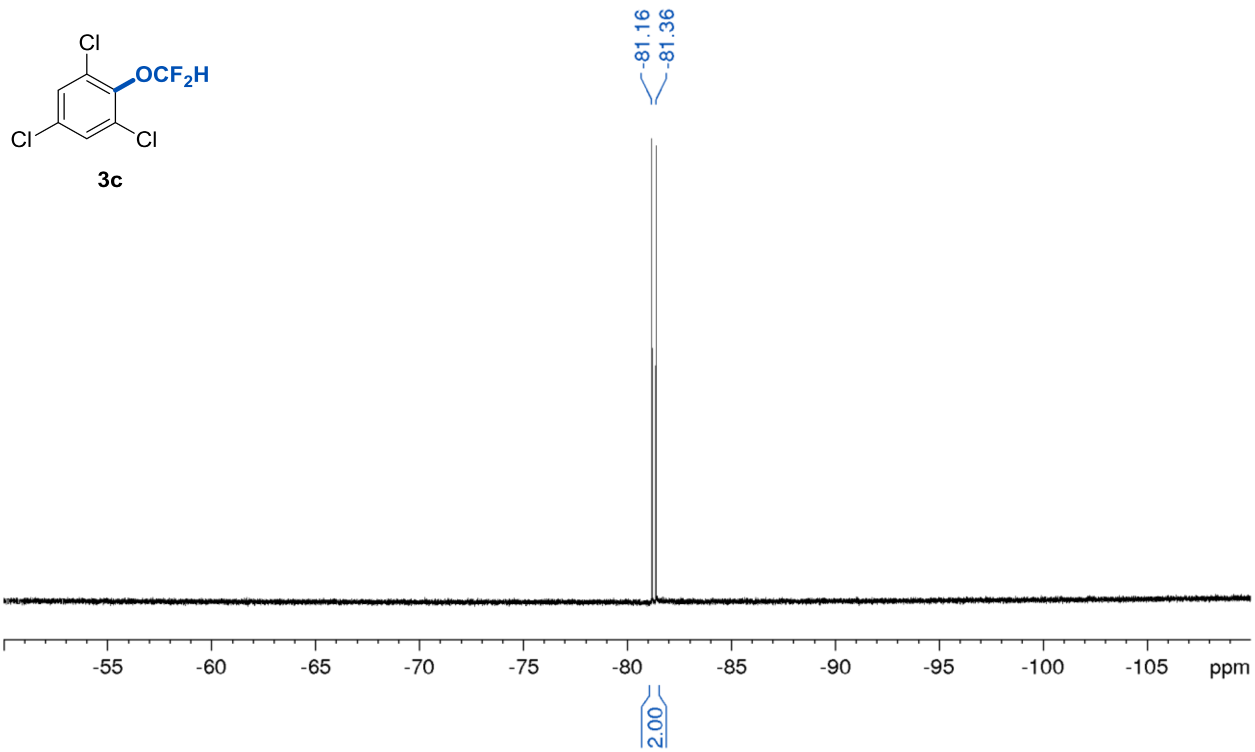

^{1}H NMR (700 MHz, $CDCl_3$, 25 °C) of 3d

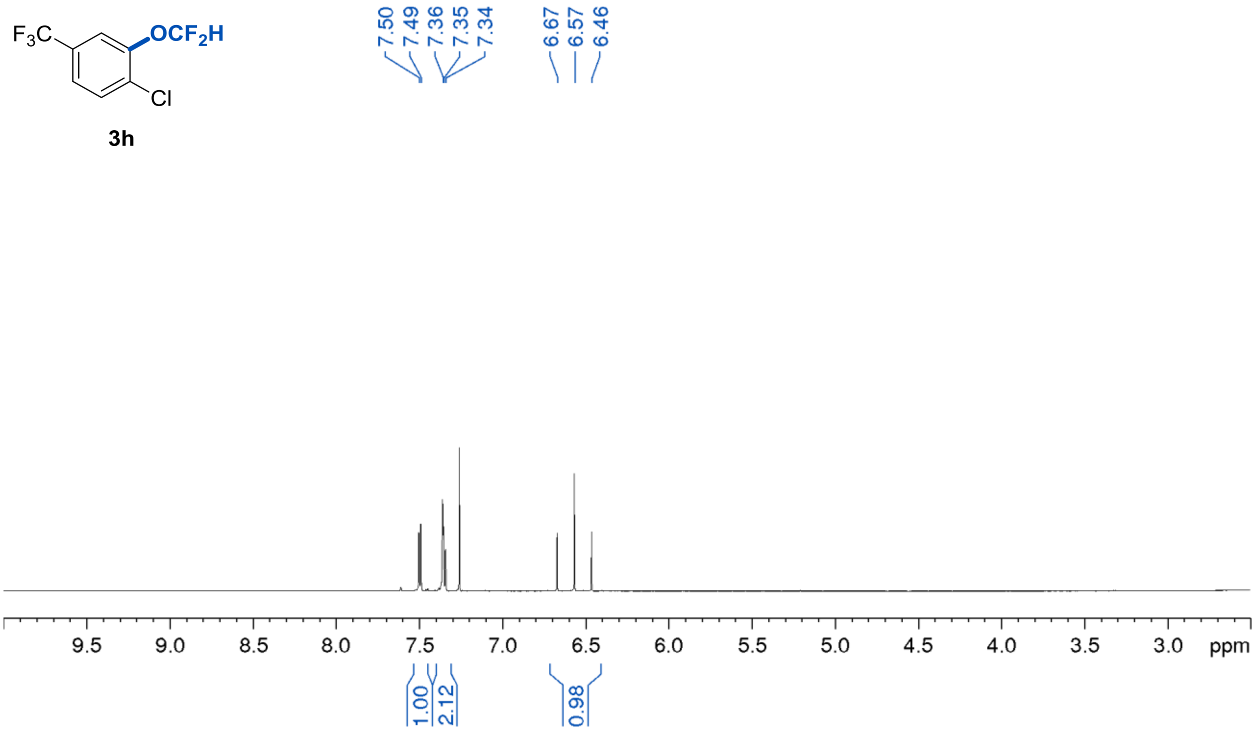

^{13}C NMR (175 MHz, $CDCl_3$, 25 ºC) of 3d

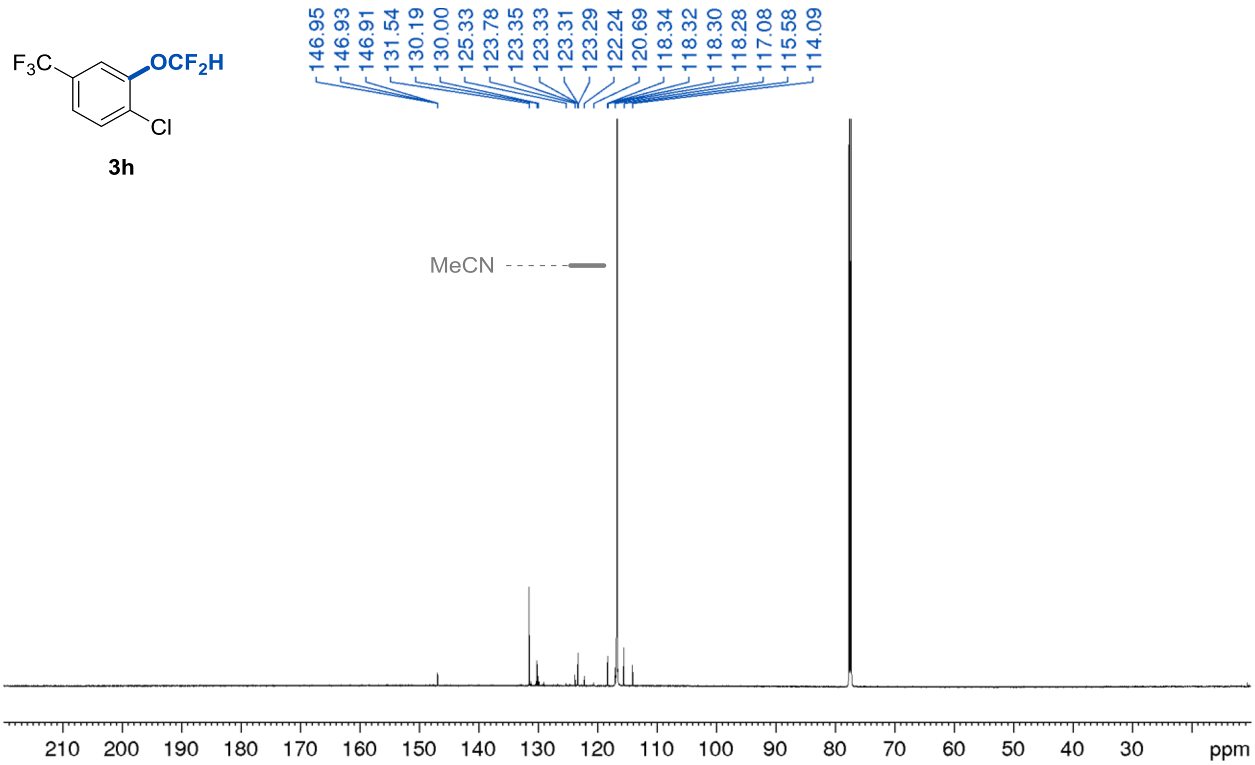

^{19}F NMR (376 MHz, $CDCl_3$, 25 ºC) of 3d

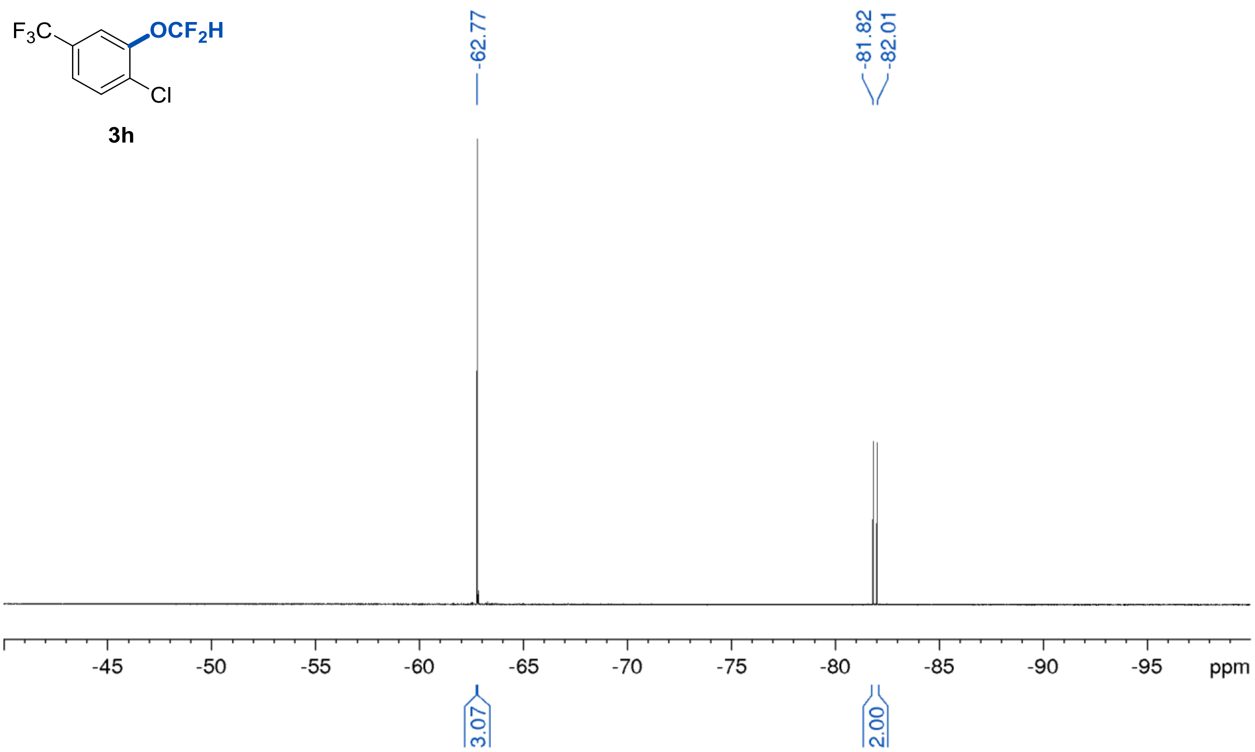

^{1}H NMR (700 MHz, $CDCl_3$, 25 °C) of 3e-*ortho*

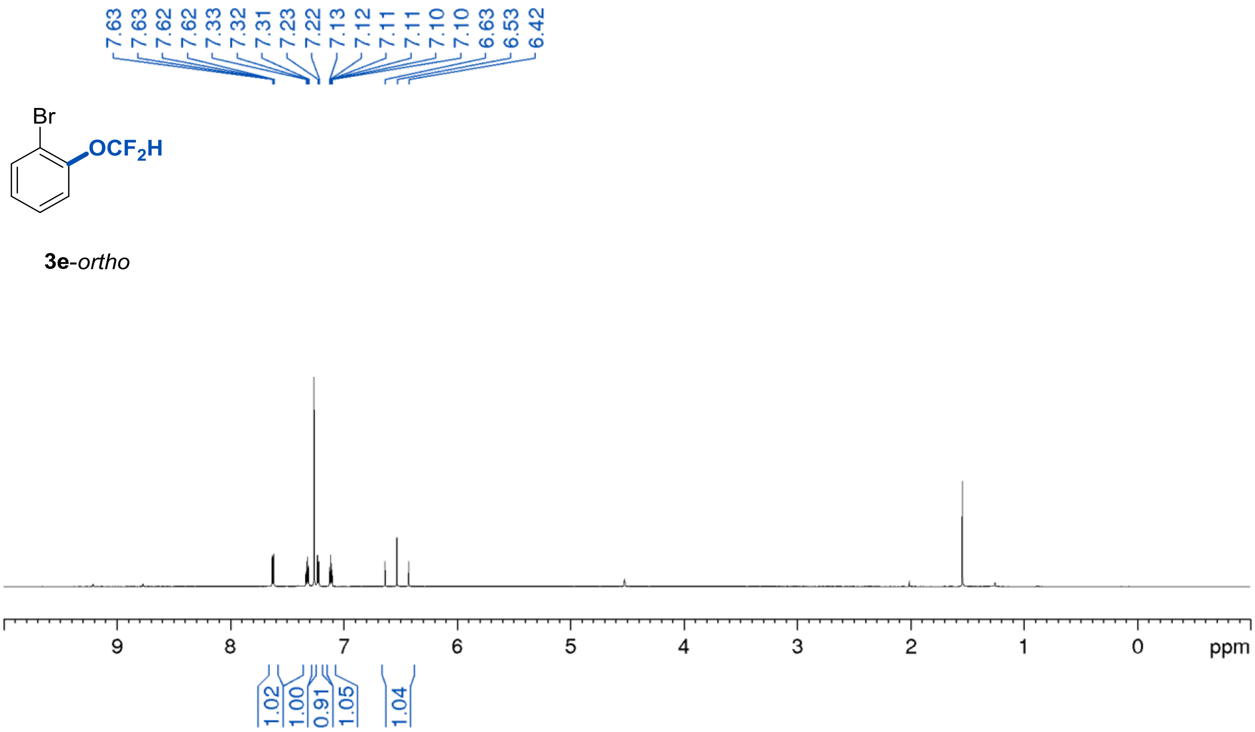

^{13}C NMR (175 MHz, $CDCl_3$, 25 °C) of 3e-*ortho*

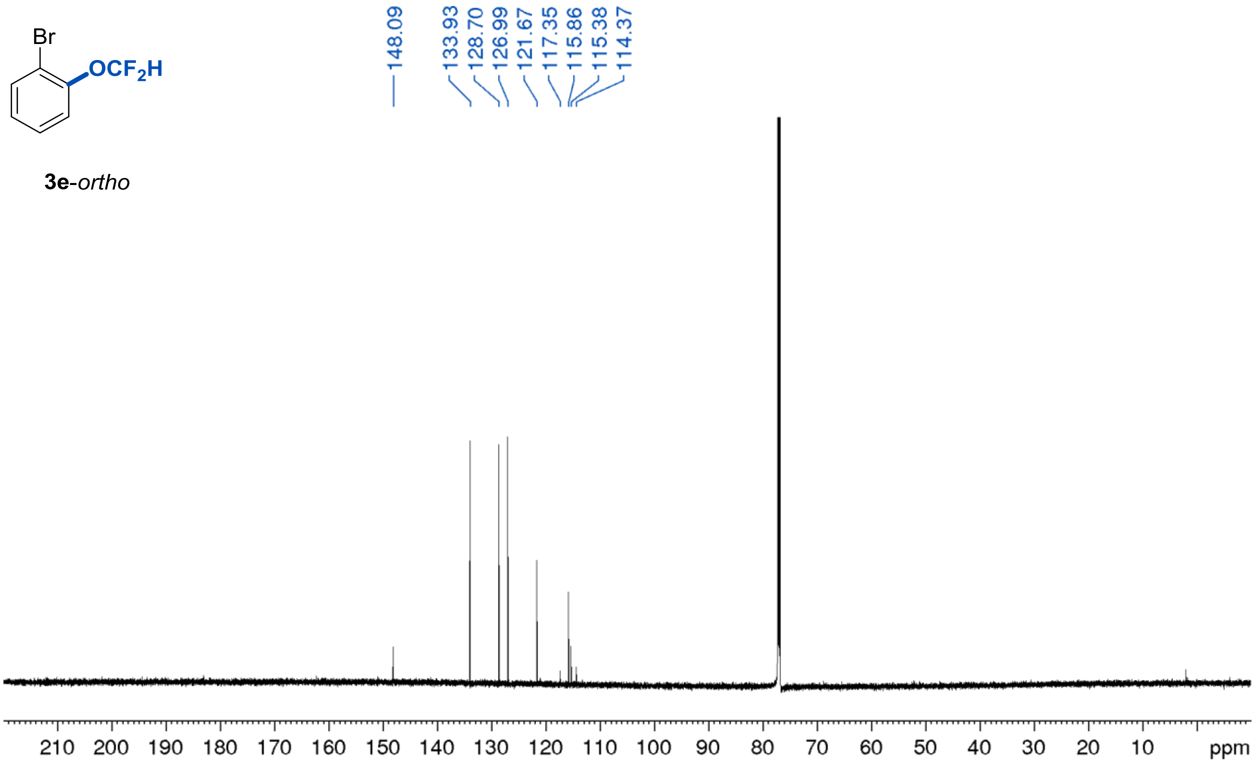

^{19}F NMR (376 MHz, $CDCl_3$, 25 °C) of 3e-*ortho*

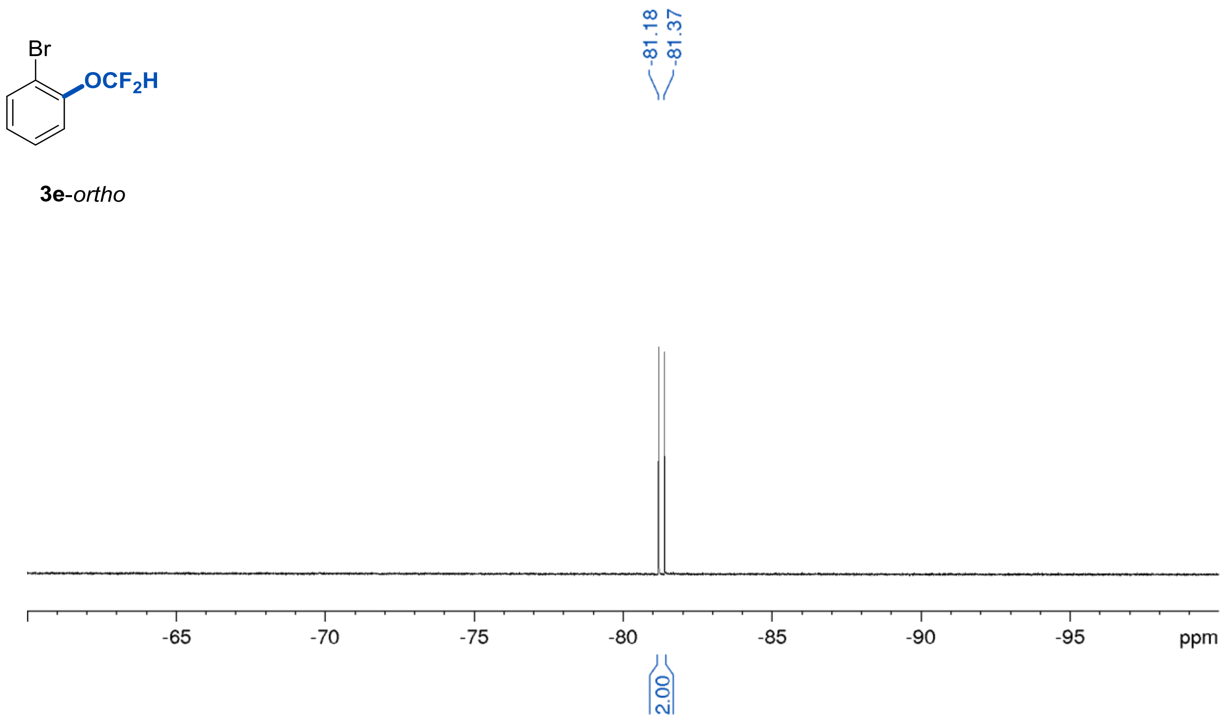

^{1}H NMR (700 MHz, $CDCl_3$, 25 °C) of 3e-*meta* and -*para*

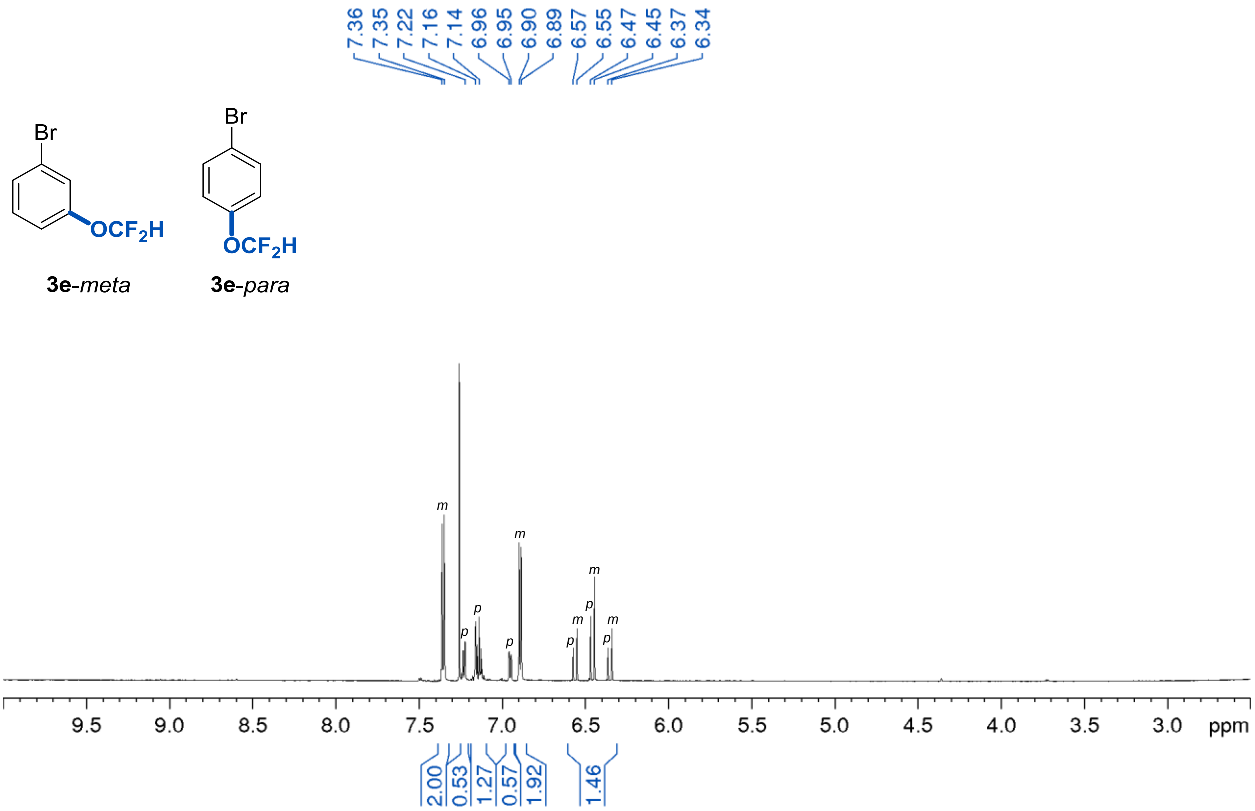

^{13}C NMR (175 MHz, $CDCl_3$, 25 ºC) of 3e-*meta* and -*para*

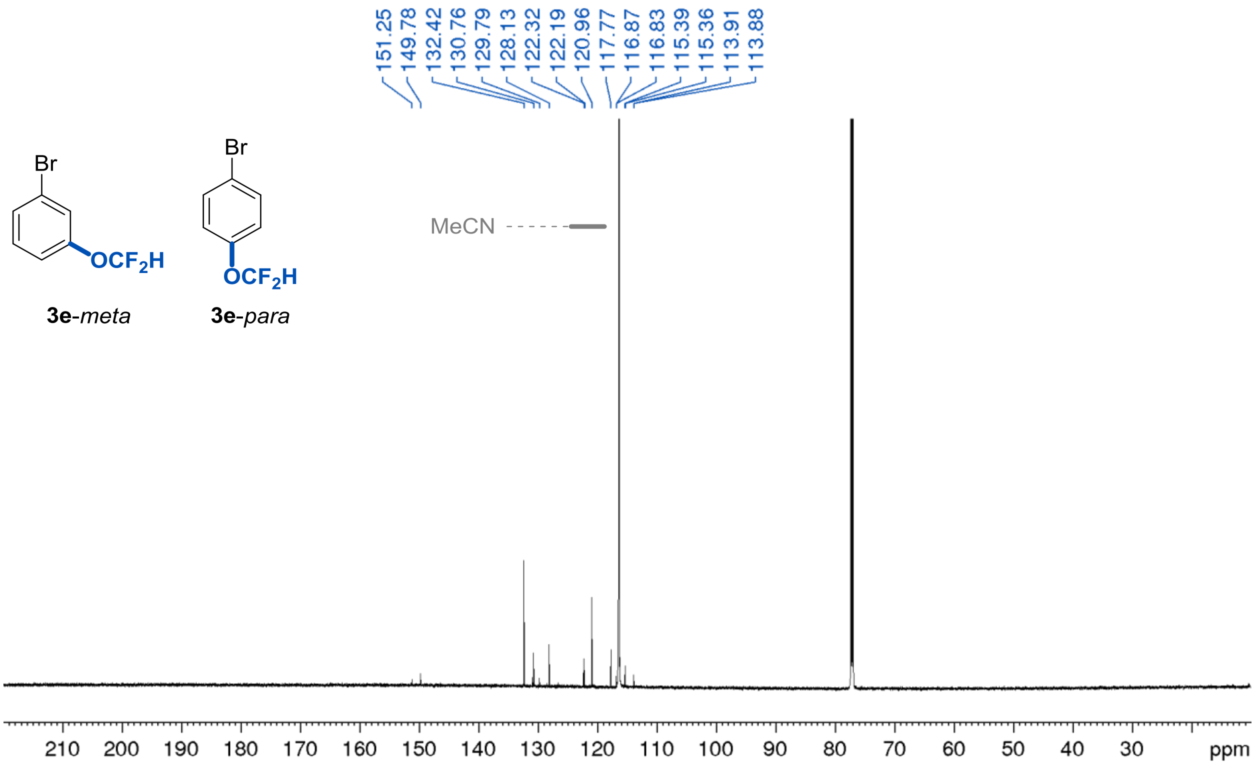

^{19}F NMR (376 MHz, $CDCl_3$, 25 ºC) of 3e-*meta* and -*para*

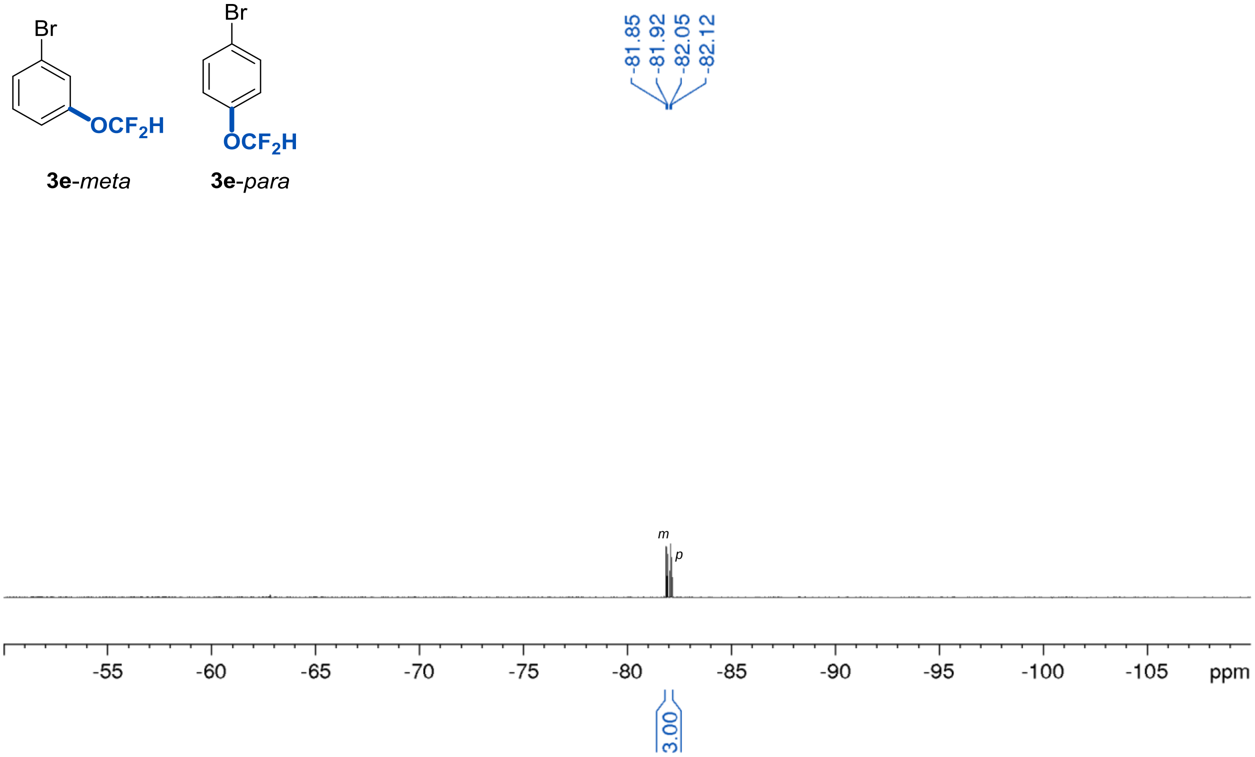

^{1}H NMR (700 MHz, $CDCl_3$, 25 ºC) of 3f and 3f'

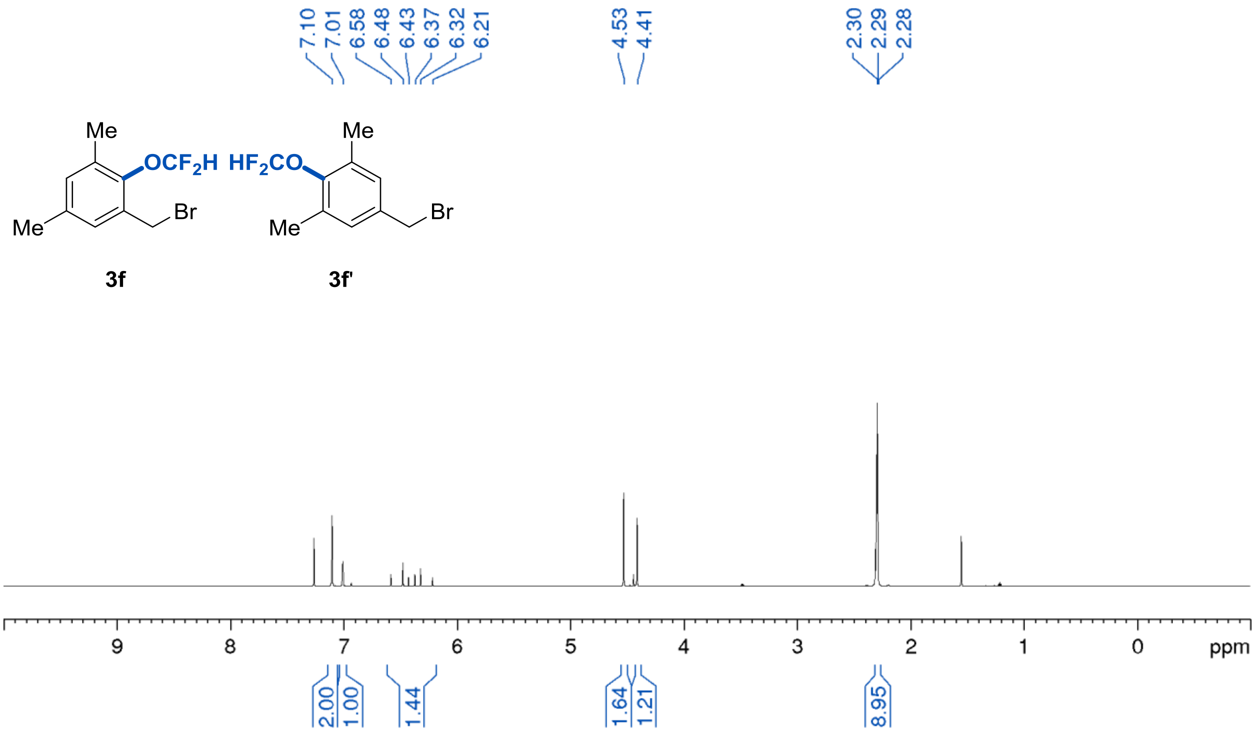

^{13}C NMR (175 MHz, $CDCl_3$, 25 ºC) of 3f and 3f'

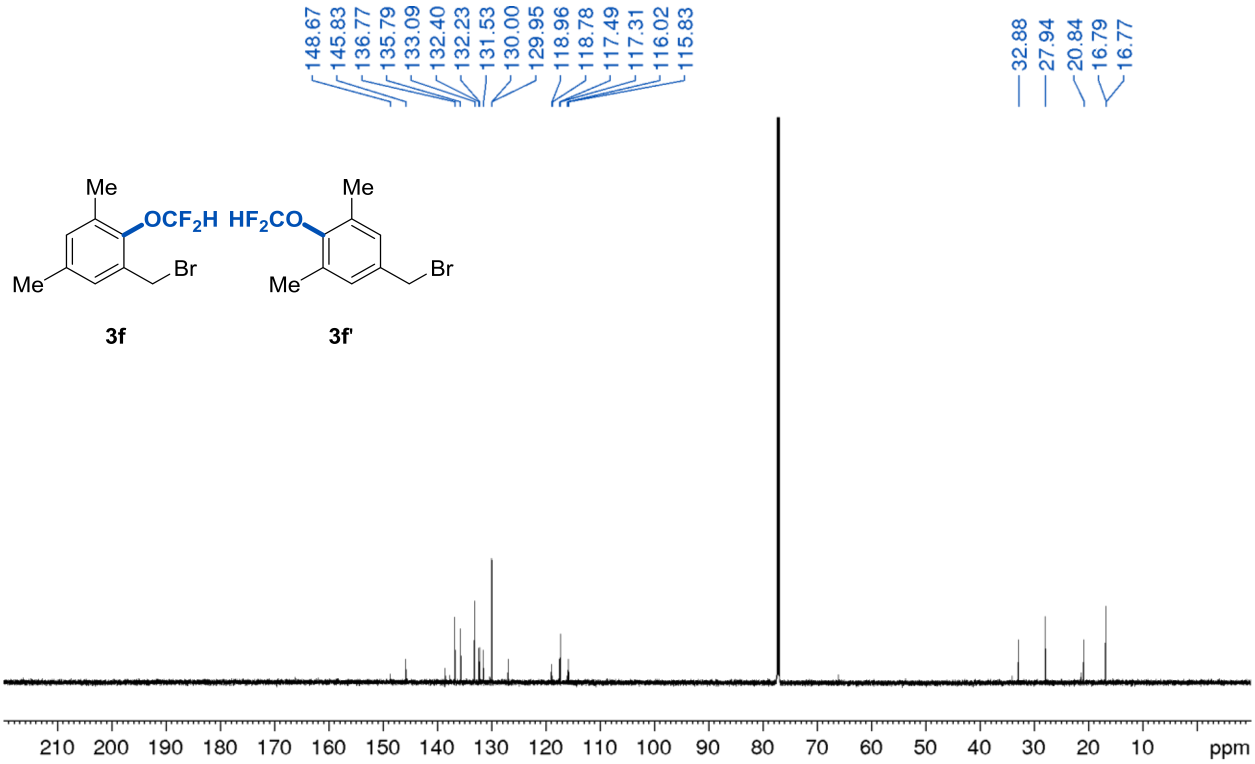

^{19}F NMR (376 MHz, CDCl$_3$, 25 ºC) of 3f and 3f'

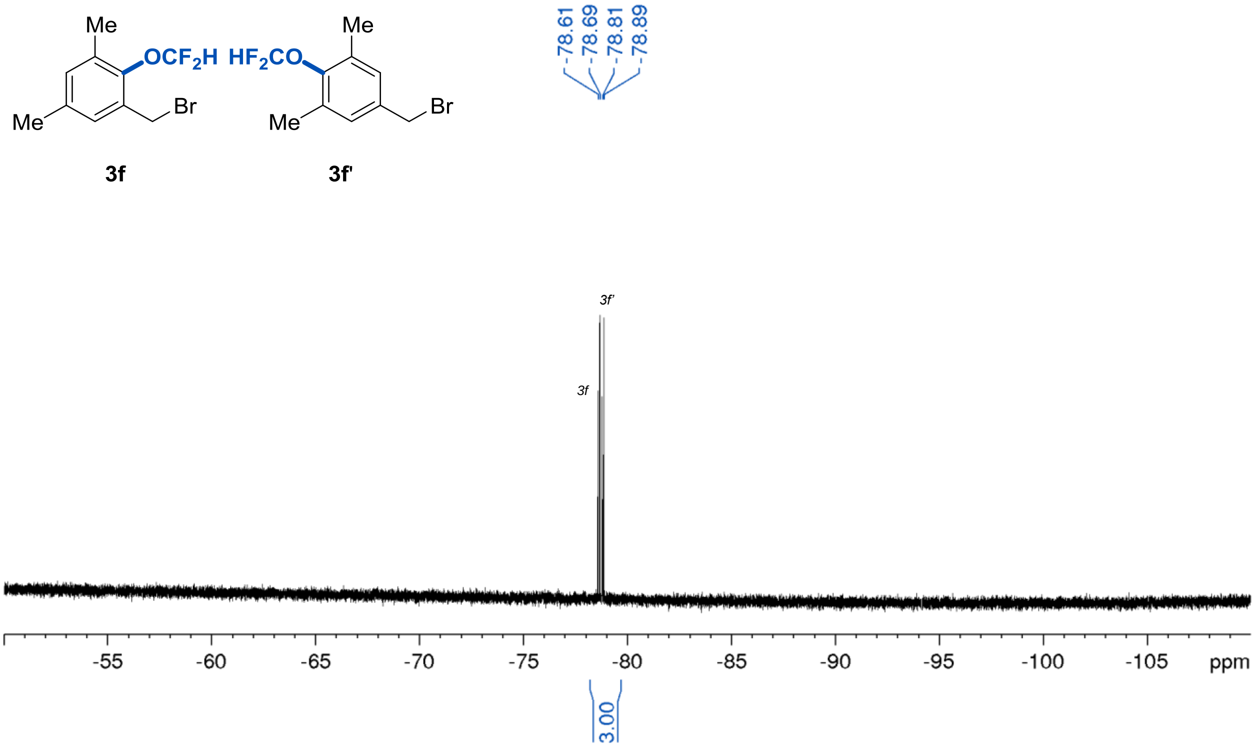

^{1}H NMR (700 MHz, CDCl$_3$, 25 ºC) of 3g-*ortho*, -*meta*, and -*para*

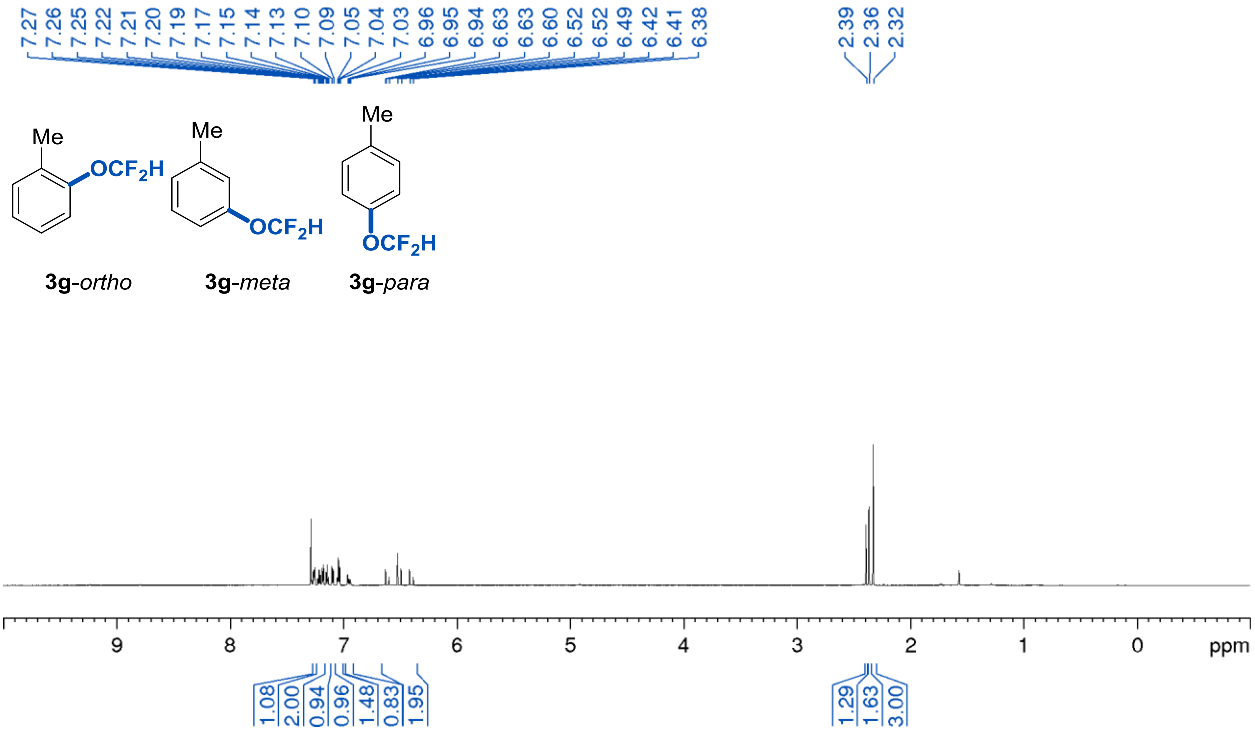

^{13}C NMR (175 MHz, CDCl$_3$, 25 ºC) of 3g-*ortho*, -*meta*, and -*para*

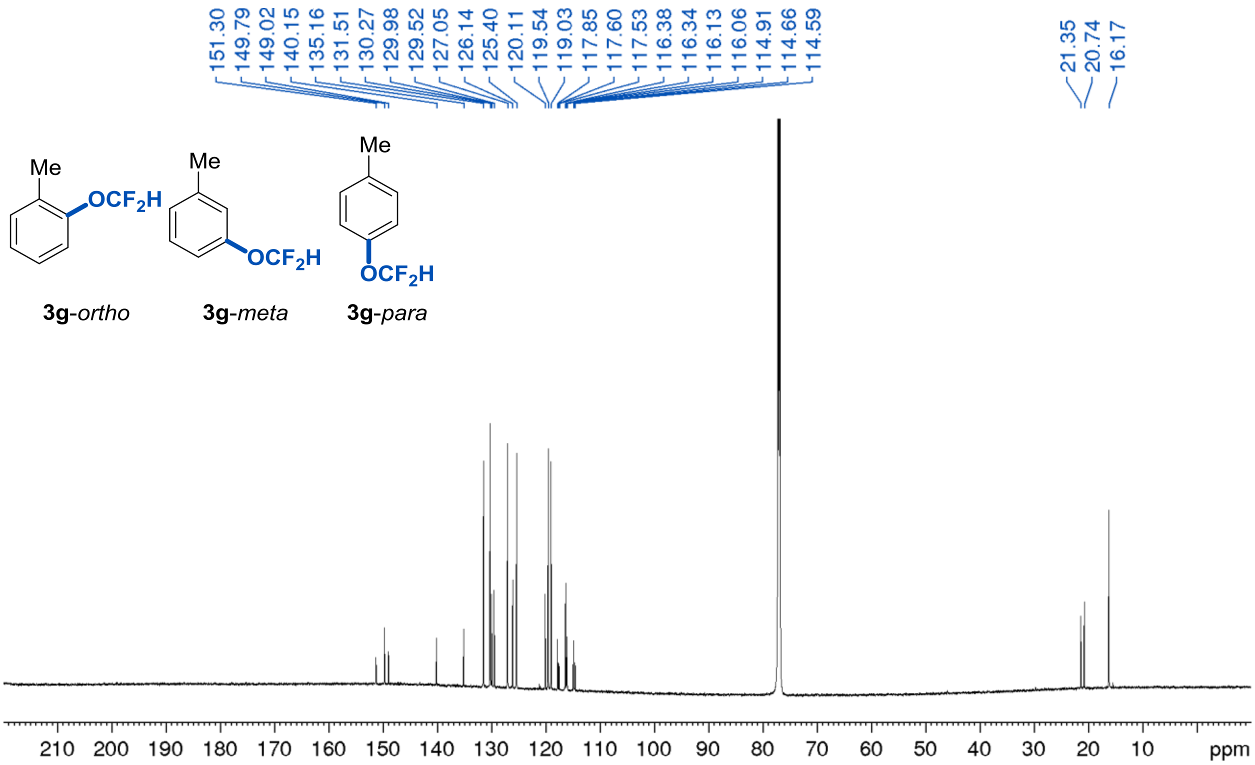

^{19}F NMR (376 MHz, CDCl$_3$, 25 ºC) of 3g-*ortho*, -*meta*, and -*para*

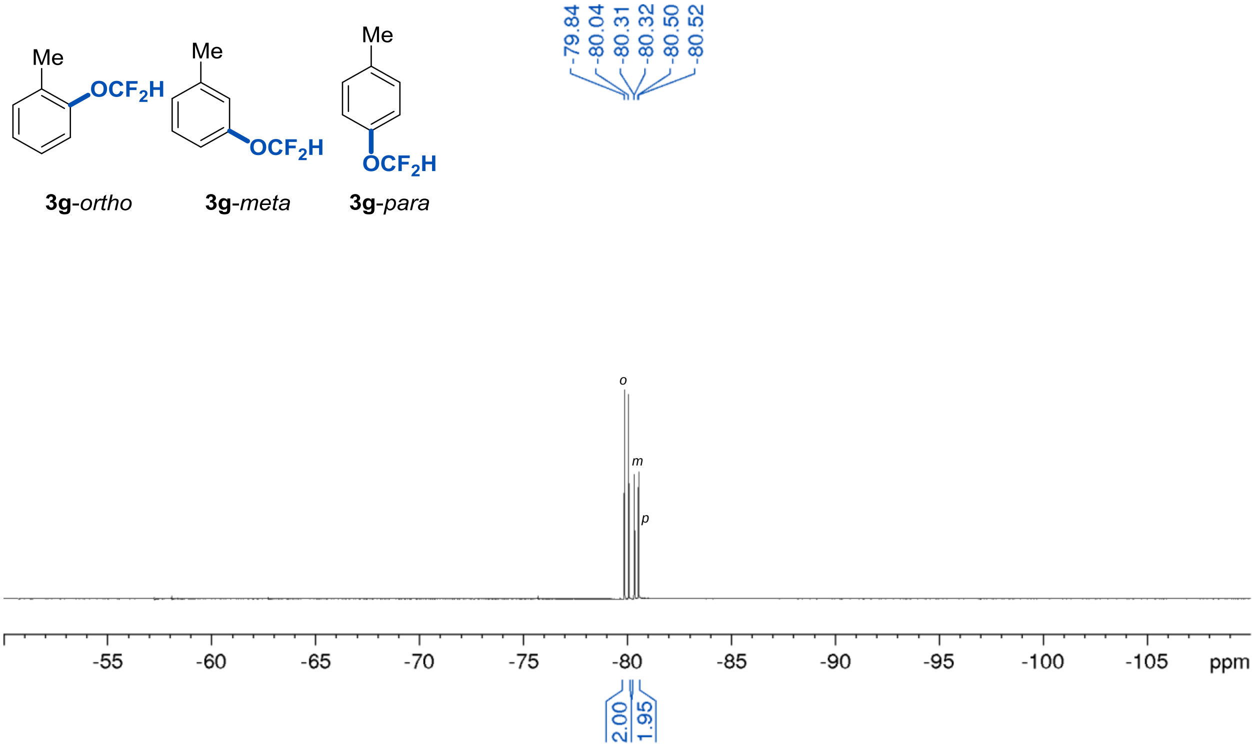

^{1}H NMR (700 MHz, $CDCl_3$, 25 °C) of 3h

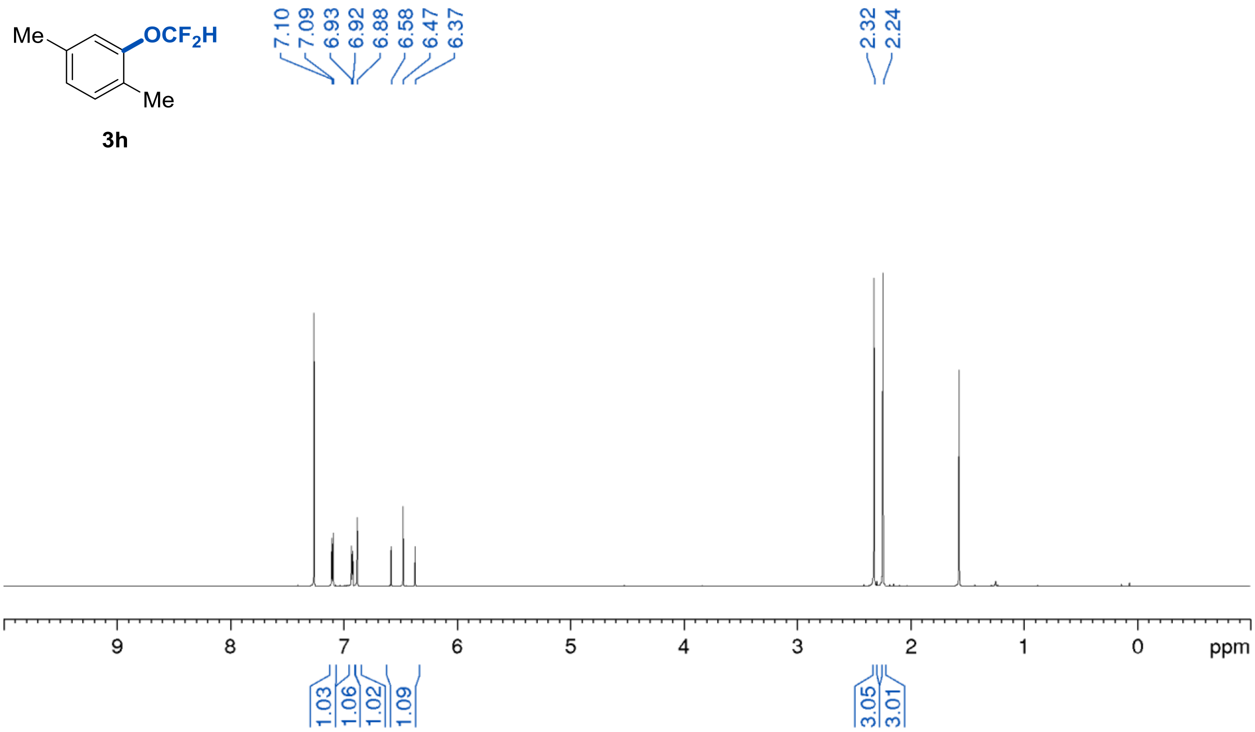

^{13}C NMR (175 MHz, $CDCl_3$, 25 °C) of 3h

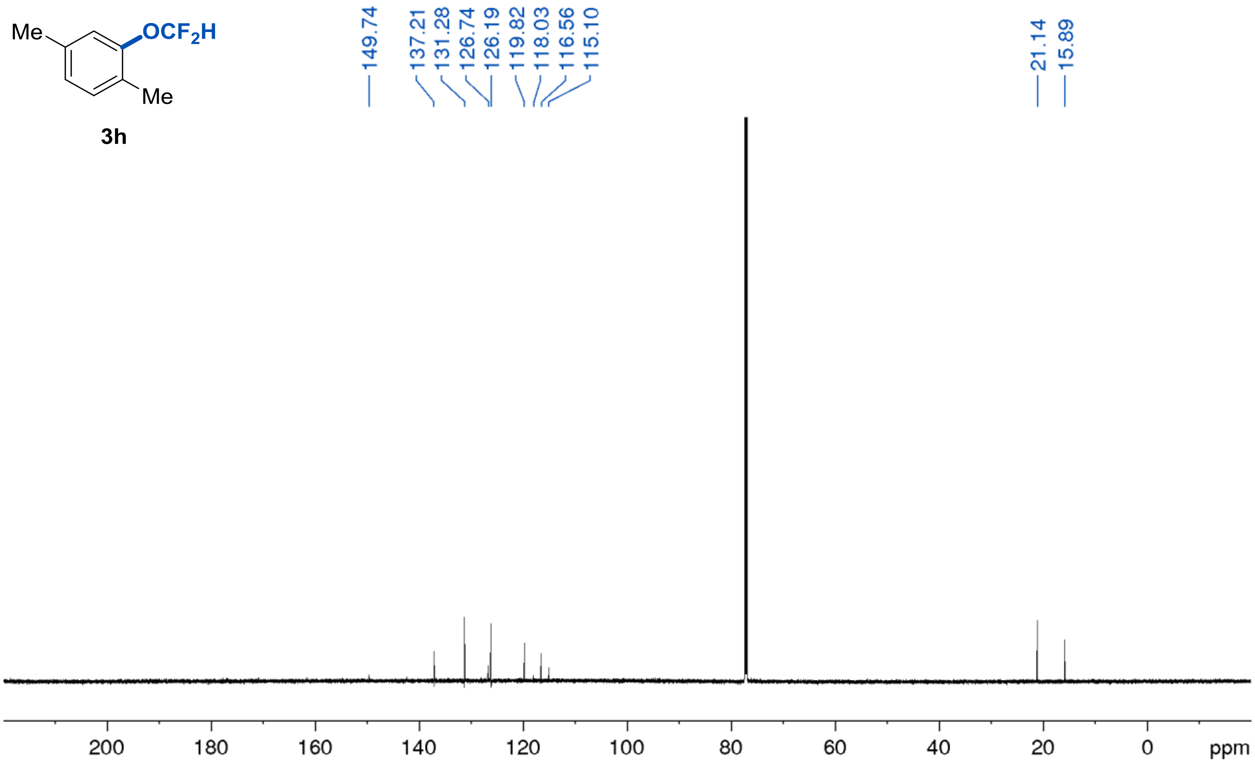

^{19}F NMR (376 MHz, $CDCl_3$, 25 ºC) of 3h

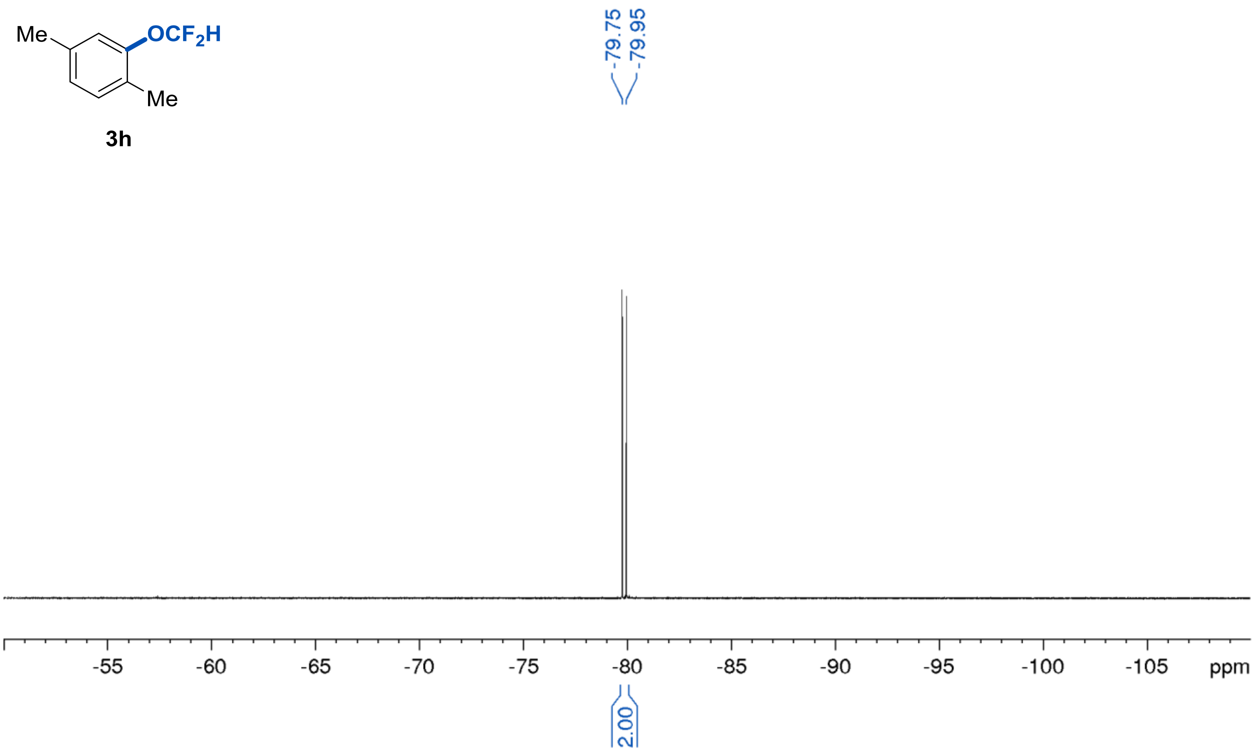

^{1}H NMR (700 MHz, $CDCl_3$, 25 ºC) of 3i-*ortho*, -*meta*, and -*para*

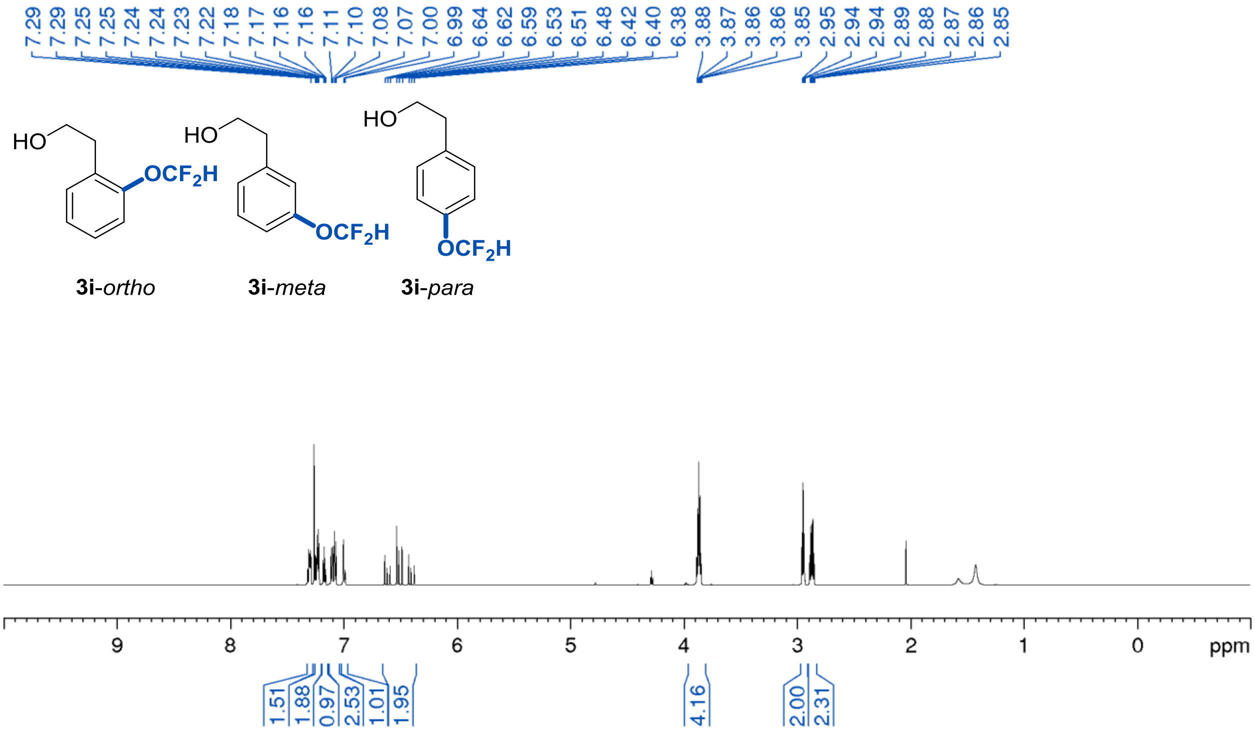

^{13}C NMR (175 MHz, CDCl$_3$, 25 ºC) of 3i-*ortho*, -*meta*, and -*para*

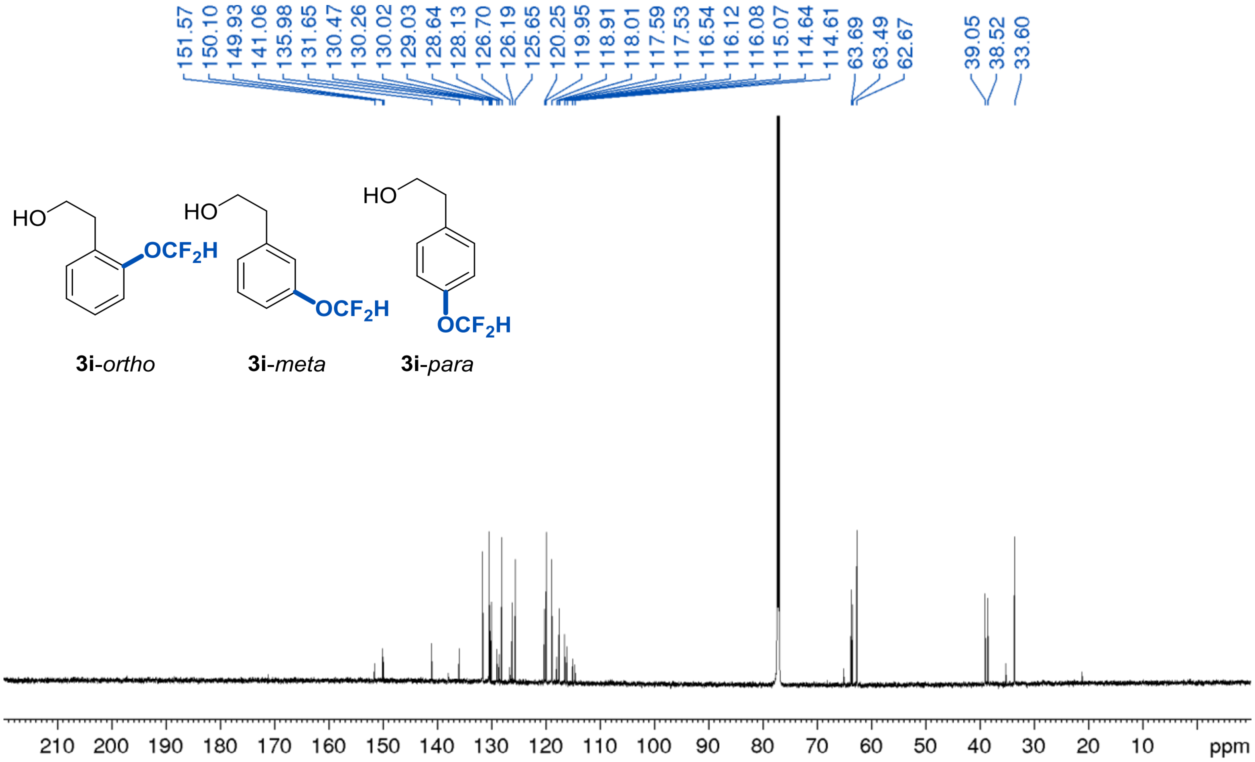

^{19}F NMR (376 MHz, CDCl$_3$, 25 ºC) of 3i-*ortho*, -*meta*, and -*para*

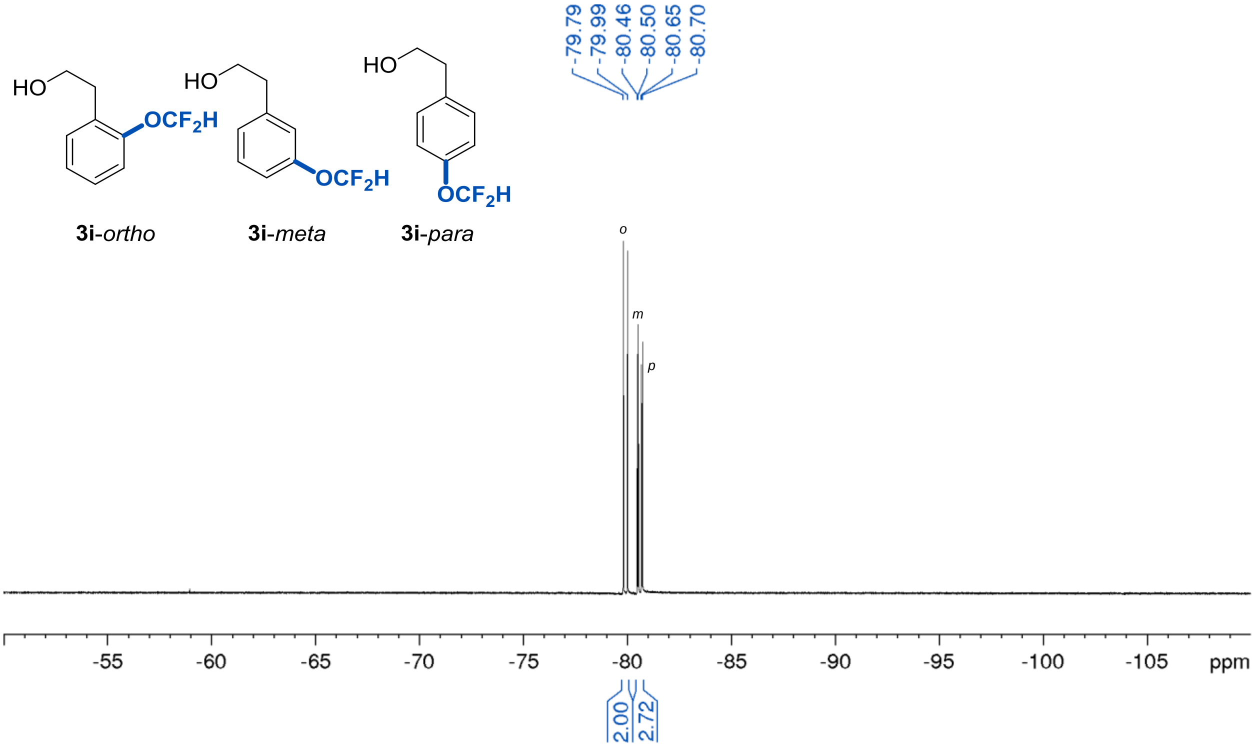

^{1}H NMR (700 MHz, $CDCl_3$, 25 °C) of 3j

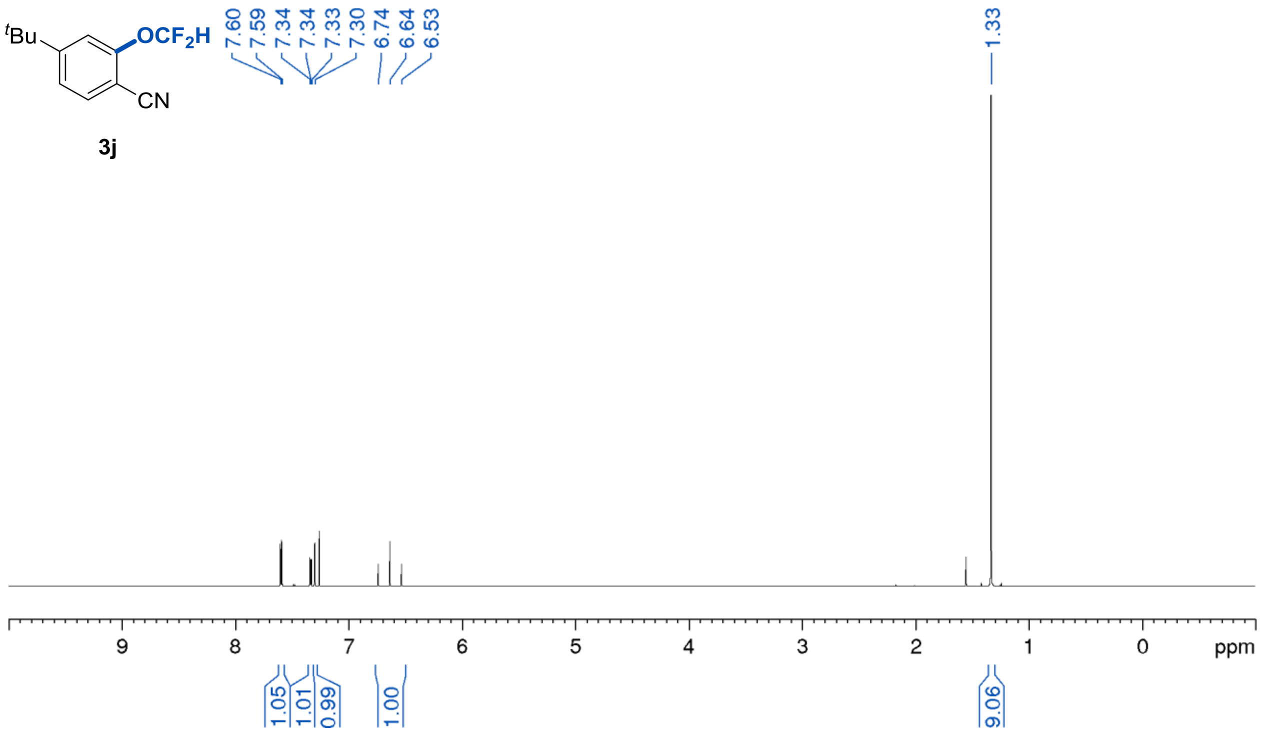

^{13}C NMR (175 MHz, $CDCl_3$, 25 °C) of 3j

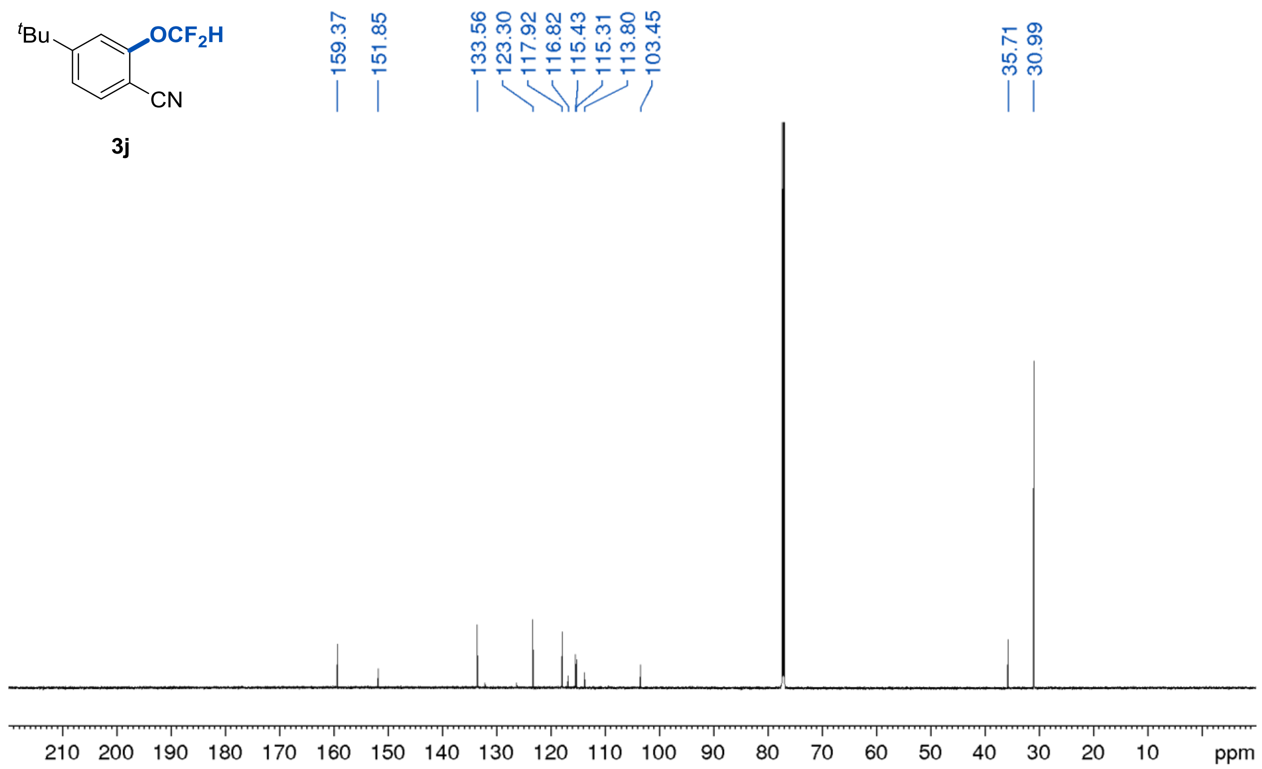

^{19}F NMR (376 MHz, CDCl$_3$, 25 ºC) of 3j

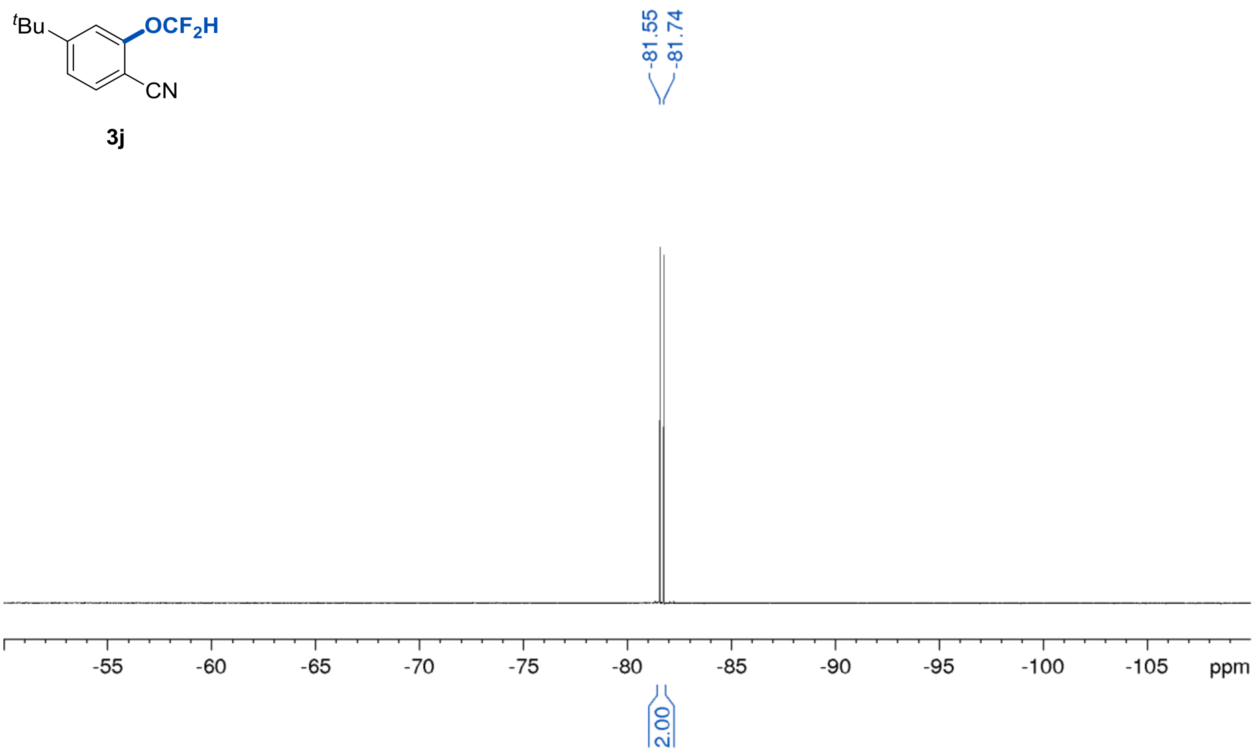

^{1}H NMR (700 MHz, CDCl$_3$, 25 ºC) of 3j'

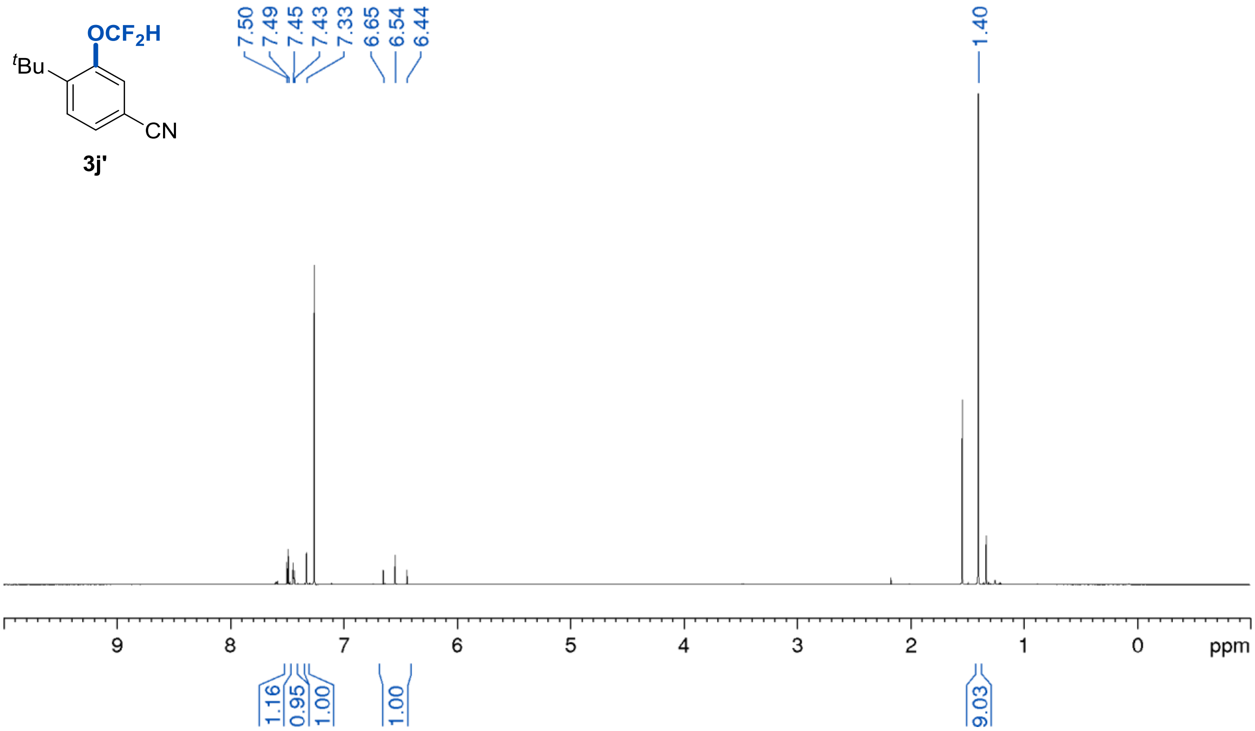

^{13}C NMR (175 MHz, $CDCl_3$, 25 ºC) of 3j’

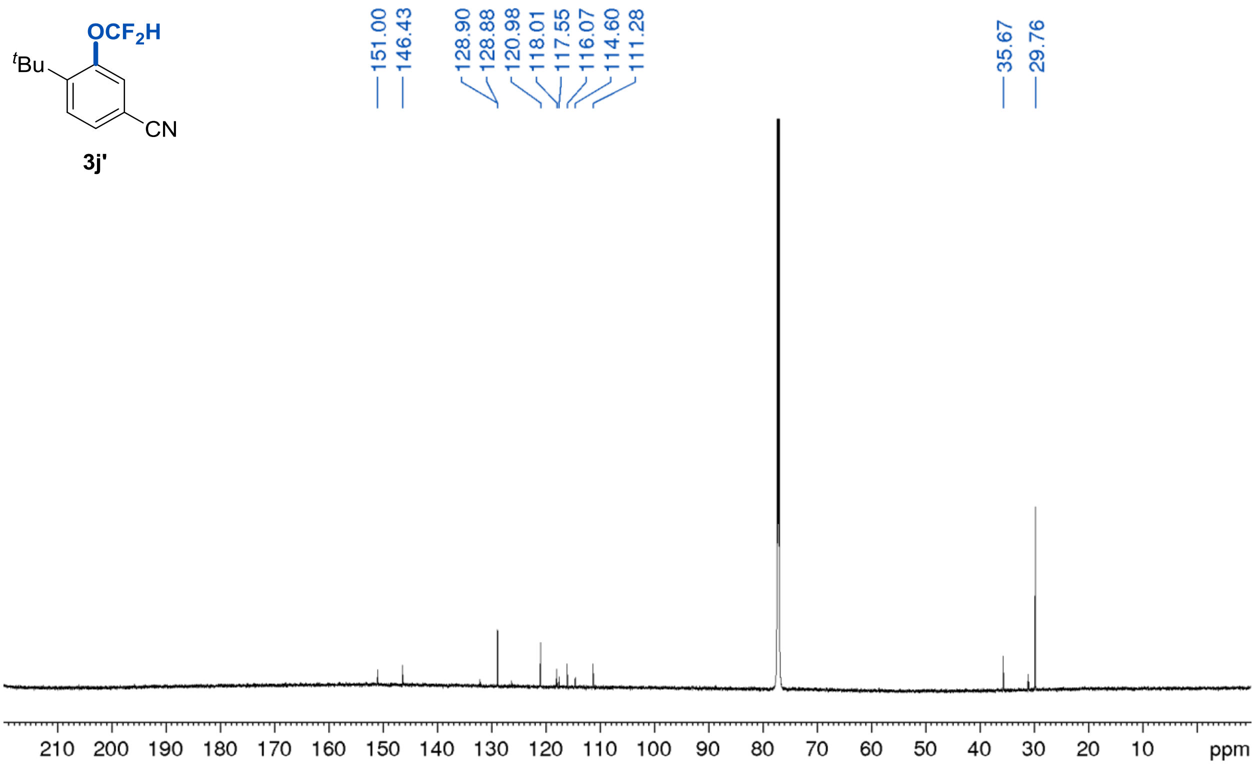

^{19}F NMR (376 MHz, $CDCl_3$, 25 ºC) of 3j’

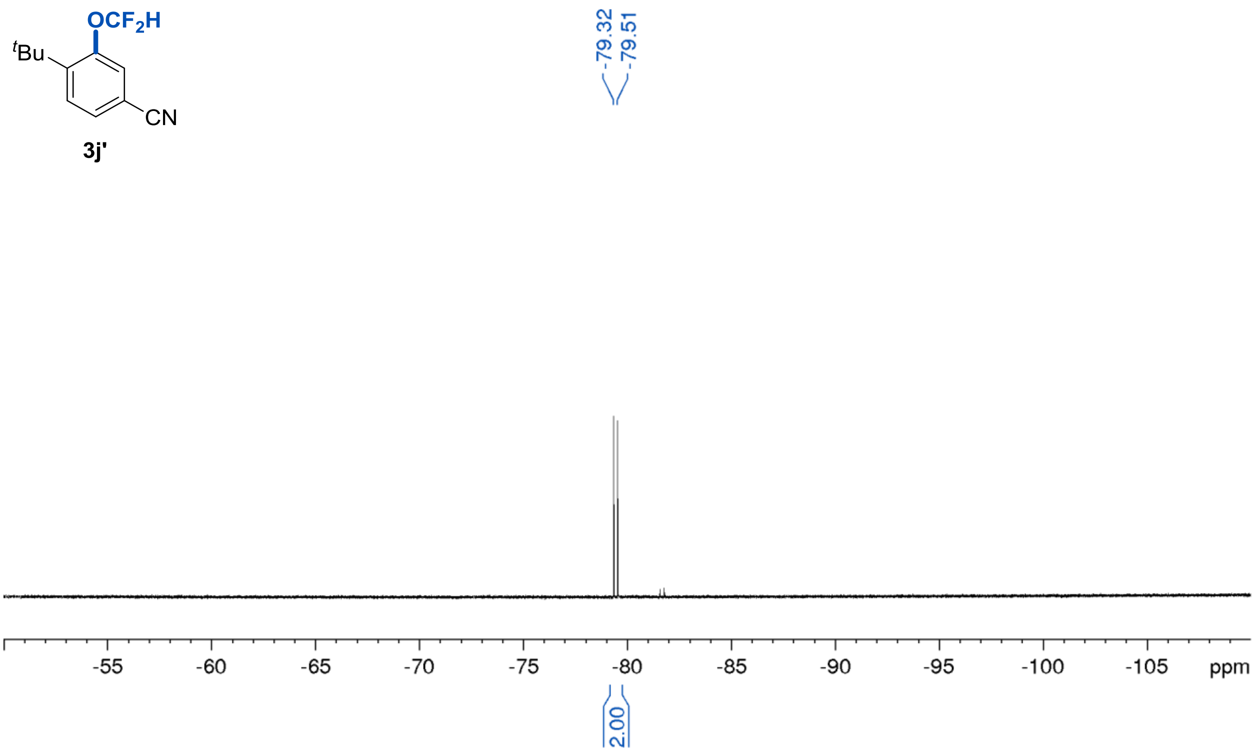

^{1}H NMR (700 MHz, CDCl$_3$, 25 °C) of 3k and 3k'

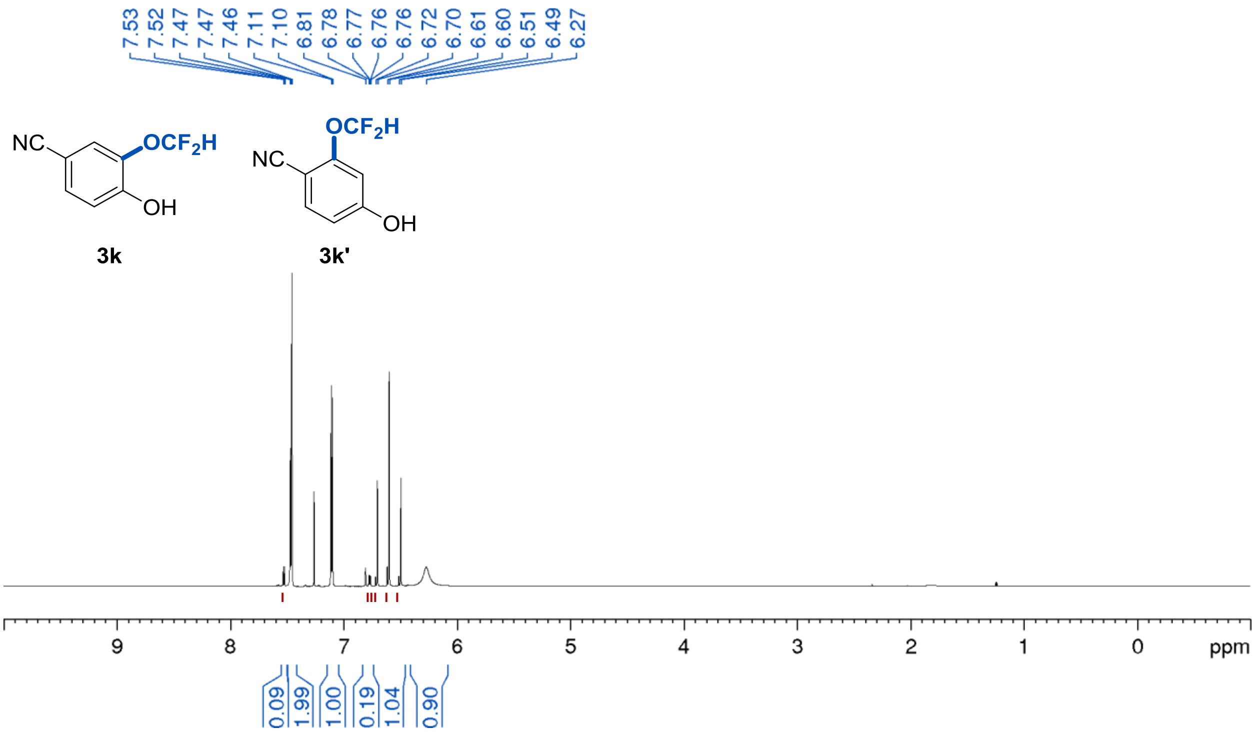

^{13}C NMR (175 MHz, CDCl$_3$, 25 °C) of 3k and 3k'

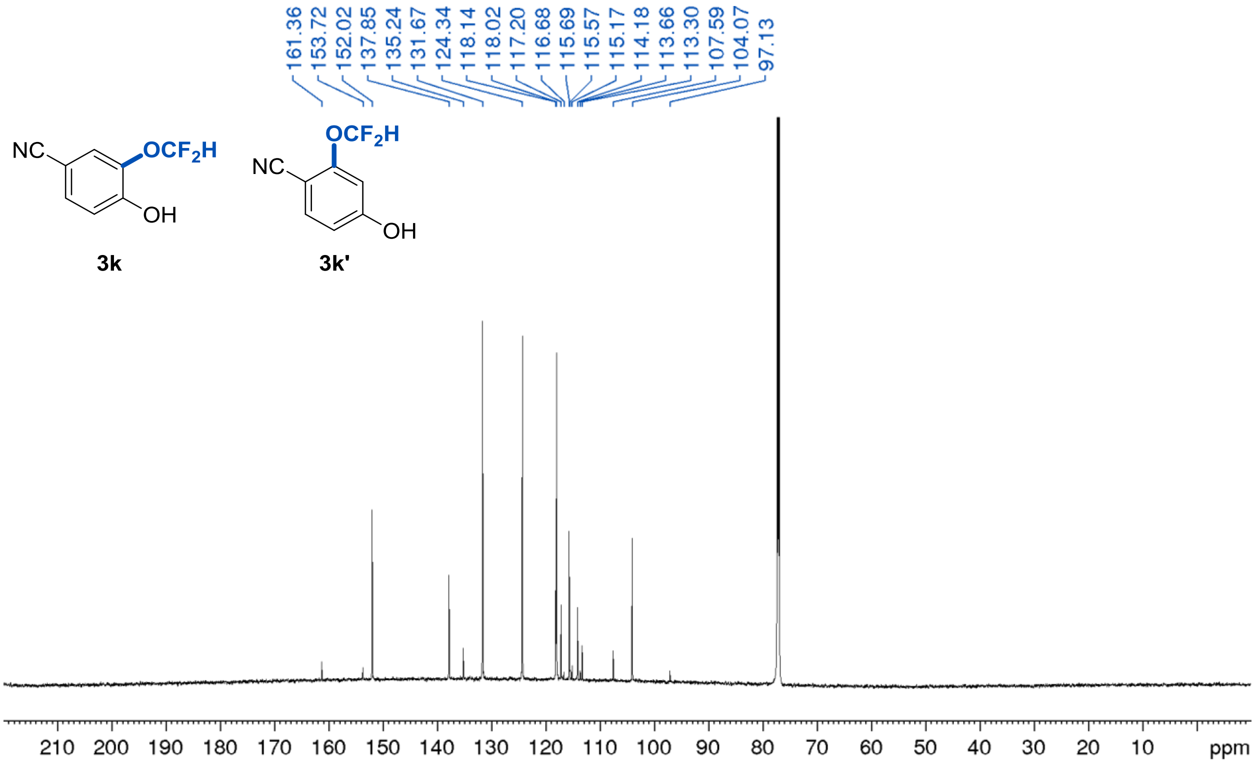

^{19}F NMR (376 MHz, $CDCl_3$, 25 ºC) of 3k and 3k'

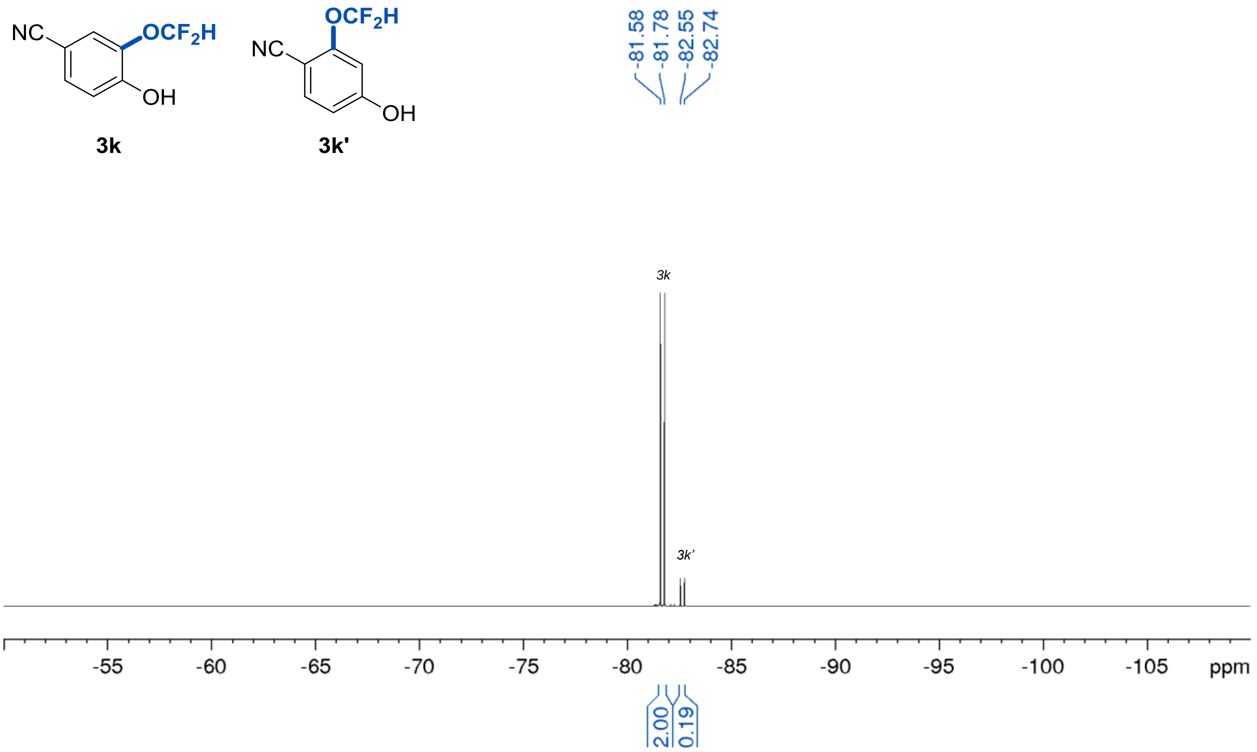

^{1}H NMR (700 MHz, $CDCl_3$, 25 ºC) of 3m, 3m', and 3m''

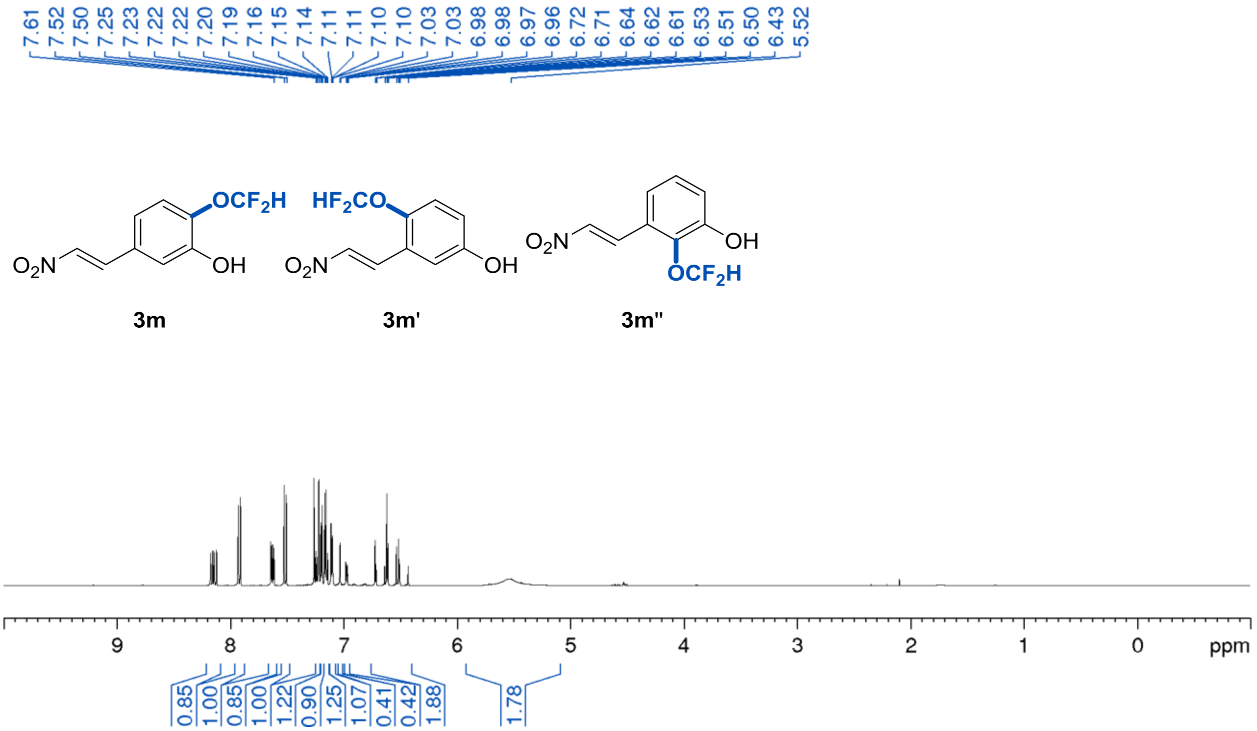

^{13}C NMR (175 MHz, $CDCl_3$, 25 ºC) of 3m, 3m’, and 3m’’

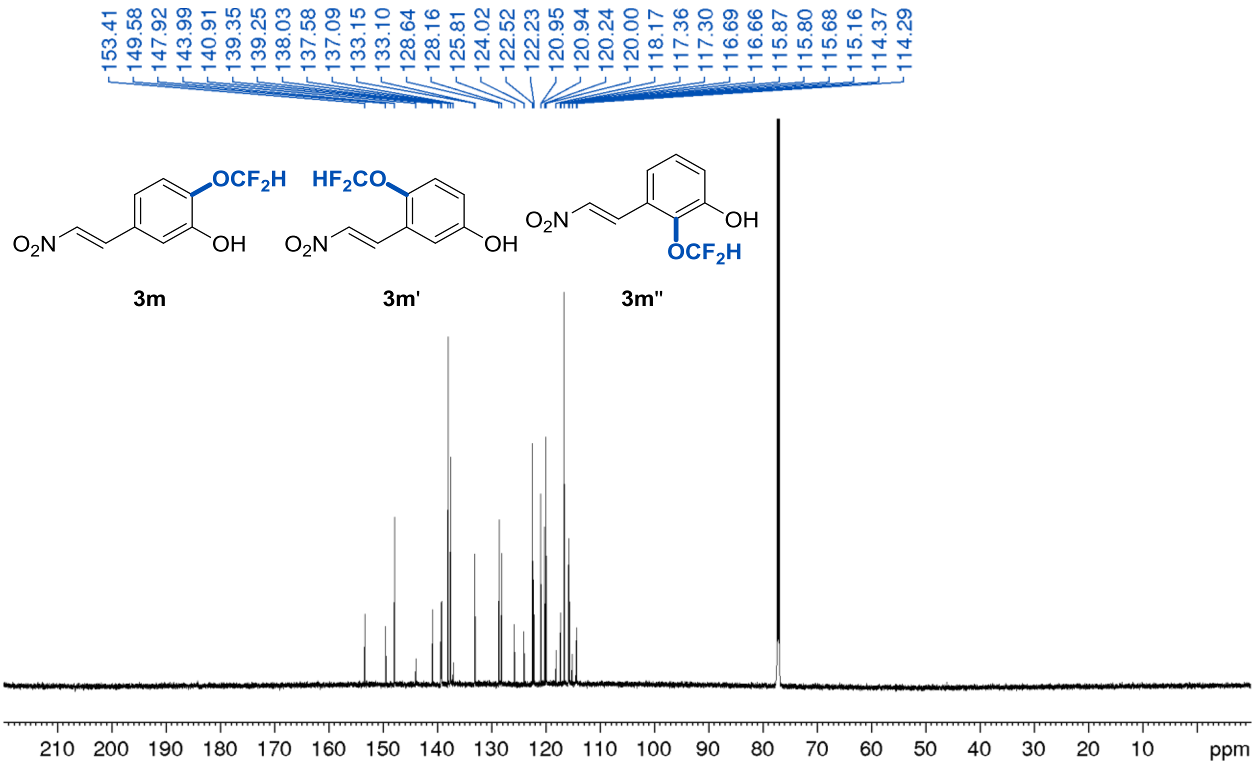

^{19}F NMR (376 MHz, $CDCl_3$, 25 ºC) of 3m, 3m’, and 3m’’

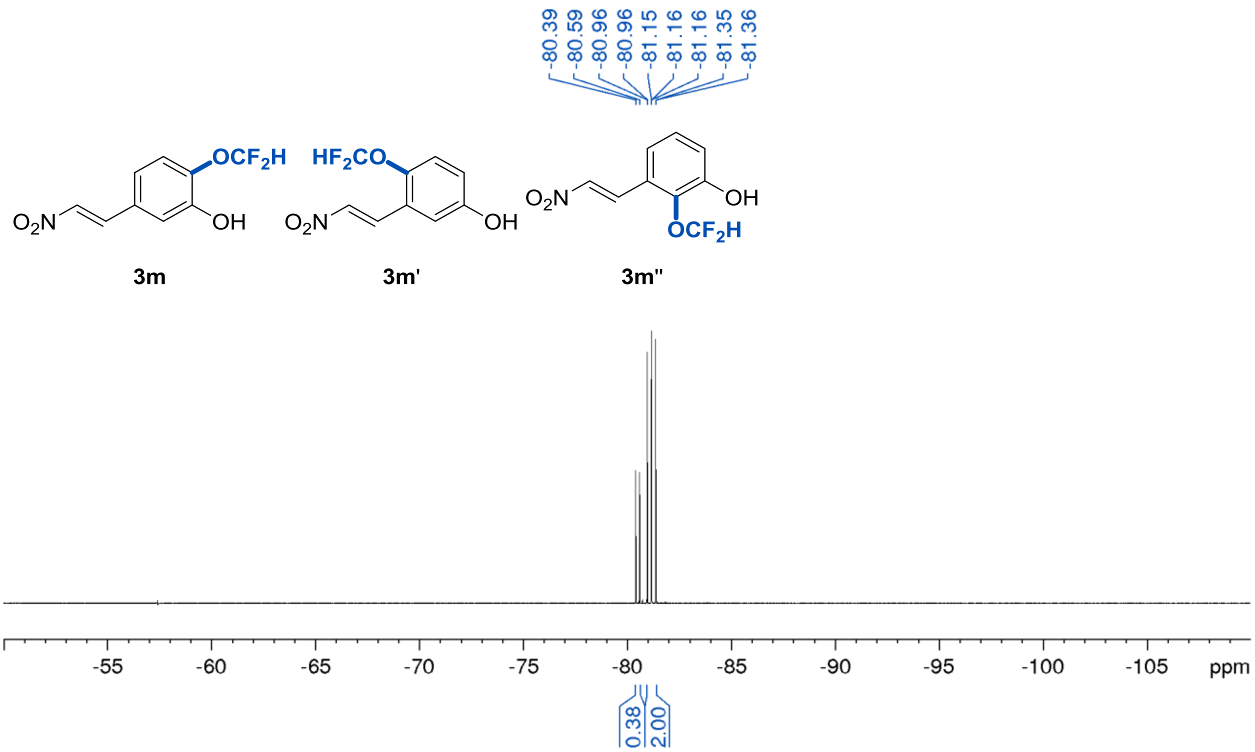

^{1}H NMR (700 MHz, $CDCl_3$, 25 ºC) of 3n and 3n'

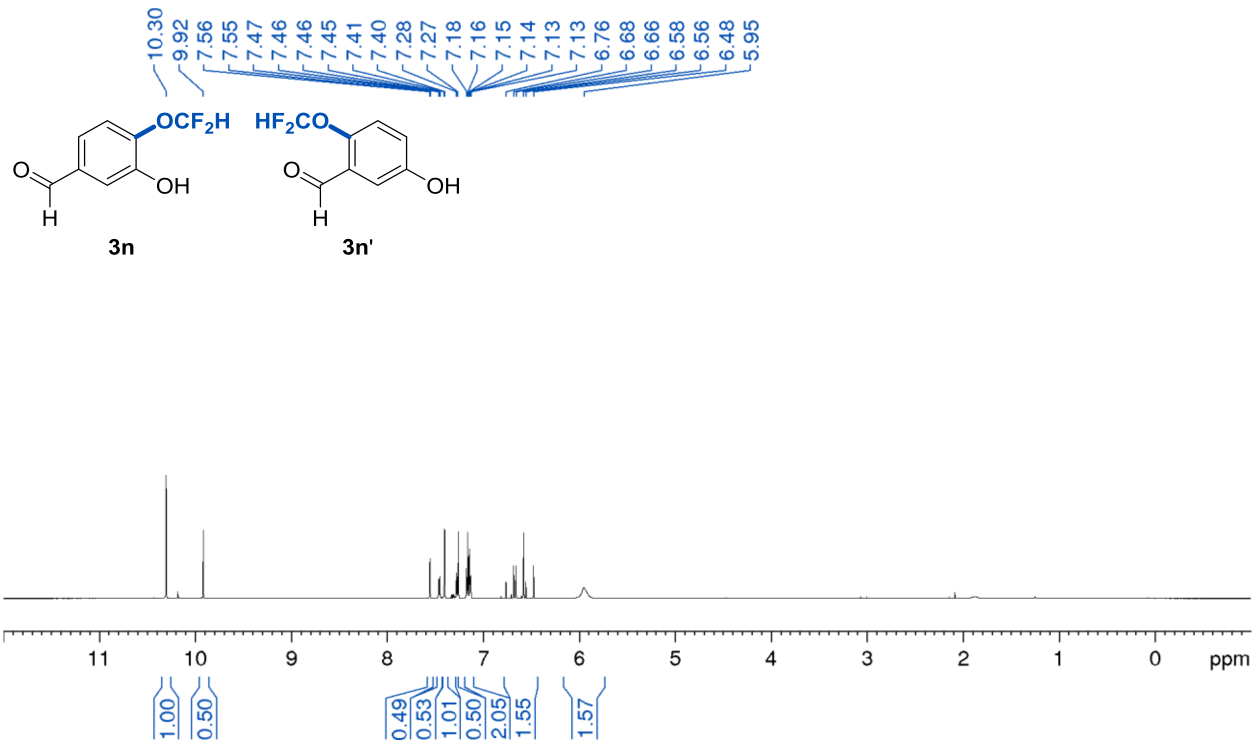

^{13}C NMR (175 MHz, $CDCl_3$, 25 ºC) of 3n and 3n'

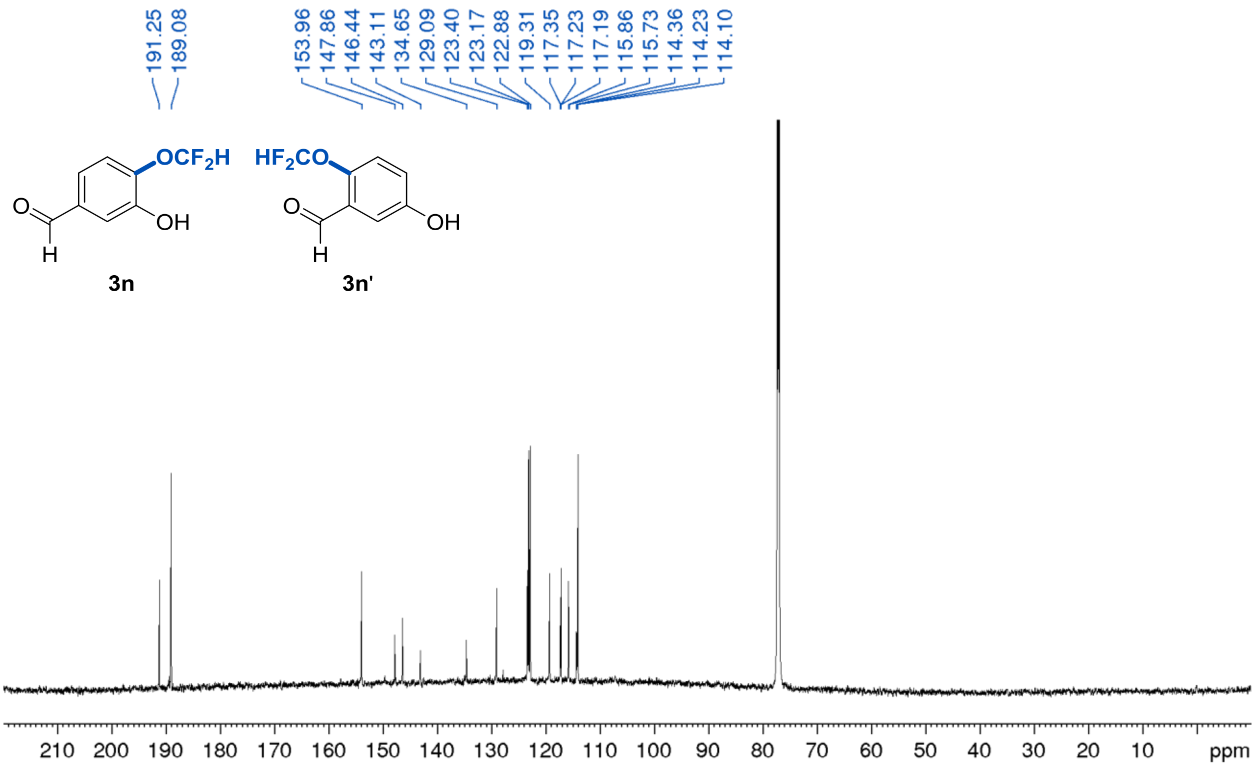

^{19}F NMR (376 MHz, CDCl$_3$, 25 ºC) of 3n and 3n'

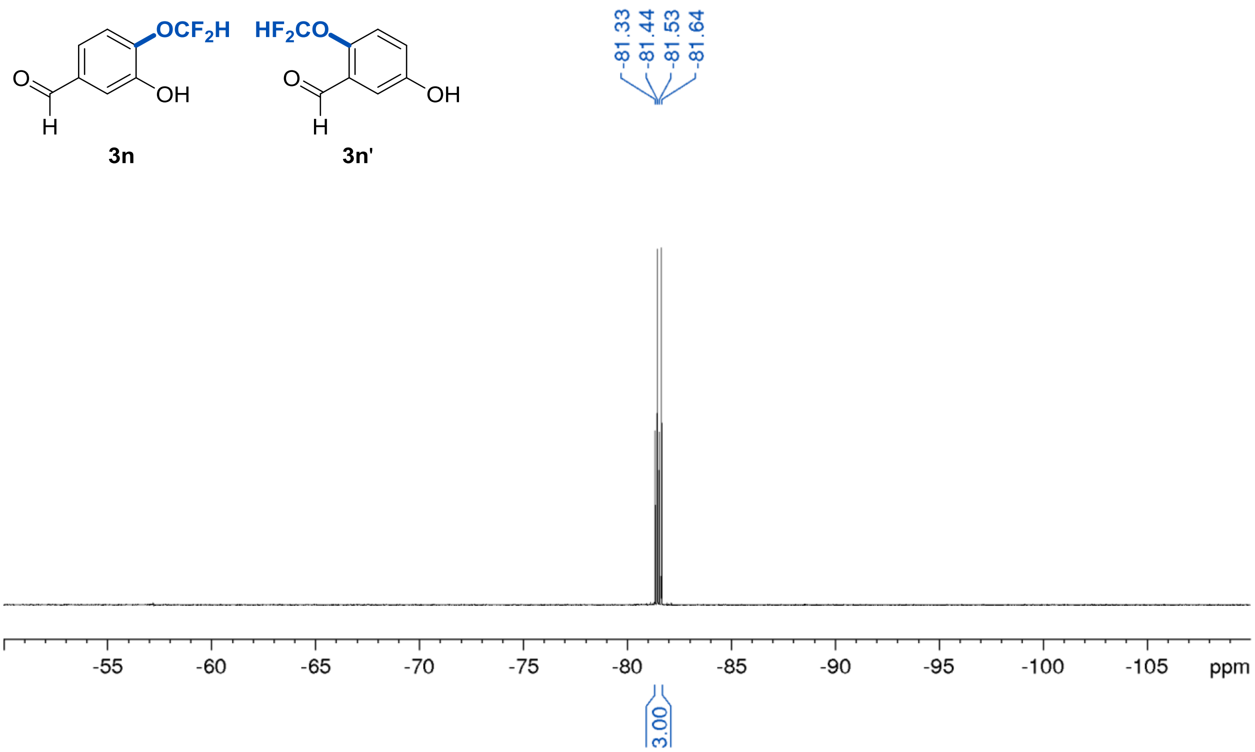

^{1}H NMR (700 MHz, CDCl$_3$, 25 ºC) of 3n''

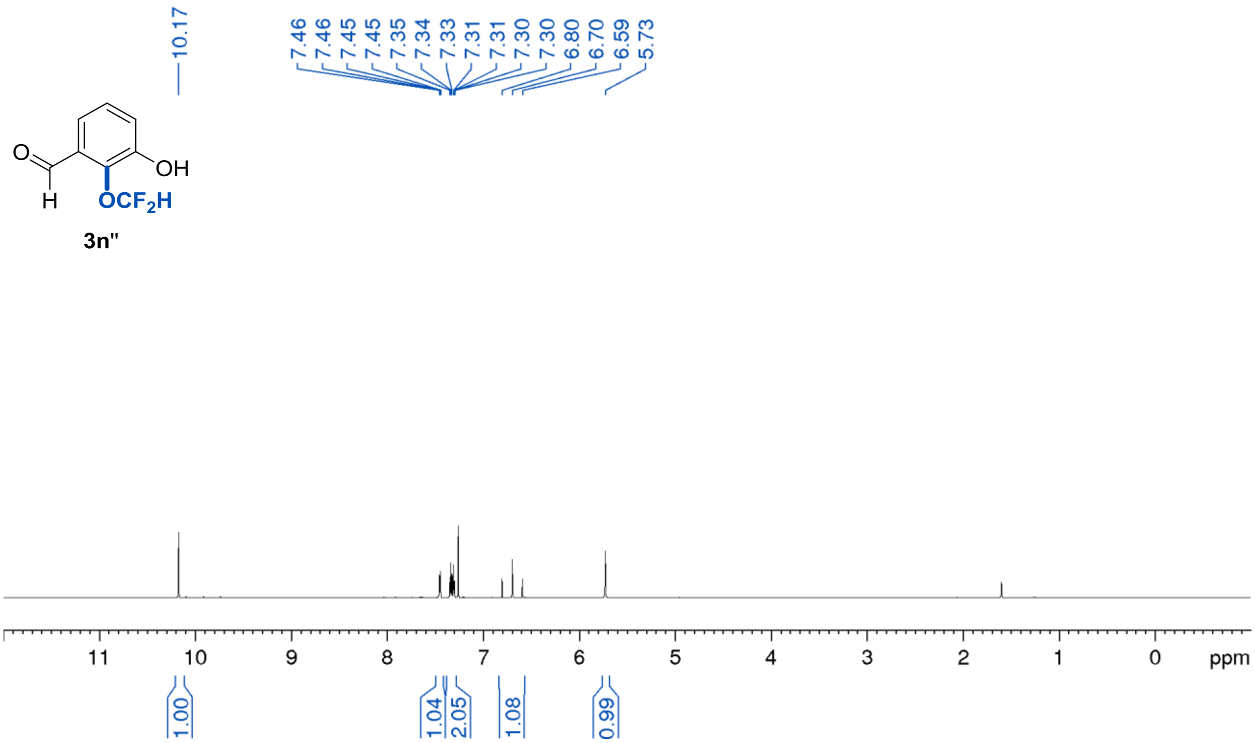

^{13}C NMR (175 MHz, $CDCl_3$, 25 ºC) of 3n''

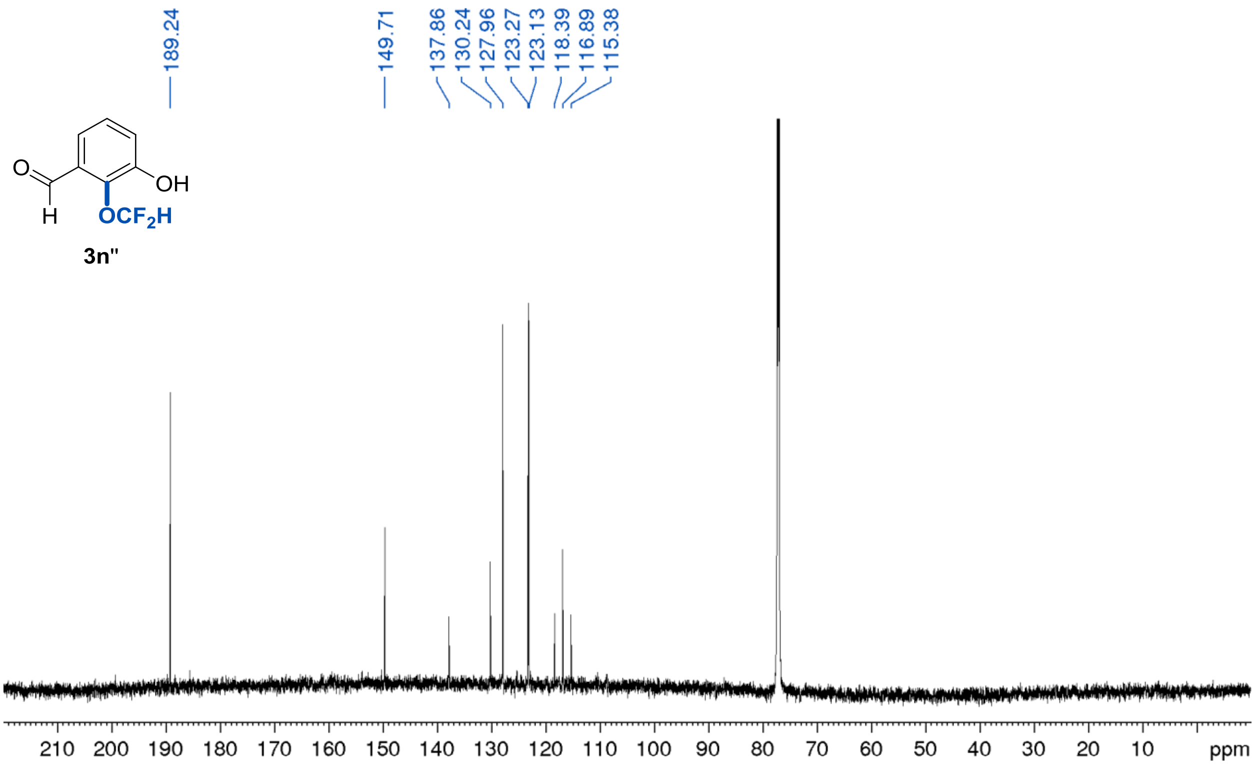

^{19}F NMR (376 MHz, $CDCl_3$, 25 ºC) of 3n''

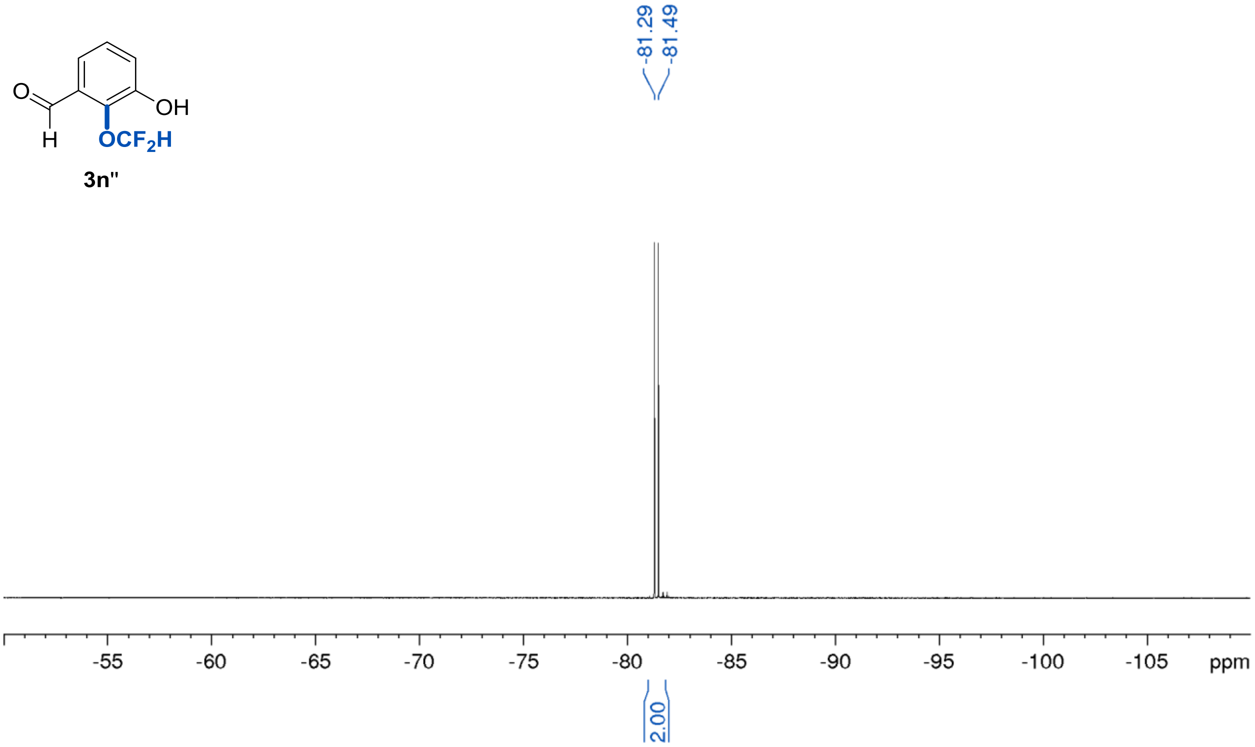

^{1}H NMR (700 MHz, $CDCl_3$, 25 ºC) of 3l

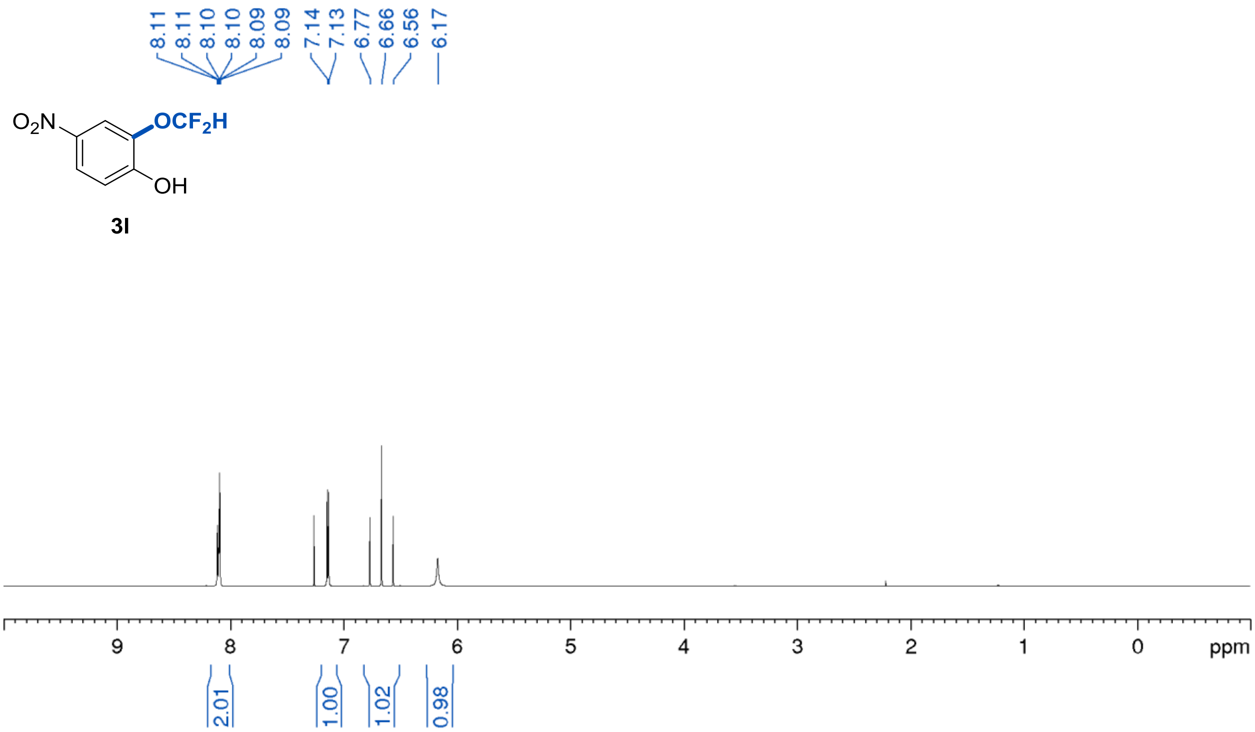

^{13}C NMR (175 MHz, $CDCl_3$, 25 ºC) of 3l

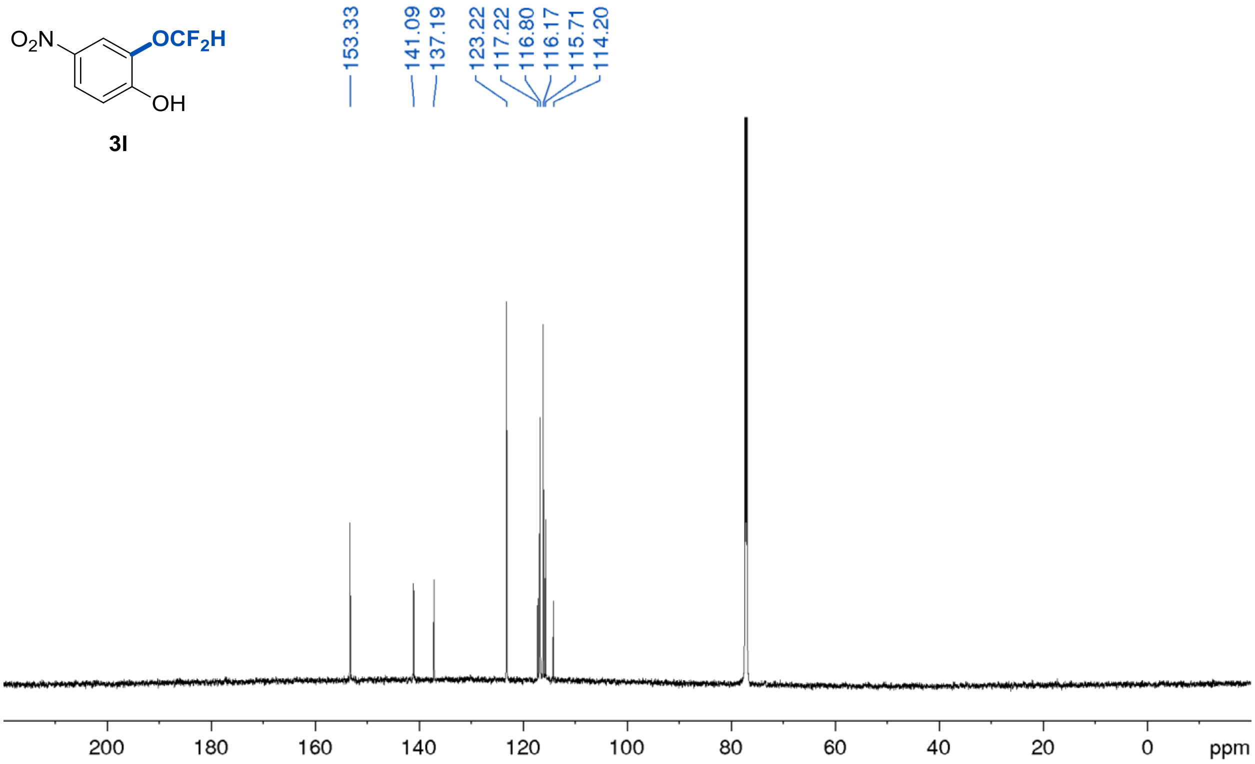

^{19}F NMR (376 MHz, CDCl$_3$, 25 ºC) of 3l

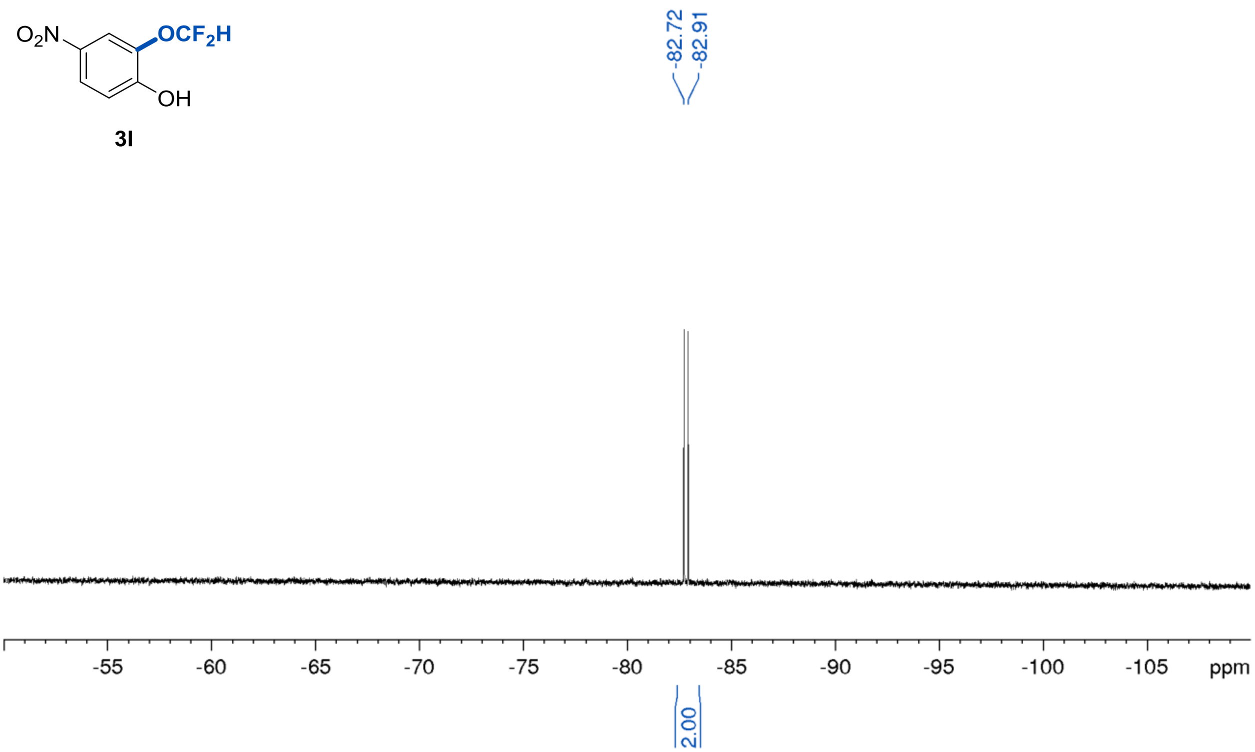

^{1}H NMR (700 MHz, CDCl$_3$, 25 ºC) of 3o-*ortho* and -*para*

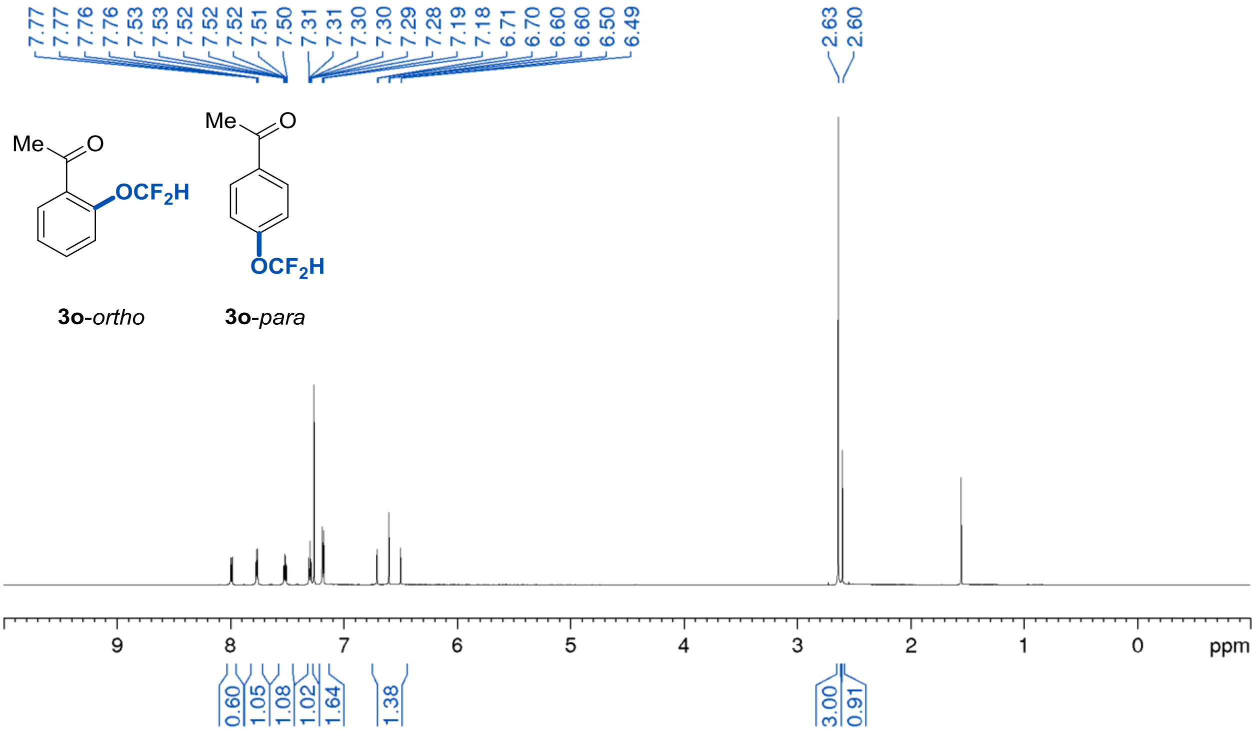

^{13}C NMR (175 MHz, $CDCl_3$, 25 ºC) of 3o-*ortho* and -*para*

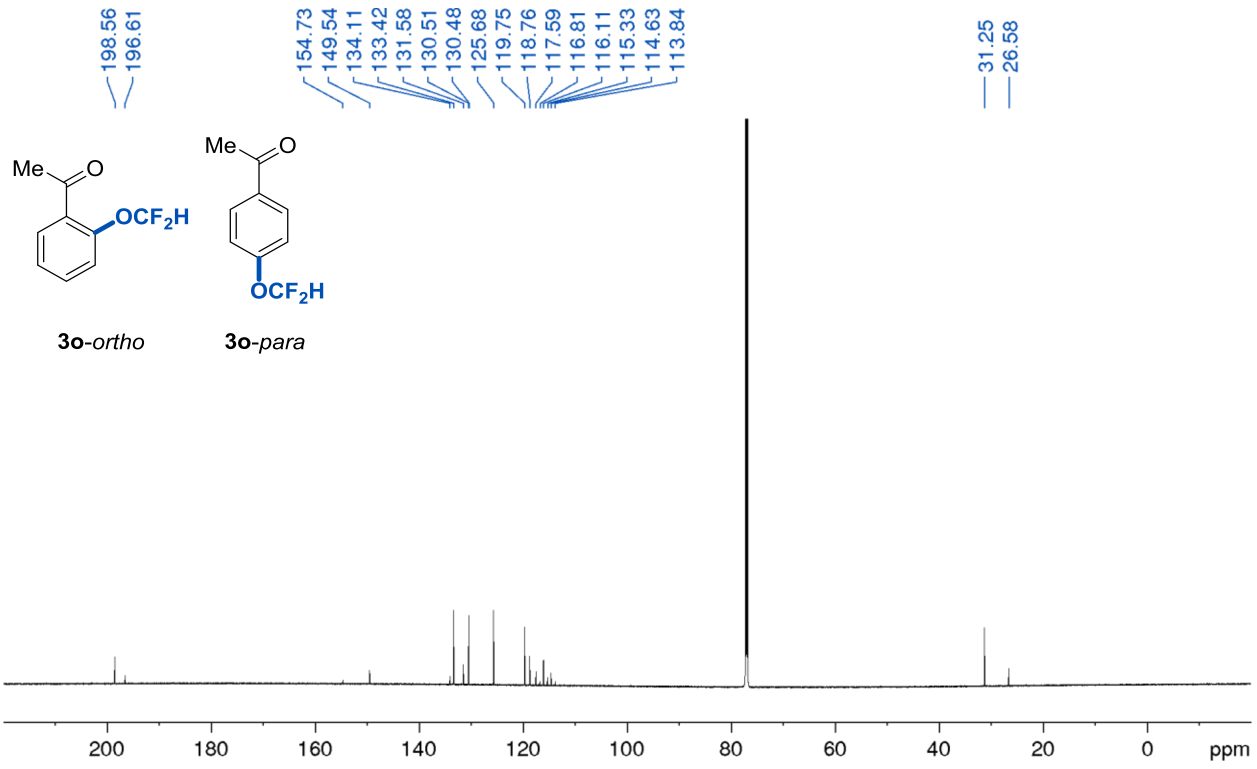

^{19}F NMR (376 MHz, $CDCl_3$, 25 ºC) of 3o-*ortho* and -*para*

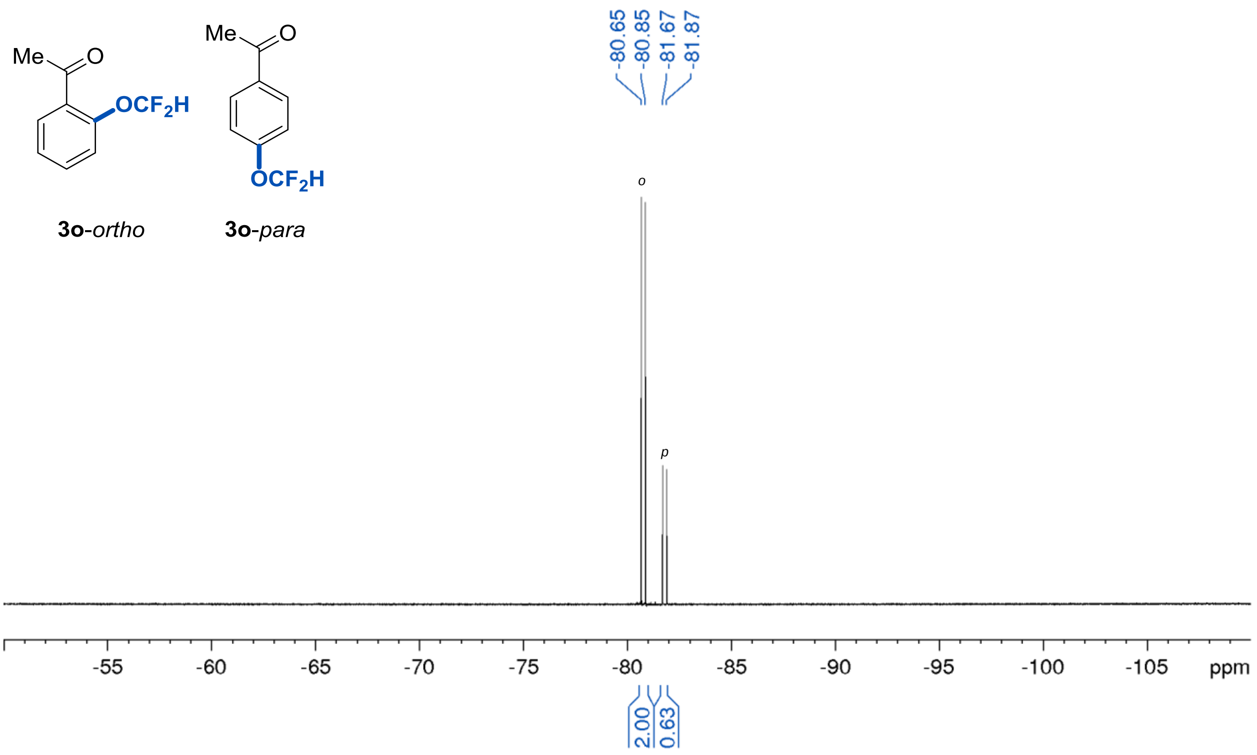

^{1}H NMR (700 MHz, CDCl$_3$, 25 °C) of 3o-*meta*

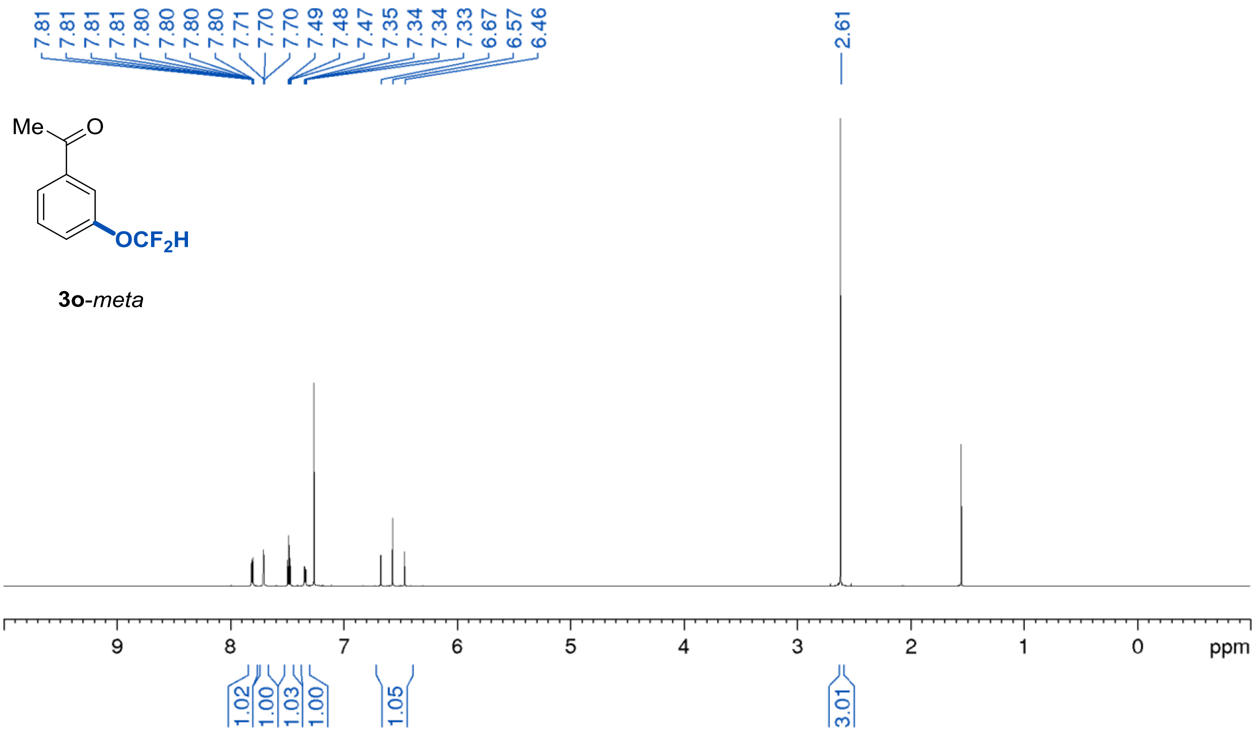

^{13}C NMR (175 MHz, CDCl$_3$, 25 °C) of 3o-*meta*

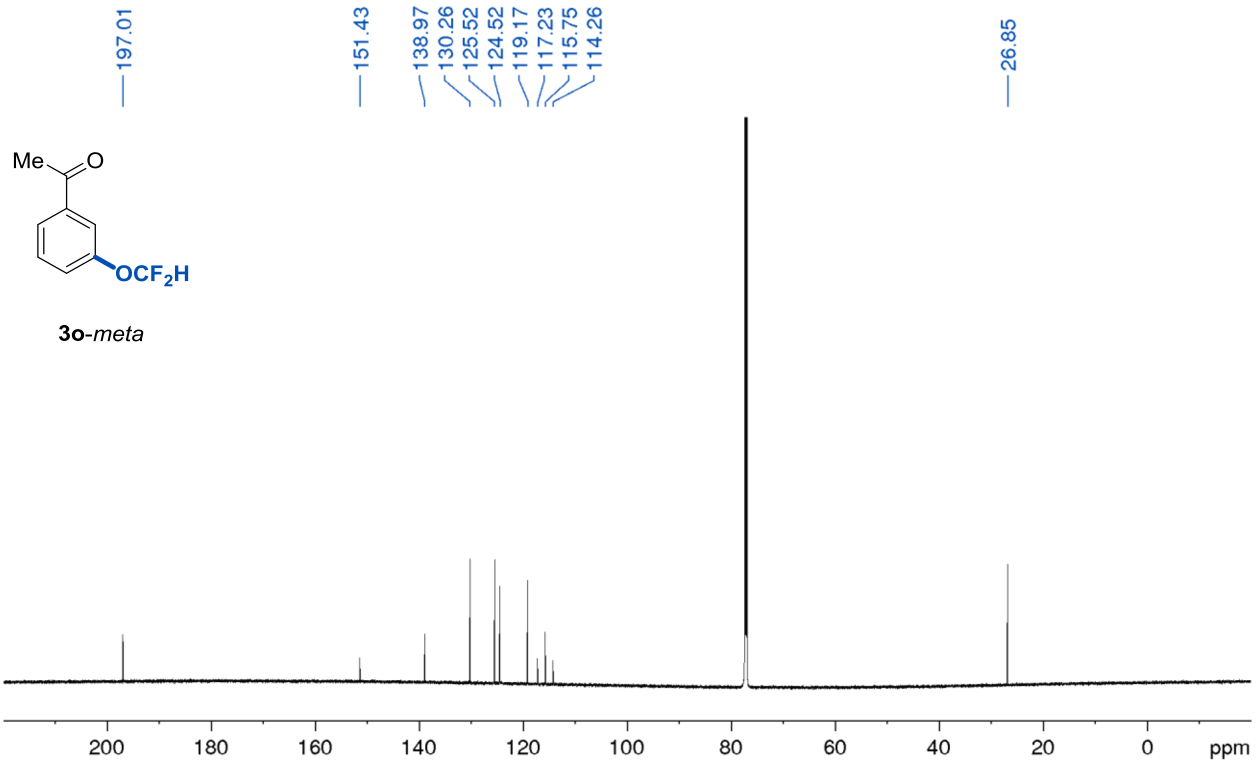

^{19}F NMR (376 MHz, $CDCl_3$, 25 ºC) of 3o-*meta*

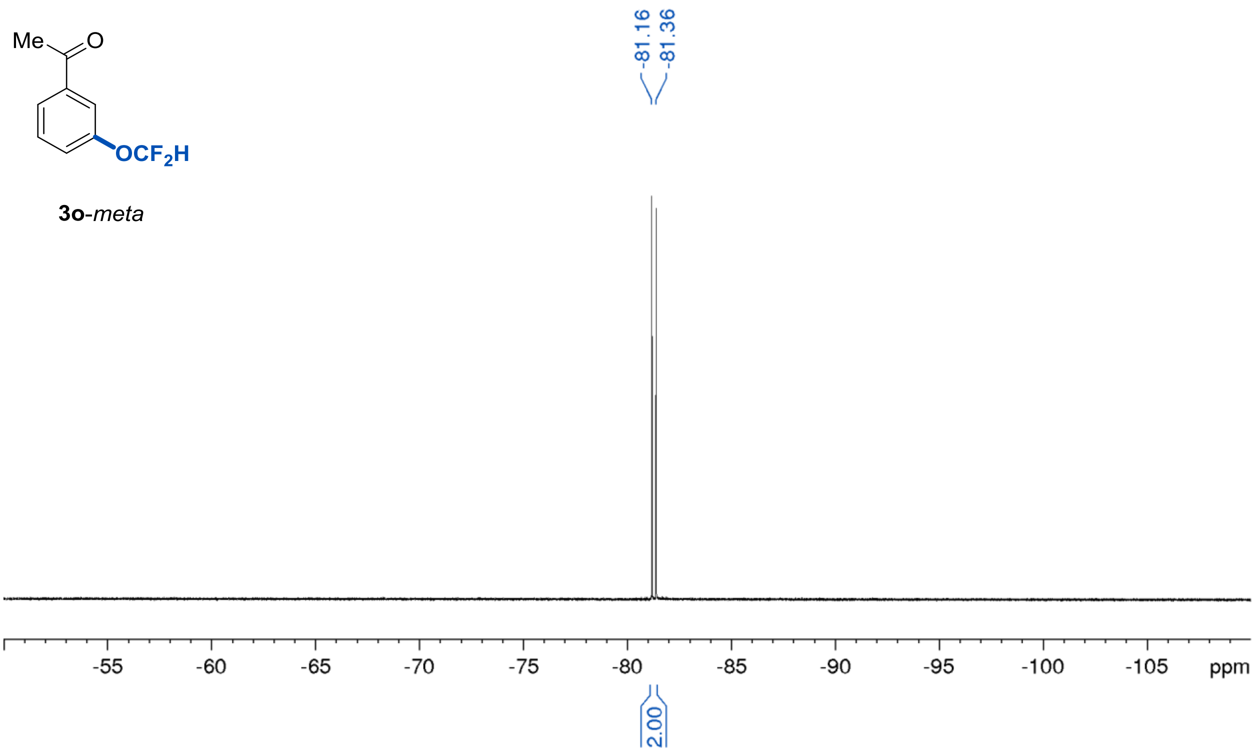

^{1}H NMR (700 MHz, $CDCl_3$, 25 ºC) of 3p-*ortho*

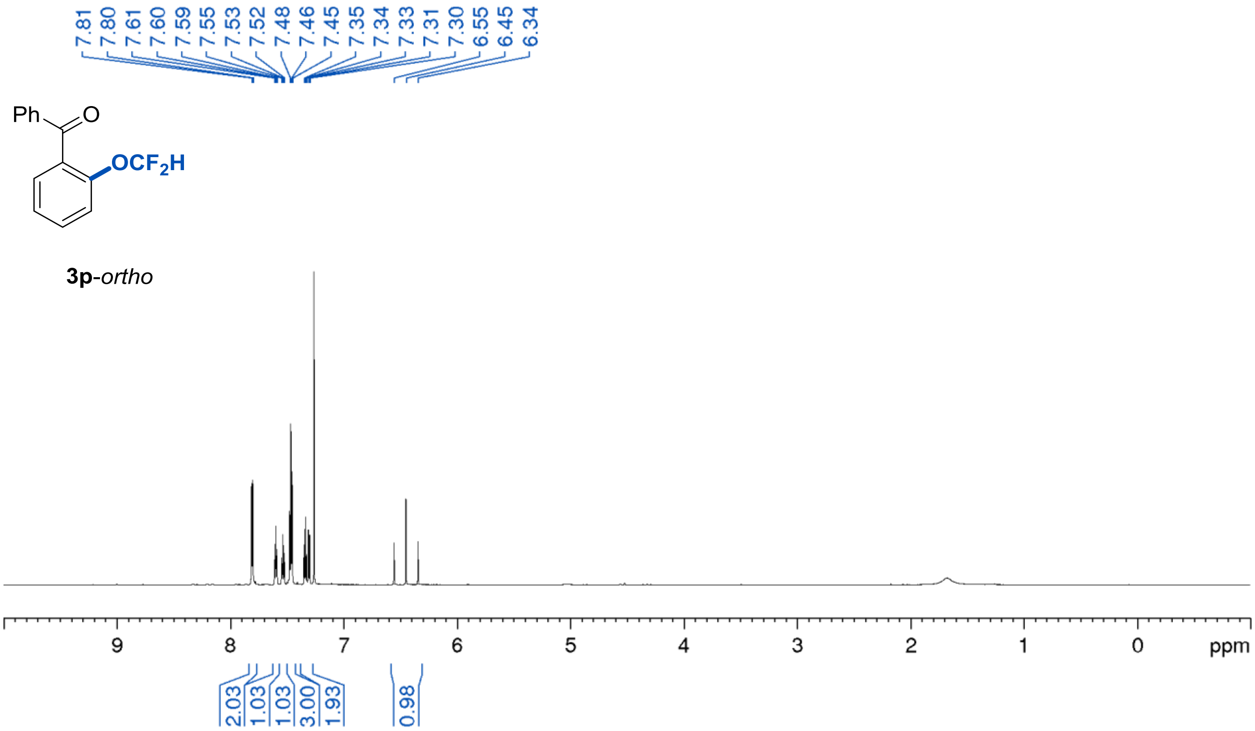

^{13}C NMR (175 MHz, $CDCl_3$, 25 ºC) of 3p-*ortho*

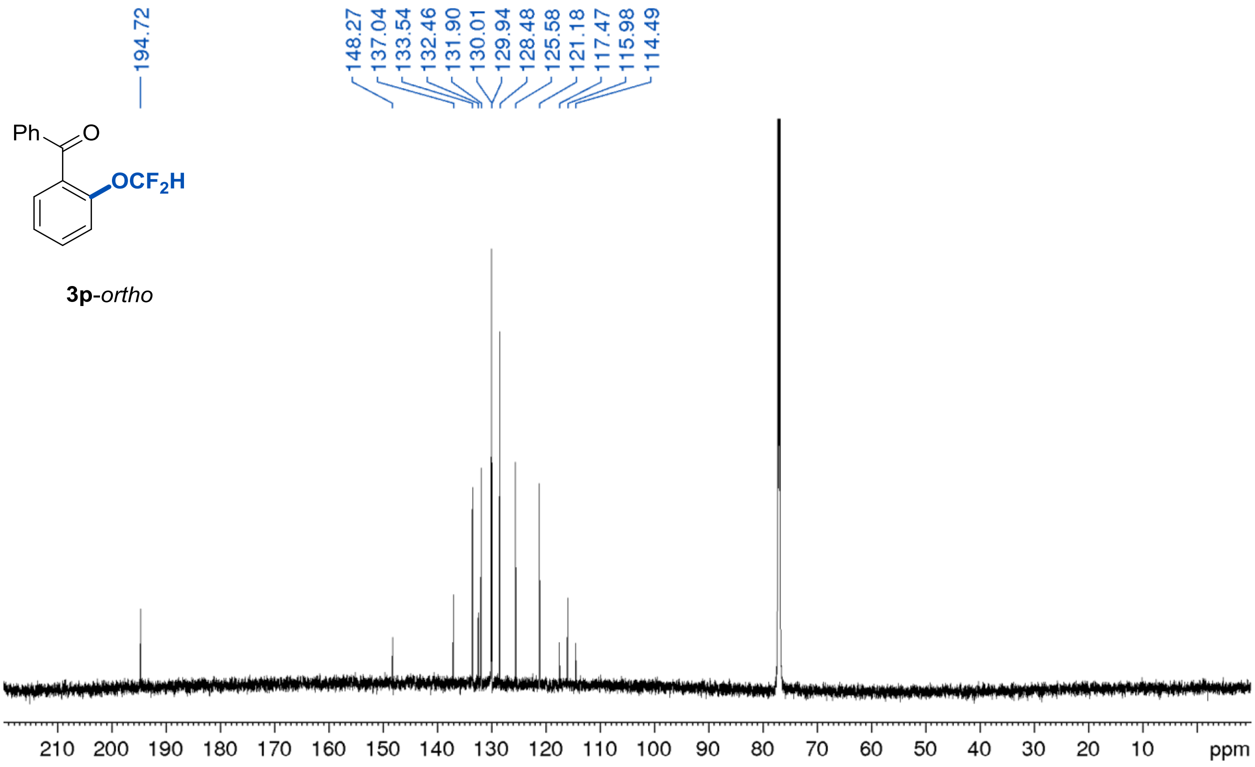

^{19}F NMR (376 MHz, $CDCl_3$, 25 ºC) of 3p-*ortho*

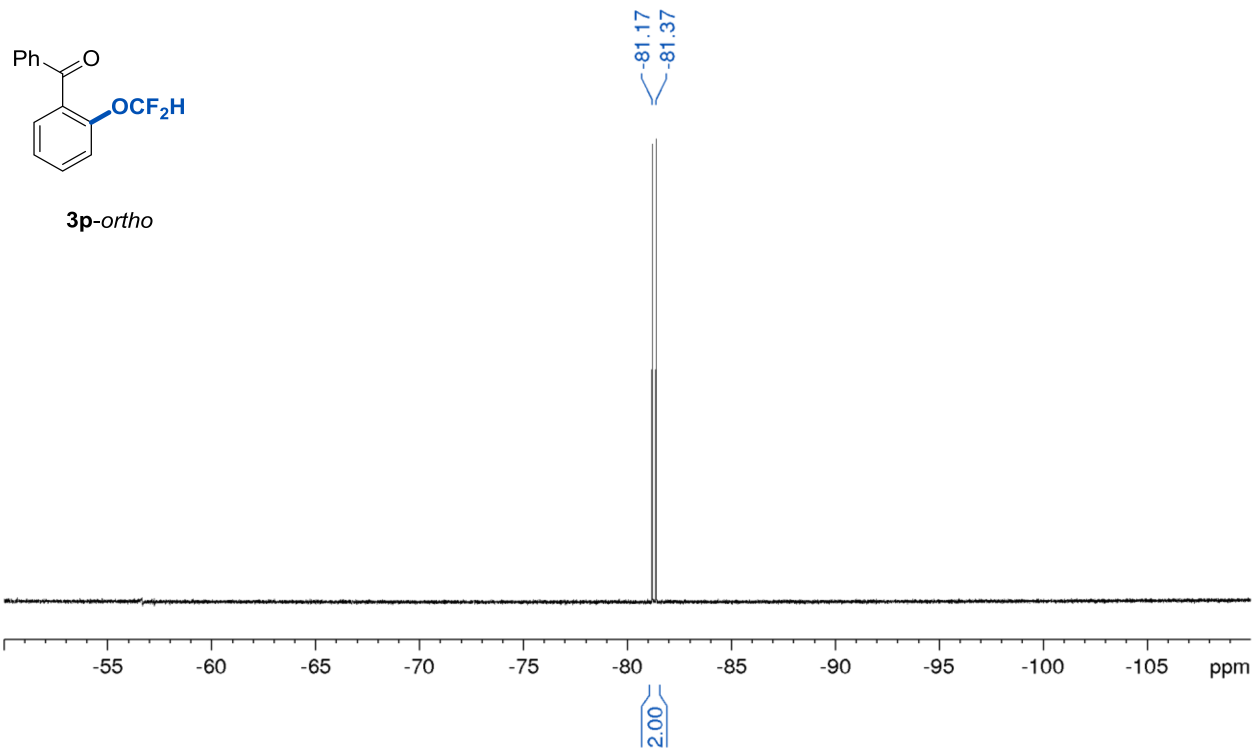

^{1}H NMR (700 MHz, $CDCl_3$, 25 °C) of 3p-*meta* and -*para*

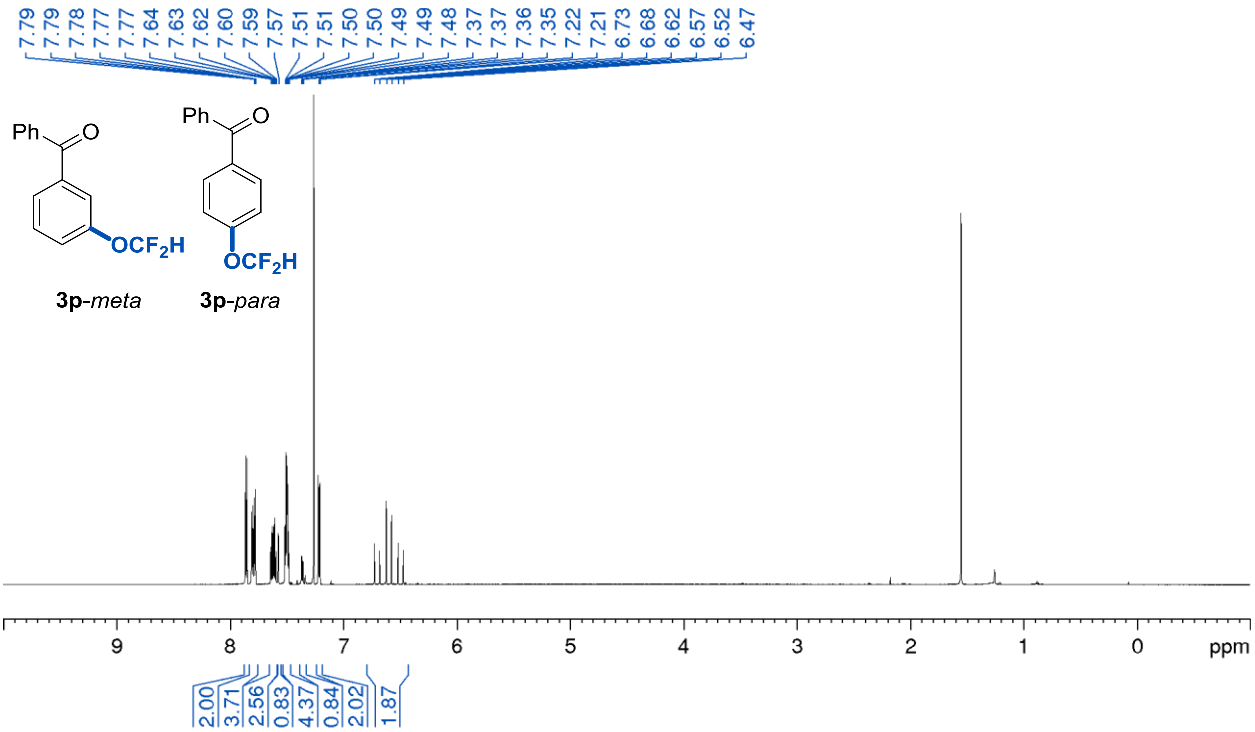

^{13}C NMR (175 MHz, $CDCl_3$, 25 °C) of 3p-*meta* and -*para*

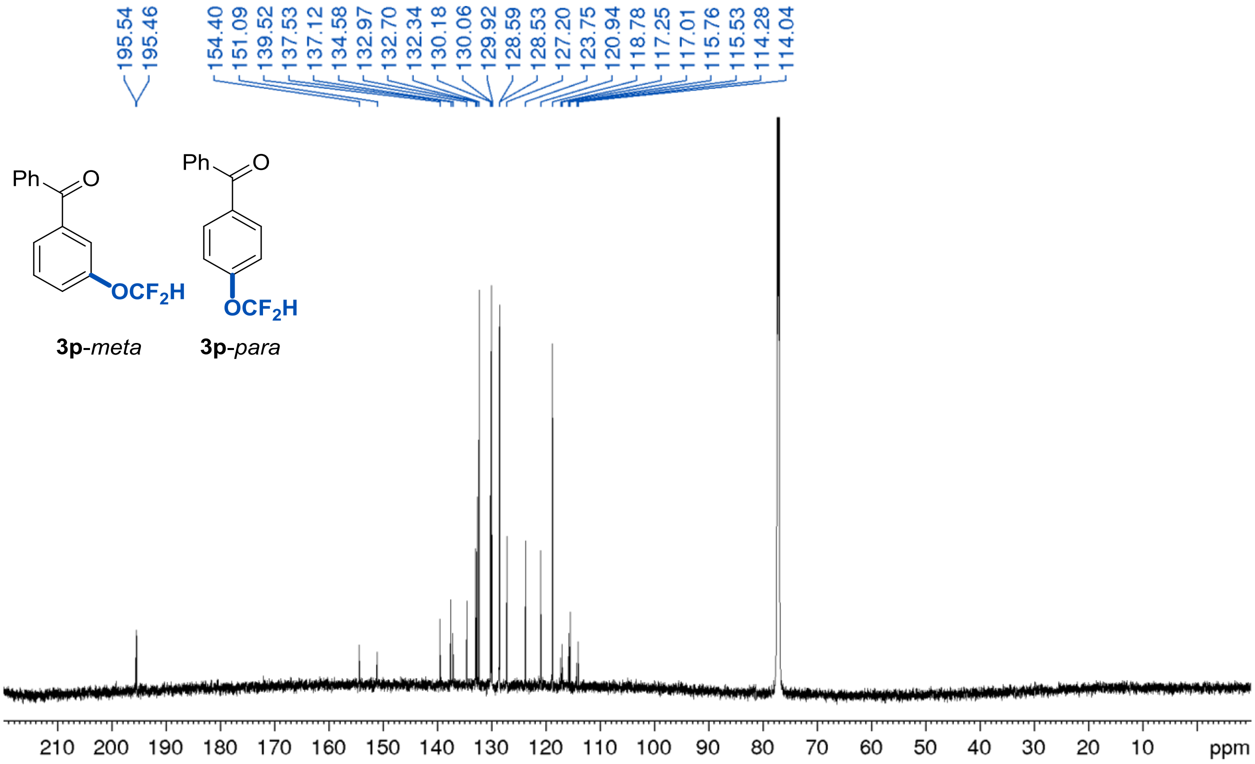

^{19}F NMR (376 MHz, $CDCl_3$, 25 ºC) of 3p-*meta* and -*para*

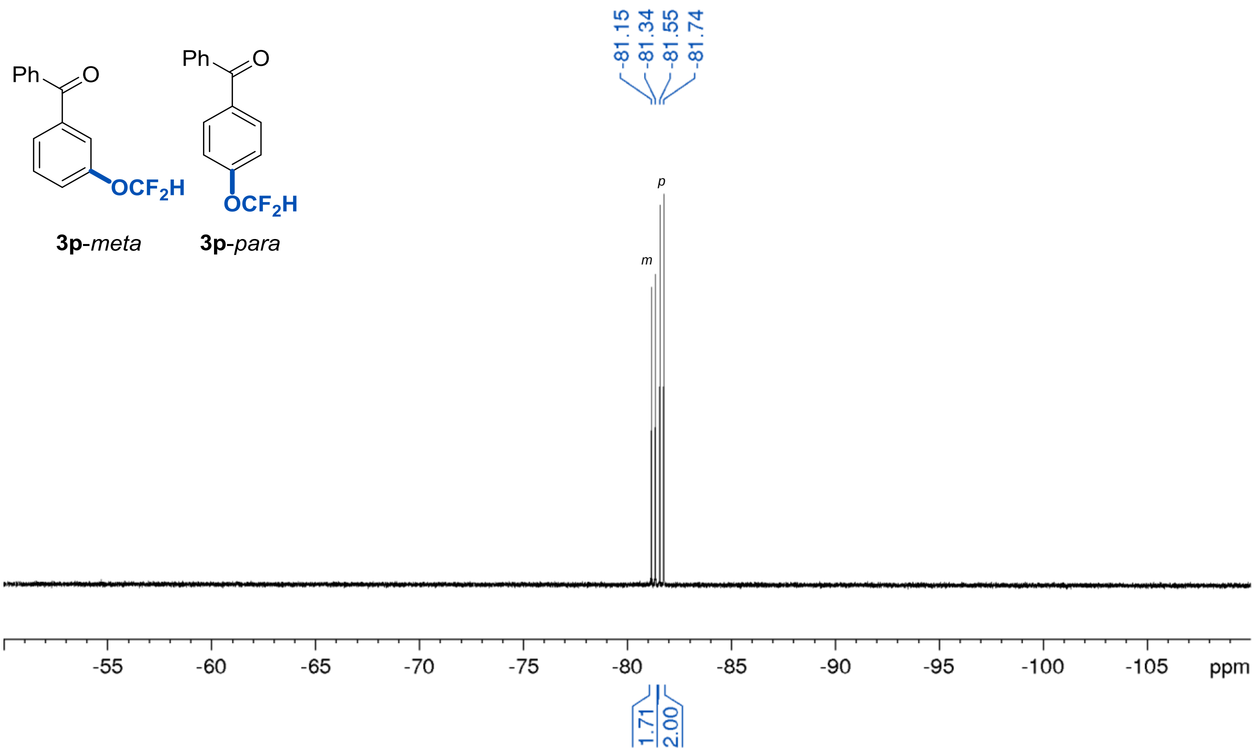

^{1}H NMR (700 MHz, $CDCl_3$, 25 ºC) of 3q

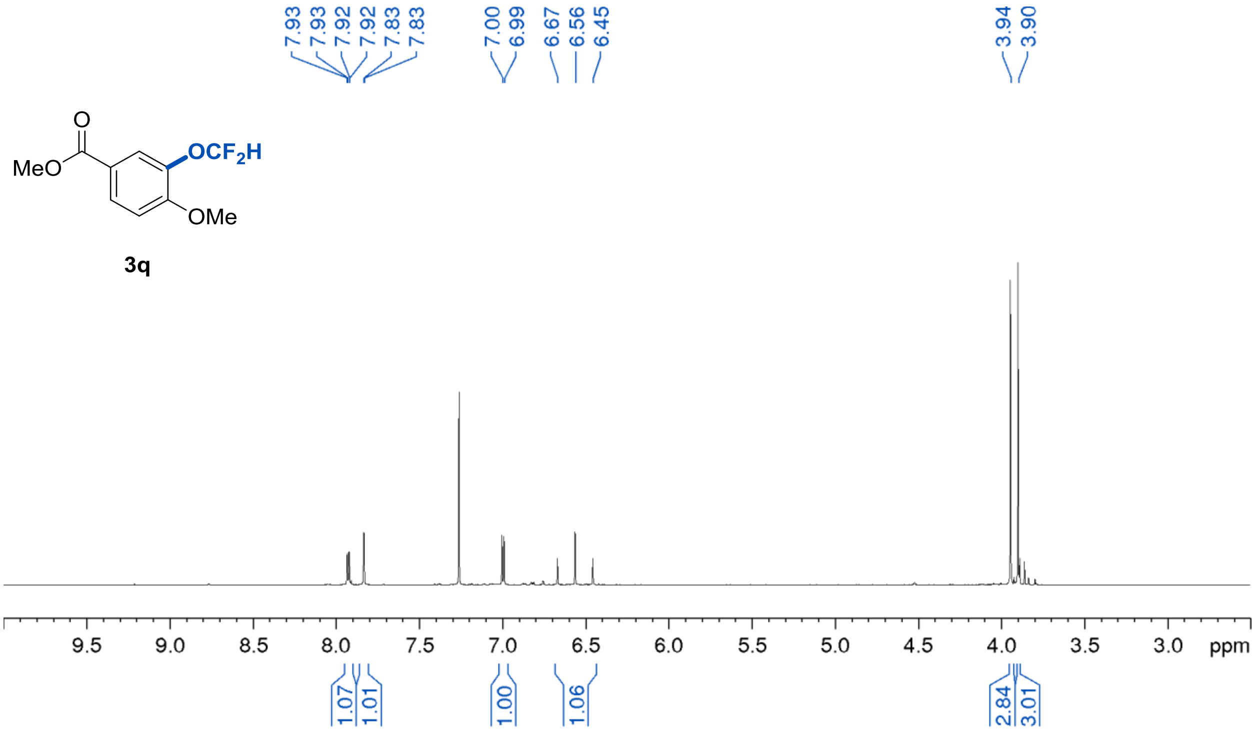

^{13}C NMR (175 MHz, $CDCl_3$, 25 ºC) of 3q

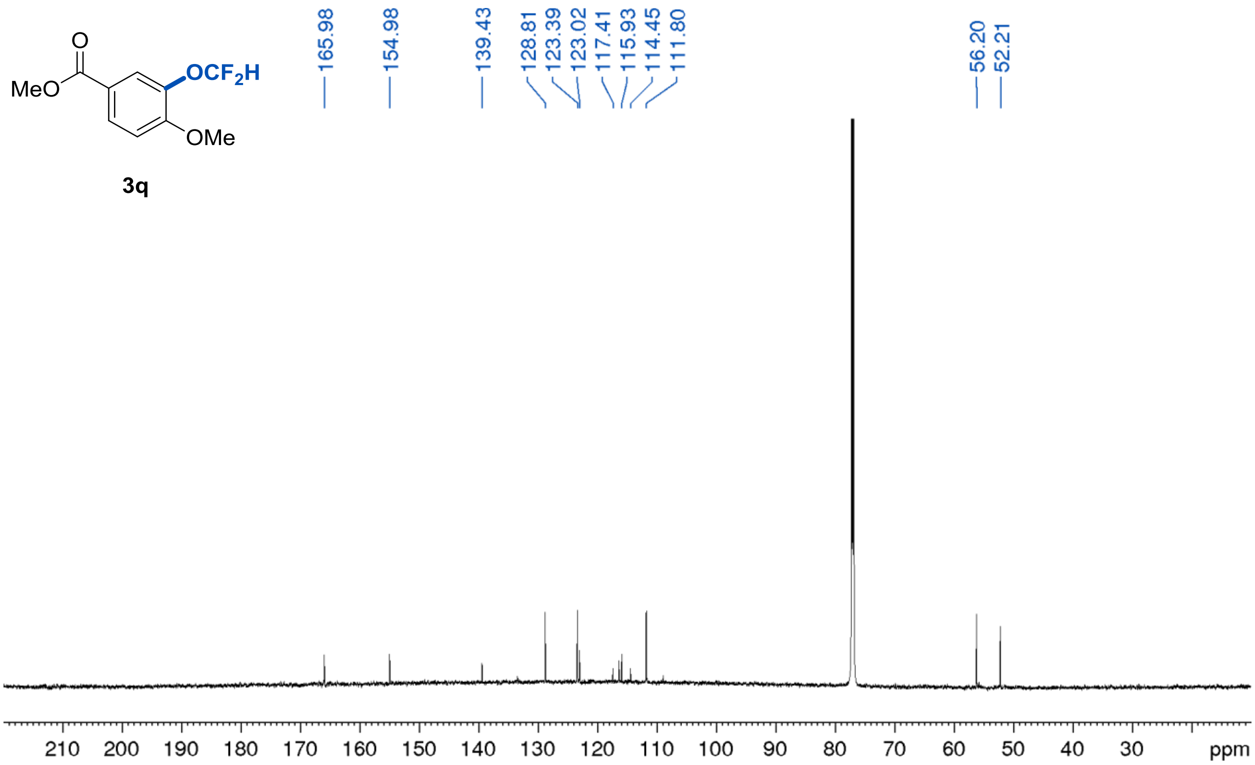

^{19}F NMR (376 MHz, $CDCl_3$, 25 ºC) of 3q

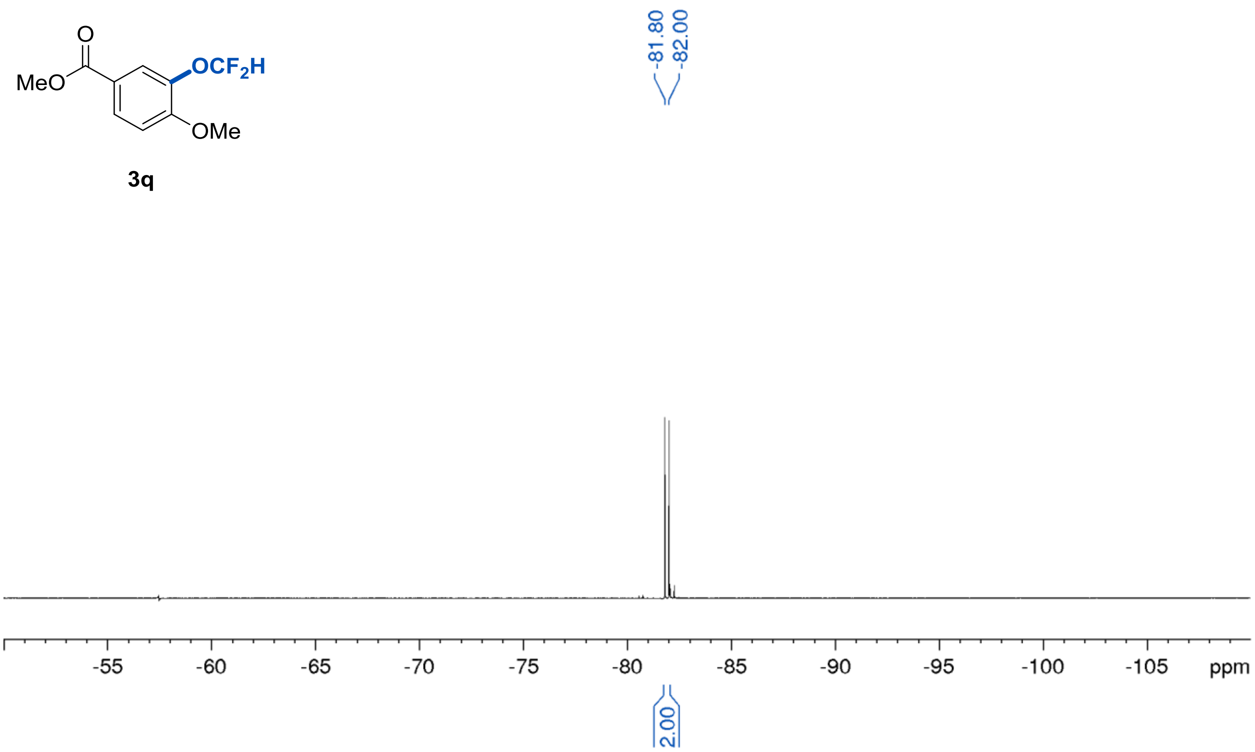

^{1}H NMR (700 MHz, $CDCl_3$, 25 °C) of 3r

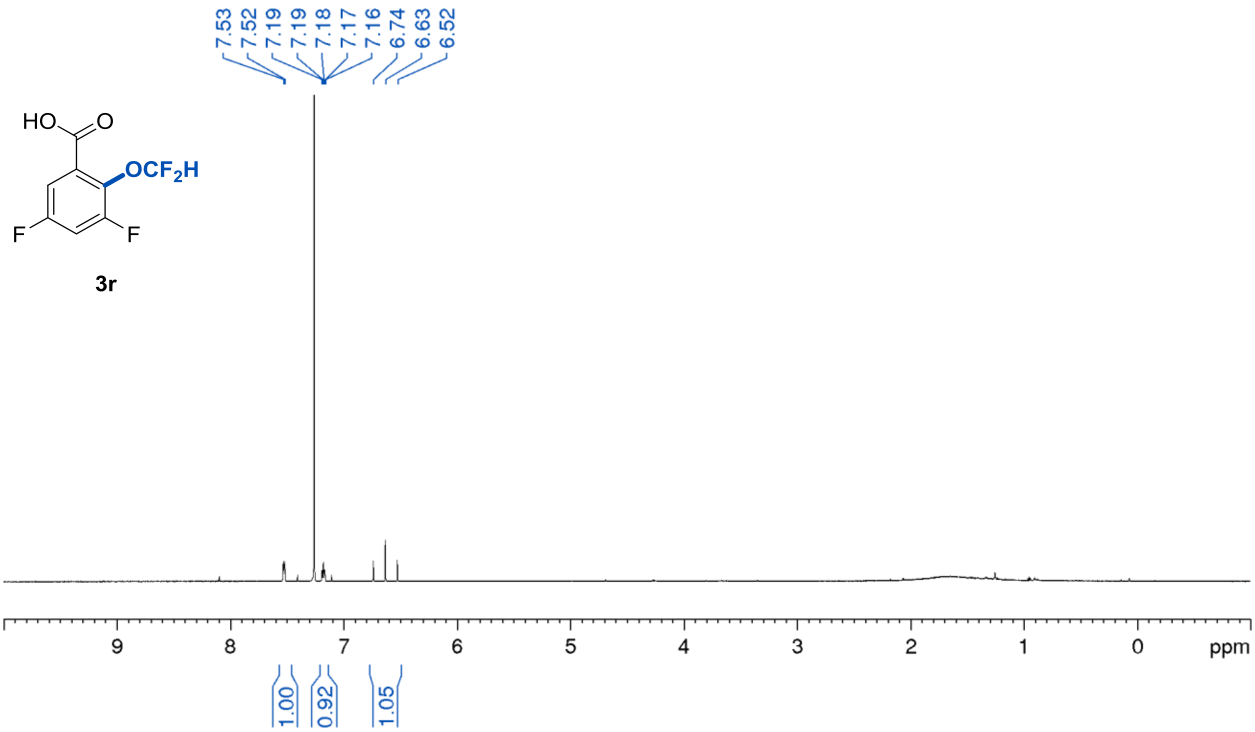

^{19}F NMR (376 MHz, $CDCl_3$, 25 °C) of 3r

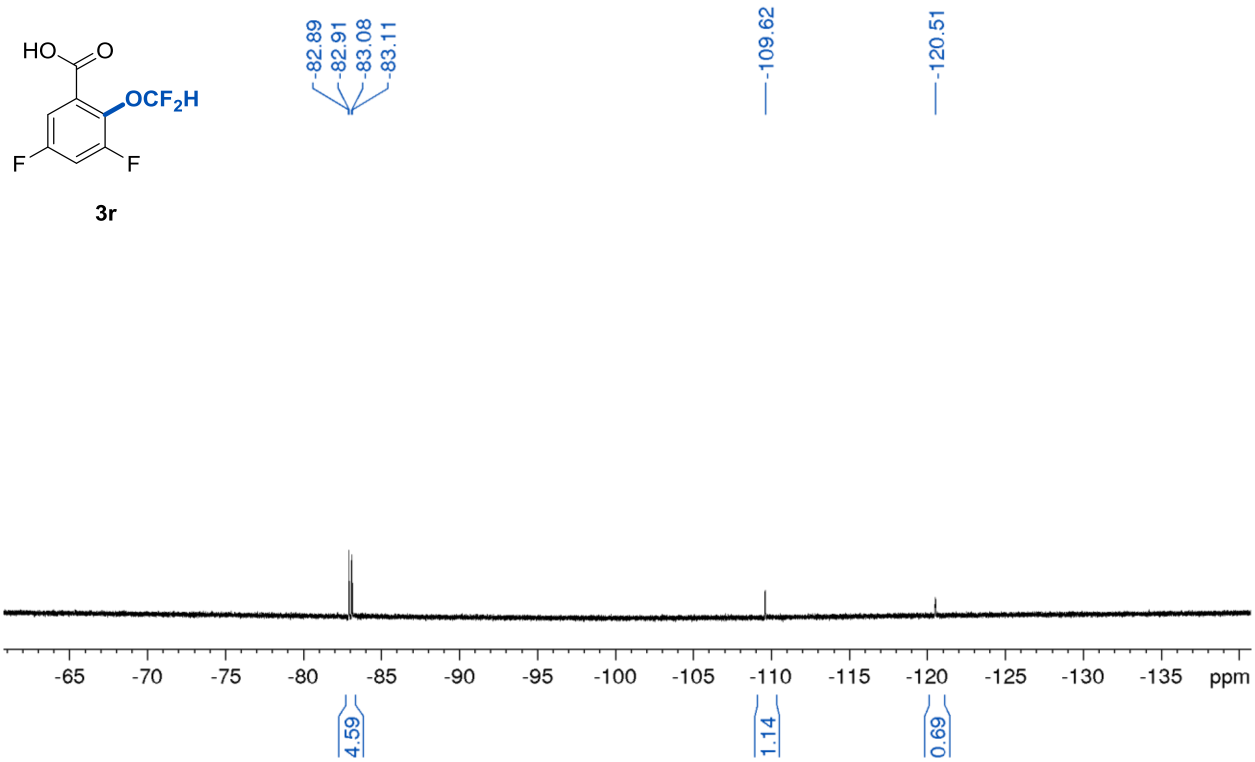

^{1}H NMR (700 MHz, $CDCl_3$, 25 °C) of 3r'

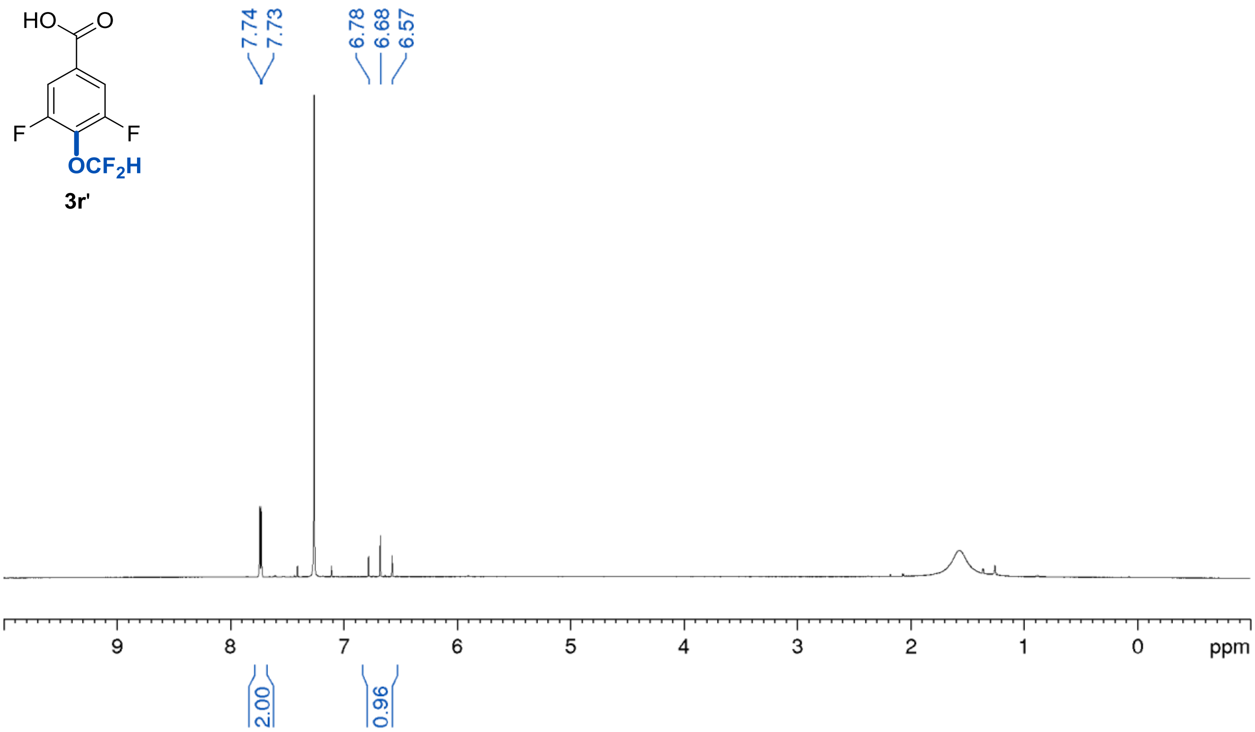

^{19}F NMR (376 MHz, $CDCl_3$, 25 °C) of 3r'

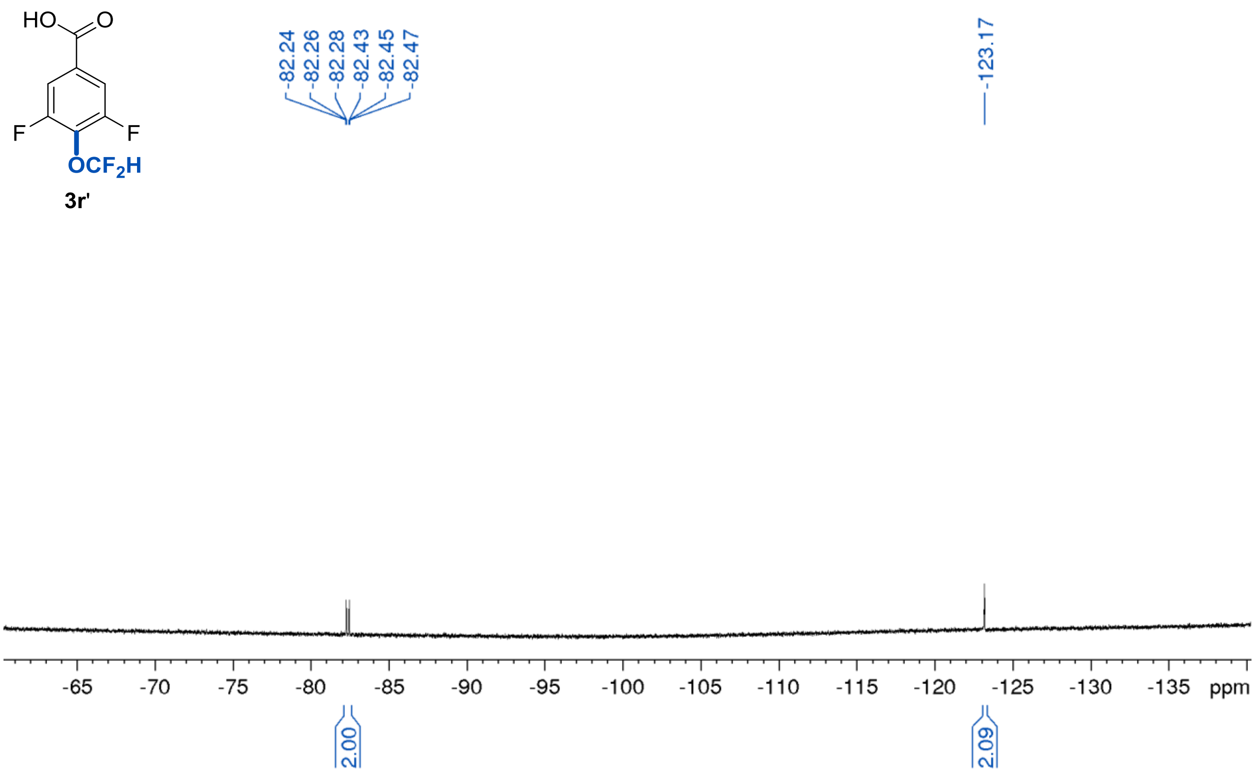

^{1}H NMR (700 MHz, $CDCl_3$, 25 °C) of 3s-*ortho*

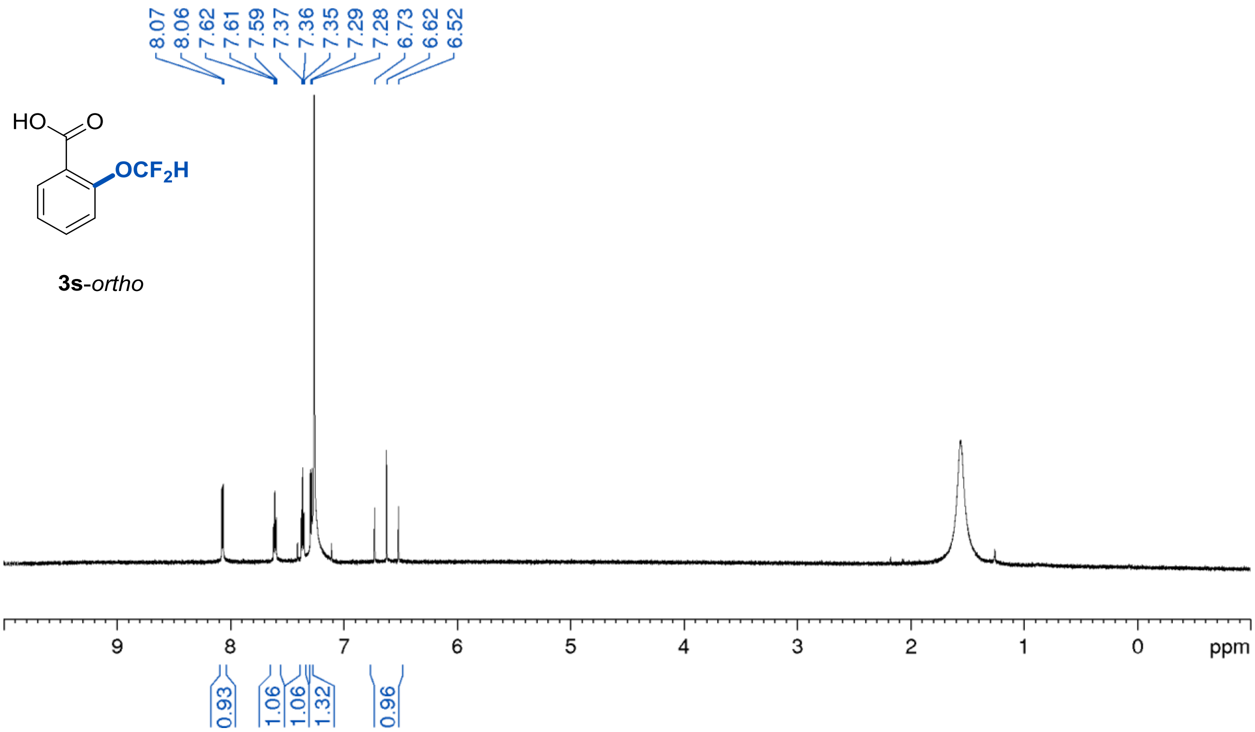

^{13}C NMR (175 MHz, $CDCl_3$, 25 °C) of 3s-*ortho*

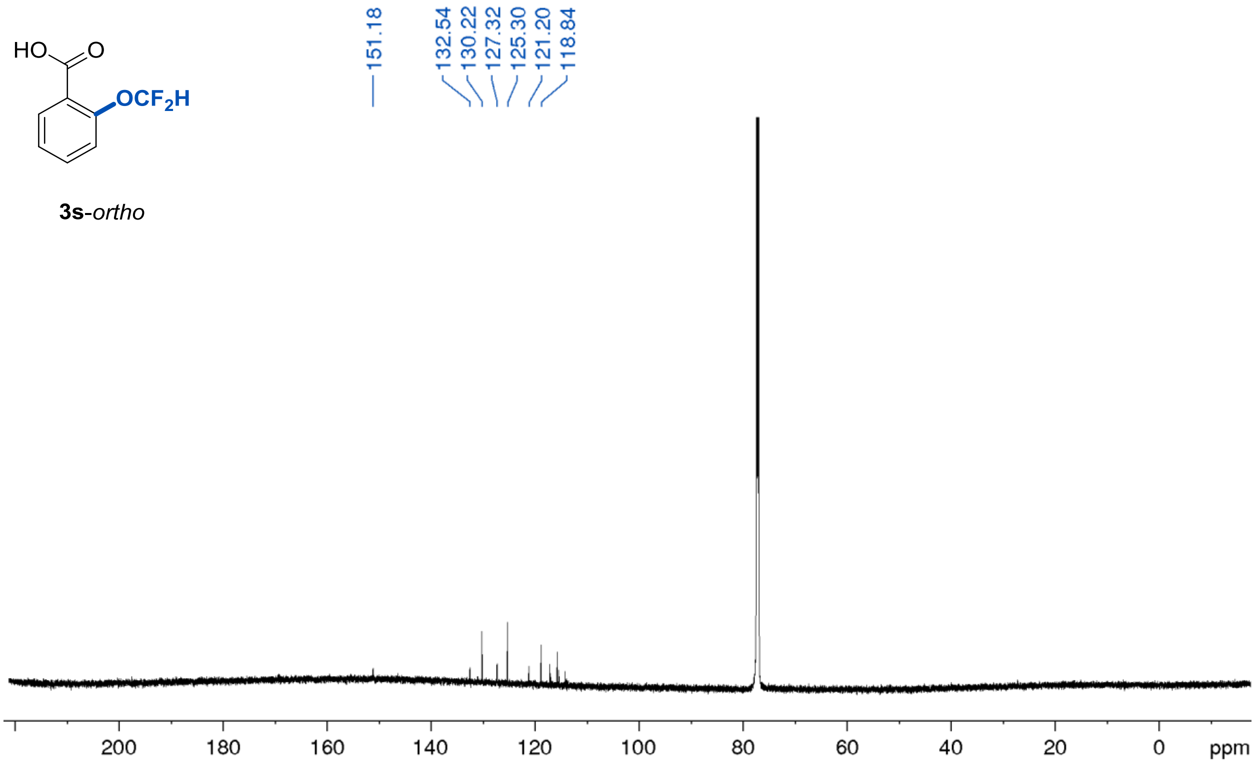

^{19}F NMR (376 MHz, CDCl₃, 25 ºC) of 3s-*ortho*

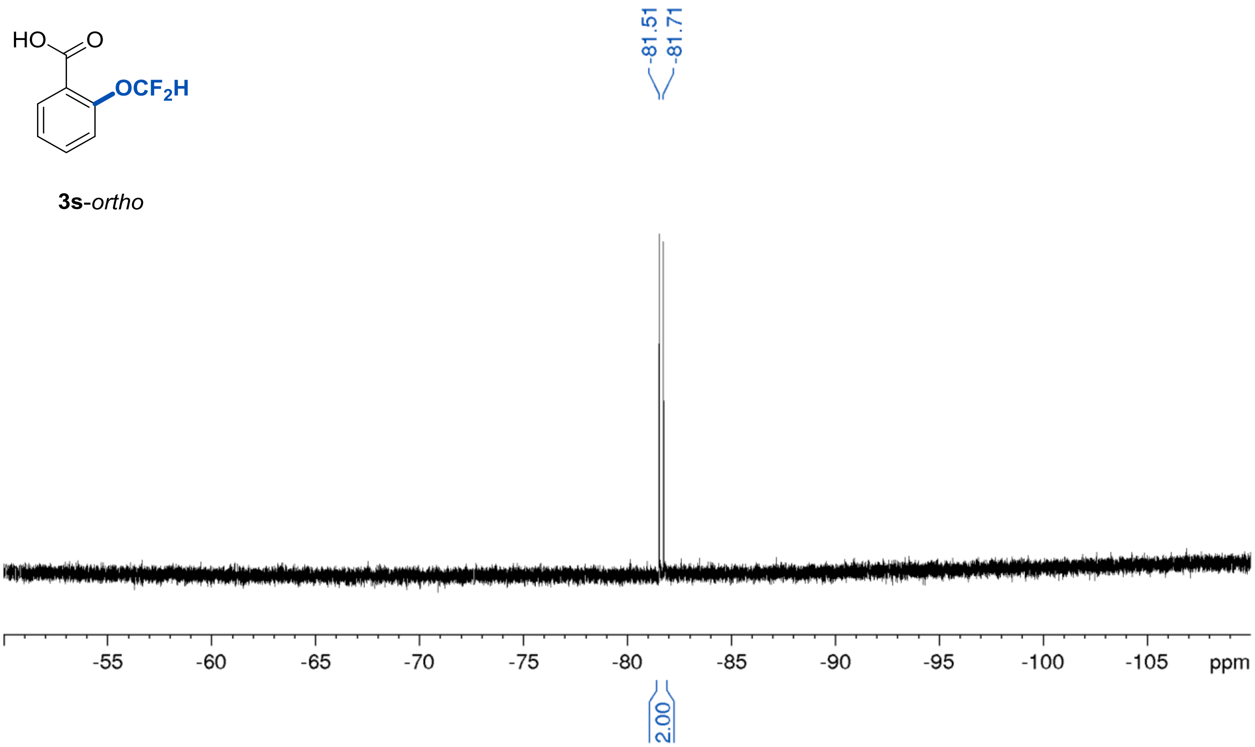

^{1}H NMR (700 MHz, CDCl₃, 25 ºC) of 3s-*meta* and -*para*

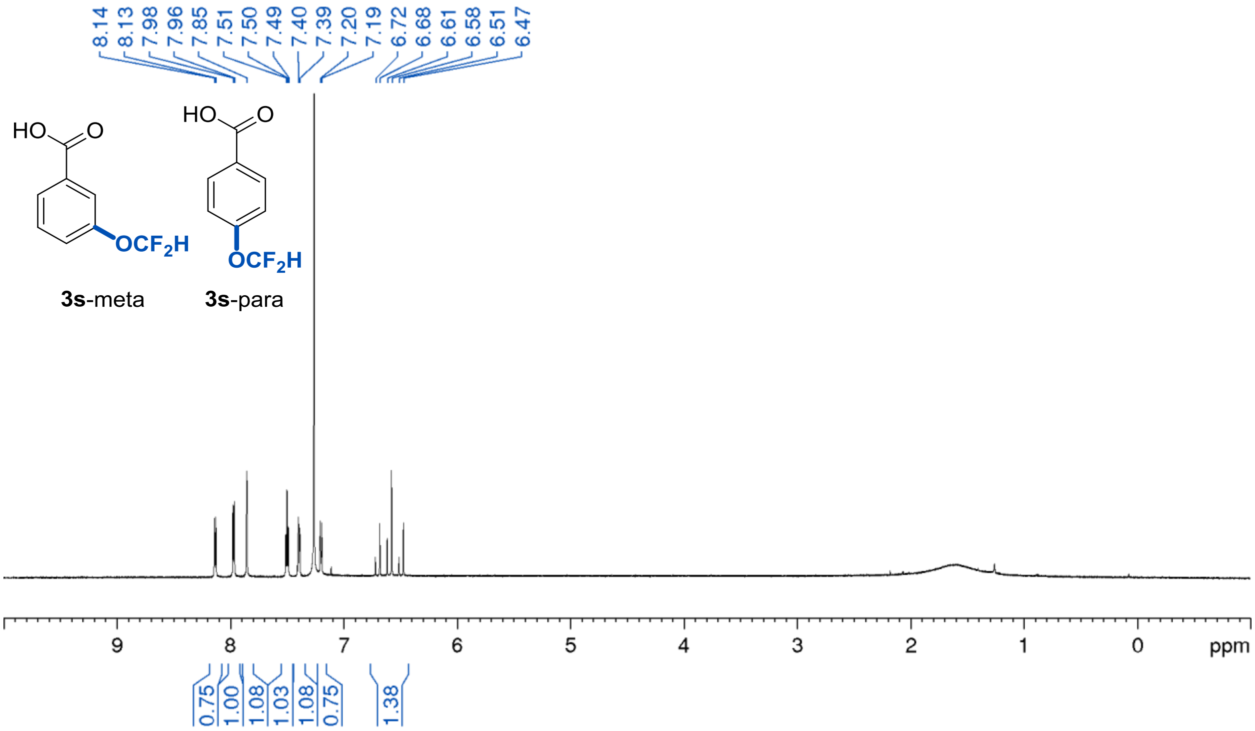

^{13}C NMR (175 MHz, $CDCl_3$, 25 ºC) of 3s-*meta* and -*para*

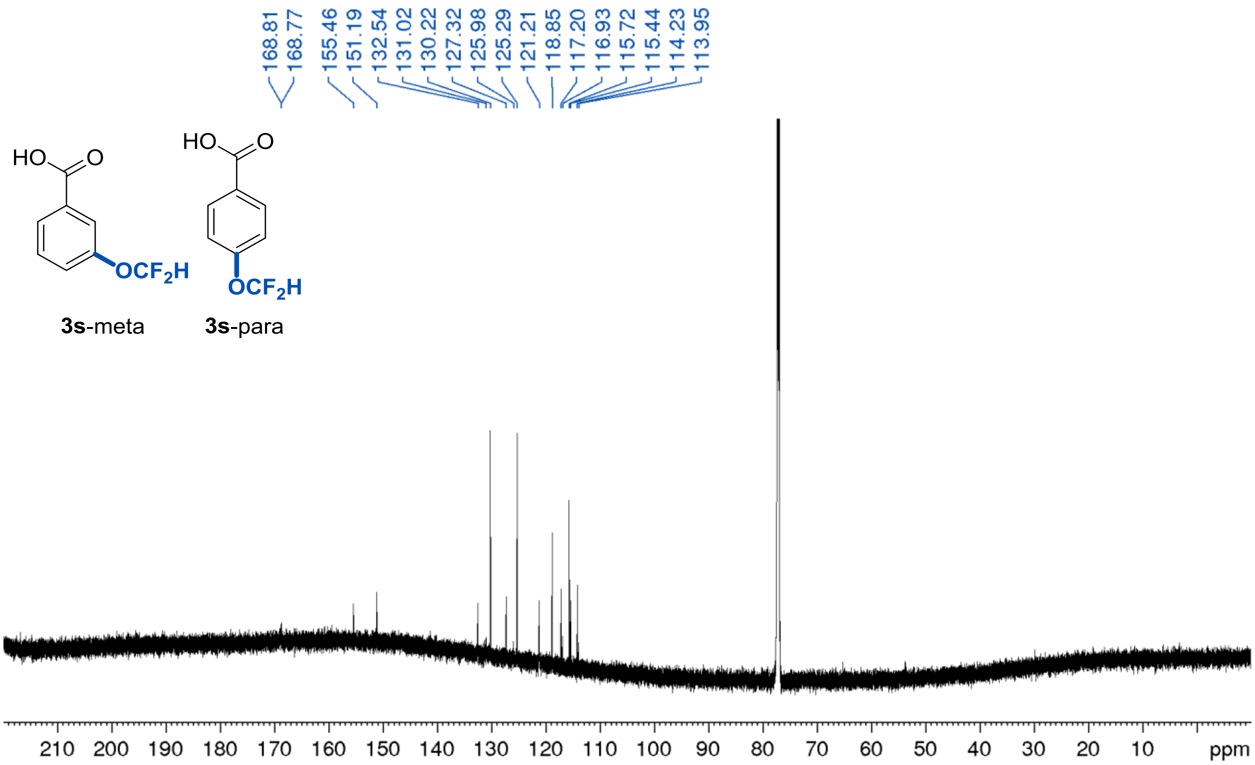

^{19}F NMR (376 MHz, $CDCl_3$, 25 ºC) of 3s-*meta* and -*para*

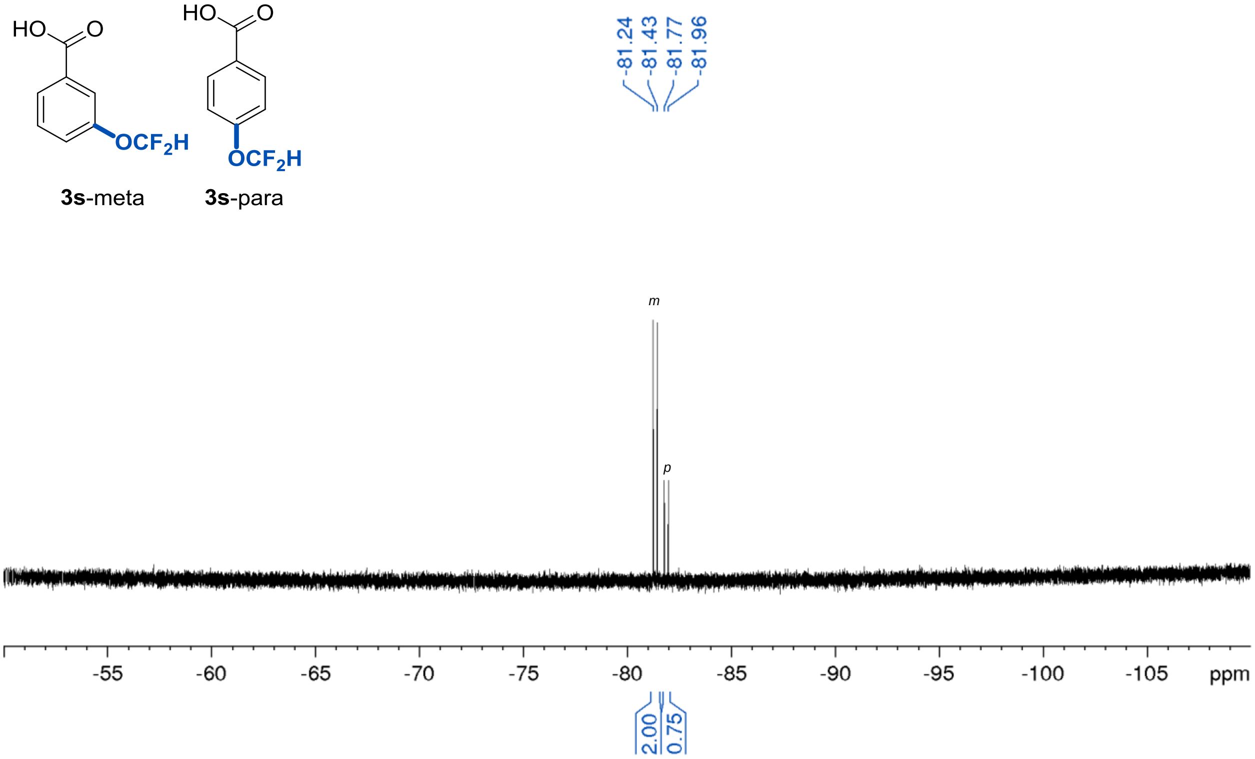

^{1}H NMR (700 MHz, $CDCl_3$, 25 °C) of 3t

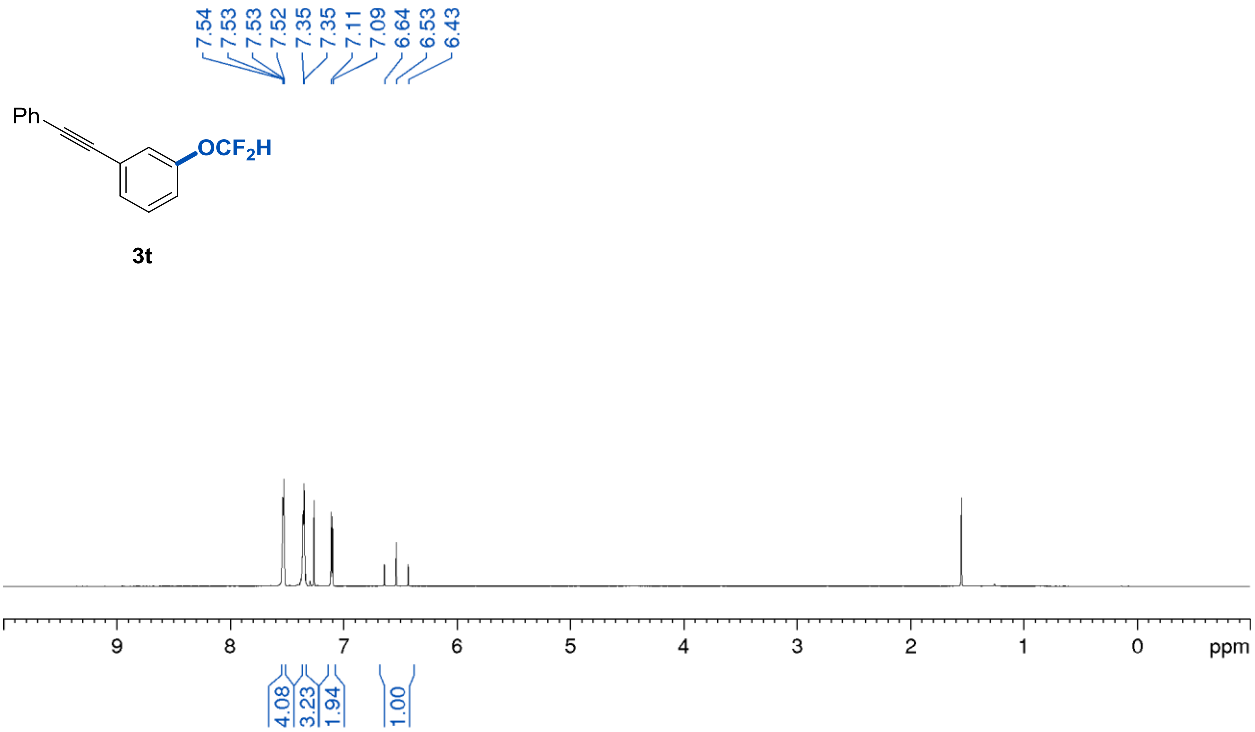

^{13}C NMR (175 MHz, $CDCl_3$, 25 °C) of 3t

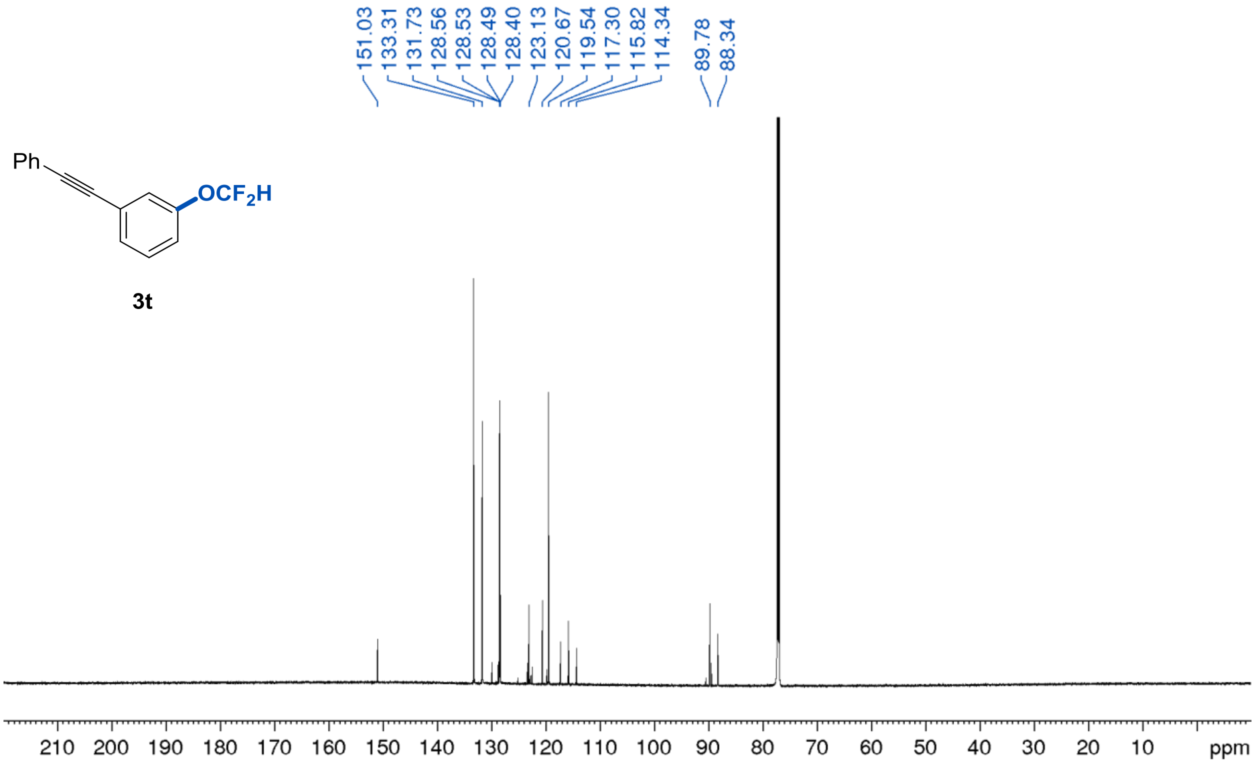

^{19}F NMR (376 MHz, $CDCl_3$, 25 ºC) of 3t

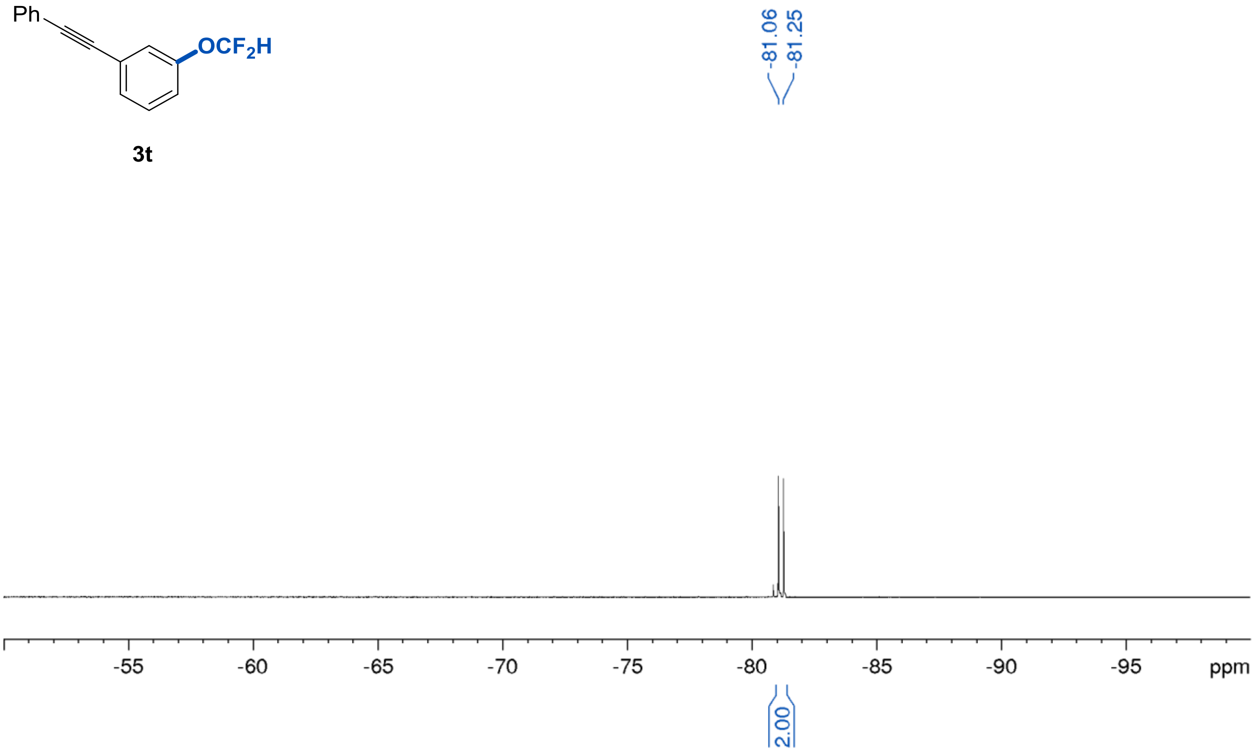

^{1}H NMR (700 MHz, $CDCl_3$, 25 ºC) of 3t'

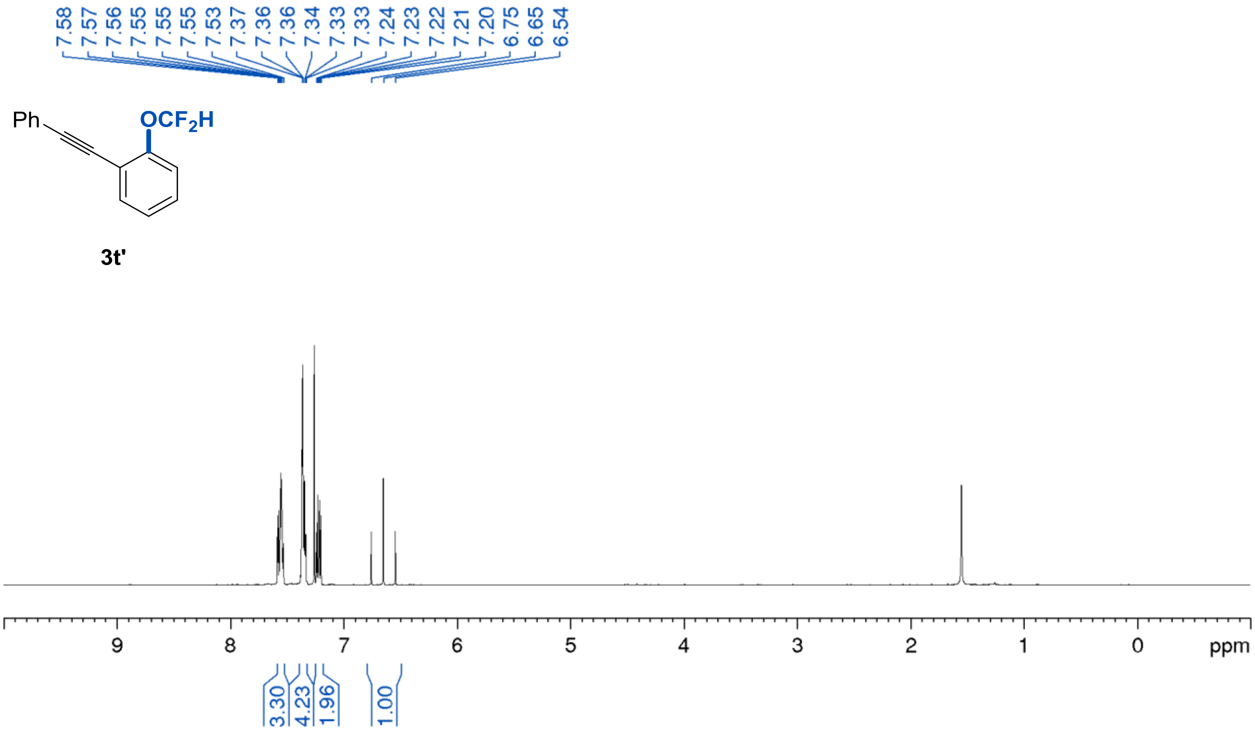

^{13}C NMR (175 MHz, CDCl$_3$, 25 ºC) of 3t’

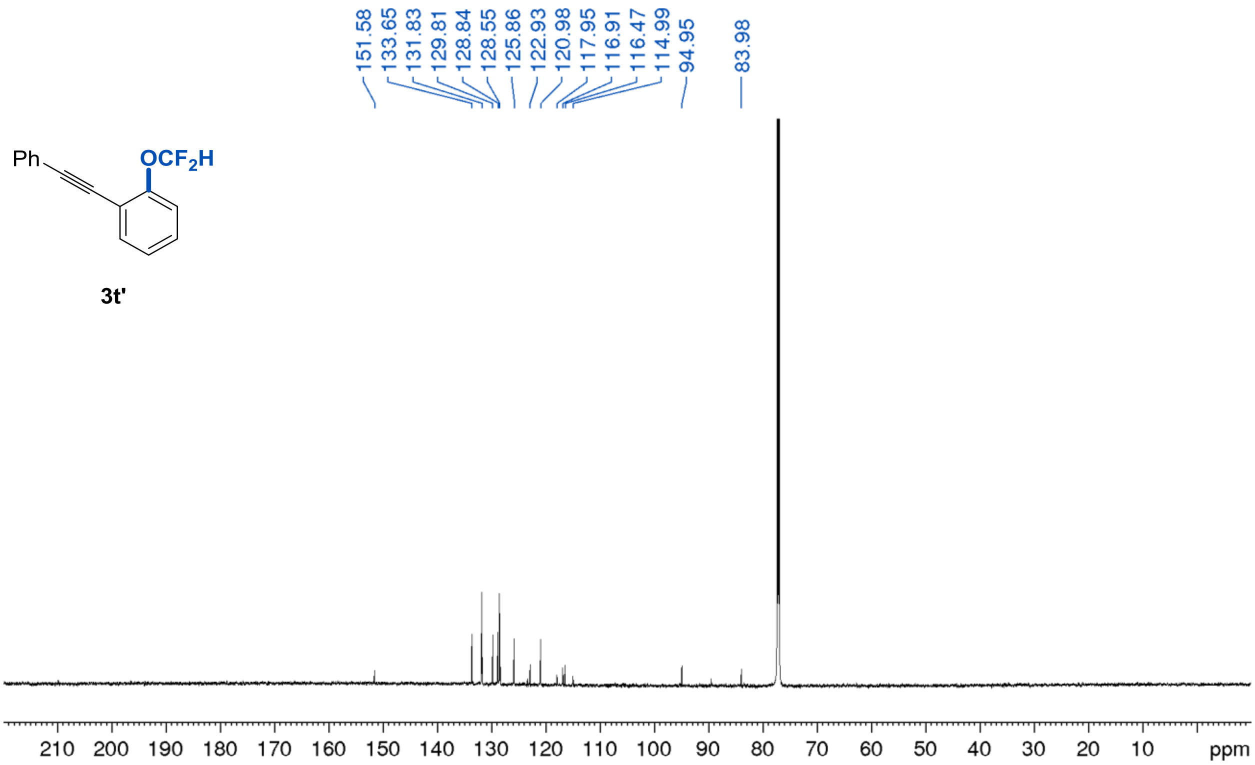

^{19}F NMR (376 MHz, CDCl$_3$, 25 ºC) of 3t’

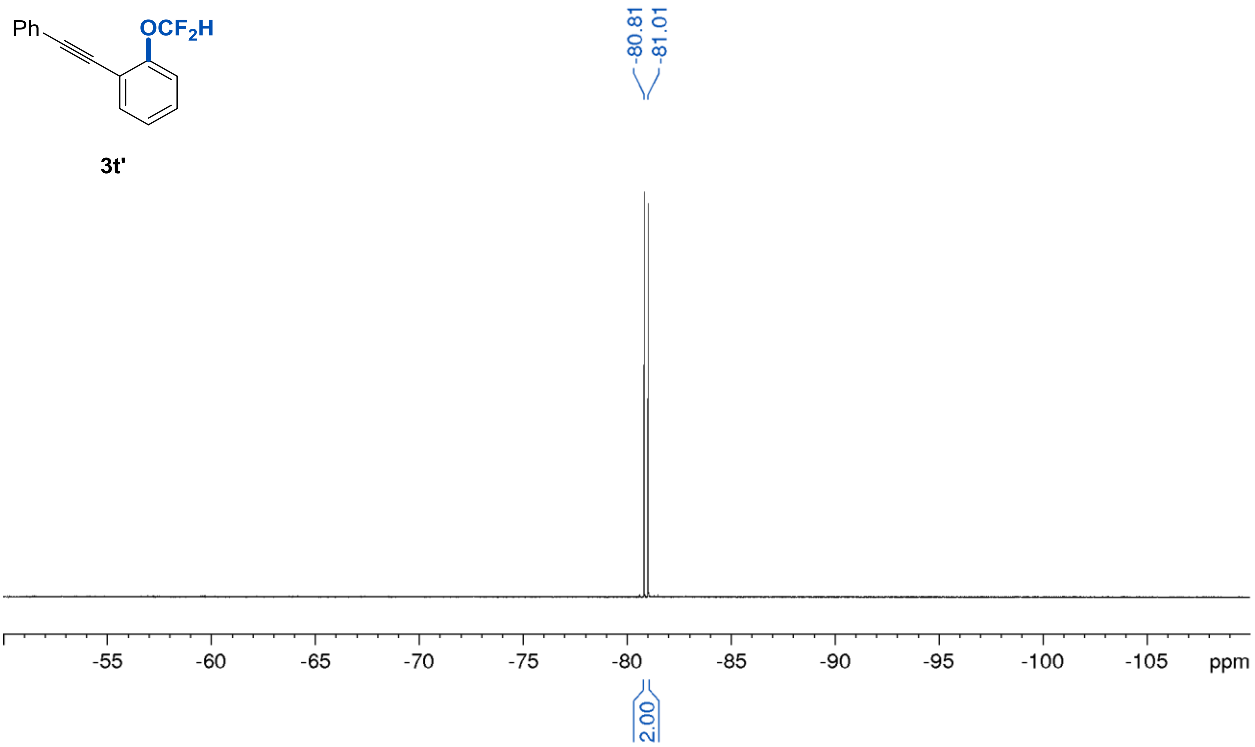

^{1}H NMR (700 MHz, $CDCl_3$, 25 °C) of 3u

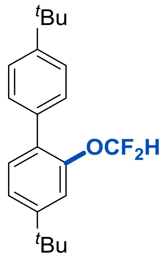

3u

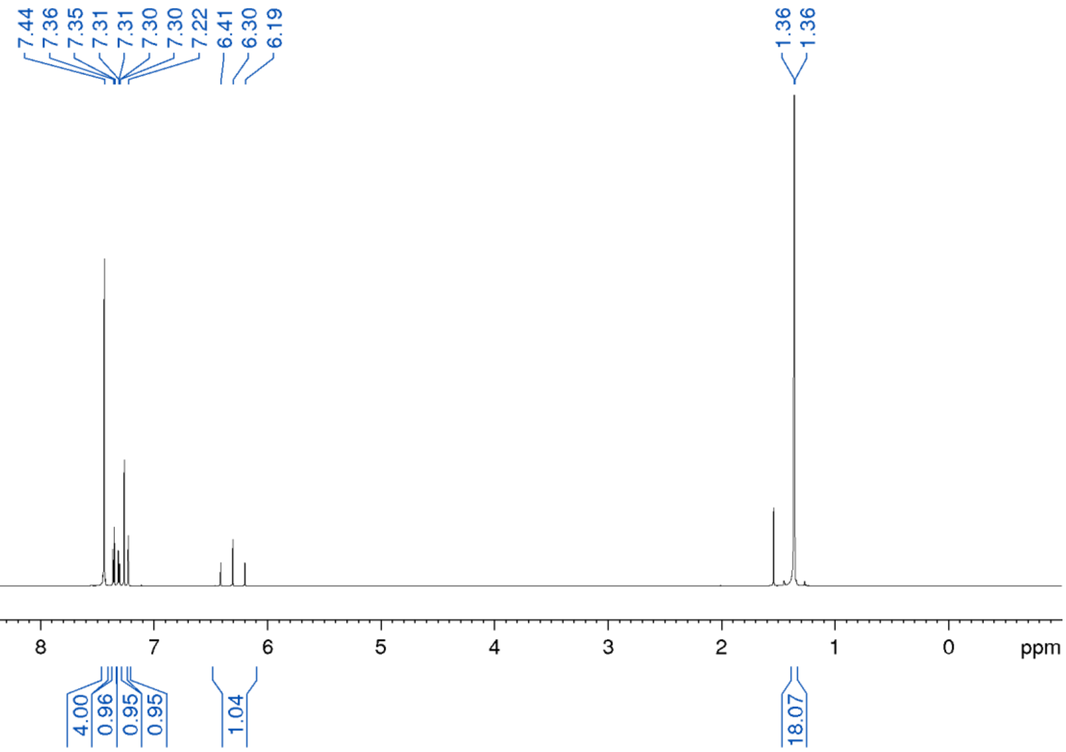

^{13}C NMR (175 MHz, $CDCl_3$, 25 °C) of 3u

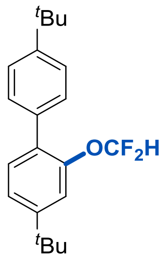

3u

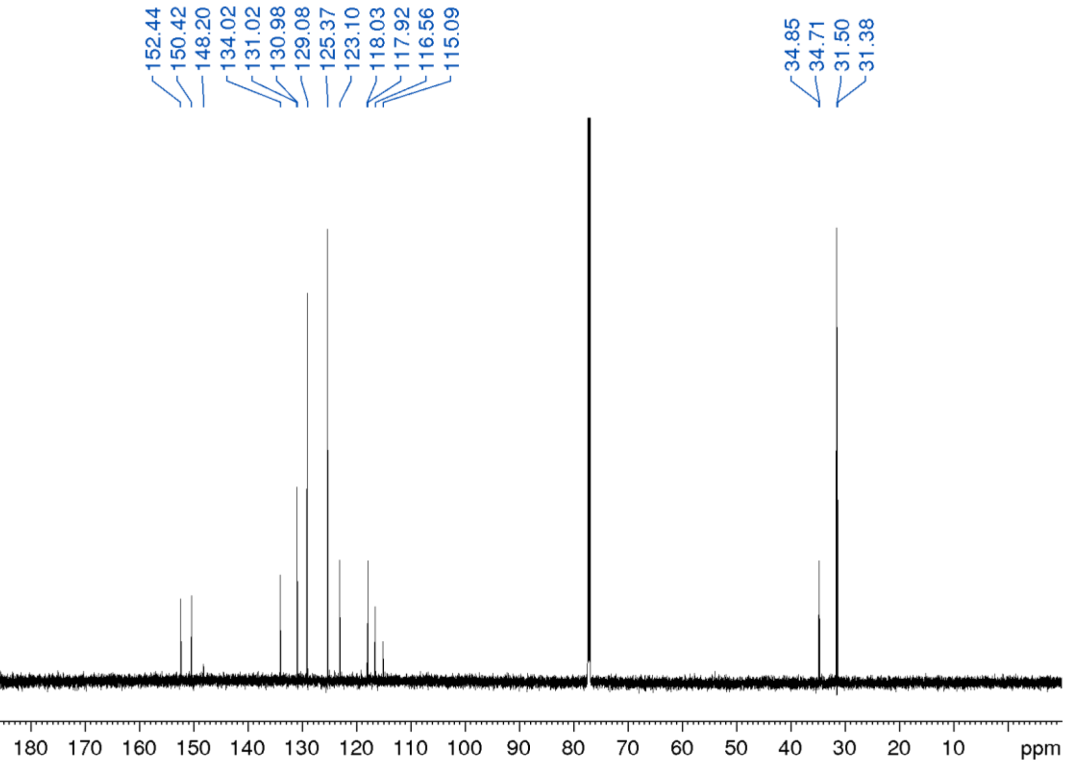

^{19}F NMR (376 MHz, $CDCl_3$, 25 ºC) of 3u

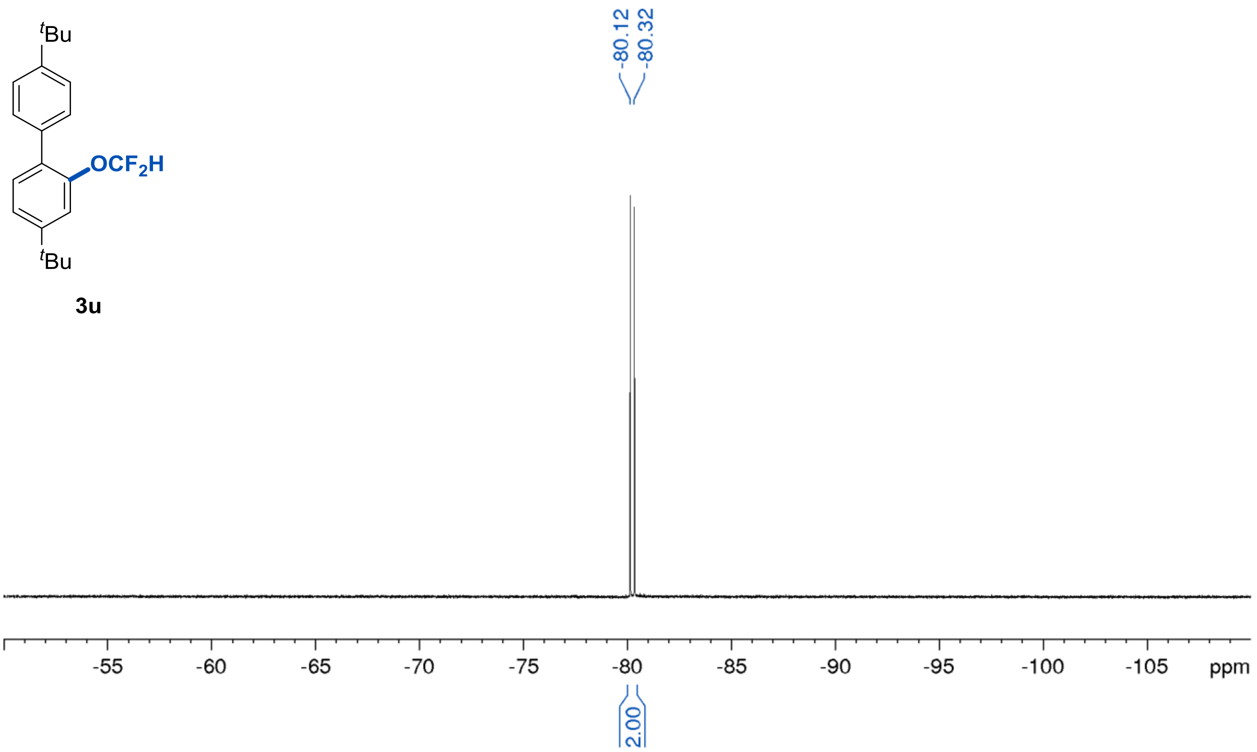

^{1}H NMR (700 MHz, $CDCl_3$, 25 ºC) of 3u'

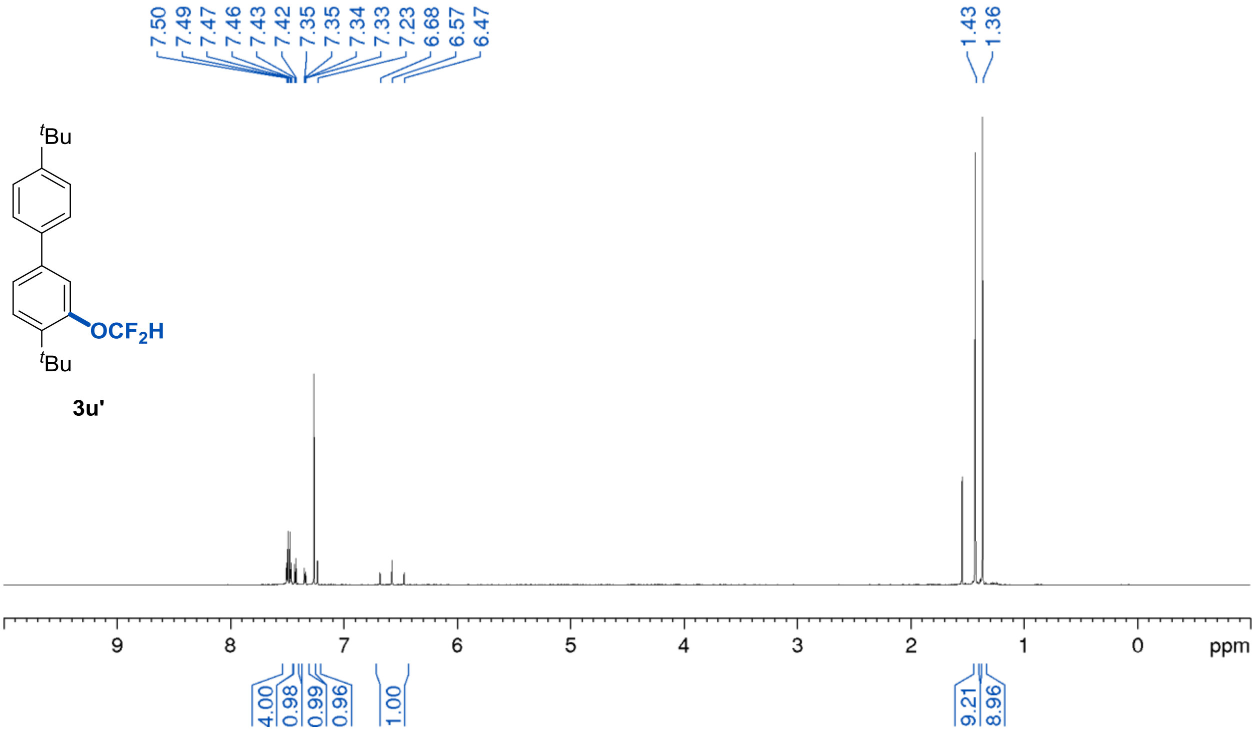

^{13}C NMR (175 MHz, $CDCl_3$, 25 ºC) of 3u’

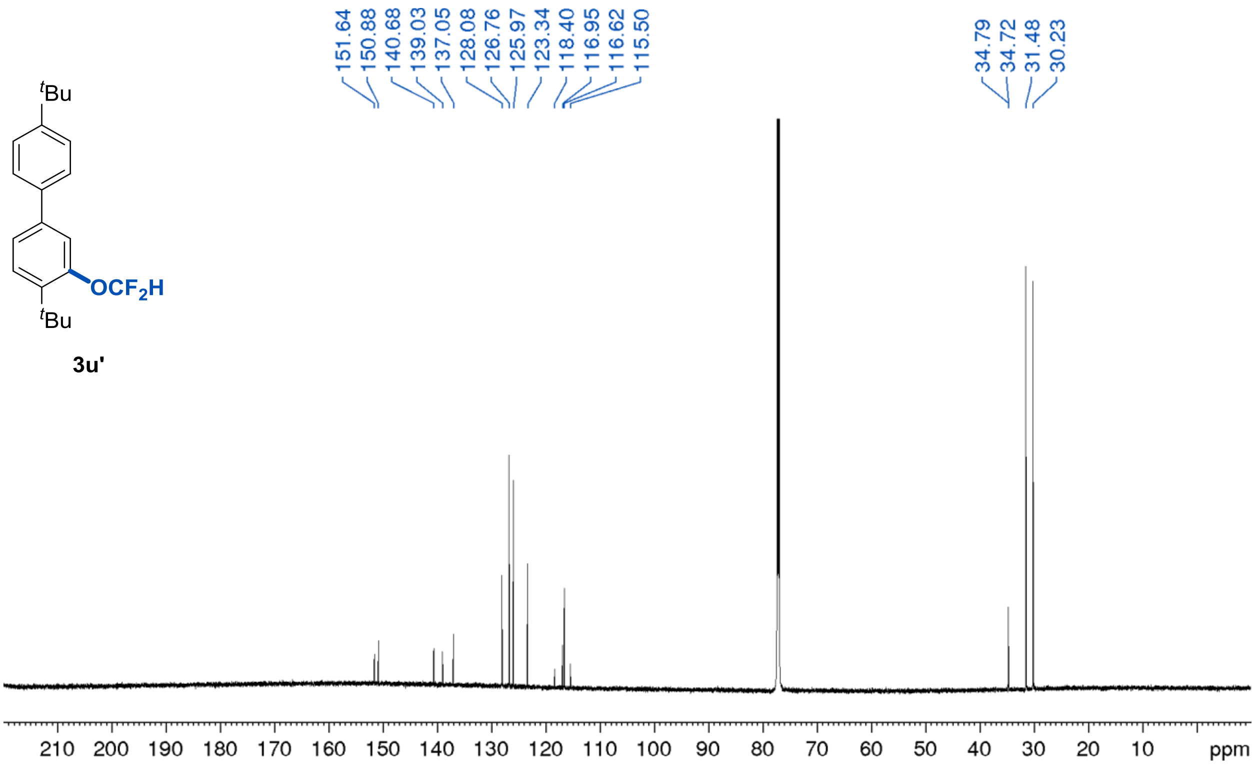

^{19}F NMR (376 MHz, $CDCl_3$, 25 ºC) of 3u’

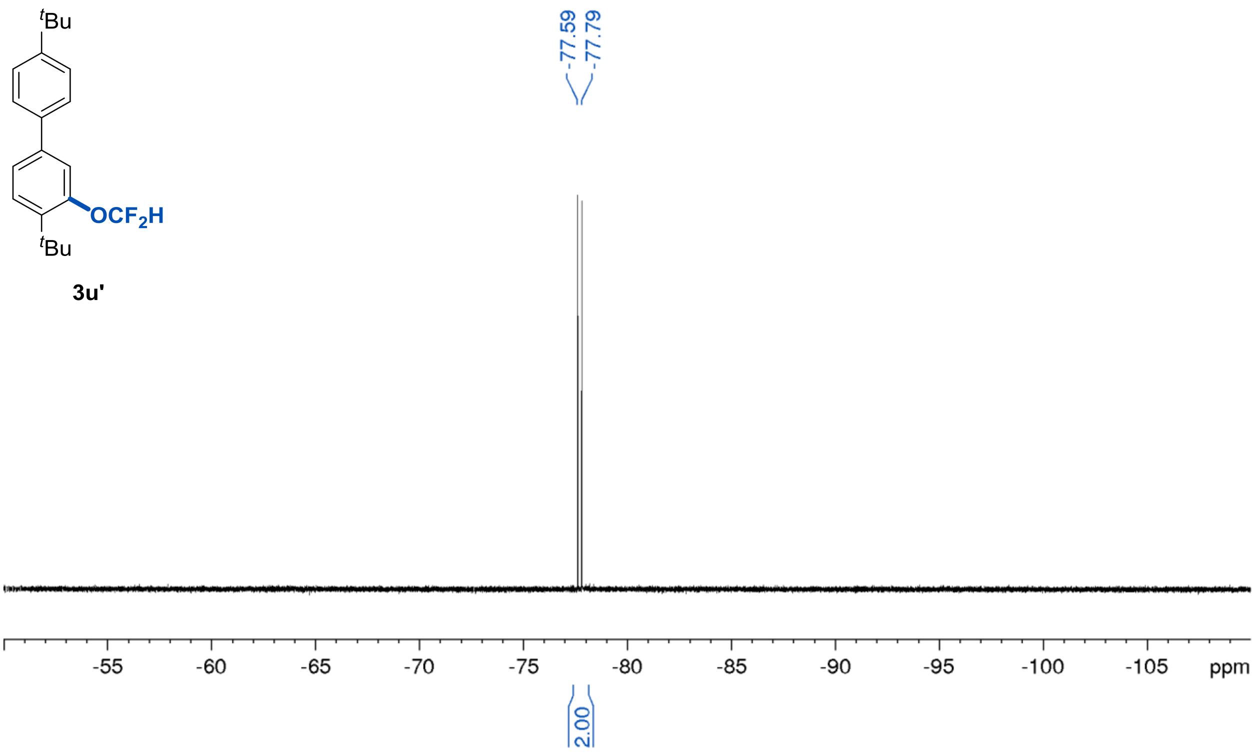

^{1}H NMR (700 MHz, $CDCl_3$, 25 °C) of 3v

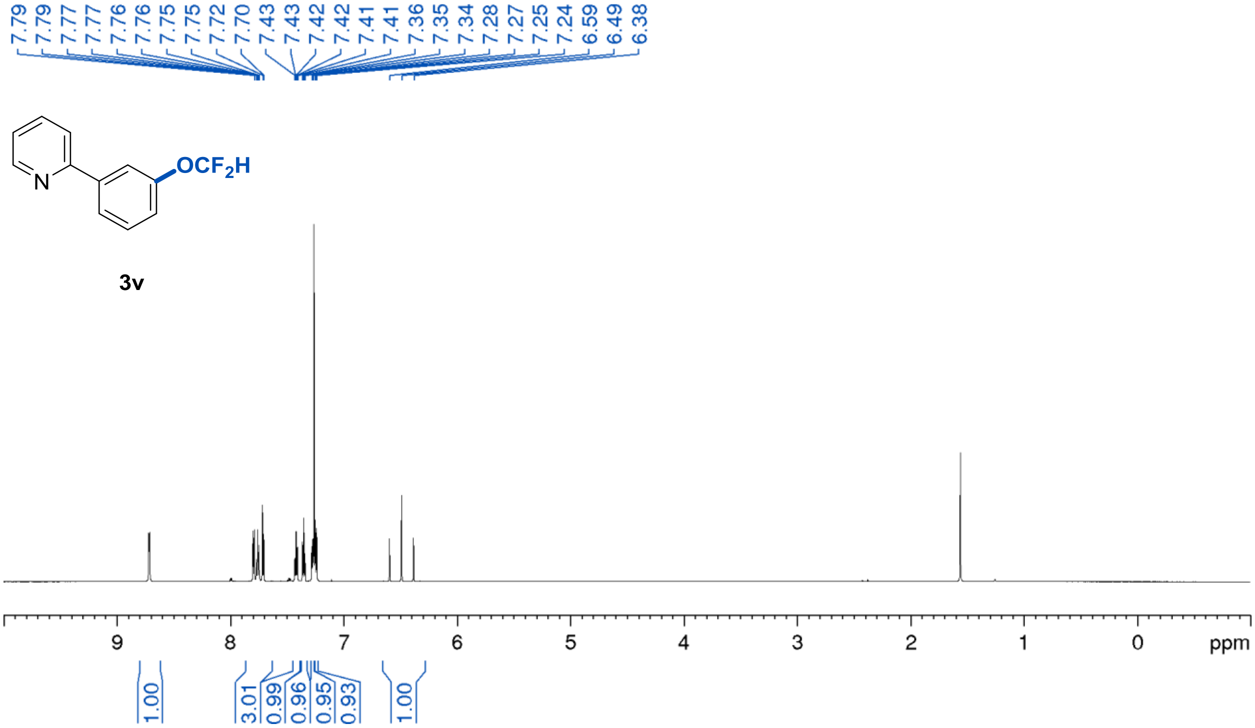

^{13}C NMR (175 MHz, $CDCl_3$, 25 °C) of 3v

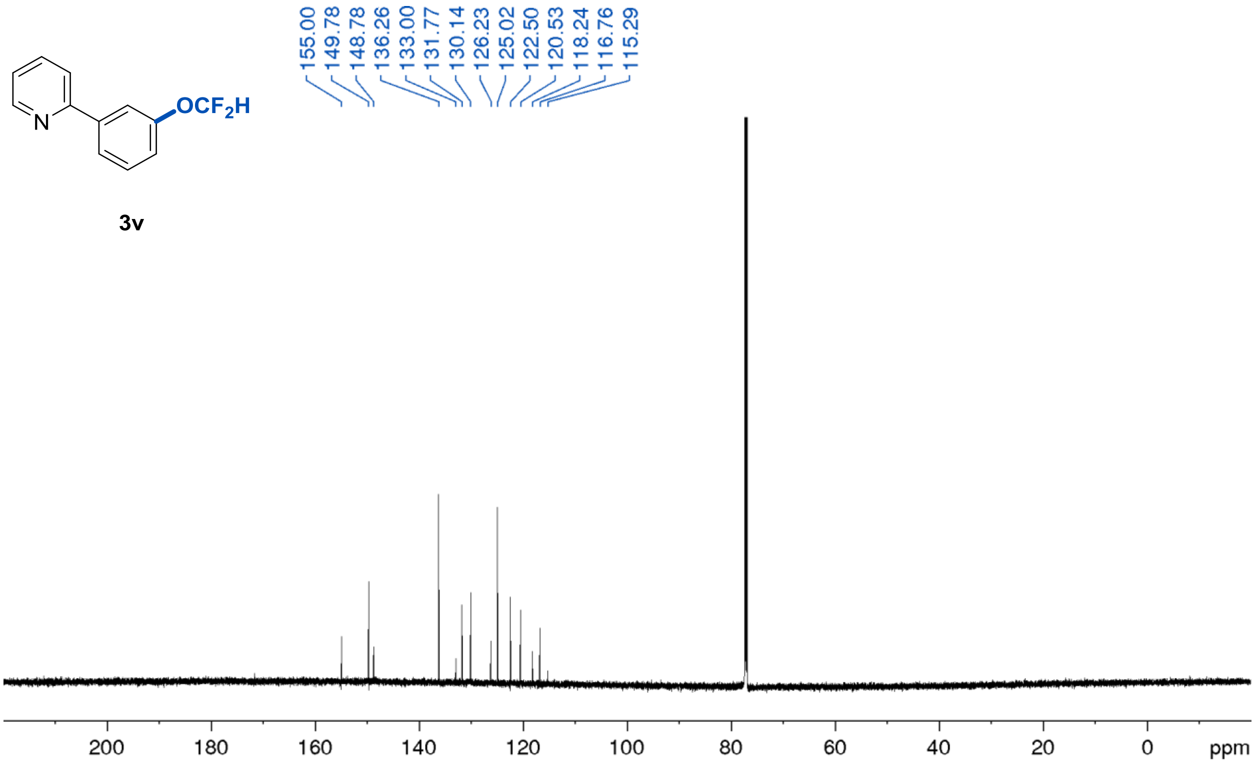

^{19}F NMR (376 MHz, CDCl$_3$, 25 ºC) of 3v

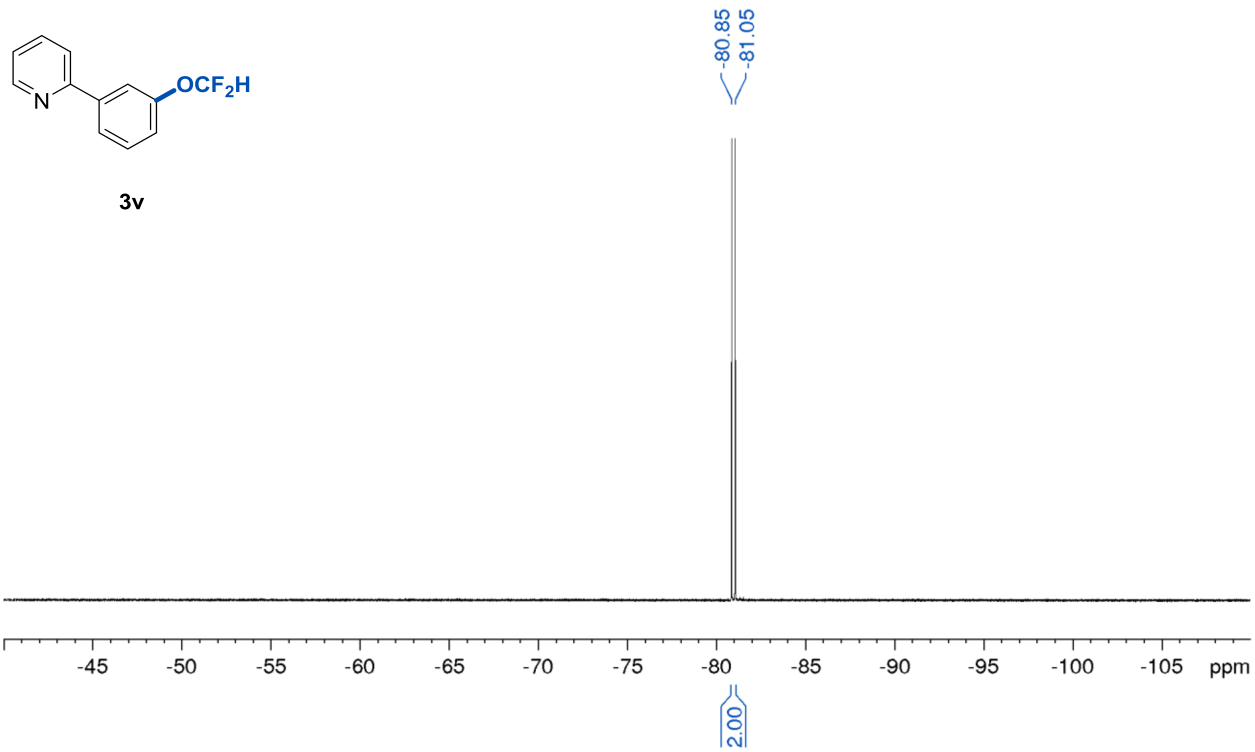

^{1}H NMR (700 MHz, CDCl$_3$, 25 ºC) of3 v' and 3v''

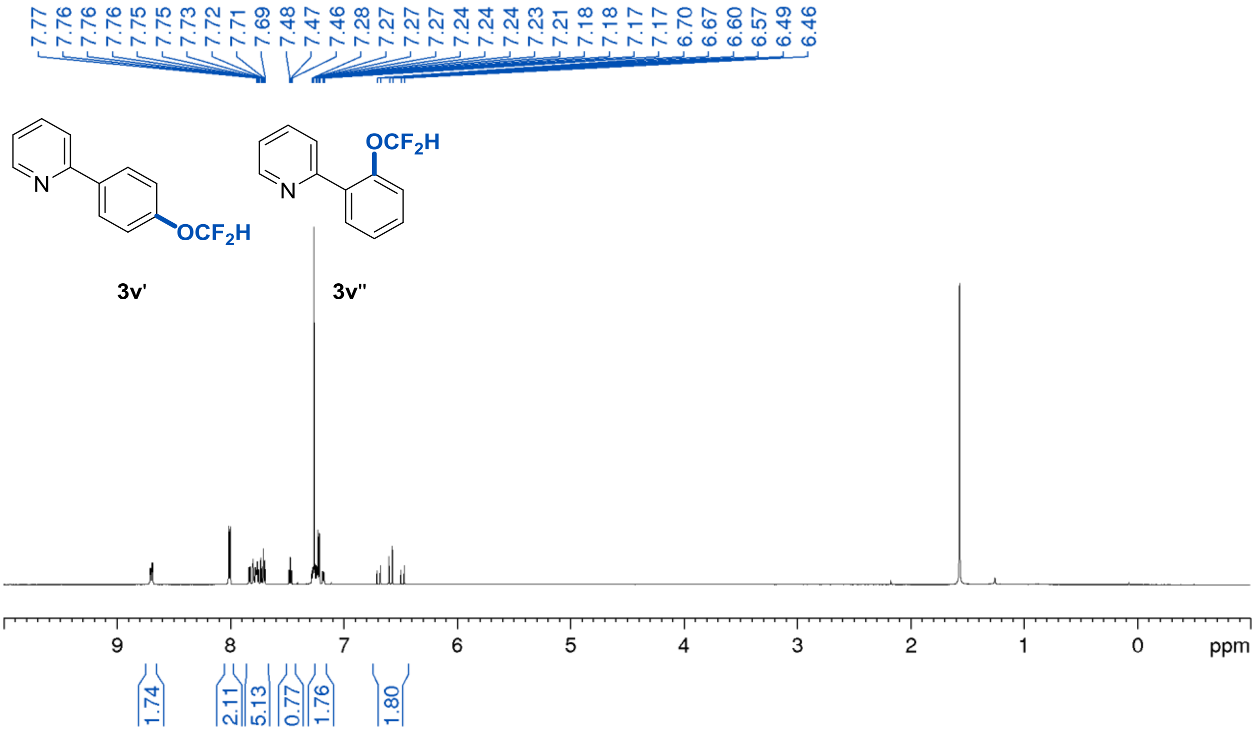

^{13}C NMR (175 MHz, $CDCl_3$, 25 ºC) of 3v' and 3v''

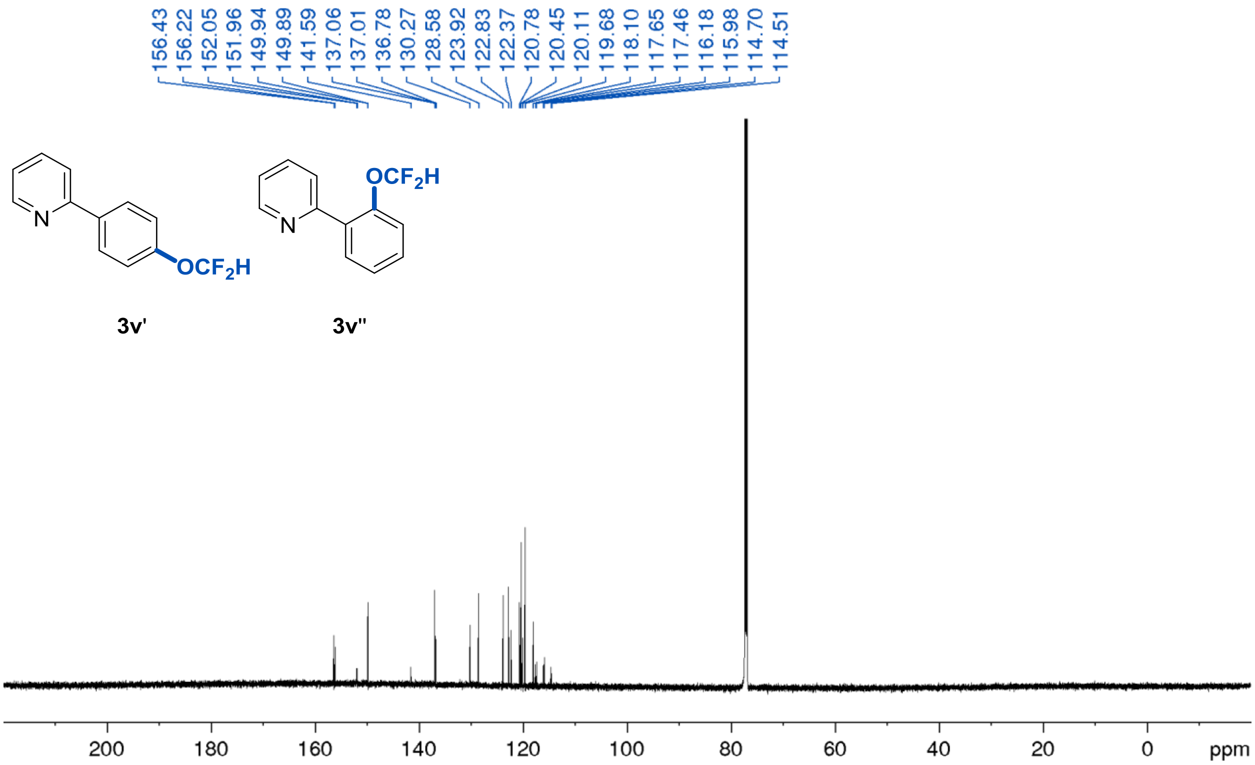

^{19}F NMR (376 MHz, $CDCl_3$, 25 ºC) of 3v' and 3v''

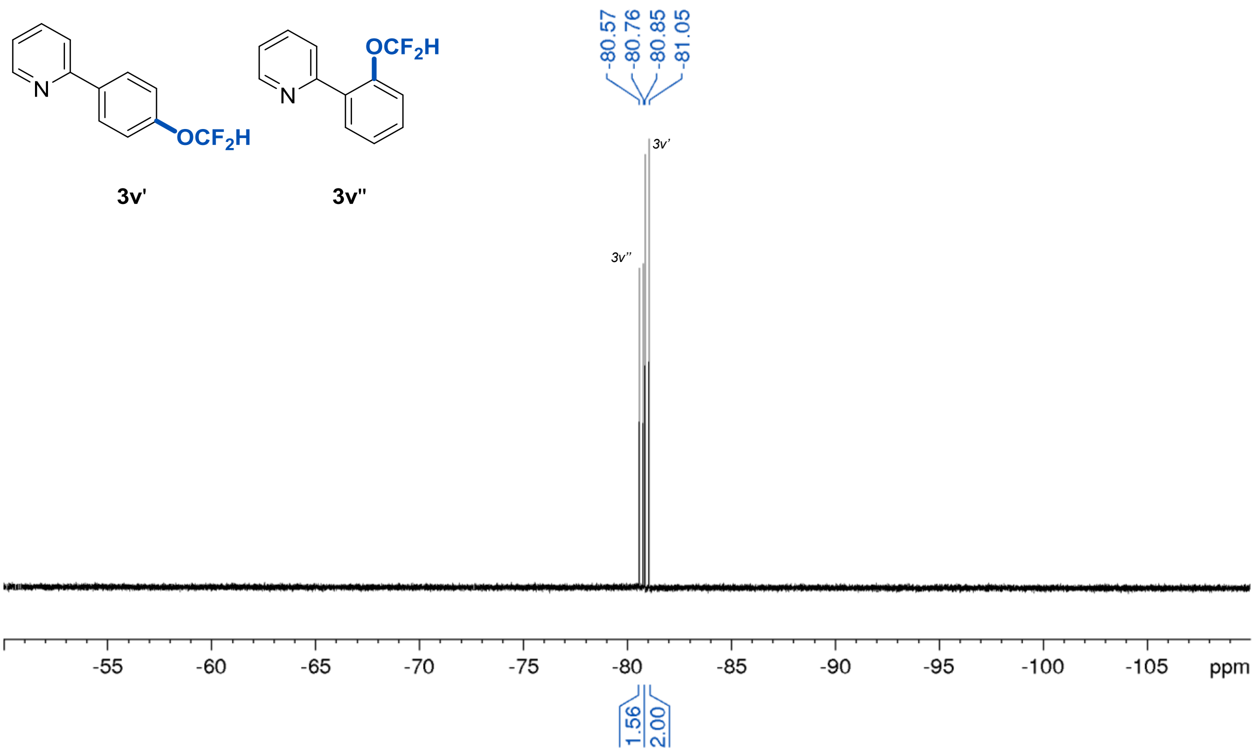

^{1}H NMR (700 MHz, $CDCl_3$, 25 ºC) of 3w and 3w'

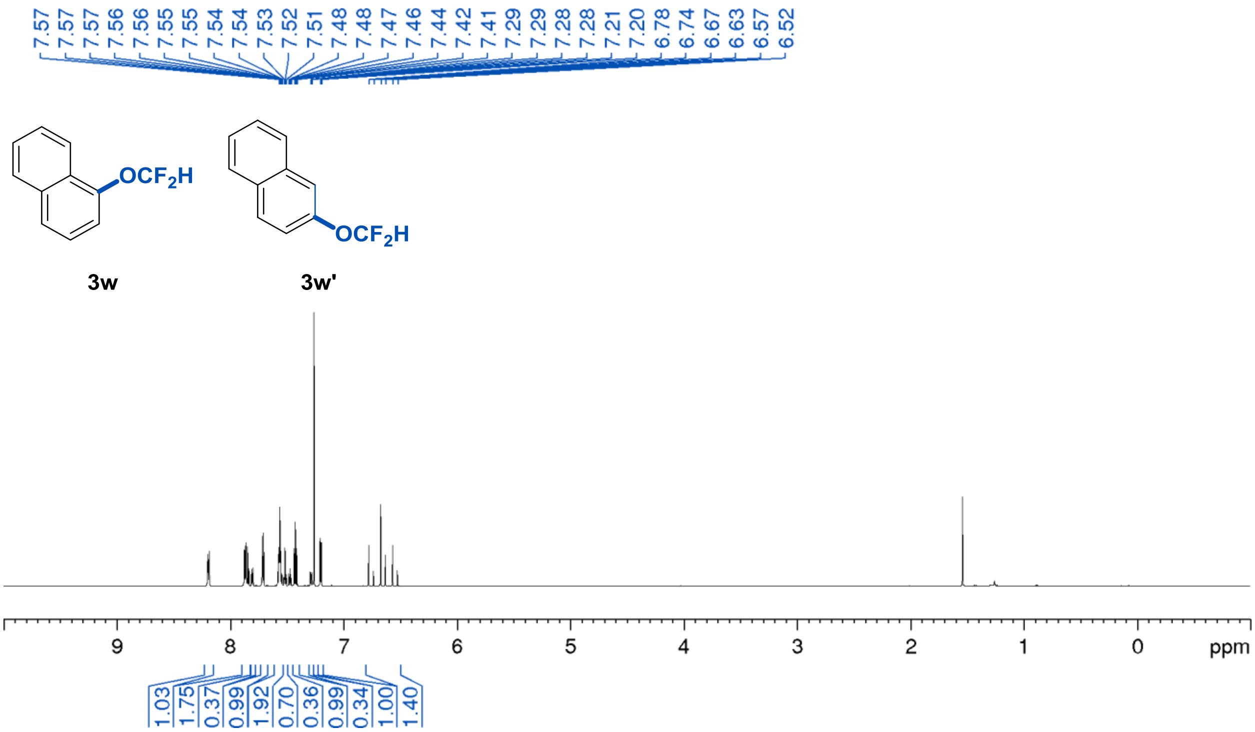

^{13}C NMR (175 MHz, $CDCl_3$, 25 ºC) of 3w and 3w'

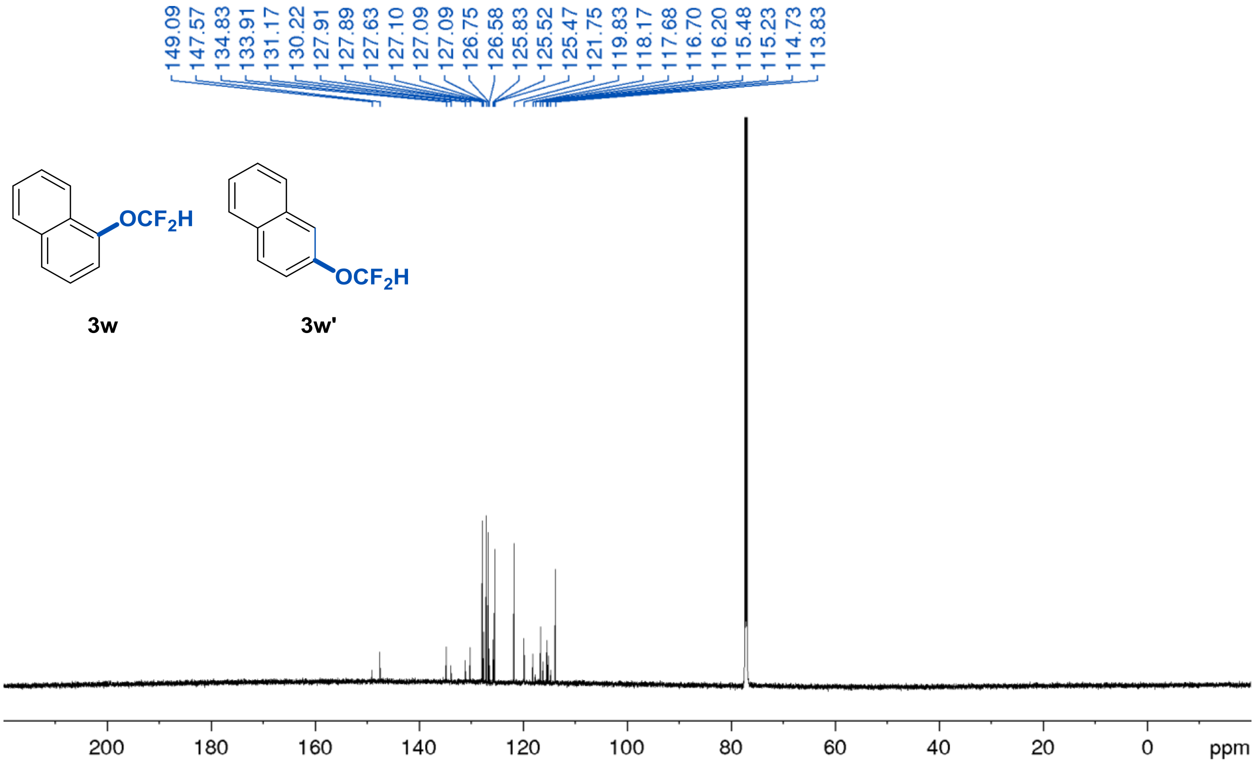

^{19}F NMR (376 MHz, $CDCl_3$, 25 ºC) of 3w and 3w'

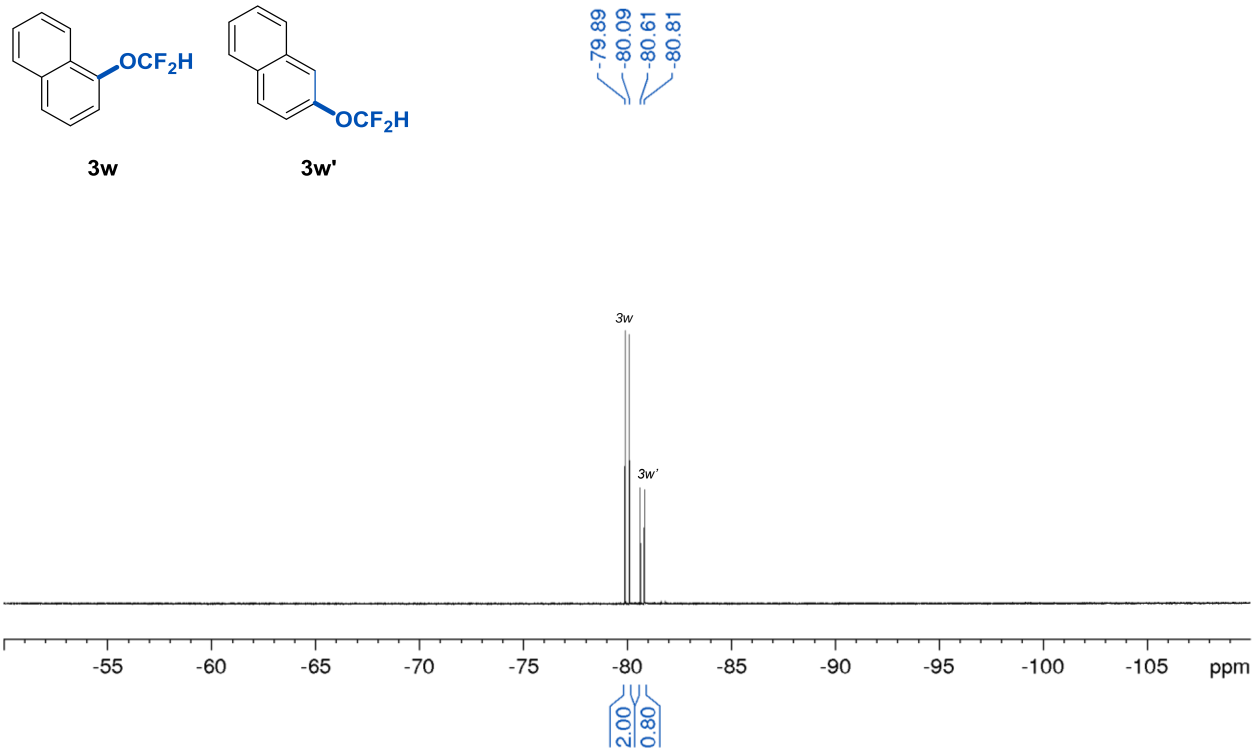

^1H NMR (700 MHz, $CDCl_3$, 25 ºC) of 3x

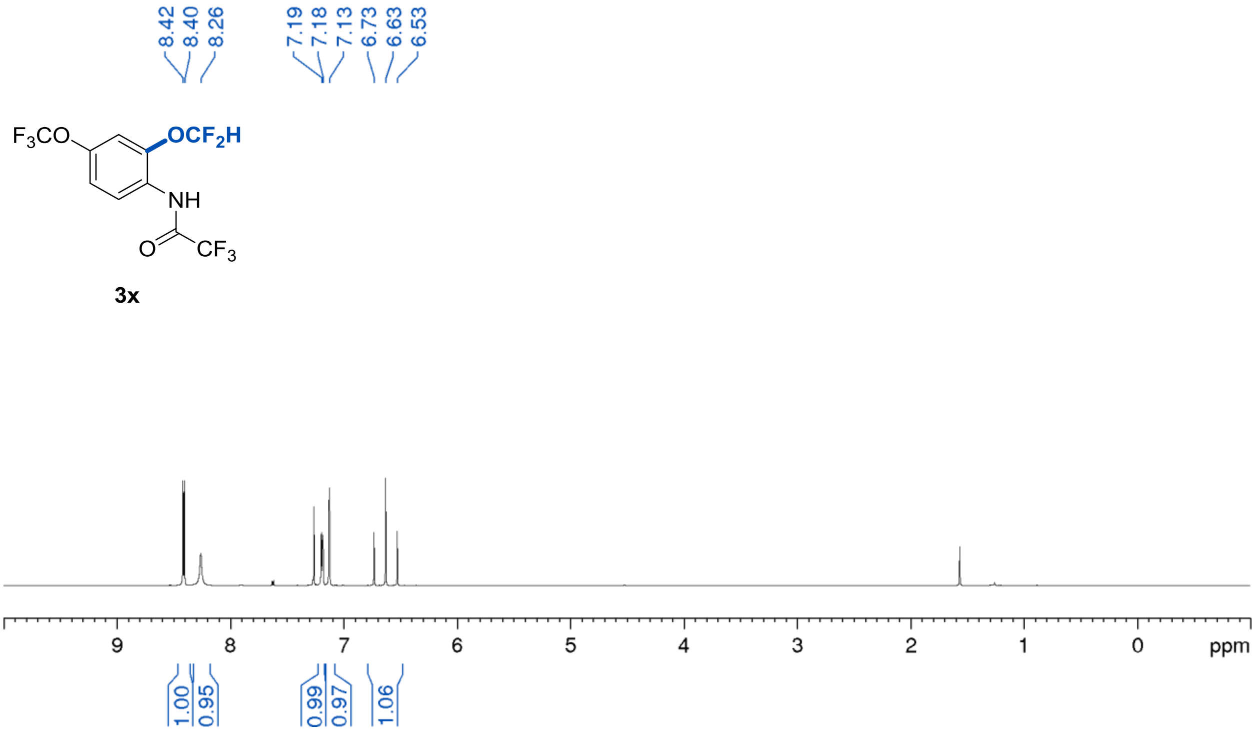

^{13}C NMR (175 MHz, $CDCl_3$, 25 ºC) of 3x

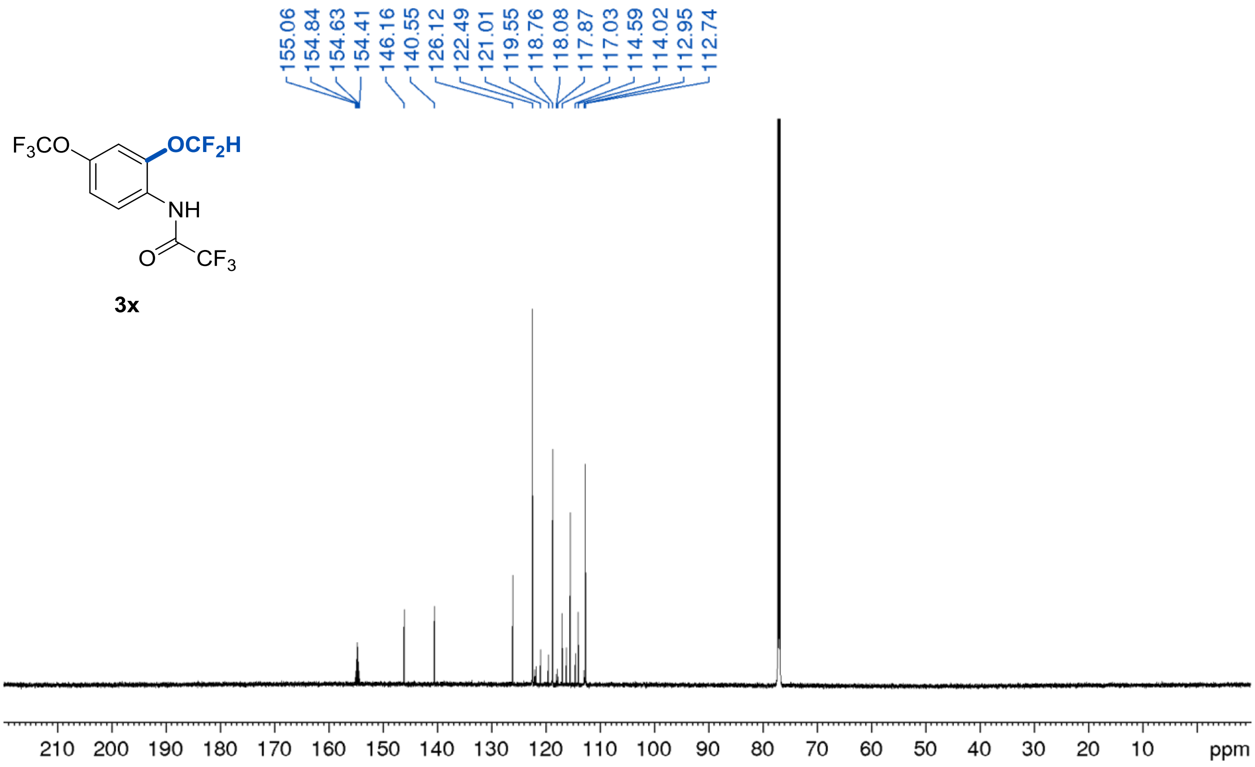

^{19}F NMR (376 MHz, $CDCl_3$, 25 ºC) of 3x

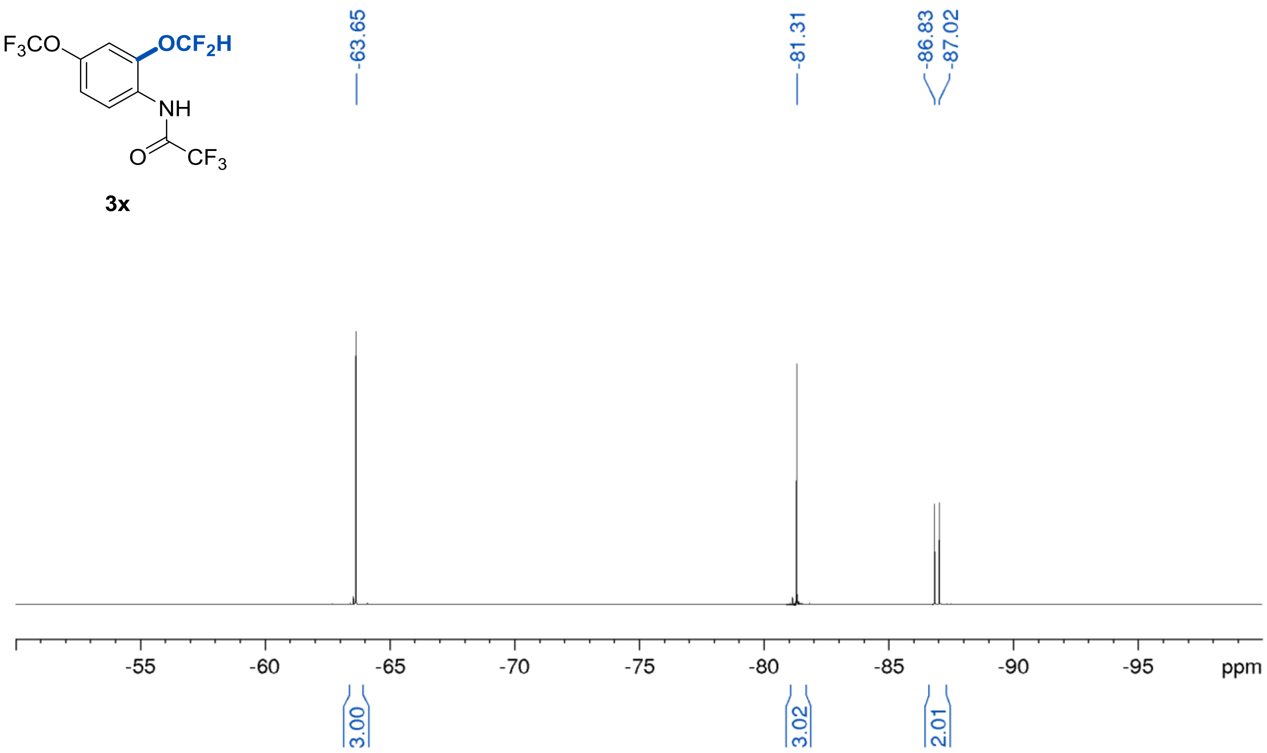

^{1}H NMR (700 MHz, CDCl₃, 25 ºC) of 3x'

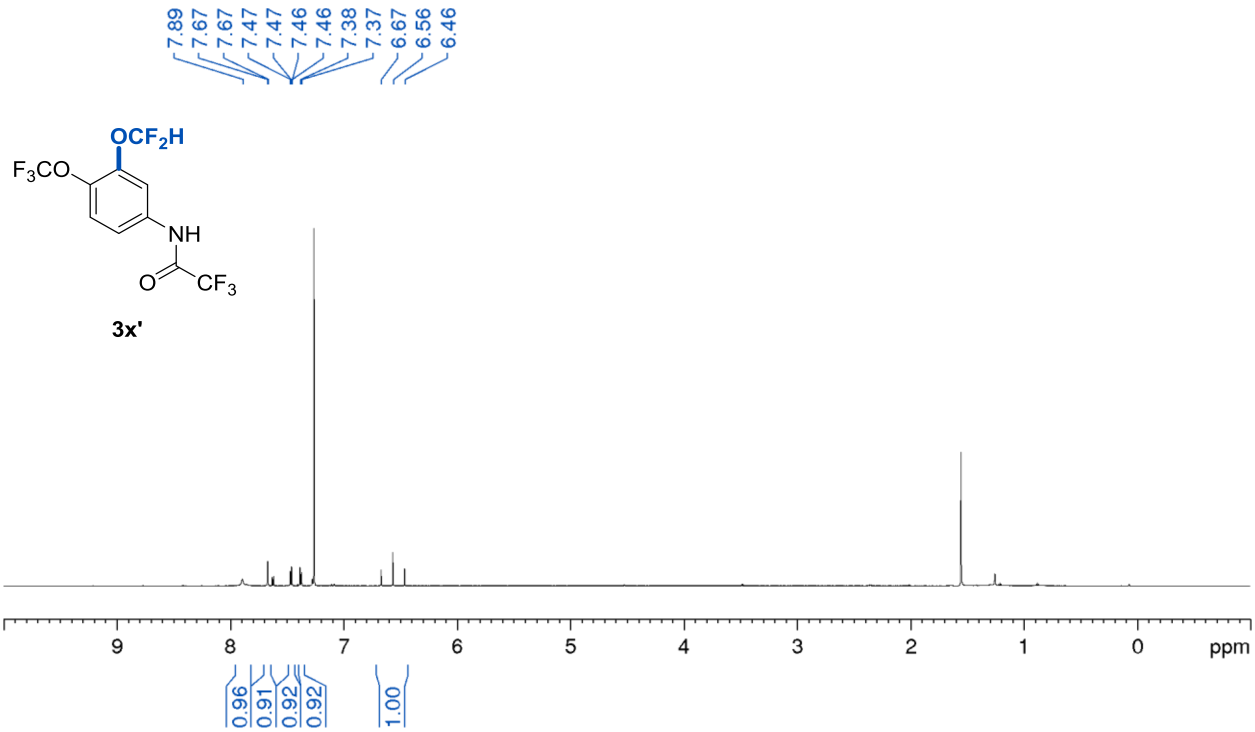

^{13}C NMR (175 MHz, CDCl₃, 25 ºC) of 3x'

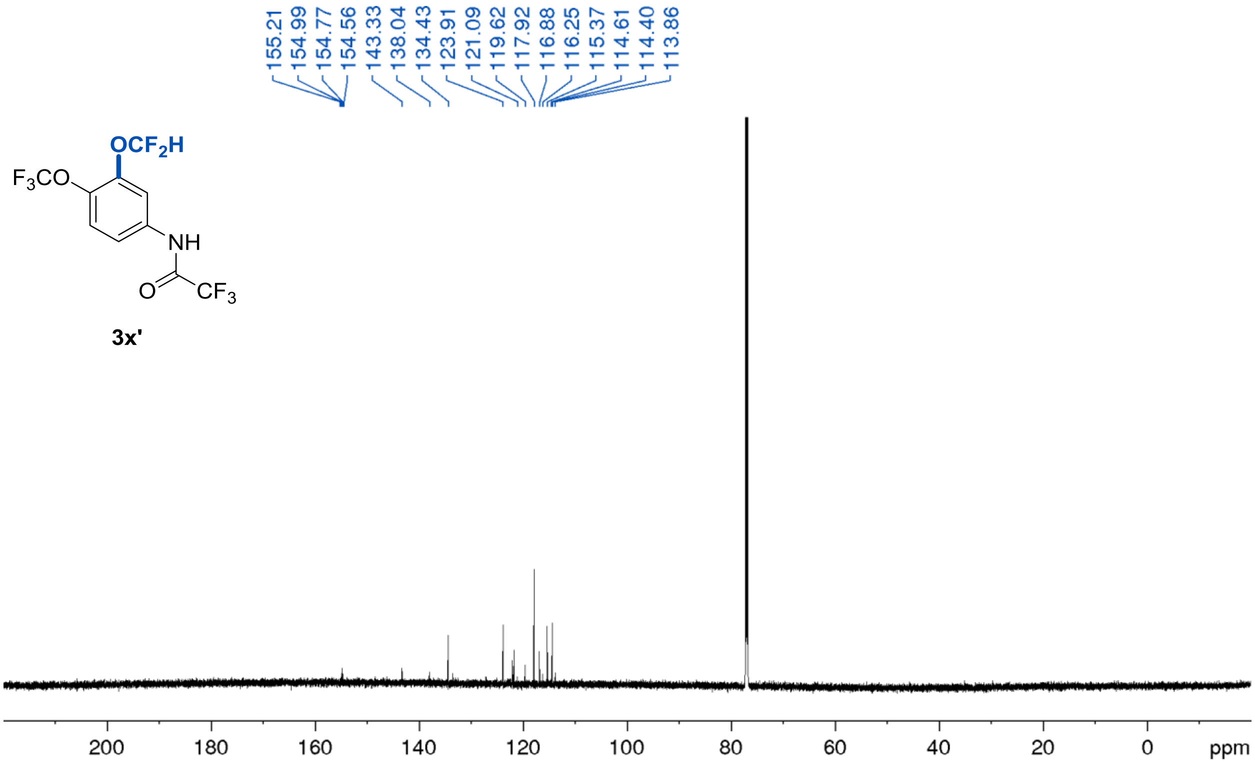

^{19}F NMR (376 MHz, CDCl$_3$, 25 ºC) of 3x'

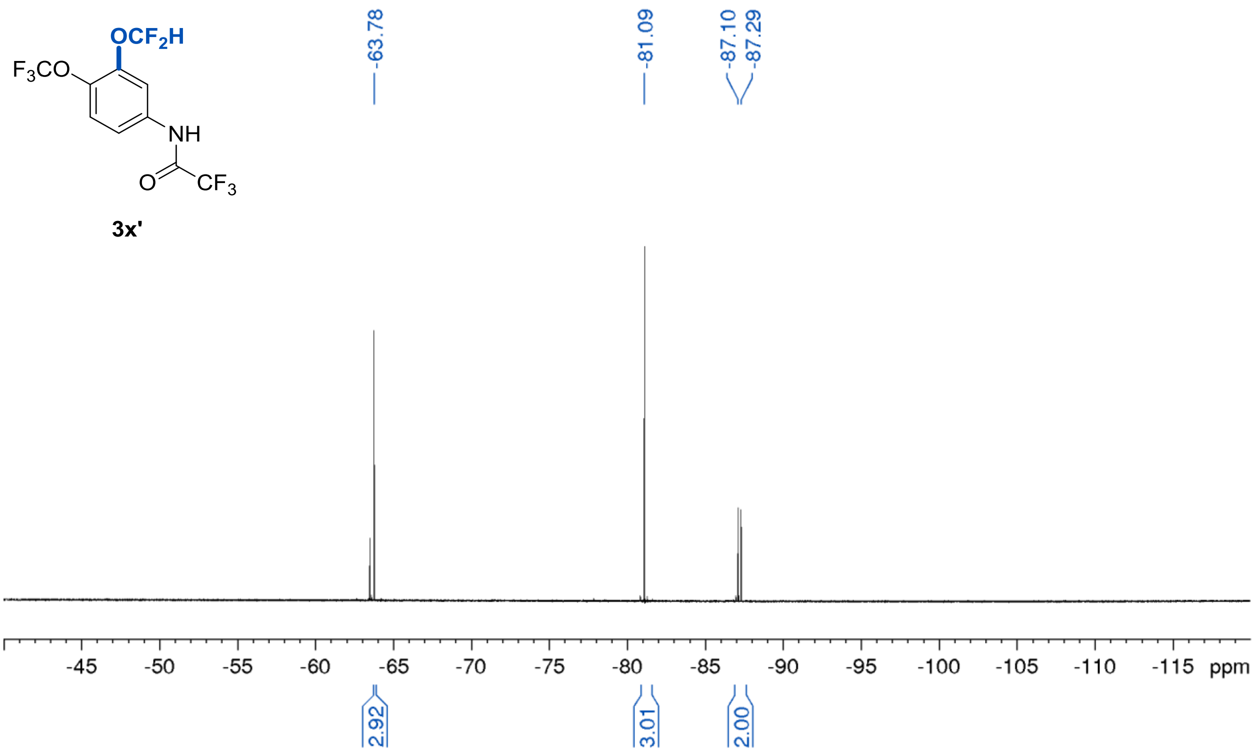

^{1}H NMR (700 MHz, CDCl$_3$, 25 ºC) of 3y-*ortho*

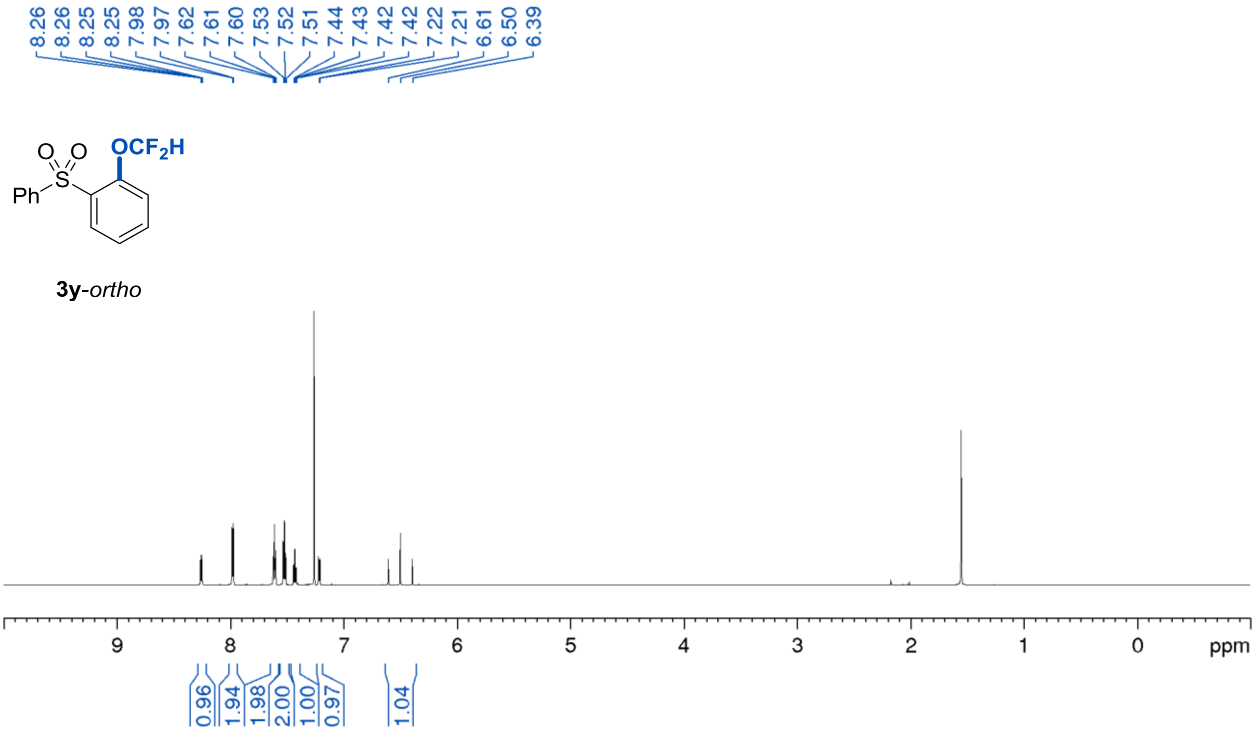

^{13}C NMR (175 MHz, $CDCl_3$, 25 ºC) of 3y-*ortho*

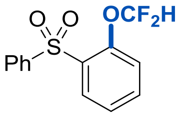

3y-*ortho*

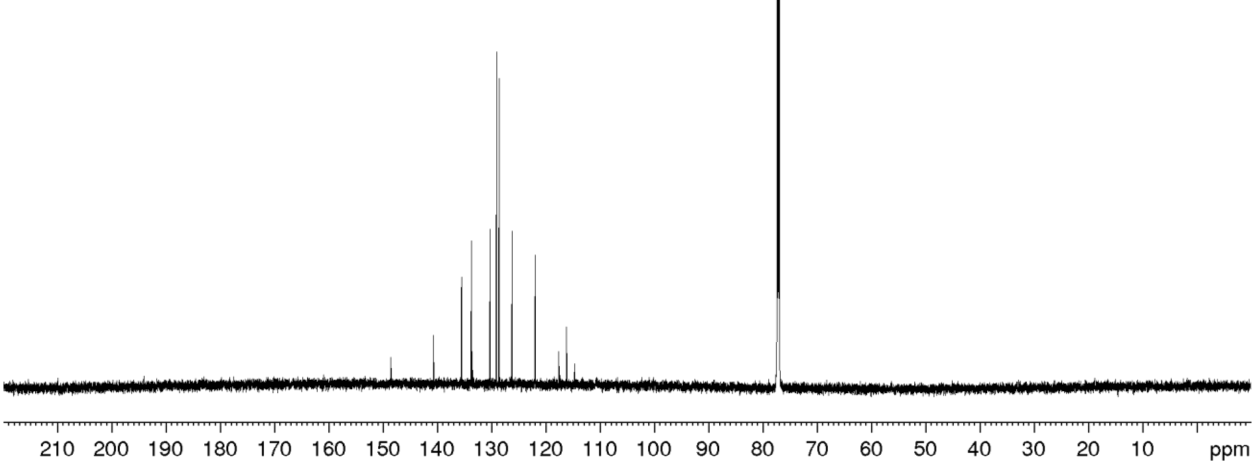

^{19}F NMR (376 MHz, $CDCl_3$, 25 ºC) of 3y-*ortho*

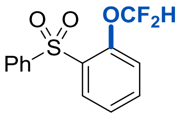

3y-*ortho*

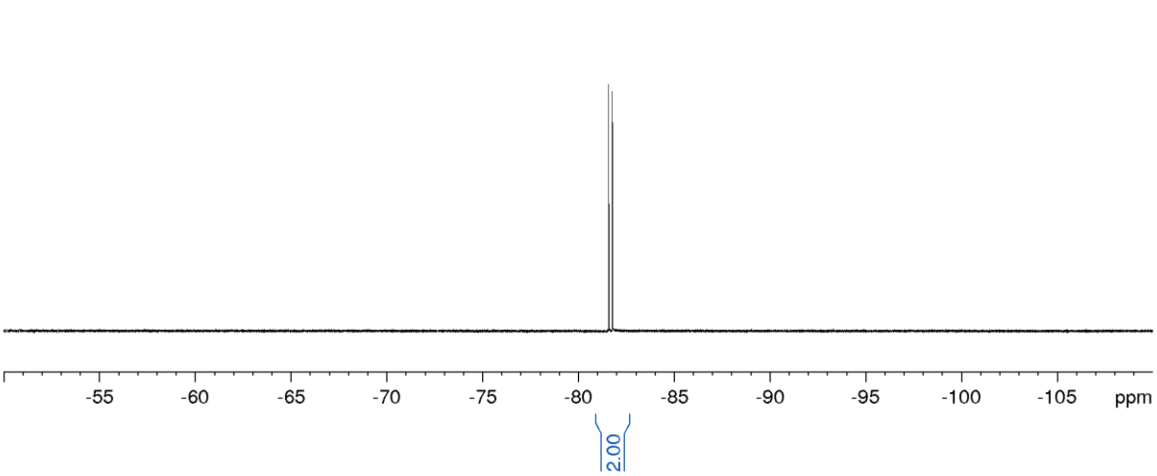

^{1}H NMR (700 MHz, $CDCl_3$, 25 °C) of 3y-*meta* and -*para*

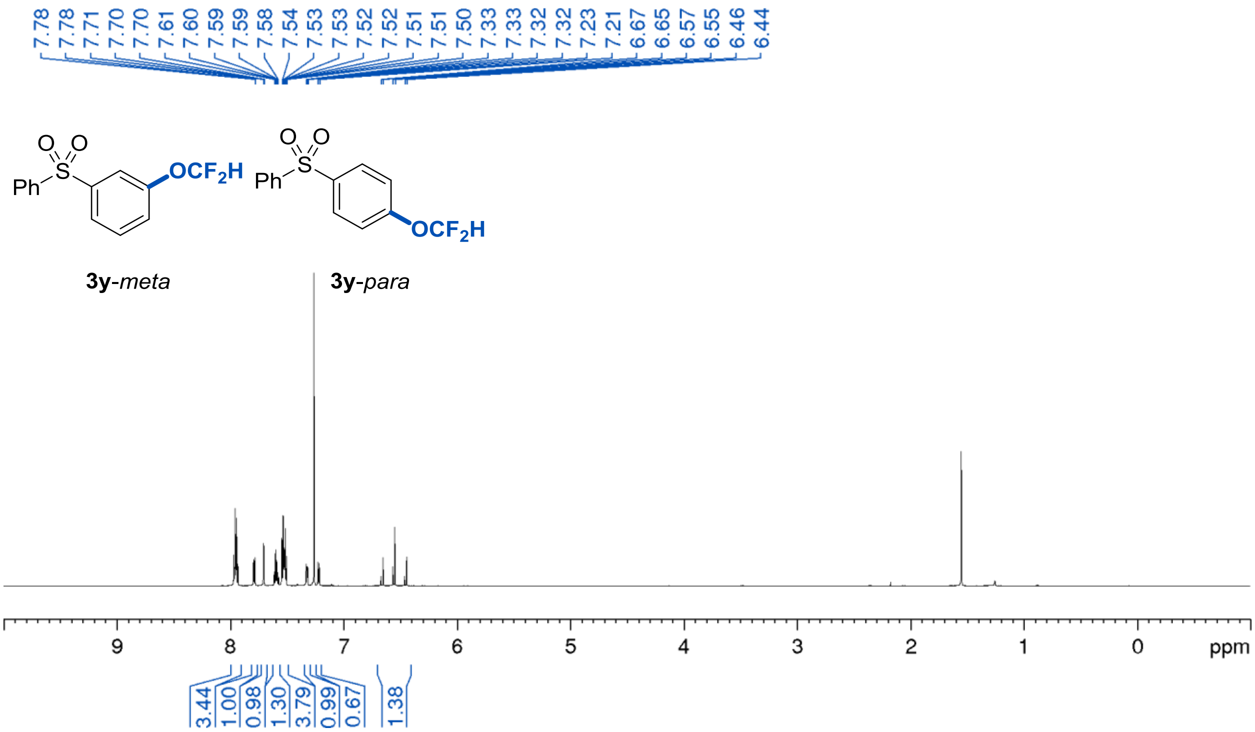

^{13}C NMR (175 MHz, $CDCl_3$, 25 °C) of 3y-*meta* and -*para*

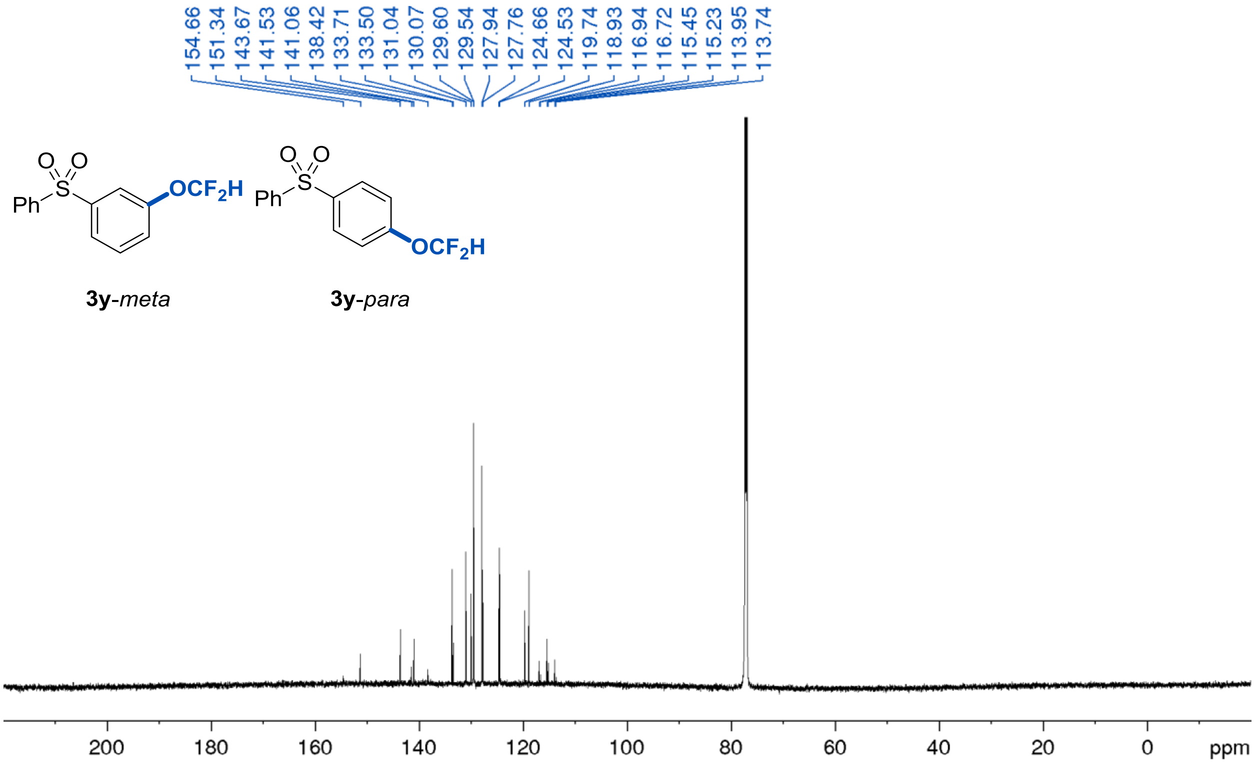

^{19}F NMR (376 MHz, CDCl$_3$, 25 ºC) of 3y-*meta* and -*para*

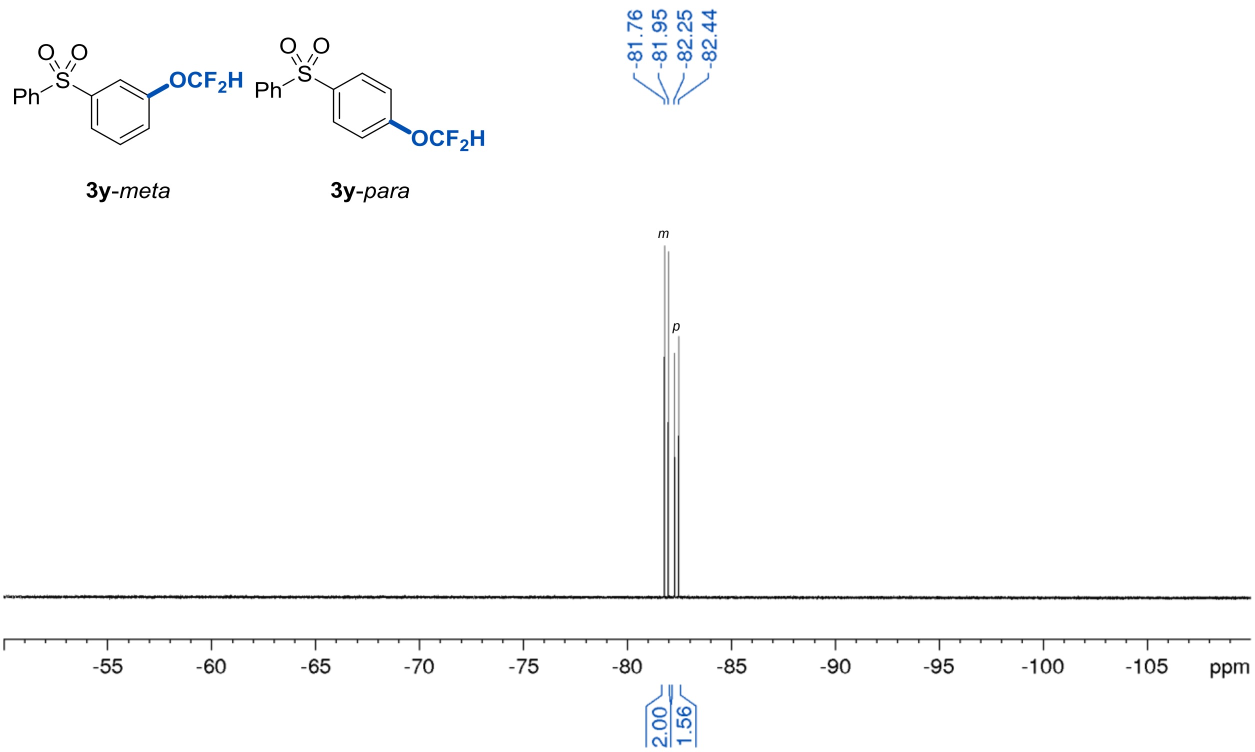

^{1}H NMR (700 MHz, CDCl$_3$, 25 ºC) of 3z-*ortho*

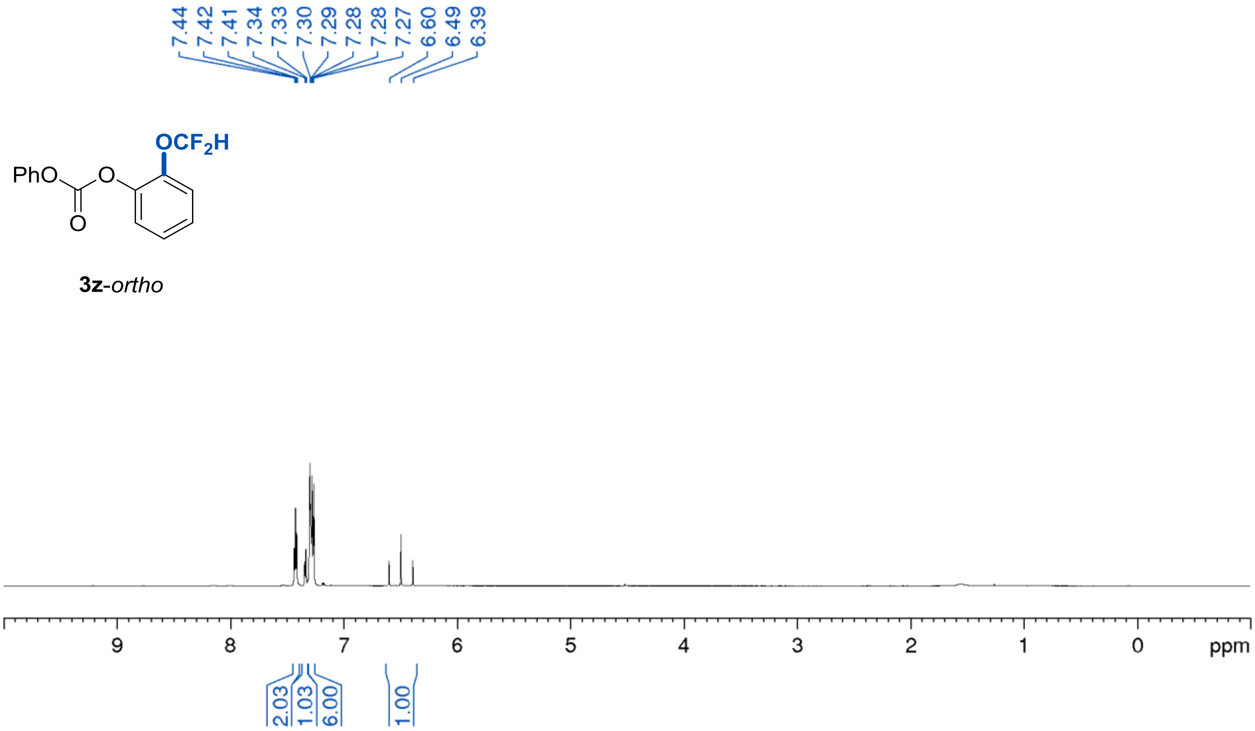

^{13}C NMR (175 MHz, $CDCl_3$, 25 ºC) of 3z-*ortho*

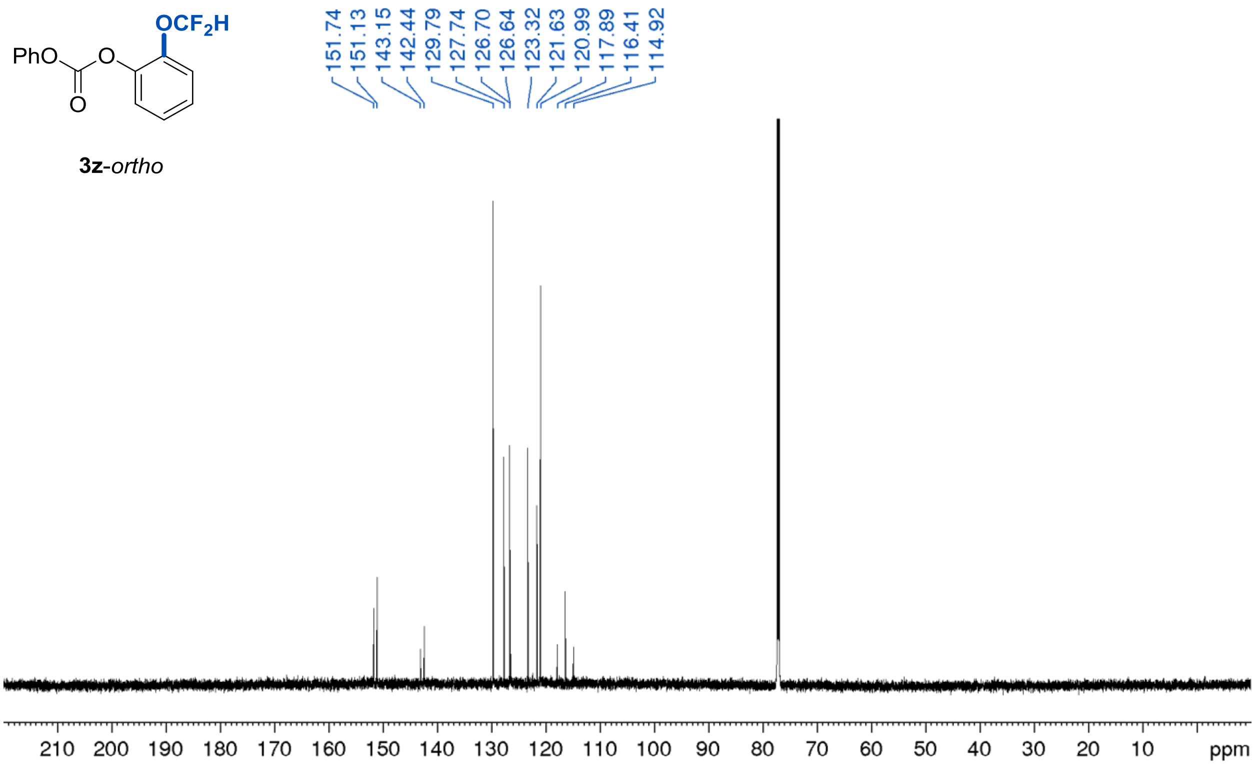

^{19}F NMR (376 MHz, $CDCl_3$, 25 ºC) of 3z-*ortho*

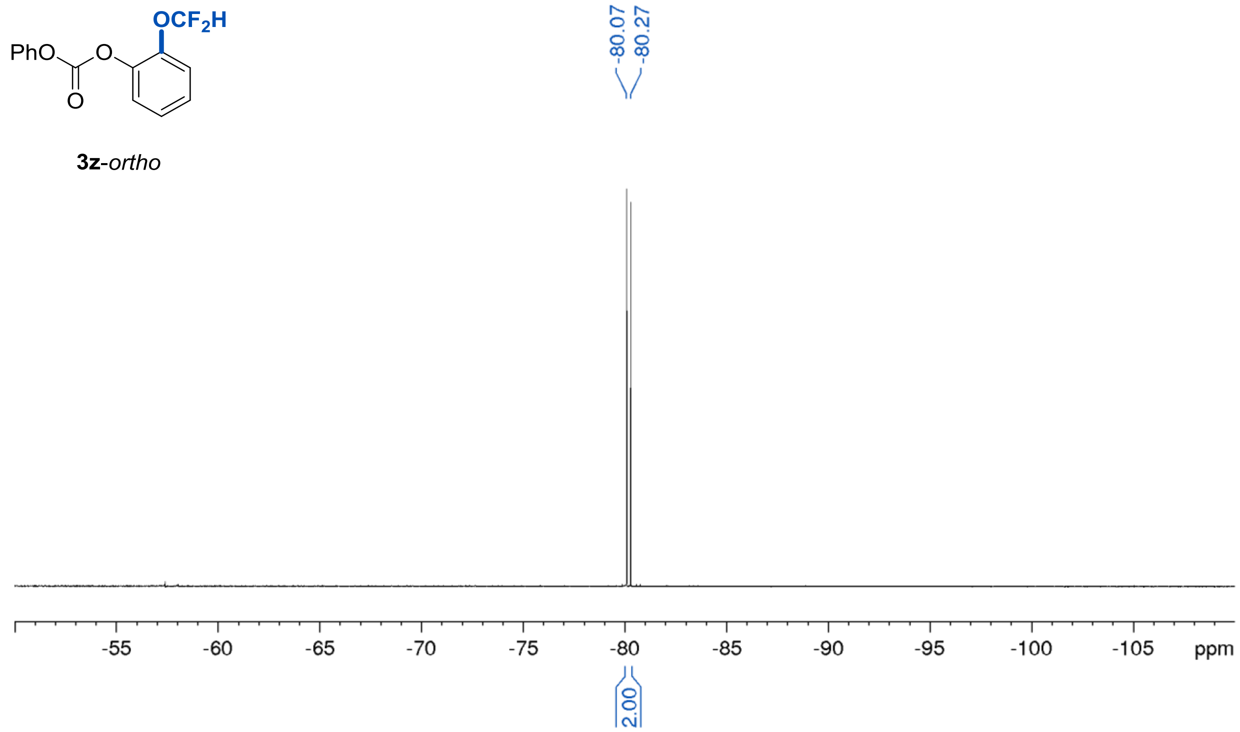

^{1}H NMR (700 MHz, $CDCl_3$, 25 °C) of 3z-*meta* and -*para*

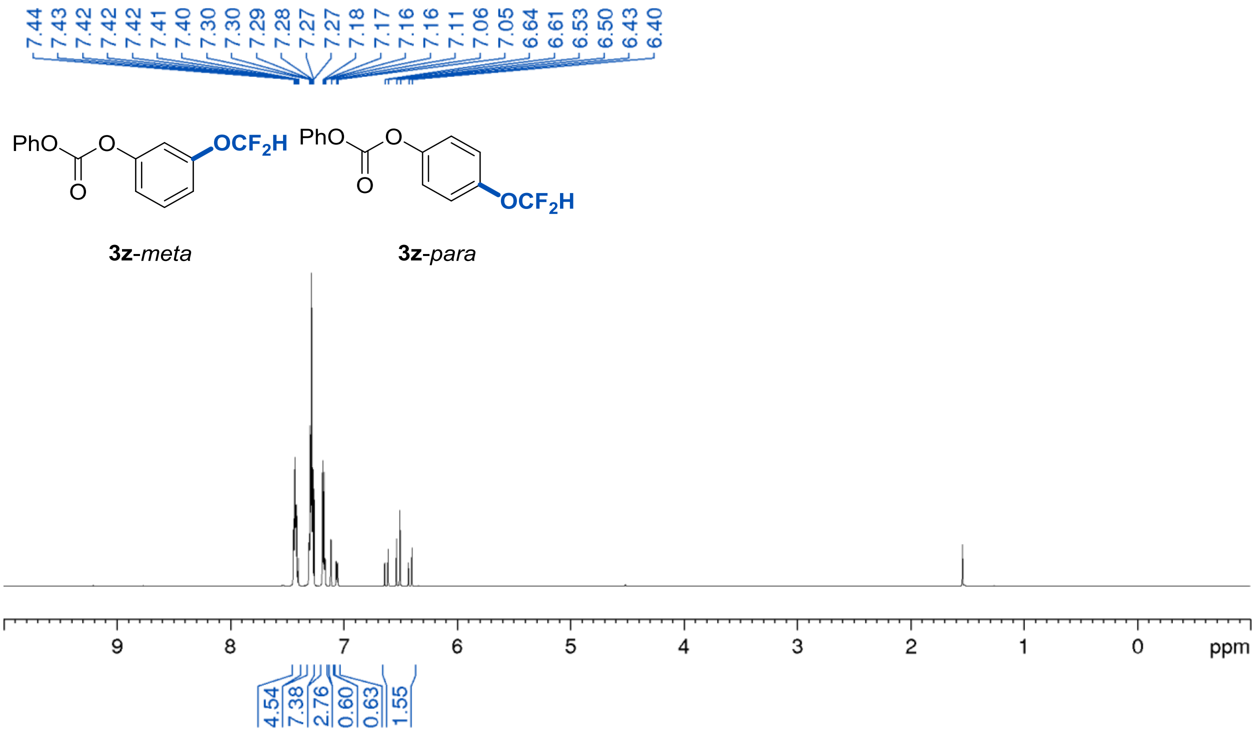

^{13}C NMR (175 MHz, $CDCl_3$, 25 °C) of 3z-*meta* and -*para*

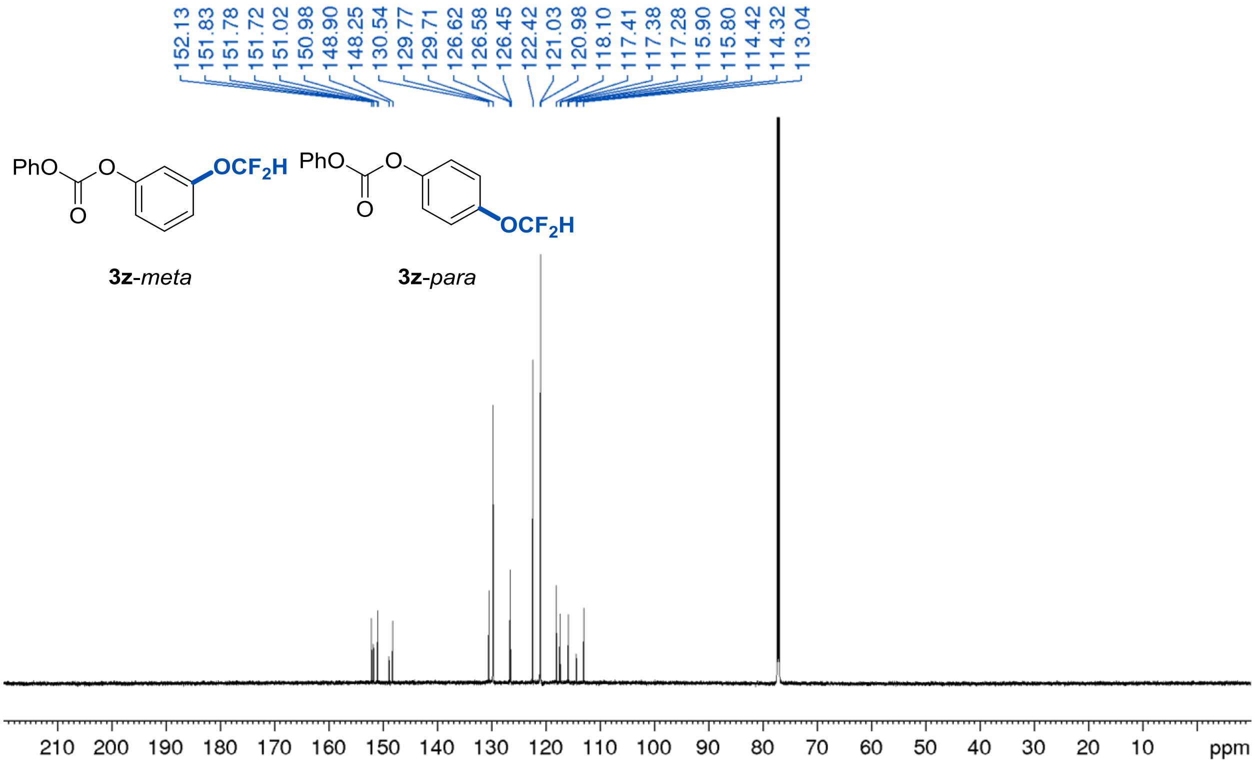

^{19}F NMR (376 MHz, $CDCl_3$, 25 ºC) of 3z-*meta* and -*para*

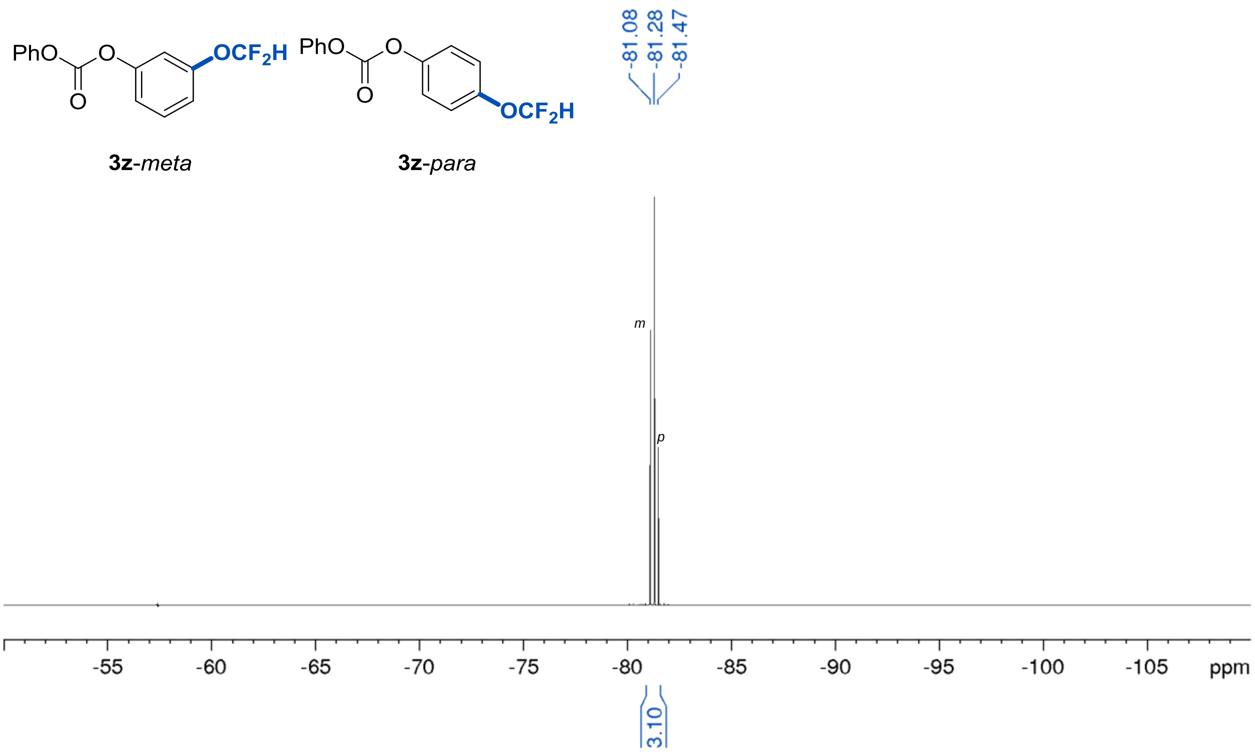

^{1}H NMR (700 MHz, $CDCl_3$, 25 ºC) of 3aa

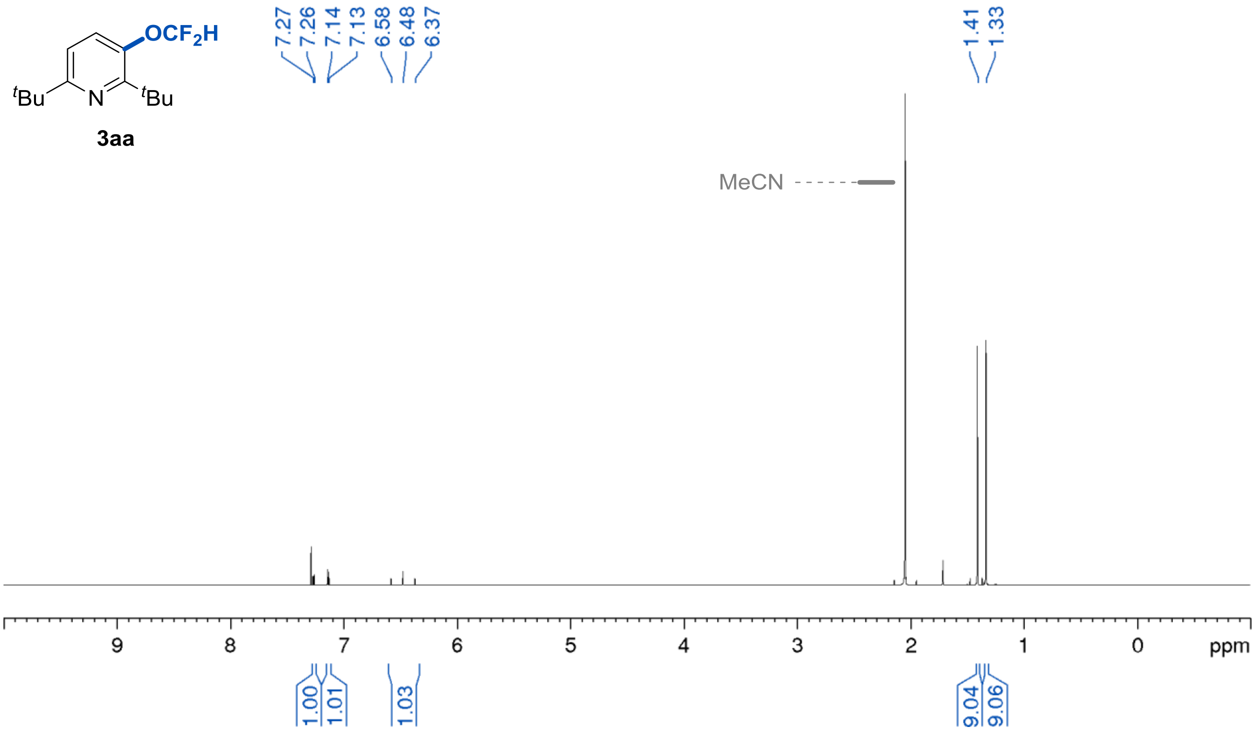

^{13}C NMR (175 MHz, $CDCl_3$, 25 ºC) of 3aa

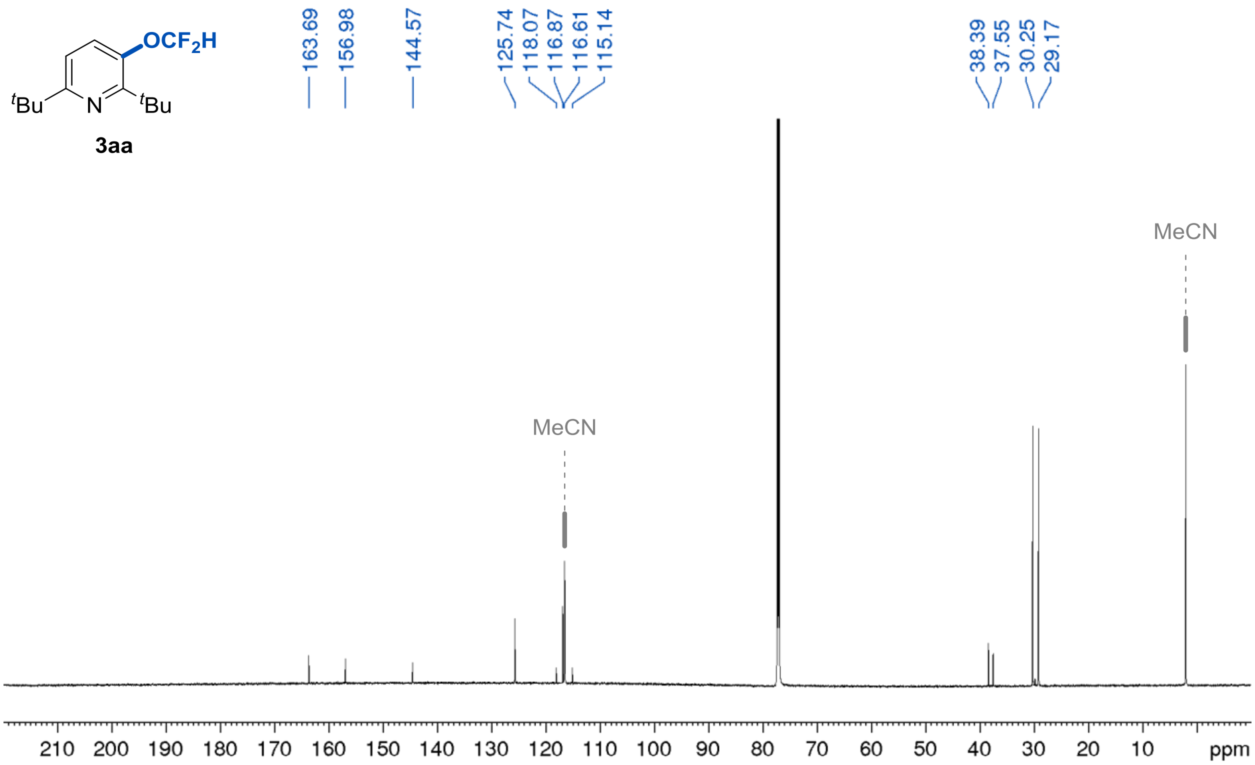

^{19}F NMR (376 MHz, $CDCl_3$, 25 ºC) of 3aa

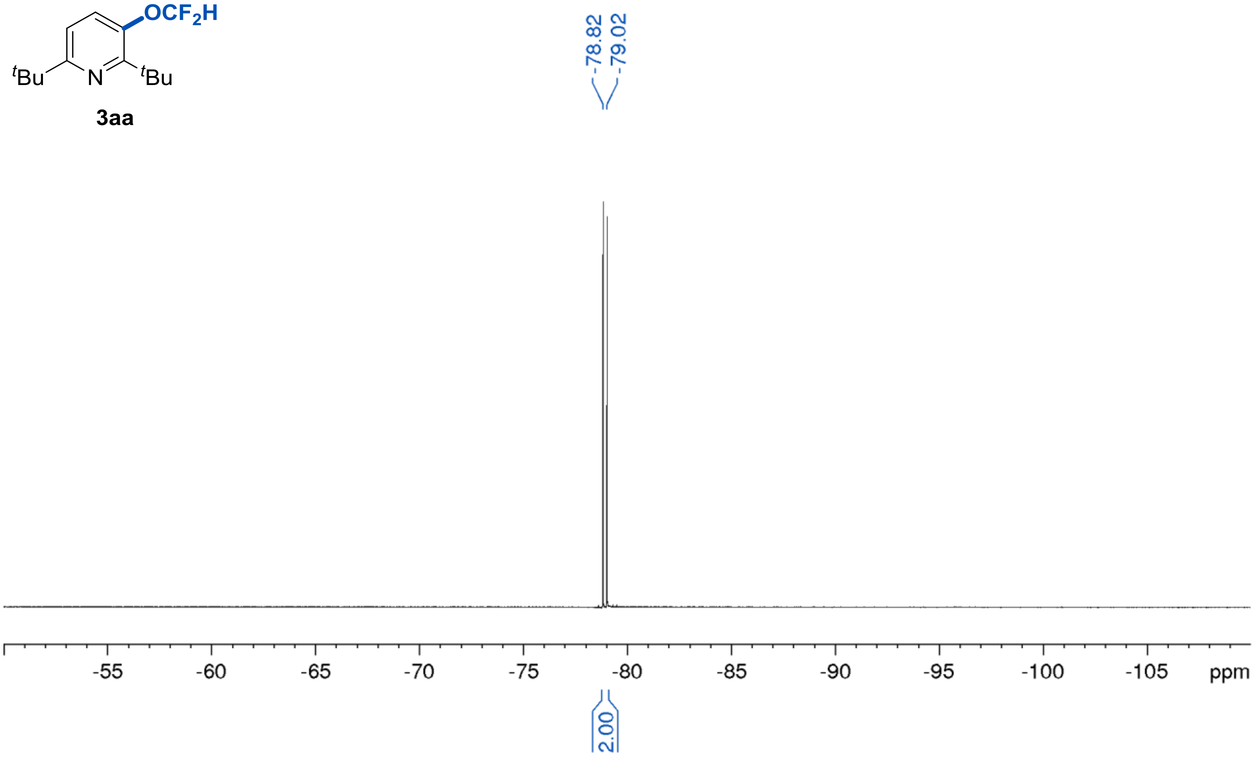

^{1}H NMR (700 MHz, $CDCl_3$, 25 °C) of 3ab

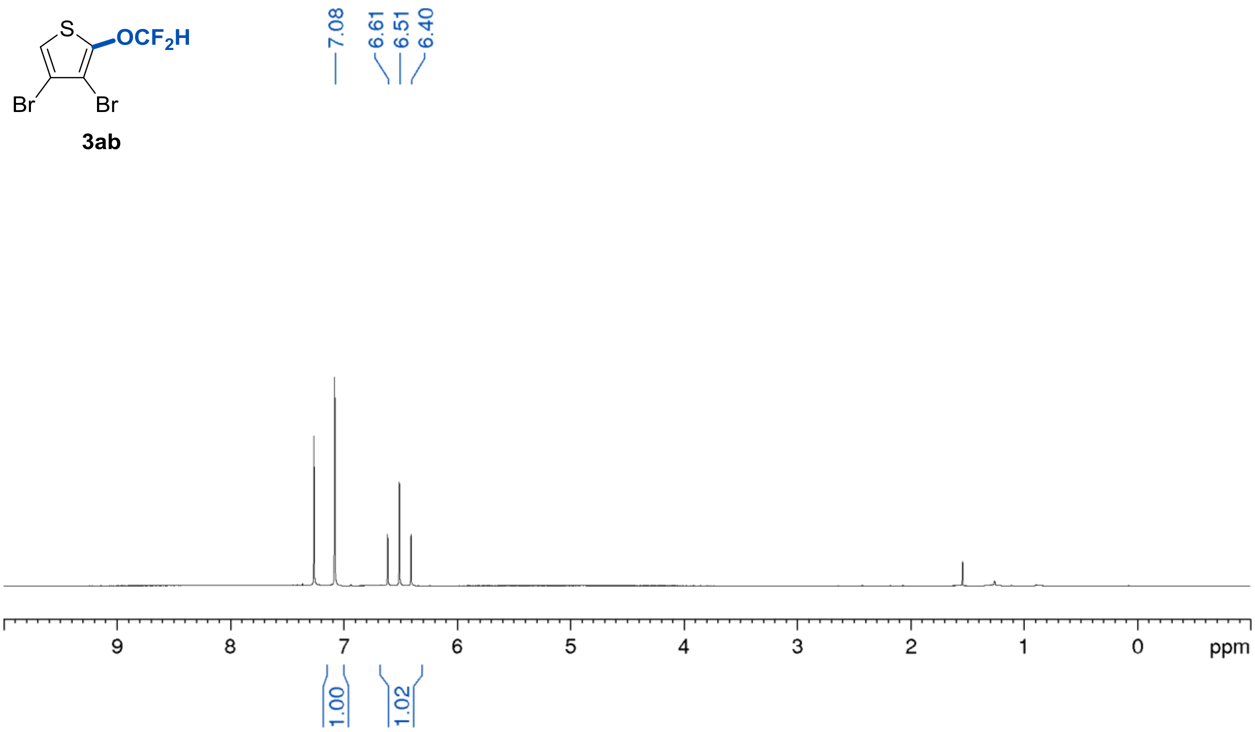

^{13}C NMR (175 MHz, $CDCl_3$, 25 °C) of 3ab

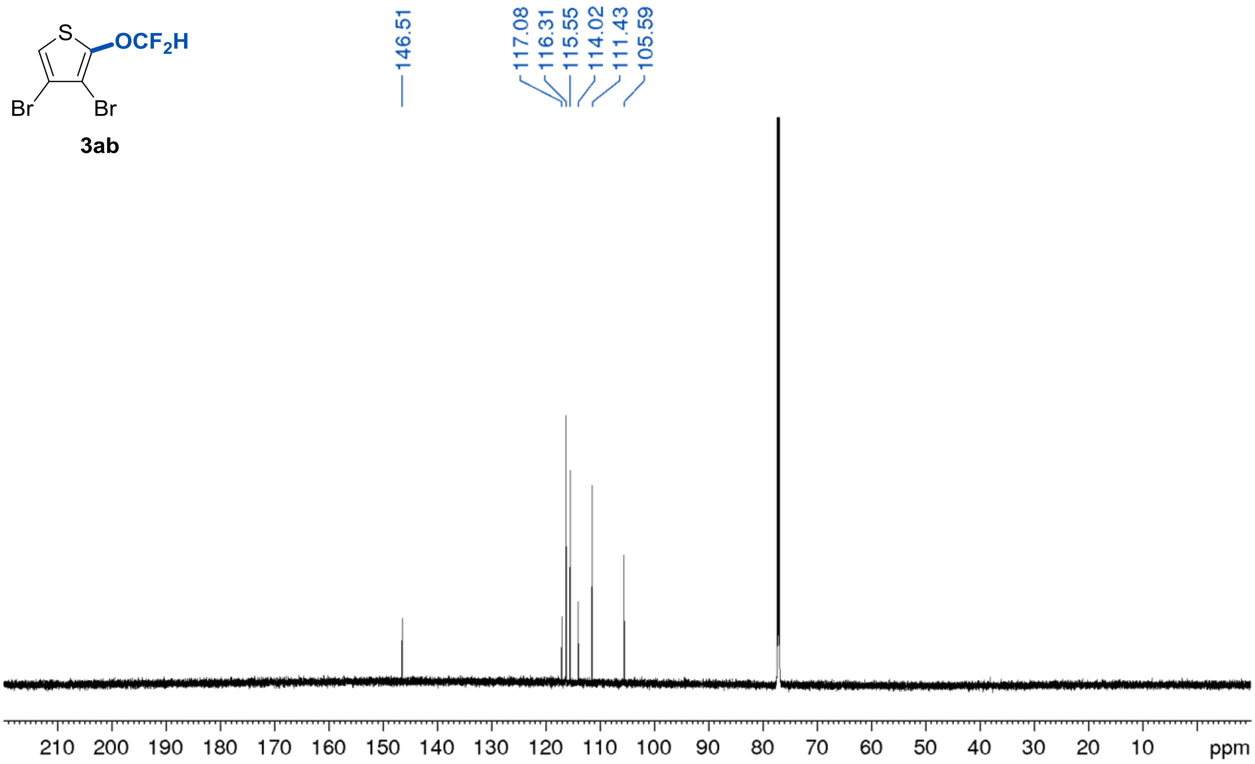

^{19}F NMR (376 MHz, CDCl$_3$, 25 °C) of 3ab

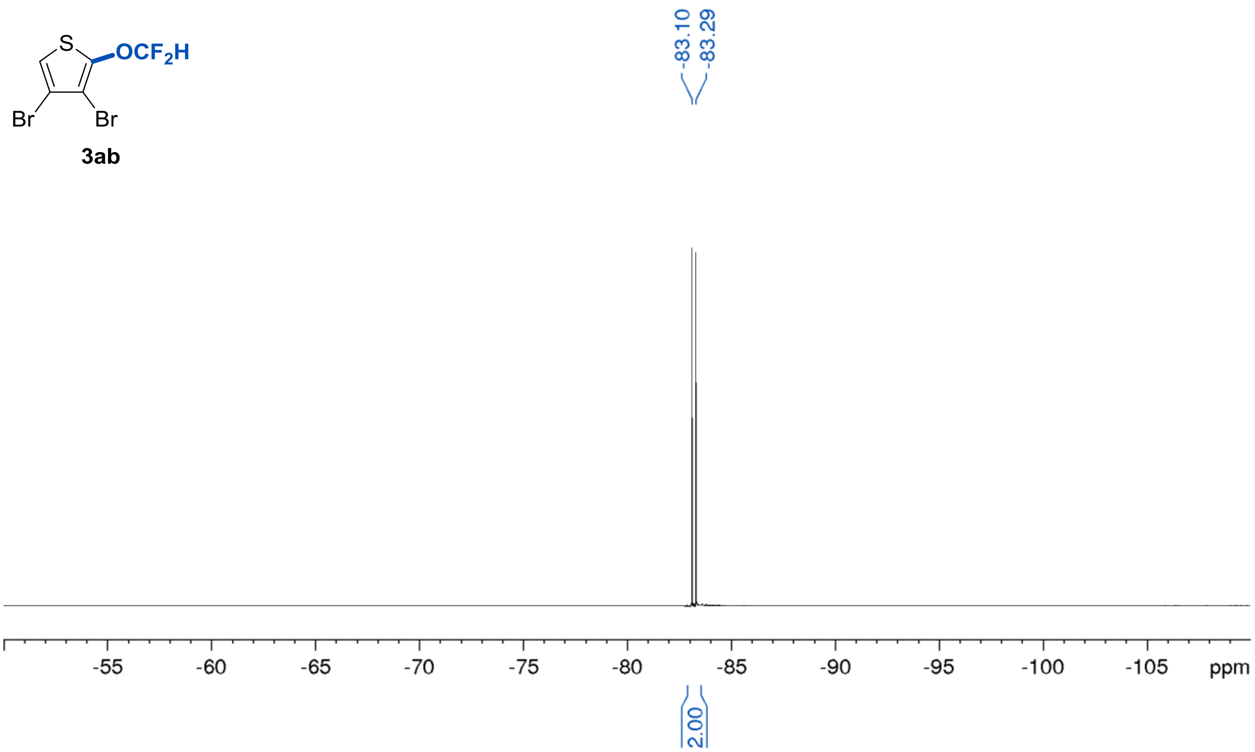

^{1}H NMR (700 MHz, CDCl$_3$, 25 °C) of 3ac

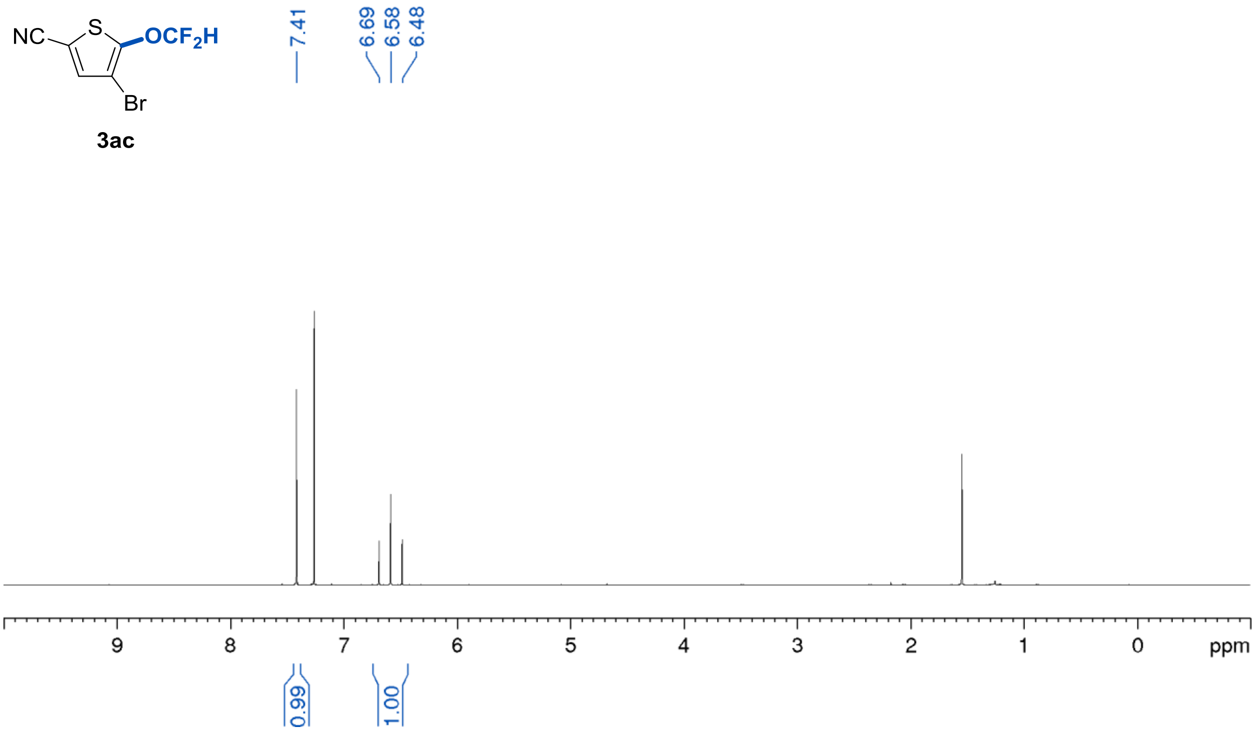

^{13}C NMR (175 MHz, $CDCl_3$, 25 ºC) of 3ac

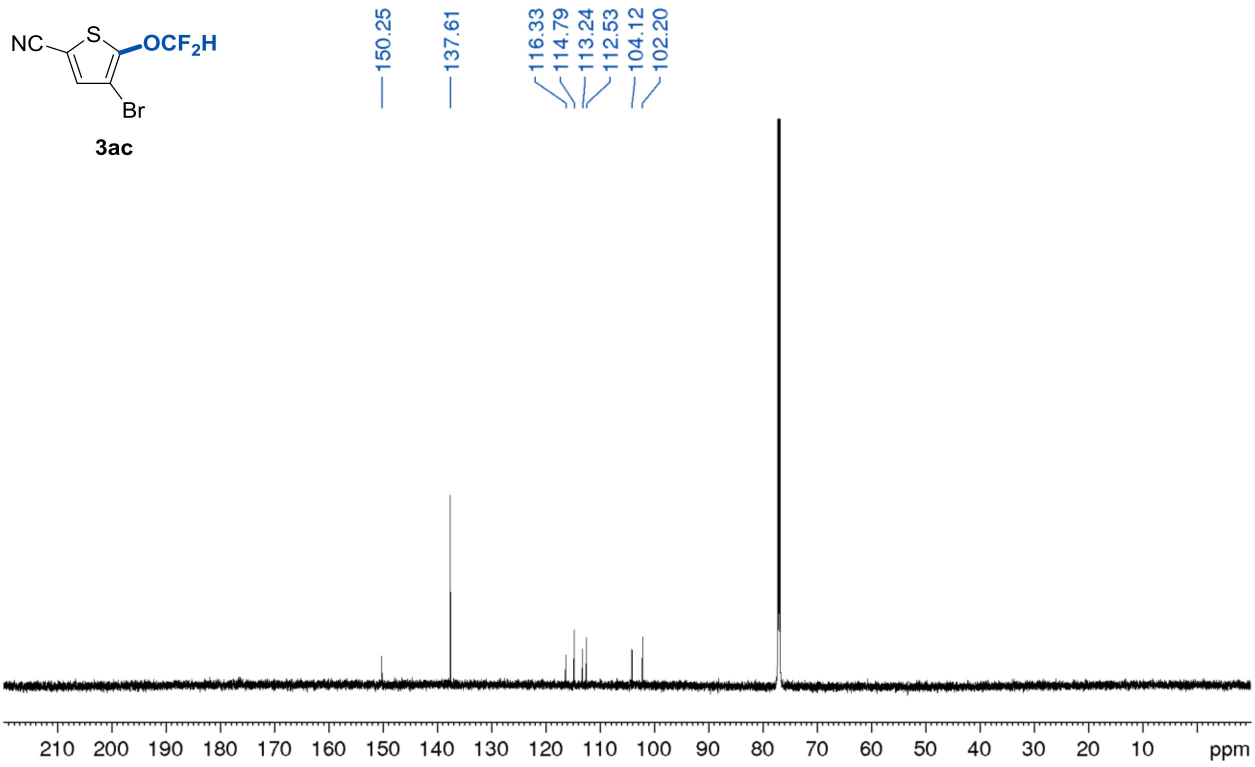

^{19}F NMR (376 MHz, $CDCl_3$, 25 ºC) of 3ac

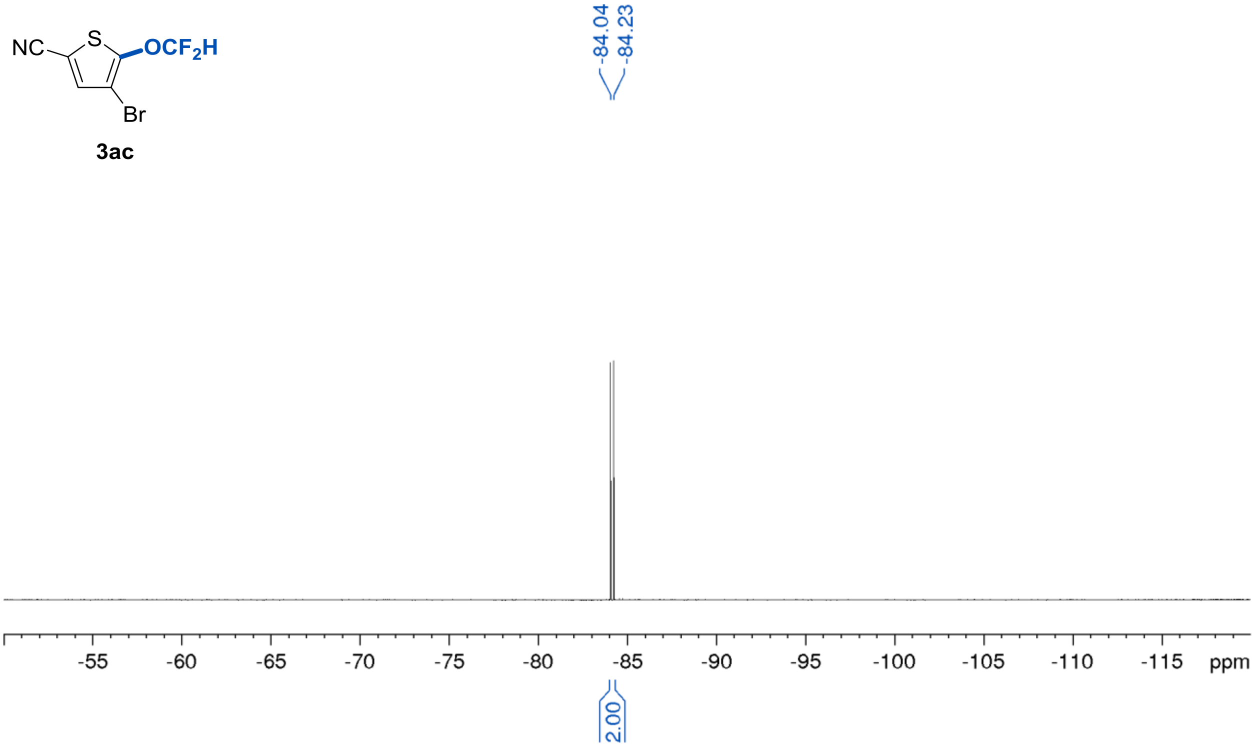

^{1}H NMR (700 MHz, $CDCl_3$, 25 ºC) of 3ac'

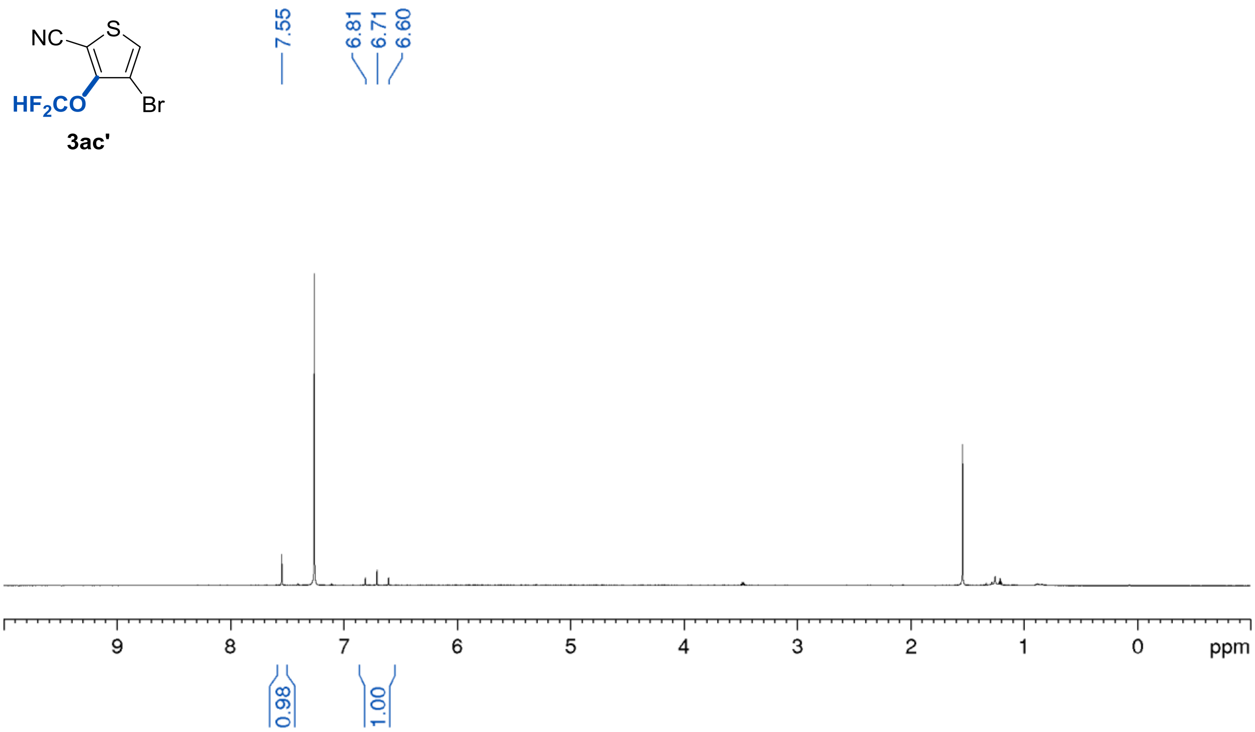

^{13}C NMR (175 MHz, $CDCl_3$, 25 ºC) of 3ac'

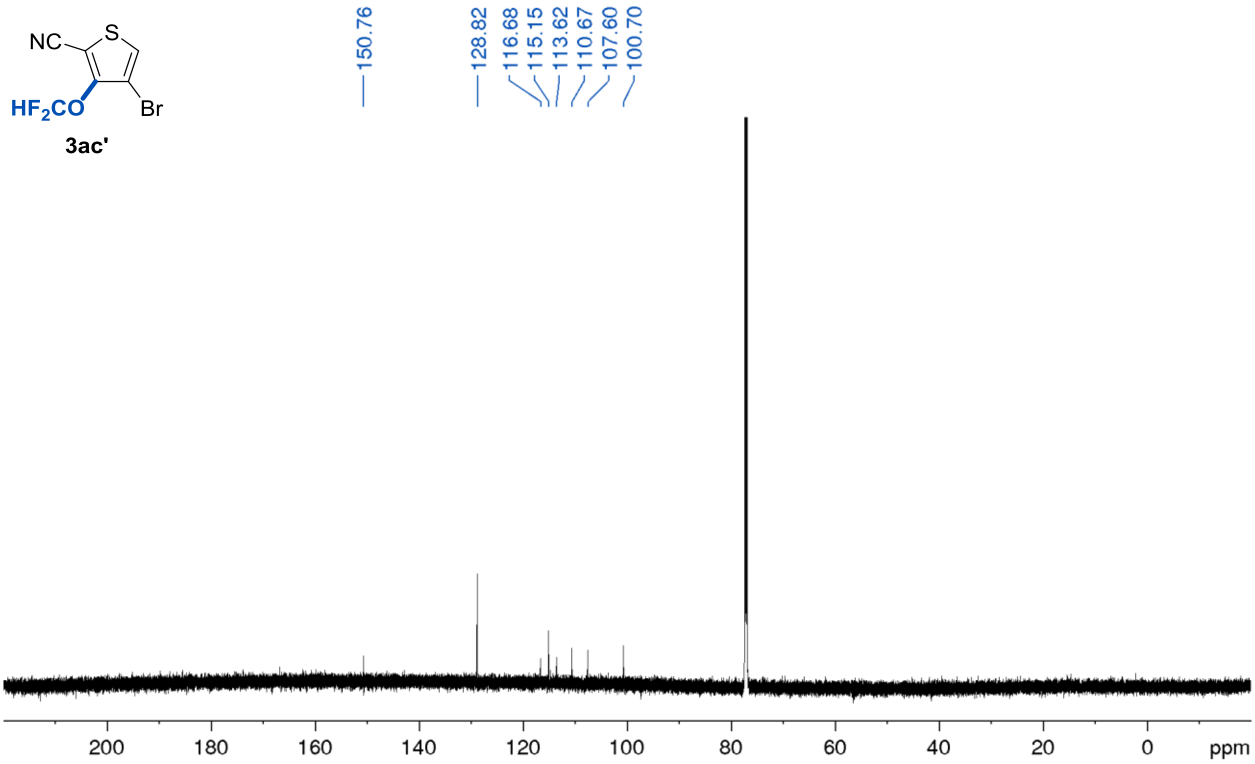

^{19}F NMR (376 MHz, $CDCl_3$, 25 ºC) of 3ac'

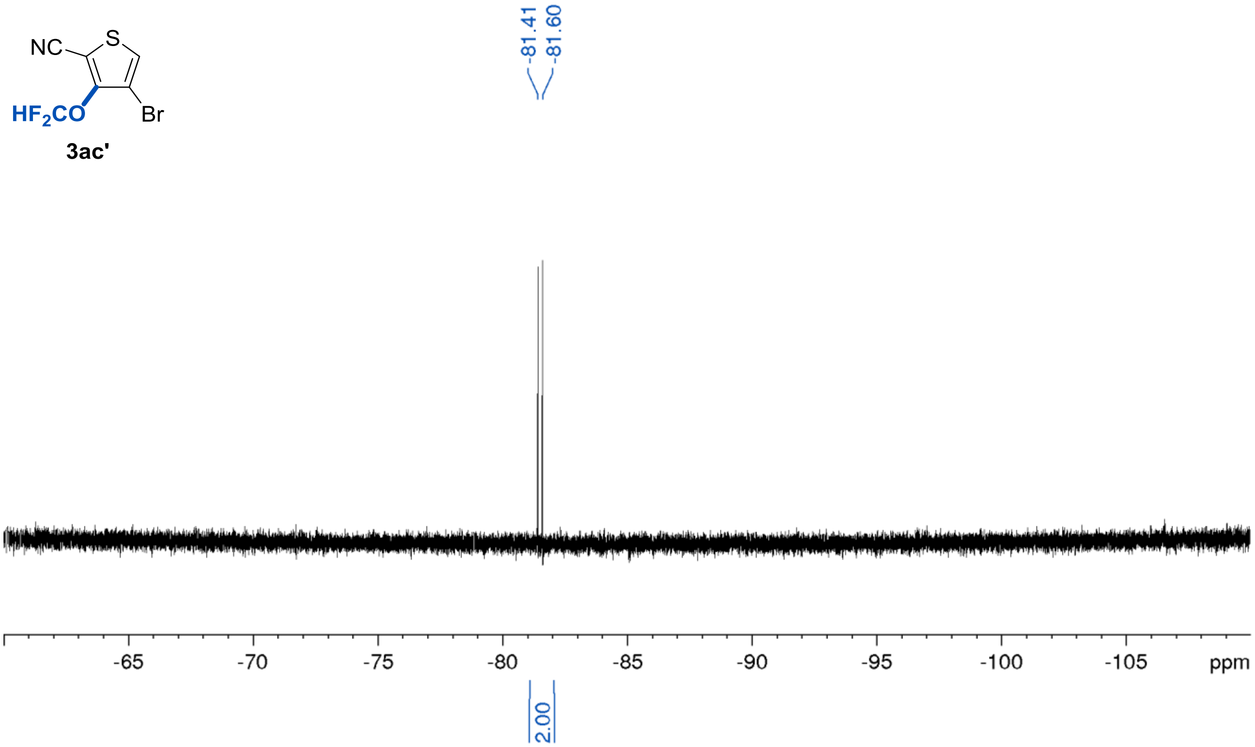

^{1}H NMR (700 MHz, $CDCl_3$, 25 ºC) of 3ad

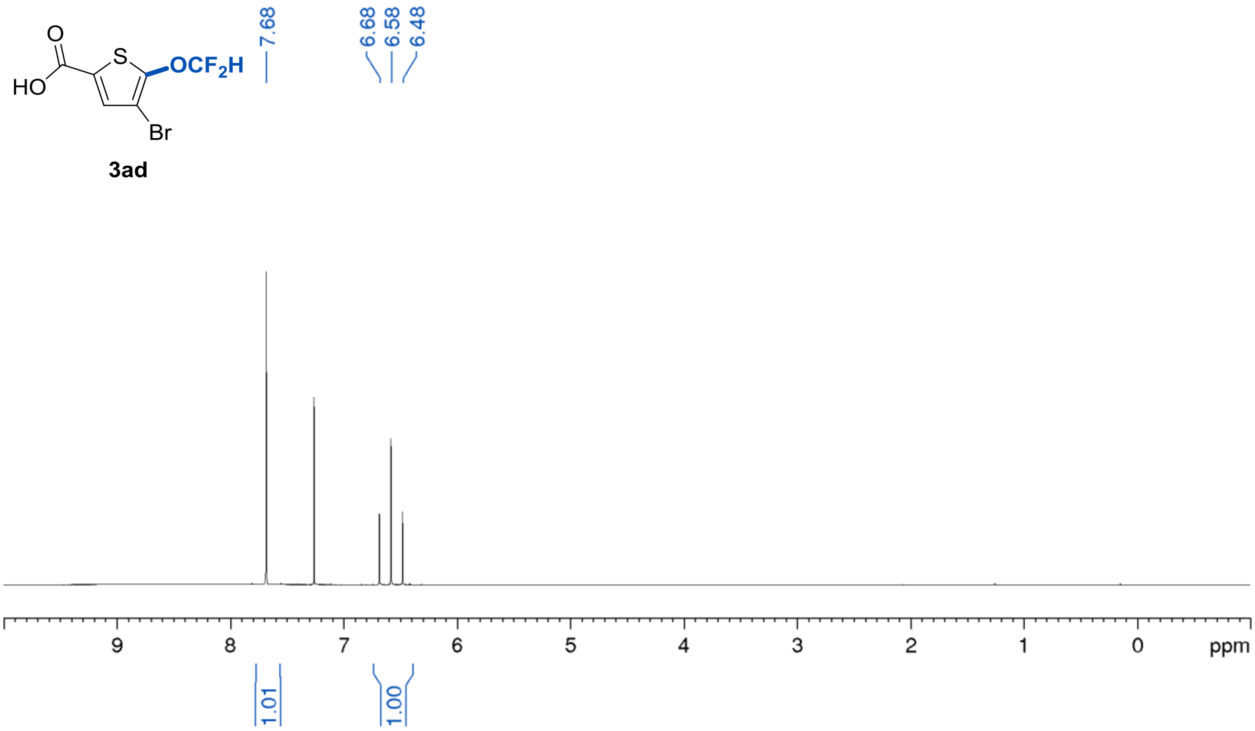

^{13}C NMR (175 MHz, $CDCl_3$, 25 ºC) of 3ad

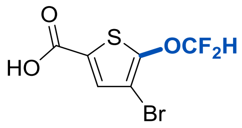

3ad

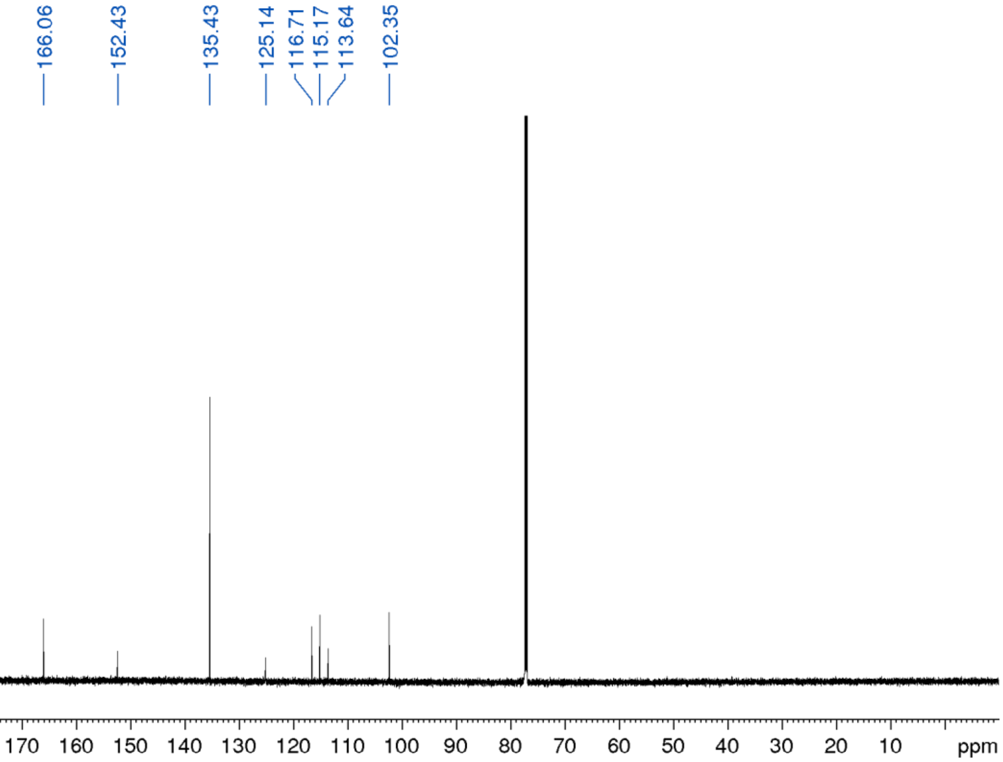

^{19}F NMR (376 MHz, $CDCl_3$, 25 ºC) of 3ad

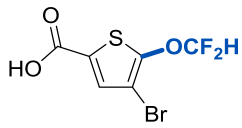

3ad

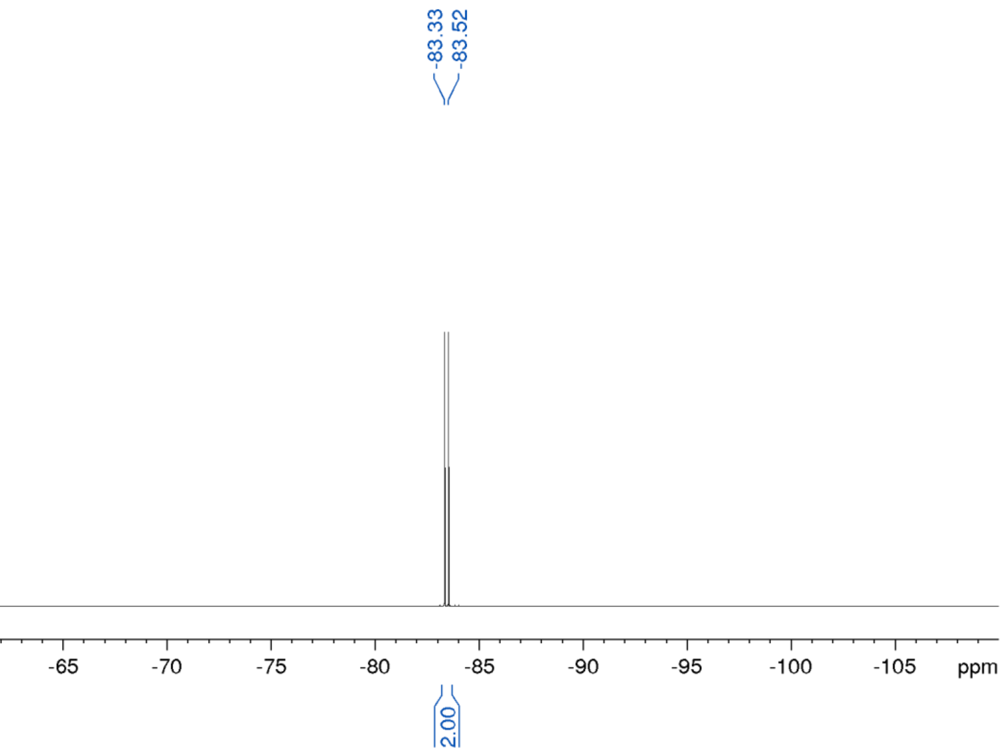

^{1}H NMR (700 MHz, $CDCl_3$, 25 ºC) of 3ad'

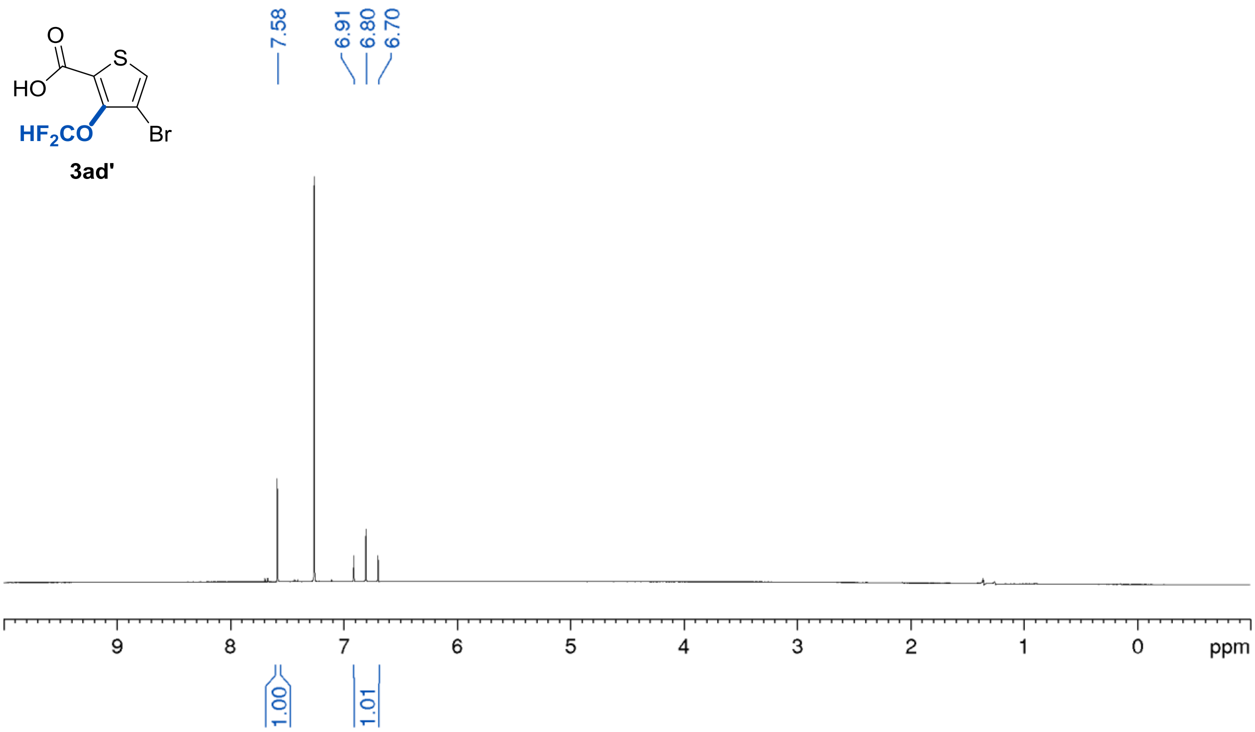

^{13}C NMR (175 MHz, $CDCl_3$, 25 ºC) of 3ad'

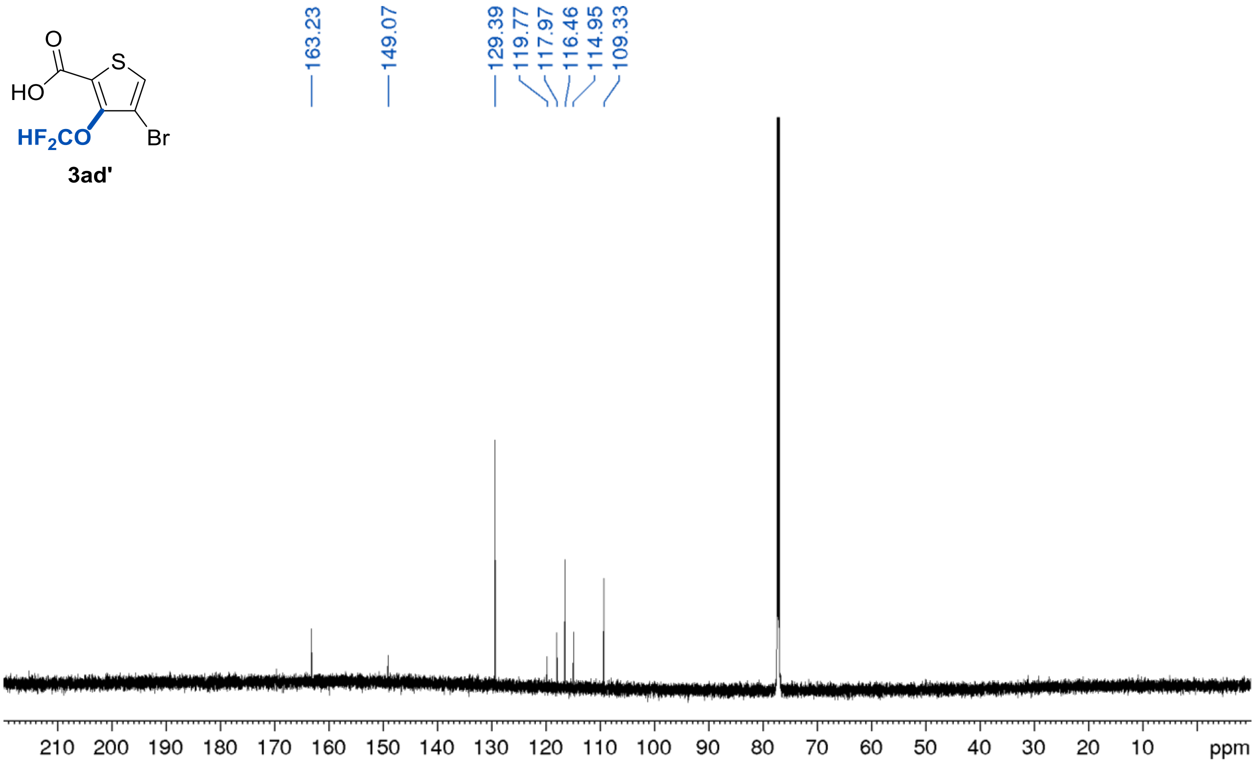

^{19}F NMR (376 MHz, CDCl$_3$, 25 ºC) of 3ad’

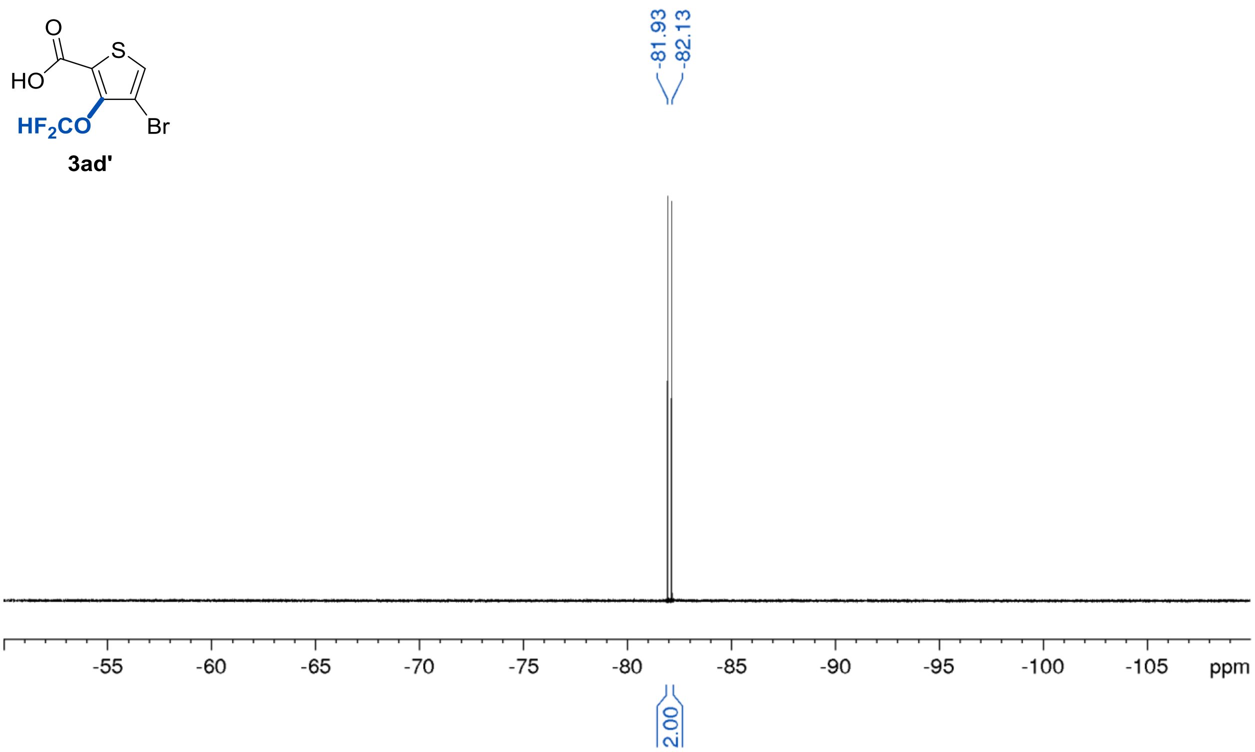

^{1}H NMR (700 MHz, CDCl$_3$, 25 ºC) of 5a’’ and 5a’’’

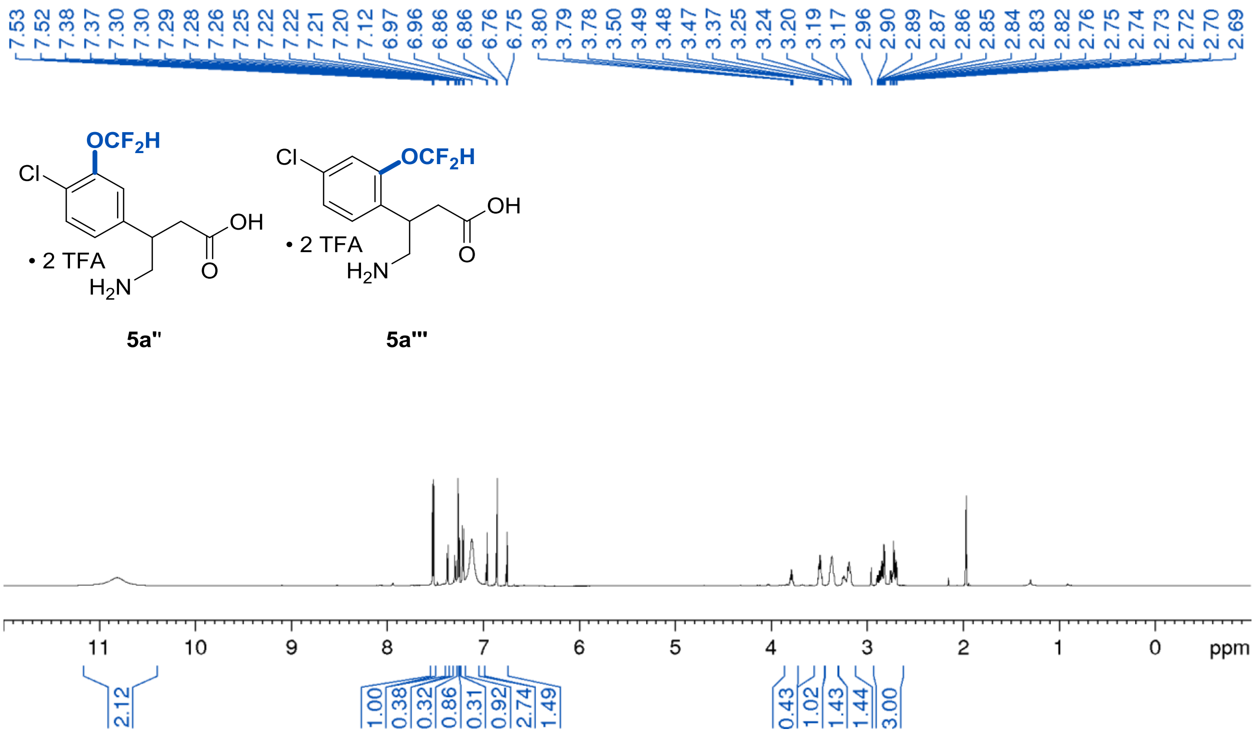

^{13}C NMR (175 MHz, CDCl$_3$, 25 ºC) of 5a’’ and 5a’’’

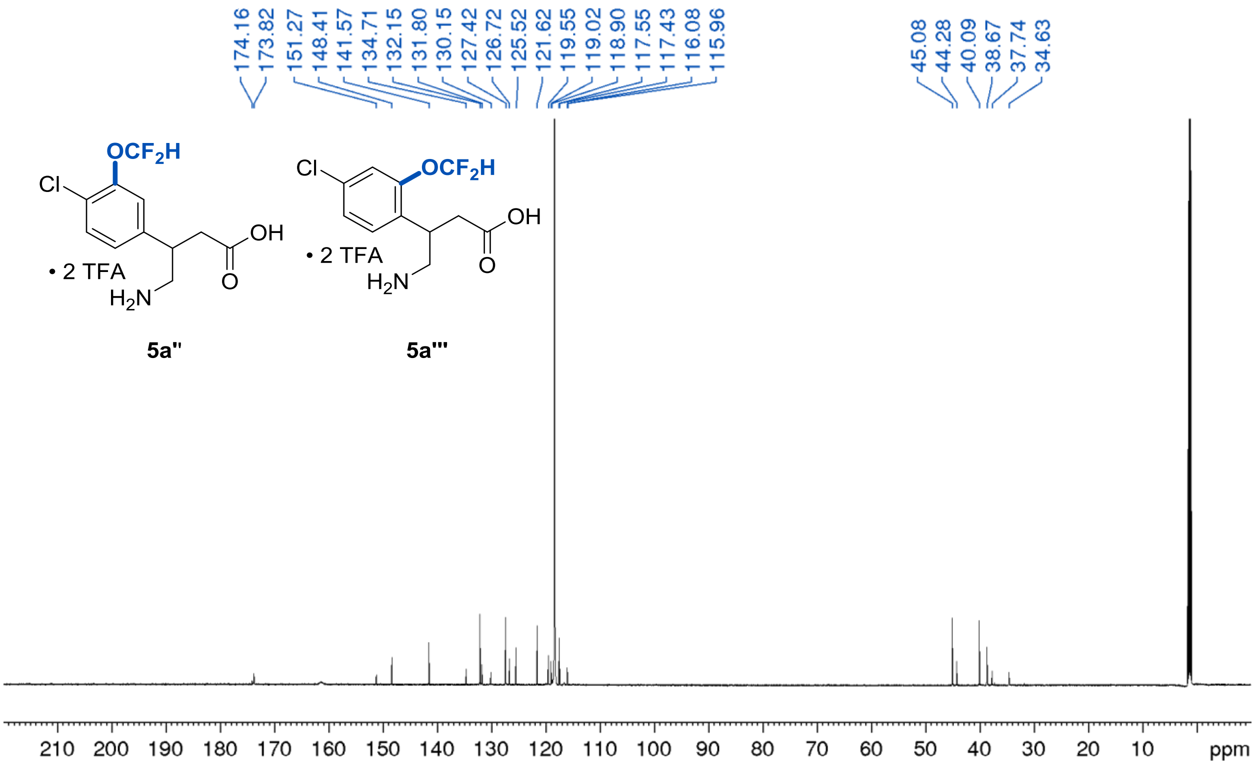

^{19}F NMR (376 MHz, CDCl$_3$, 25 ºC) of 5a’’ and 5a’’’

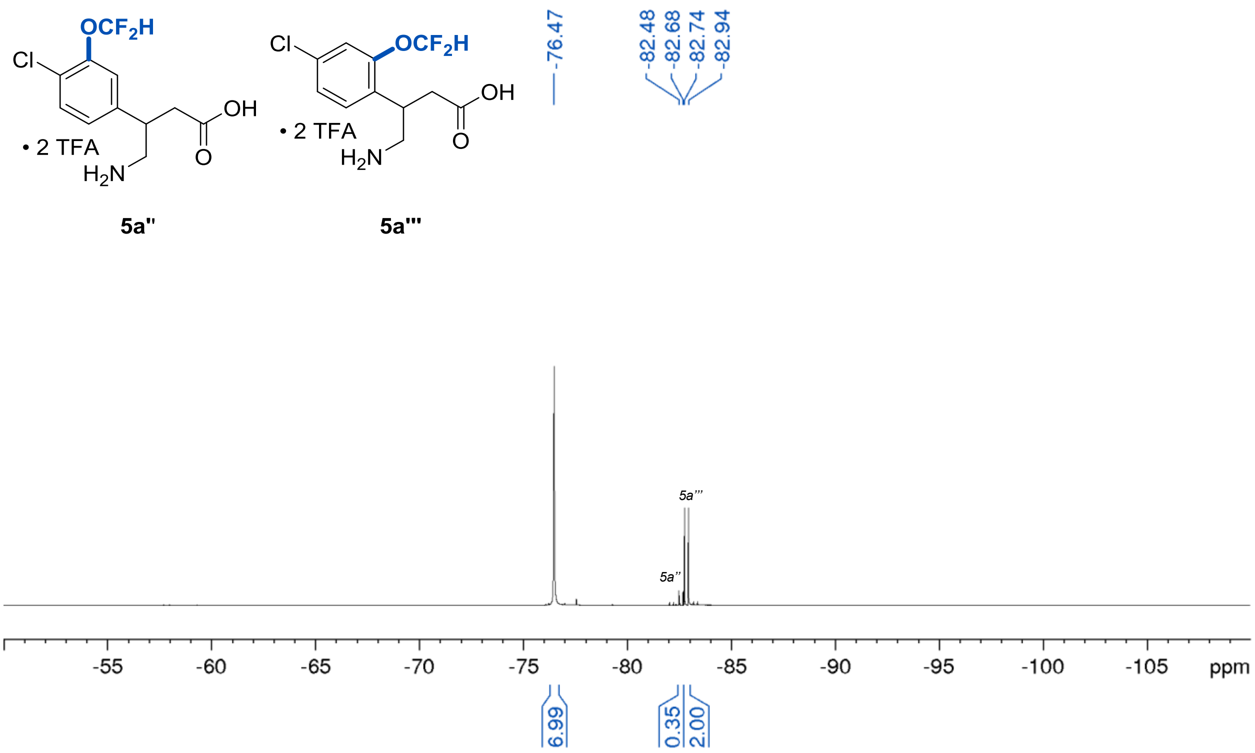

^{1}H NMR (700 MHz, CDCl$_3$, 25 °C) of 5b

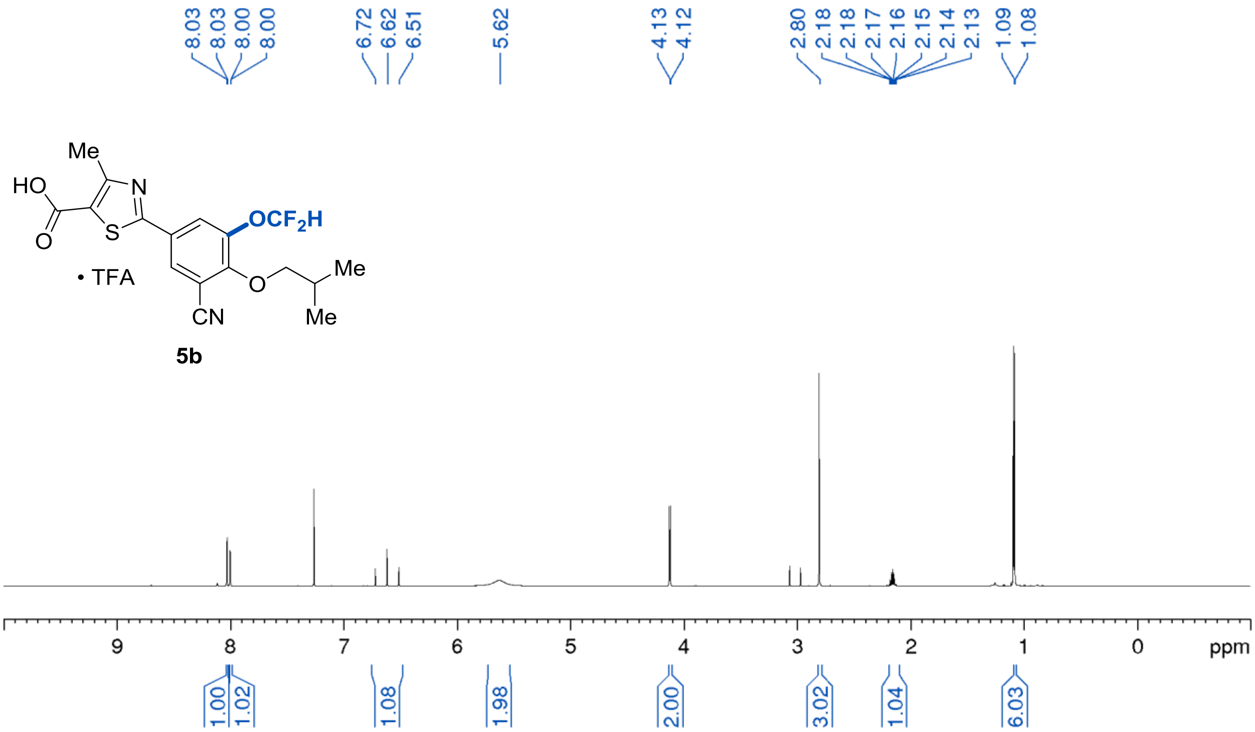

^{13}C NMR (175 MHz, CDCl$_3$, 25 °C) of 5b

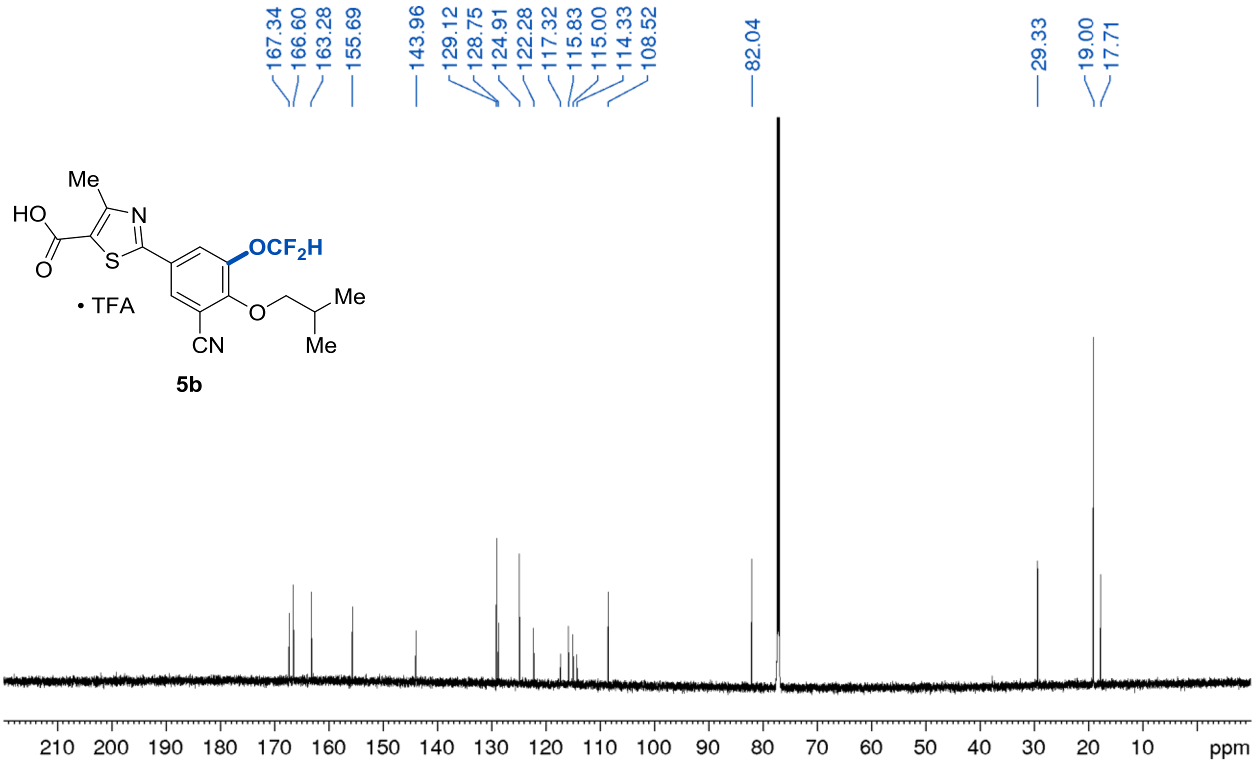

^{19}F NMR (376 MHz, $CDCl_3$, 25 °C) of 5b

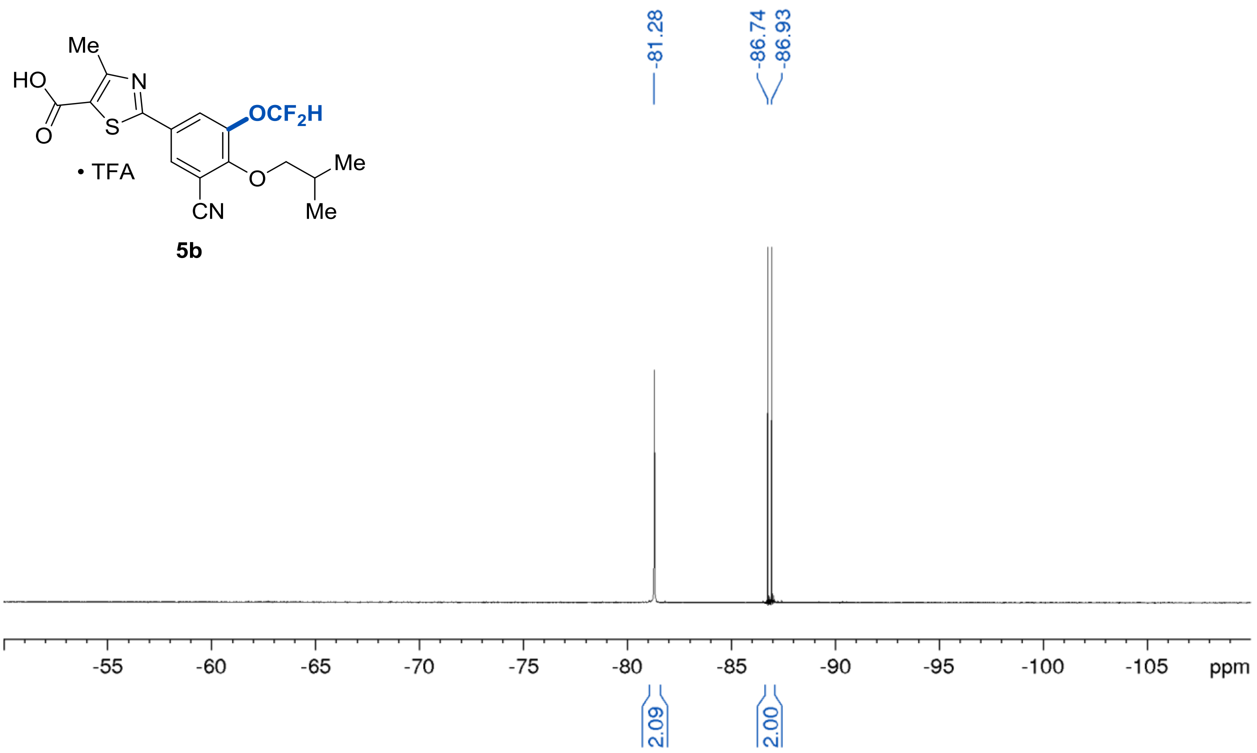

^{1}H NMR (700 MHz, $CDCl_3$, 25 °C) of 5c

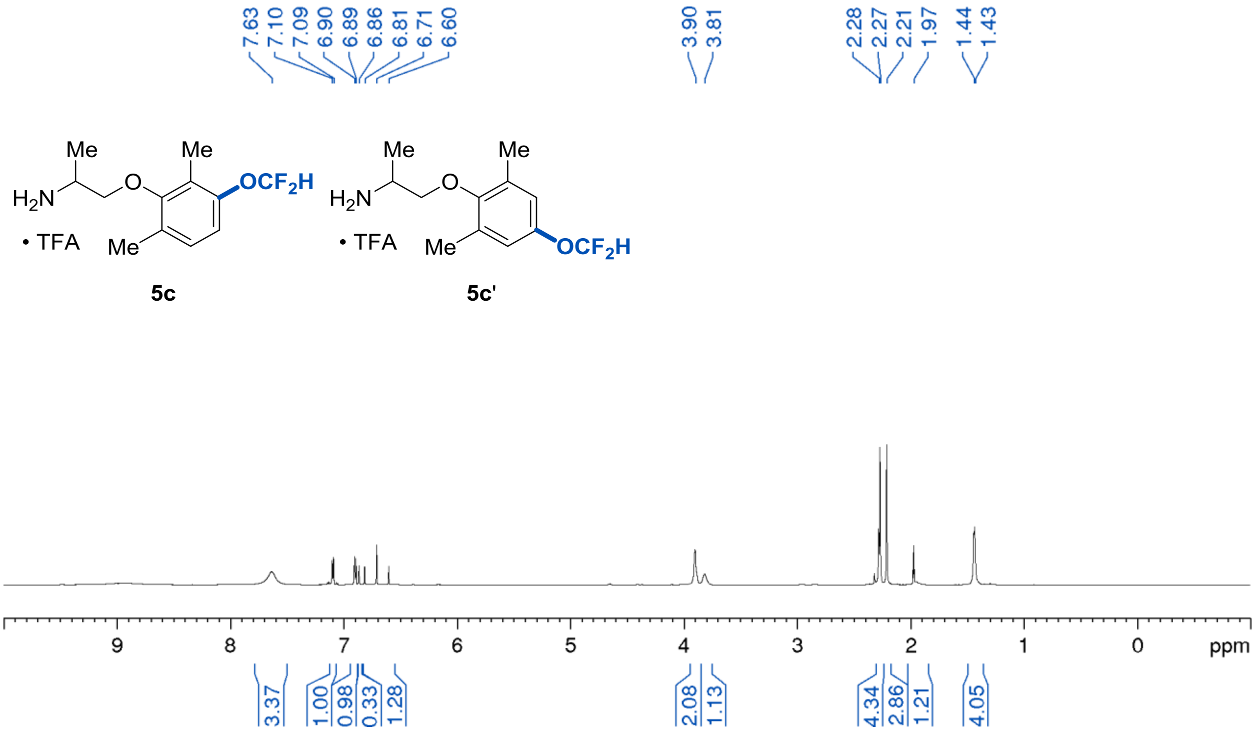

^{13}C NMR (175 MHz, $CDCl_3$, 25 ºC) of 5c

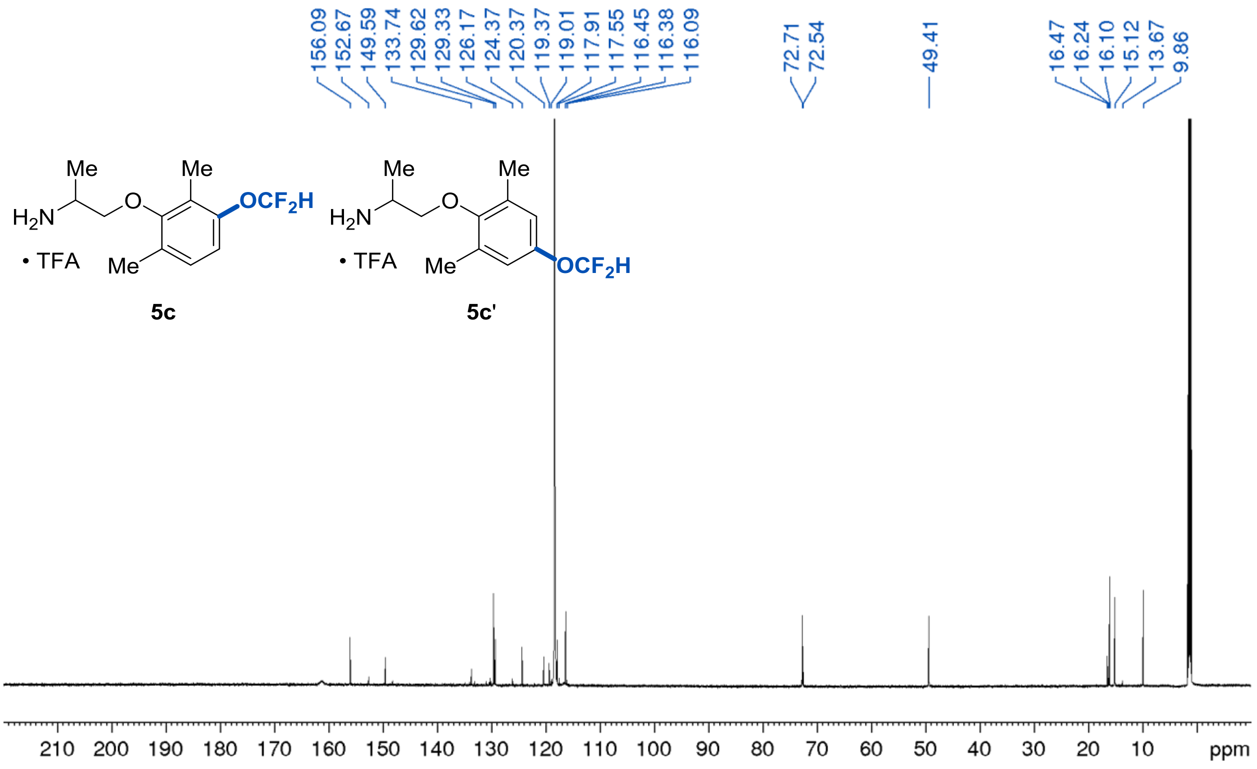

^{19}F NMR (376 MHz, $CDCl_3$, 25 ºC) of 5c

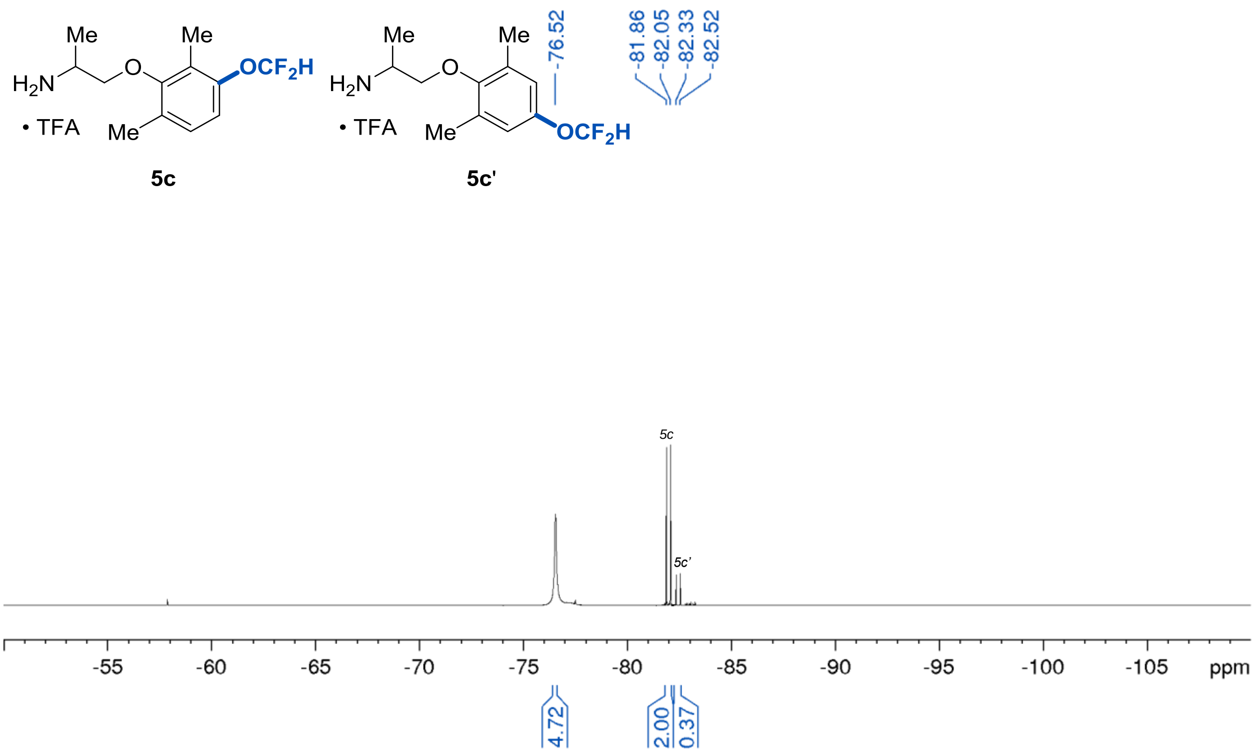

^{1}H NMR (700 MHz, $CDCl_3$, 25 °C) of 5d

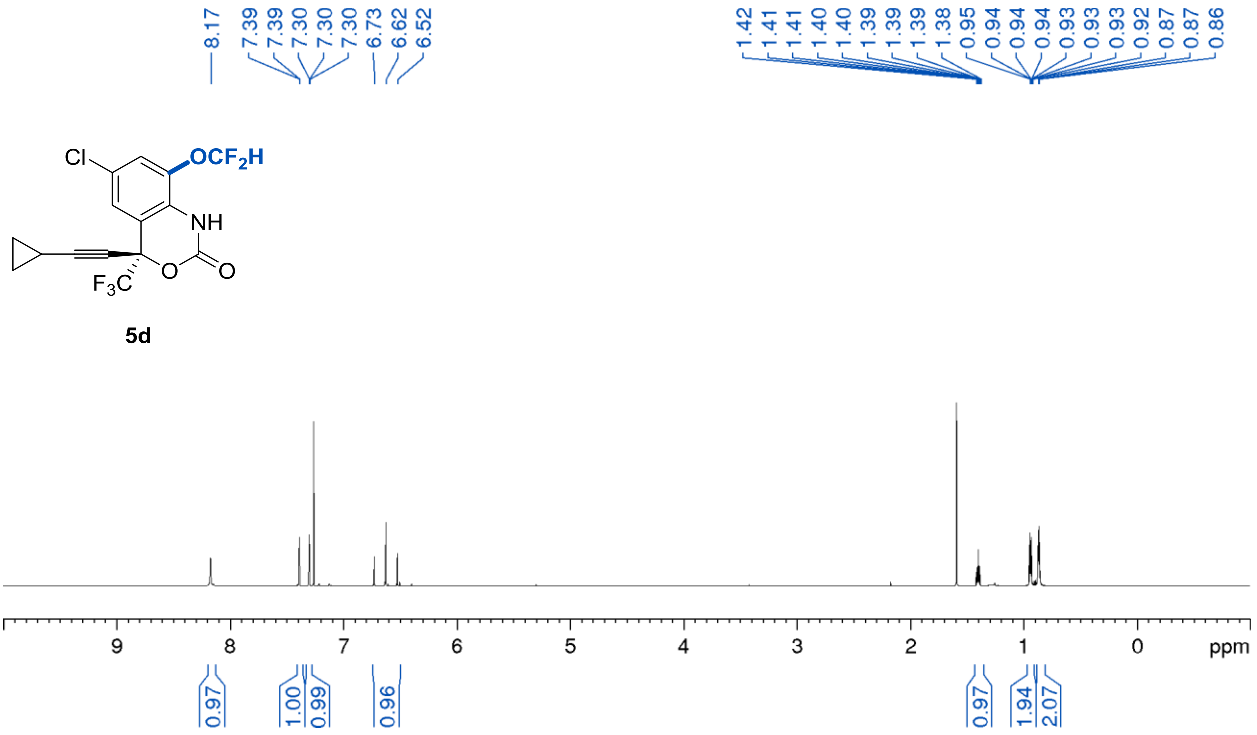

^{13}C NMR (175 MHz, $CDCl_3$, 25 °C) of 5d

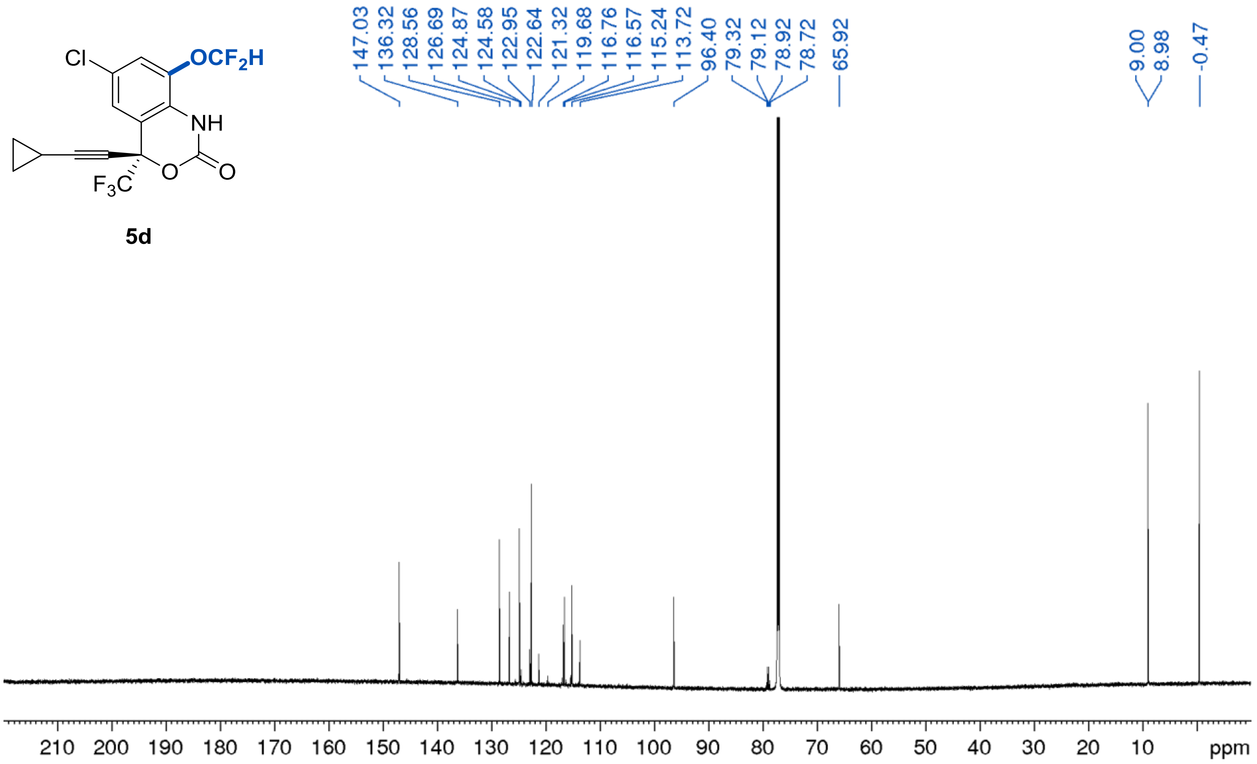

^{19}F NMR (376 MHz, $CDCl_3$, 25 °C) of 5d

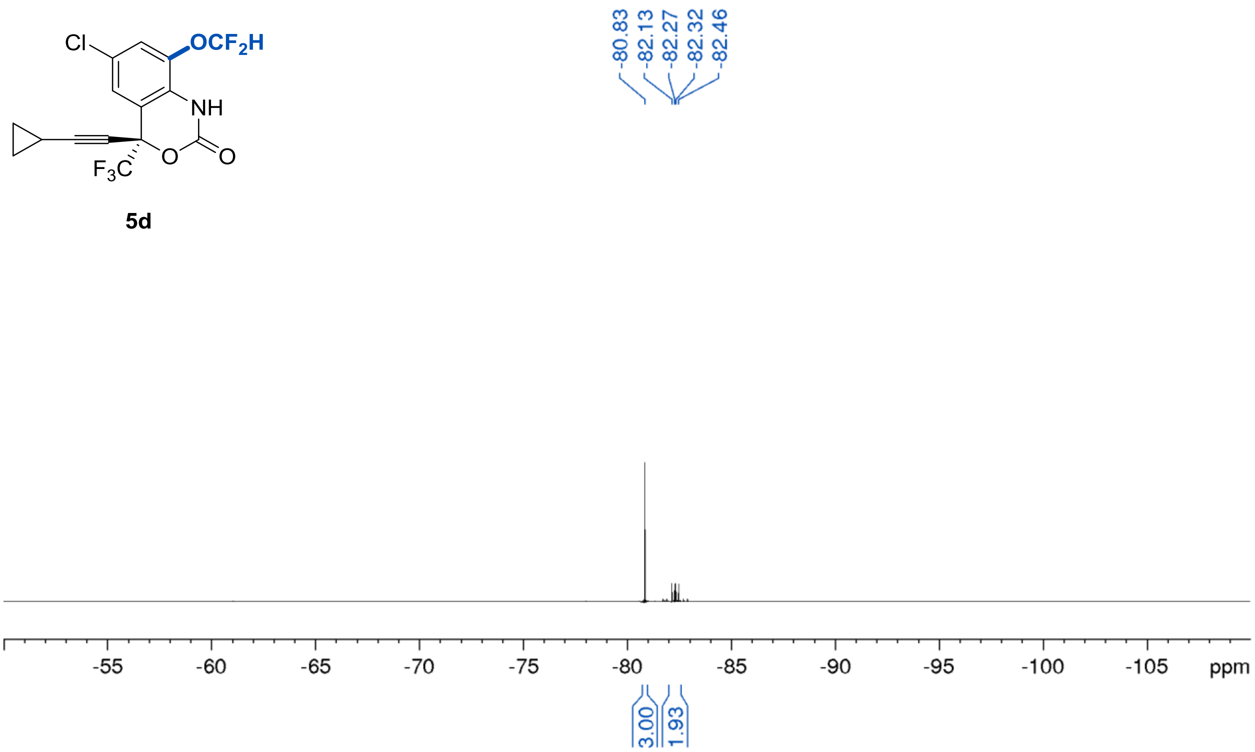

^{1}H NMR (700 MHz, $CDCl_3$, 25 °C) of 5e

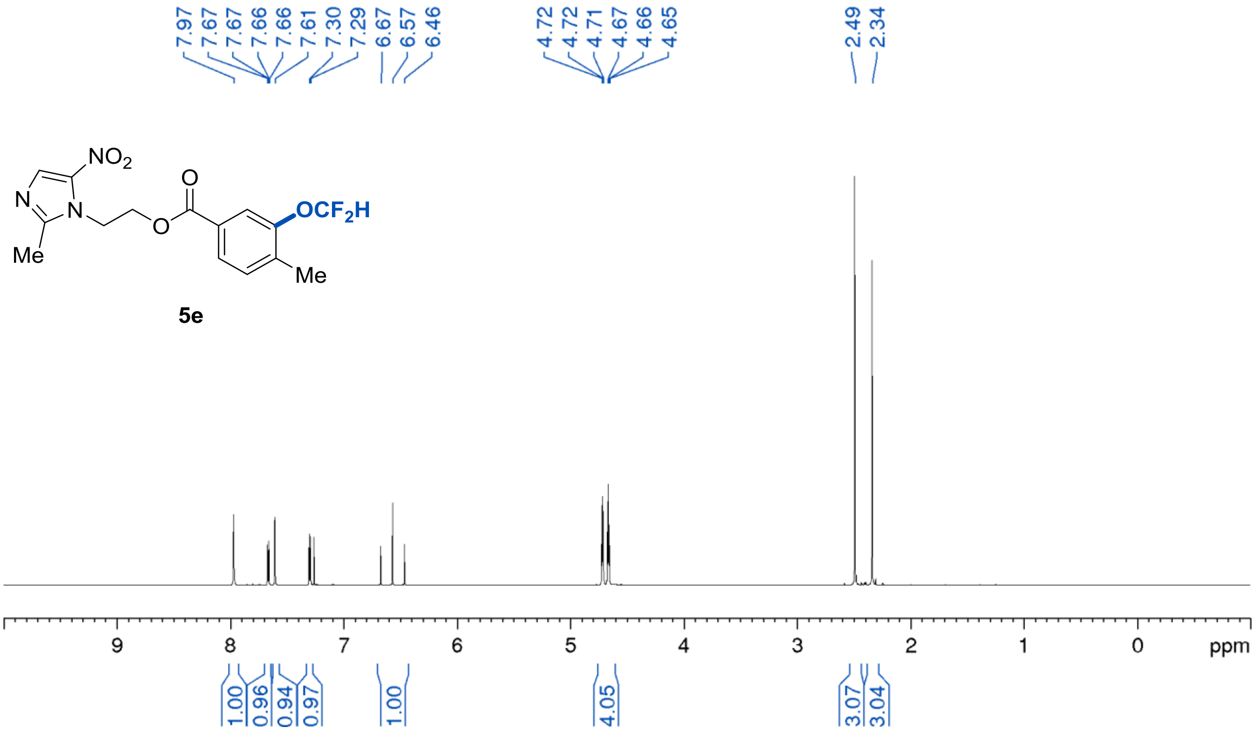

^{13}C NMR (175 MHz, $CDCl_3$, 25 °C) of 5e

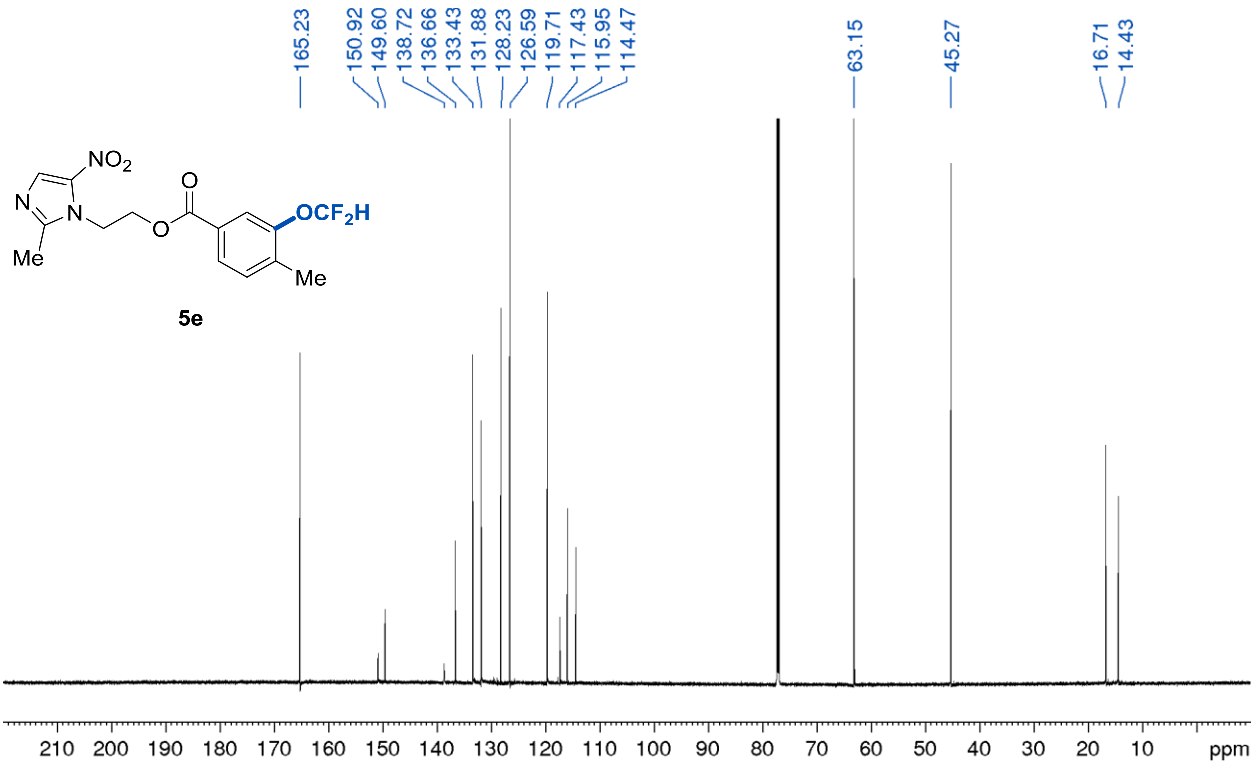

^{19}F NMR (376 MHz, $CDCl_3$, 25 °C) of 5e

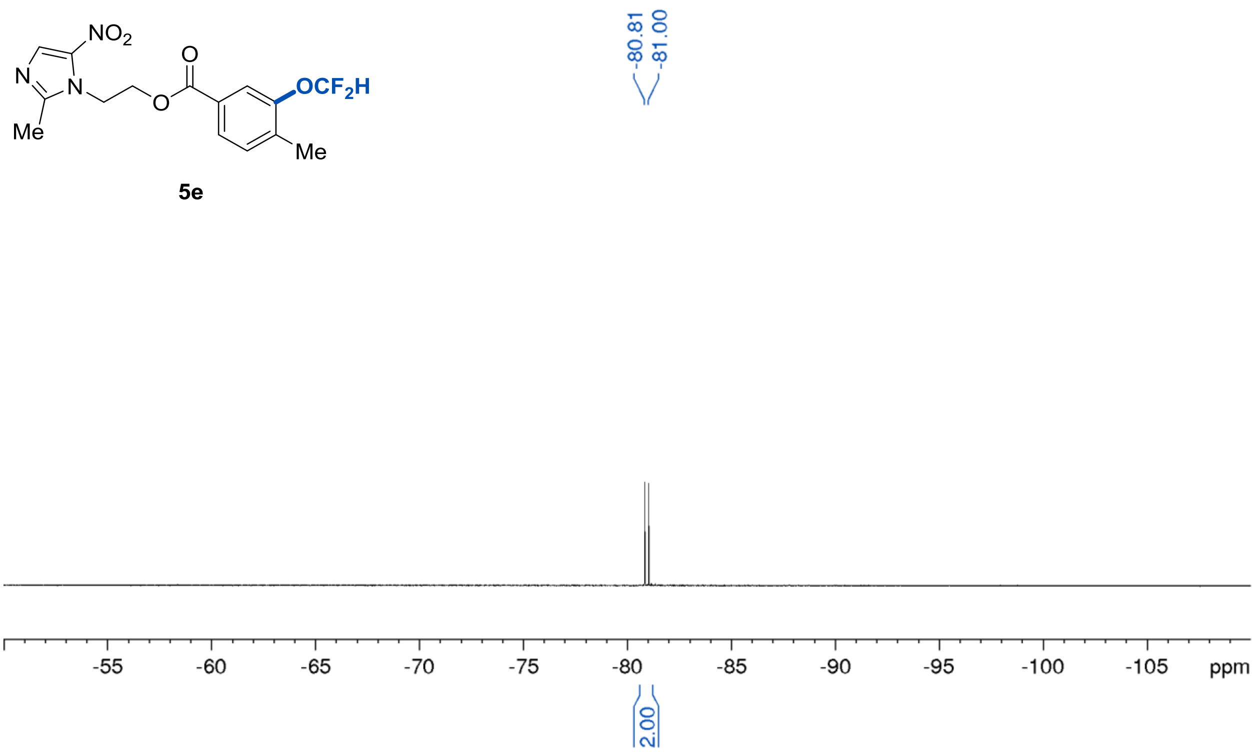

^{1}H NMR (700 MHz, $CDCl_3$, 25 ºC) of 5f

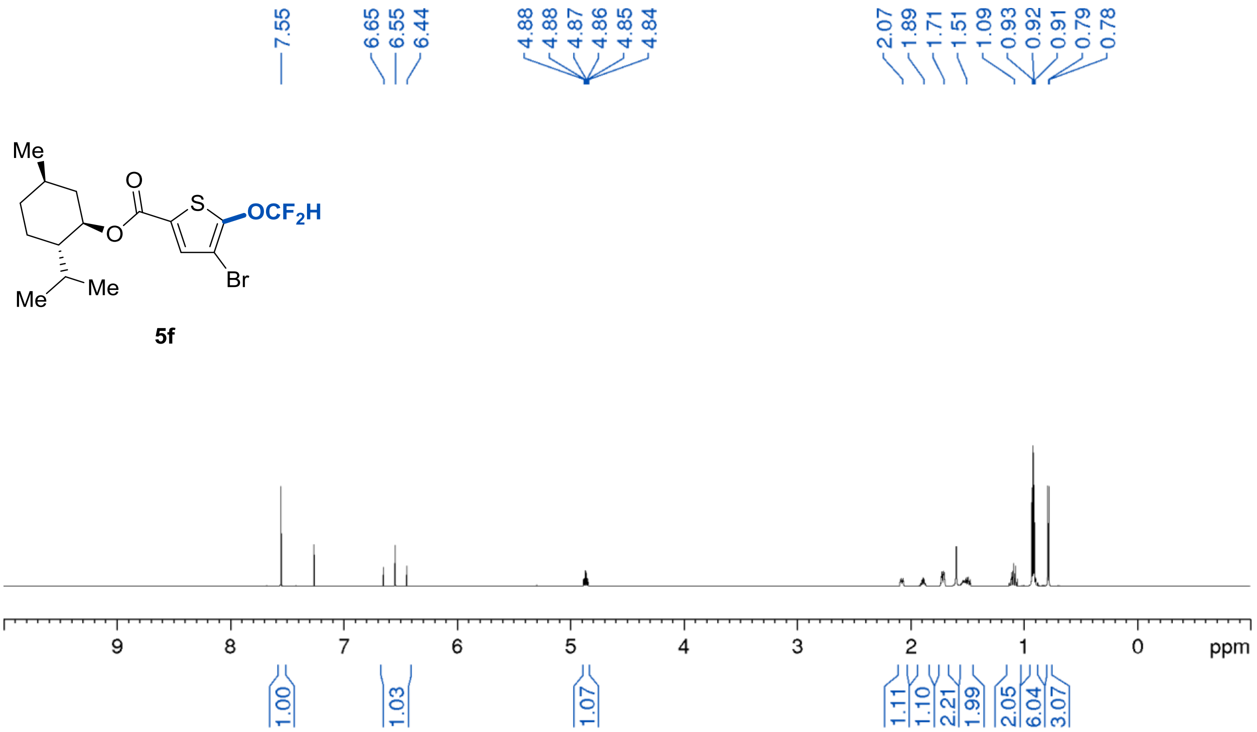

^{13}C NMR (175 MHz, $CDCl_3$, 25 ºC) of 5f

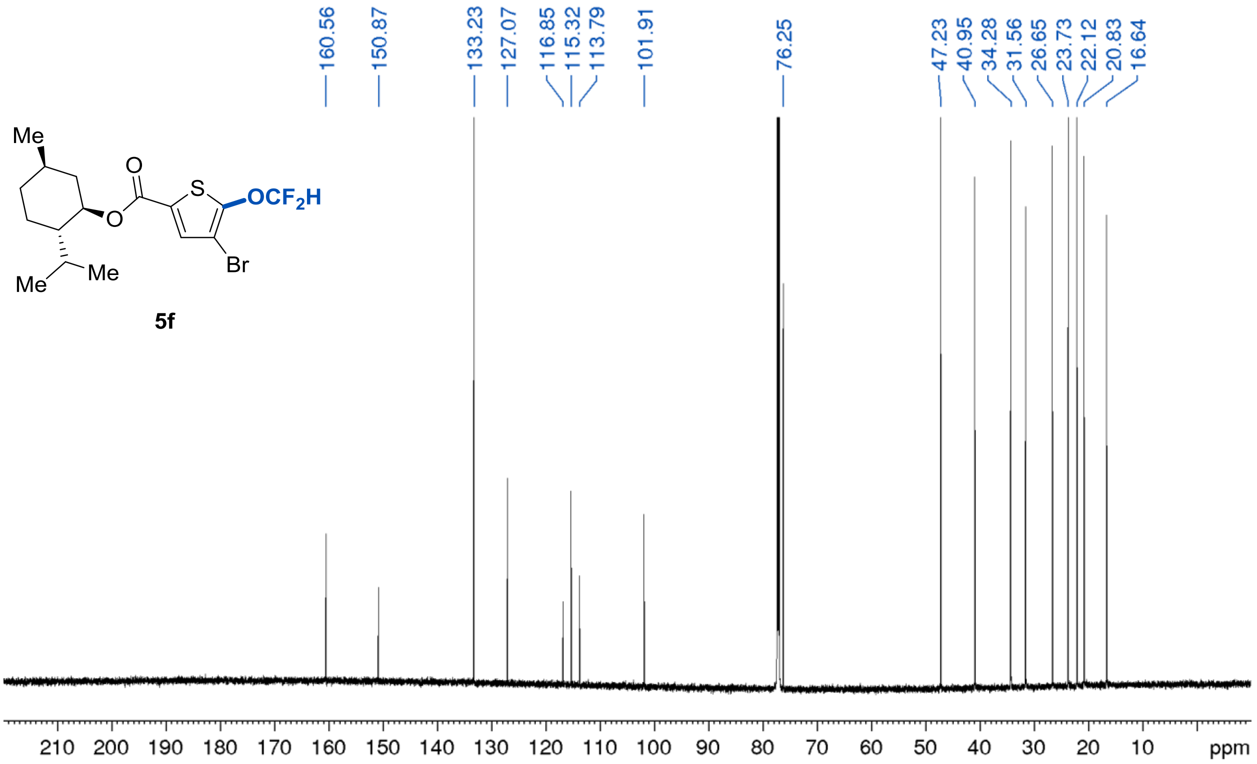

^{19}F NMR (376 MHz, $CDCl_3$, 25 ºC) of 5f

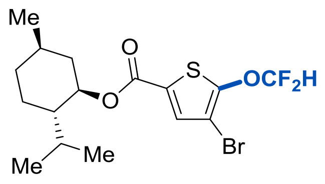

5f

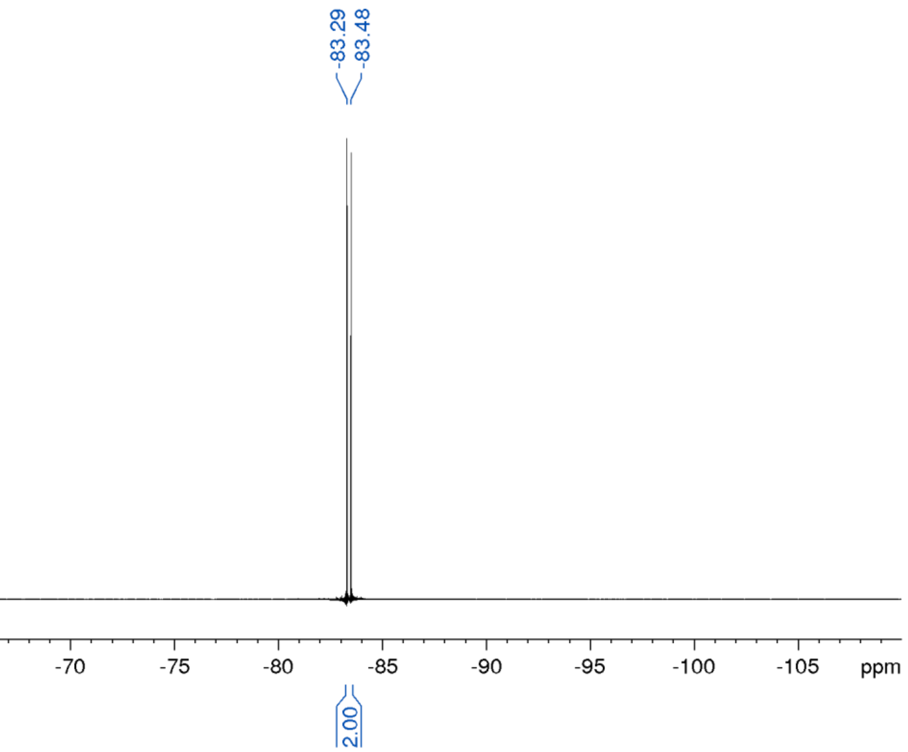

Cartesian Coordinates

1a
M06-2X/6-31+G(d) SCF energy in solution: -1289.58797247 a.u.
M06-2X/6-31+G(d) enthalpy: -1289.394592 a.u.
M06-2X/6-31+G(d) free energy: -1289.459986 a.u.
M06-2X/6-311++G(d,p) SCF energy in solution: -1290.02460582 a.u.
M06-2X/6-311++G(d,p) enthalpy: -1289.831225 a.u.
M06-2X/6-311++G(d,p) free energy: -1289.896619 a.u.

Cartesian coordinates

ATOM	X	Y	Z
C	0.268809	-0.733928	-0.226210
C	-0.772723	0.182262	-0.407668
C	-0.614920	1.572776	-0.372029
C	0.674167	1.972588	-0.136078
C	1.762187	1.091833	0.060366
C	1.582959	-0.271347	0.019249
H	-1.426334	2.278924	-0.512623
H	2.744937	1.515060	0.244619
N	0.945892	3.433630	-0.079757
O	2.089562	3.762384	0.145307
O	-0.002558	4.165662	-0.263383
C	2.744422	-1.214041	0.230097
F	3.870966	-0.556971	0.458815
F	2.501798	-2.024582	1.273052
F	2.914572	-1.993739	-0.849325
C	0.266760	-3.326556	-0.266067
H	1.023827	-3.429172	-1.042606
H	0.705508	-3.456779	0.722392
H	-0.548782	-4.030172	-0.424112
O	-3.118986	-0.206719	-0.911750
C	-3.899191	-0.015183	0.264511
H	-4.883589	0.293239	-0.083254
F	-3.306805	0.920038	1.014754
F	-3.928143	-1.145523	0.961187
N	-1.867739	-0.614738	-0.610132
N	-1.599319	-1.882637	-0.585732
N	-0.322550	-1.976988	-0.351404

1a’
M06-2X/6-31+G(d) SCF energy in solution: -1289.82029631 a.u.
M06-2X/6-31+G(d) enthalpy: -1289.629580 a.u.
M06-2X/6-31+G(d) free energy: -1289.696999 a.u.
M06-2X/6-311++G(d,p) SCF energy in solution: -1290.18141644 a.u.
M06-2X/6-311++G(d,p) enthalpy: -1289.990700 a.u.
M06-2X/6-311++G(d,p) free energy: -1290.058119 a.u.

Cartesian coordinates

ATOM	X	Y	Z
C	0.390364	-0.769971	-0.007415
C	-0.746259	0.059601	-0.035020
C	-0.694794	1.432168	-0.044568
C	0.589994	1.982924	-0.017888
C	1.745265	1.209079	0.030370
C	1.669649	-0.180185	0.035421
H	-1.581310	2.054264	-0.071226
H	2.708774	1.704262	0.062845
N	0.724923	3.438697	-0.032768
O	1.851059	3.905310	0.004969
O	-0.301651	4.095179	-0.082828
C	2.926677	-0.994961	0.068454
F	4.015915	-0.235741	0.214420
F	2.916071	-1.883332	1.083056
F	3.091294	-1.712598	-1.062970
C	0.629225	-3.317830	-0.054929
H	1.415233	-3.310224	-0.811048
H	1.059671	-3.517290	0.928333
H	-0.109914	-4.080512	-0.297679
O	-2.876607	-0.490569	-0.912622
C	-4.040887	-0.199399	-0.236386
H	-4.799037	0.009599	-0.990194
F	-3.866439	0.875646	0.564407
F	-4.419650	-1.217942	0.550957
N	-1.851313	-0.806423	0.001977
N	-1.441084	-2.113214	-0.126660
N	-0.079089	-2.047961	-0.053153

1a’’

M06-2X/6-31+G(d) SCF energy in solution: -976.39870255 a.u.
M06-2X/6-31+G(d) enthalpy: -976.240258 a.u.
M06-2X/6-31+G(d) free energy: -976.296296 a.u.
M06-2X/6-311++G(d,p) SCF energy in solution: -976.66470930 a.u.
M06-2X/6-311++G(d,p) enthalpy: -976.506265 a.u.
M06-2X/6-311++G(d,p) free energy: -976.562303 a.u.

Cartesian coordinates

ATOM	X	Y	Z
C	0.254019	1.754229	-0.005378
C	-0.723948	0.742216	0.000780
C	-0.349898	-0.621020	0.004372
C	0.997044	-0.911682	0.005432
C	1.949825	0.126744	0.001901
C	1.621292	1.464079	-0.005364
H	1.341664	-1.939154	0.008917
H	2.379229	2.239523	-0.009852
N	-1.893865	1.429643	-0.003131

N	-1.641267	2.761827	-0.008527
N	-0.381683	2.972653	-0.011507
C	-3.279002	0.984907	0.030665
H	-3.466263	0.276678	-0.776818
H	-3.504632	0.521507	0.992521
H	-3.889168	1.877344	-0.105827
C	-1.367575	-1.722934	-0.004350
F	-0.806439	-2.932927	0.036501
F	-2.136726	-1.672523	-1.111029
F	-2.205254	-1.627491	1.046943
N	3.374328	-0.245881	0.004921
O	4.189179	0.657727	0.000841
O	3.643547	-1.433521	0.011495

OCF2H_radical
M06-2X/6-31+G(d) SCF energy in solution: -313.43514990 a.u.
M06-2X/6-31+G(d) enthalpy: -313.406251 a.u.
M06-2X/6-31+G(d) free energy: -313.437411 a.u.
M06-2X/6-311++G(d,p) SCF energy in solution: -313.53404390 a.u.
M06-2X/6-311++G(d,p) enthalpy: -313.505145 a.u.
M06-2X/6-311++G(d,p) free energy: -313.536305 a.u.

Cartesian coordinates

ATOM	X	Y	Z
O	-1.333200	0.002832	-0.149182
C	-0.070439	0.000161	0.299345
F	0.622194	-1.085360	-0.111233
F	0.626835	1.082699	-0.111236
H	-0.153033	0.000326	1.399602

Benzene
M06-2X/6-31+G(d) SCF energy in solution: -232.14445297 a.u.
M06-2X/6-31+G(d) enthalpy: -232.037591 a.u.
M06-2X/6-31+G(d) free energy: -232.070371 a.u.
M06-2X/6-311++G(d,p) SCF energy in solution: -232.20789623 a.u.
M06-2X/6-311++G(d,p) enthalpy: -232.101034 a.u.
M06-2X/6-311++G(d,p) free energy: -232.133814 a.u.

Cartesian coordinates

ATOM	X	Y	Z
C	-1.278331	0.557449	0.000000
C	-1.121965	-0.828428	0.000008
C	0.156376	-1.385646	0.000003
C	1.278353	-0.557398	0.000002
C	1.121999	0.828383	-0.000001
C	-0.156431	1.385640	-0.000004
H	-2.273930	0.992147	-0.000005

H	-1.996126	-1.473350	-0.000023
H	0.278264	-2.465024	-0.000019
H	2.273897	-0.992221	-0.000015
H	1.996081	1.473411	0.000009
H	-0.278186	2.465033	0.000000

IV
M06-2X/6-31+G(d) SCF energy in solution: -644.83491975 a.u.
M06-2X/6-31+G(d) enthalpy: -644.704224 a.u.
M06-2X/6-31+G(d) free energy: -644.750926 a.u.
M06-2X/6-311++G(d,p) SCF energy in solution: -645.01968157 a.u.
M06-2X/6-311++G(d,p) enthalpy: -644.888986 a.u.
M06-2X/6-311++G(d,p) free energy: -644.935688 a.u.

Cartesian coordinates

ATOM	X	Y	Z
C	1.876734	-0.014045	0.107782
F	2.262449	-0.882155	-0.850094
F	2.750746	-0.089311	1.105086
F	1.974705	1.213797	-0.446628
O	0.654014	-0.271566	0.576192
C	-0.443753	-0.145653	-0.401017
C	-1.352928	-1.311031	-0.186024
C	-1.109891	1.182993	-0.234475
C	-2.671683	-1.151150	0.111509
C	-2.434044	1.290169	0.065682
H	-0.486682	2.063139	-0.360072
C	-3.245047	0.140147	0.239611
H	-3.296988	-2.026990	0.260069
H	-2.878560	2.274705	0.180115
H	-4.295942	0.246994	0.483848
H	0.024792	-0.217958	-1.394373
H	-0.906170	-2.296932	-0.272814

V
M06-2X/6-31+G(d) SCF energy in solution: -644.55809373 a.u.
M06-2X/6-31+G(d) enthalpy: -644.426211 a.u.
M06-2X/6-31+G(d) free energy: -644.471879 a.u.
M06-2X/6-311++G(d,p) SCF energy in solution: -644.83231804 a.u.
M06-2X/6-311++G(d,p) enthalpy: -644.700435 a.u.
M06-2X/6-311++G(d,p) free energy: -644.746103 a.u.

Cartesian coordinates

ATOM	X	Y	Z
C	-1.909598	-0.011210	-0.015936
F	-2.007185	-0.832025	1.034075
F	-2.927812	-0.176316	-0.817289

F	-1.893958	1.239910	0.461973
O	-0.763799	-0.270214	-0.725305
C	0.437971	-0.123154	-0.016950
C	1.307432	-1.310298	-0.110939
C	1.087700	1.199330	-0.125429
C	2.662840	-1.176241	0.015406
C	2.446105	1.300533	0.000123
H	0.442951	2.070106	-0.221202
C	3.220121	0.120384	0.065237
H	3.310479	-2.046172	0.022474
H	2.935602	2.268699	-0.005647
H	4.302107	0.215682	0.130285
H	0.247662	-0.149571	1.097813
H	0.816760	-2.277214	-0.199188

3a
M06-2X/6-31+G(d) SCF energy in solution: -644.29828548 a.u.
M06-2X/6-31+G(d) enthalpy: -644.177449 a.u.
M06-2X/6-31+G(d) free energy: -644.221966 a.u.
M06-2X/6-311++G(d,p) SCF energy in solution: -644.47905454 a.u.
M06-2X/6-311++G(d,p) enthalpy: -644.358218 a.u.
M06-2X/6-311++G(d,p) free energy: -644.402735 a.u.

Cartesian coordinates

ATOM	X	Y	Z
C	-1.805362	-0.000026	0.051008
F	-1.726592	-1.076573	0.847003
F	-2.994694	-0.000297	-0.537476
F	-1.726177	1.077641	0.845410
O	-0.861347	-0.000931	-0.912085
C	0.470654	-0.000367	-0.472706
C	1.118030	-1.215459	-0.292797
C	1.117277	1.215066	-0.293034
C	2.457344	-1.207897	0.091294
H	0.575501	-2.141162	-0.454900
C	2.456656	1.208233	0.091088
H	0.574409	2.140568	-0.455145
C	3.126066	0.000401	0.284568
H	2.977576	-2.149600	0.236920
H	2.976294	2.150276	0.236633
H	4.170167	0.000716	0.582219

1a’’’
M06-2X/6-31+G(d) SCF energy in solution: -976.03625086 a.u.
M06-2X/6-31+G(d) enthalpy: -975.880229 a.u.
M06-2X/6-31+G(d) free energy: -975.938059 a.u.
M06-2X/6-311++G(d,p) SCF energy in solution: -976.38630794 a.u.

M06-2X/6-311++G(d,p) enthalpy: -976.230286 a.u.
M06-2X/6-311++G(d,p) free energy: -976.288116 a.u.

Cartesian coordinates

ATOM	X	Y	Z
C	0.724192	0.738967	0.000008
C	-0.282914	1.755733	-0.000016
C	-1.619251	1.478922	0.000025
C	-1.940037	0.099560	-0.000005
C	-0.995253	-0.942962	-0.000043
C	0.349276	-0.649034	-0.000013
H	-2.394777	2.240013	-0.000002
H	-1.346309	-1.970801	-0.000069
N	-3.381626	-0.269655	0.000004
O	-3.633790	-1.453021	-0.000247
O	-4.162624	0.657037	0.000235
C	1.395677	-1.744737	0.000024
F	0.841094	-2.944224	-0.000252
F	2.173854	-1.621765	-1.082340
F	2.173458	-1.622101	1.082717
C	3.271170	0.995563	-0.000018
H	3.481995	0.404250	0.894309
H	3.482378	0.406099	-0.895497
H	3.853721	1.916203	0.001031
N	0.370385	3.002381	-0.000057
N	1.595806	2.826587	-0.000051
N	1.869935	1.393949	0.000022

OCF2H_anion
M06-2X/6-31+G(d) SCF energy in solution: -313.57090299 a.u.
M06-2X/6-31+G(d) enthalpy: -313.542510 a.u.
M06-2X/6-31+G(d) free energy: -313.573313 a.u.
M06-2X/6-311++G(d,p) SCF energy in solution: -313.76171206 a.u.
M06-2X/6-311++G(d,p) enthalpy: -313.733319 a.u.
M06-2X/6-311++G(d,p) free energy: -313.764122 a.u.

Cartesian coordinates

ATOM	X	Y	Z
O	0.004057	1.334260	-0.162402
C	0.000574	0.193587	0.315302
F	-1.119759	-0.655928	-0.111450
F	1.115766	-0.662654	-0.111439
H	0.000043	0.031641	1.413402
